# Solubilizer Tag Effect on PD-L1/Inhibitor Binding Properties for *m*-Terphenyl Derivatives

**DOI:** 10.1021/acsmedchemlett.3c00306

**Published:** 2023-12-14

**Authors:** Ewa Surmiak, Julia Ząber, Jacek Plewka, Grzegorz Wojtanowicz, Justyna Kocik-Krol, Oskar Kruc, Damian Muszak, Ismael Rodríguez, Bogdan Musielak, Monica Viviano, Sabrina Castellano, Lukasz Skalniak, Katarzyna Magiera-Mularz, Tad A. Holak, Justyna Kalinowska-Tłuścik

**Affiliations:** † Faculty of Chemistry, Jagiellonian University, Gronostajowa 2, 30-387 Cracow, Poland; ‡ Doctoral School of Exact and Natural Sciences, Jagiellonian University, Łojasiewicza 11, 30-348 Cracow, Poland; § Department of Pharmacy, University of Salerno, Via Giovanni Paolo II, 84085 Fisciano, Italy

**Keywords:** PD-L1, Immune checkpoint, Small-molecule inhibitor, Cancer, *m*-Terphenyl

## Abstract

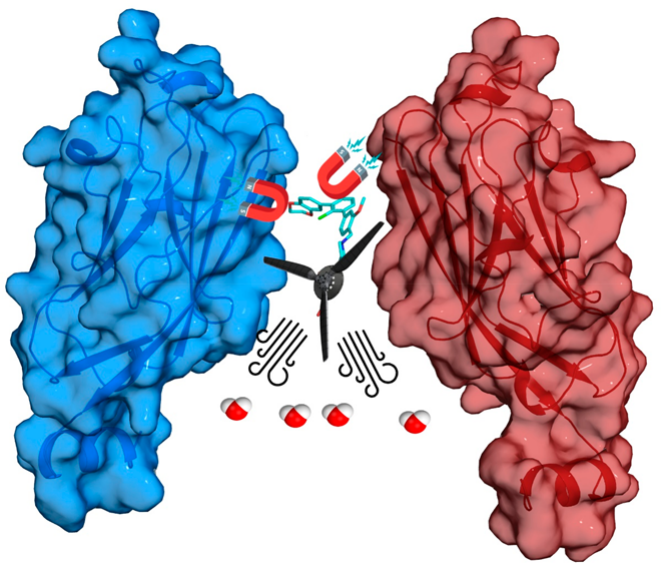

Although heavily studied, the subject of anti-PD-L1 small-molecule inhibitors is still elusive. Here we present a systematic overview of the principles behind successful anti-PD-L1 small-molecule inhibitor design on the example of the *m*-terphenyl scaffold, with a particular focus on the neglected influence of the solubilizer tag on the overall affinity toward PD-L1. The inhibitor developed according to the proposed guidelines was characterized through its potency in blocking PD-1/PD-L1 complex formation in homogeneous time-resolved fluorescence and cell-based assays. The affinity is also explained based on the crystal structure of the inhibitor itself and its costructure with PD-L1 as well as a molecular modeling study. Our results structuralize the knowledge related to the strong pharmacophore feature of the *m*-terphenyl scaffold preferential geometry and the more complex role of the solubilizer tag in PD-L1 homodimer stabilization.

Programmed cell death protein 1 (PD-1) is a 55 kDa transmembrane protein constituted of an IgV-like N-terminal extracellular domain, a transmembrane domain, and a cytoplasmic domain. PD-1 is mostly expressed on the surfaces of T cells, natural killer cells, and B cells. PD-1 binds to two natural ligands, PD-L1 and PD-L2, both of which are transmembrane proteins belonging to the immunoglobulin superfamily. In a healthy system, PD-1 engagement by its natural ligands inhibits a T-cell response, resulting in reduced effector functions, leading to cancer cell protection but also chronic infections and decreased autoimmunity.^1,2^

Dysfunctions of the regulatory effect of the PD-1/PD-L1 checkpoint toward the immune system can lead to several diseases related to autoimmunity, infections, and cancer.^3,4^ In cancer cells, overexpression of PD-L1 leads to the progression of T cells into an exhausted state and decreased tumor cell apoptosis. Disruption of the PD-1/PD-L1 interaction leads to the reactivation of T cells, laying the foundations for cancer treatments coined.^5−9^ Since its discovery, the PD-1/PD-L1 blockade has proven to be an efficient treatment of several cancer types, such as nonsmall cell lung cancer, Hodgkin lymphoma, breast cancer, *etc.*(10,11) Currently, all clinically approved anti-PD-1/PD-L1 therapeutics rely on highly selective monoclonal antibodies (mAbs), such as nivolumab against PD-1 and durvalumab against PD-L1.^6,12^ Despite their superb efficacy, there is a constant urge to develop alternative therapeutic classes, overcoming the limitations of mAbs related to their poor pharmacokinetic profile, high manufacturing costs, oral unavailability, and observed adverse effects.^13,14^ Those additional classes comprise mainly small-molecule inhibitors (SMIs) and macrocyclic peptides.^15−20^ Moreover, several studies have shown that synergistic effects were observed in therapies combining SMIs and mAbs, leading to a new wave of anti-PD-L1-oriented therapies.^21^

Although several postulated and promising SMIs have been designed and tested, only a few reached the clinical trials stage, while to date none have been approved for cancer treatment. Among all the discovered putative small-molecule drugs, the most promising results have been shown for PD-L1 binding inhibitors belonging to macrocyclic peptides and peptidomimetics.^22−24^ Among the small-molecule inhibitors, the first class and the most significant breakthrough were compounds based on a biphenyl core, disclosed by Bristol Myers Squibb in 2015.^25,26^ Since then, the biphenyl-core structures have been highly developed, with the PD-1/PD-L1 complex inhibition results reaching up to the nanomolar scale.^22,23,27^ However, except for the *C*_2_-symmetric structures, such as the most prominent **compound A** presented by Park *et al.* in 2021,^28^ this class of compounds still lacks the level of activity displayed by mAbs in the *in vitro* assays.^29^

Despite the biphenyl core being a well-established and crucial pharmacophore fragment of anti-PD-L1-active SMI agents, its solubility remains a challenge. Thus, several solubilizing tags were tested to modulate the designed molecule’s physicochemical properties.^16,18^ Nevertheless, the function of these molecular fragments (apart from increasing the compound solubility) in ligand–protein complex stabilization is poorly understood. The question arises whether it is possible to rationally design the solubilizer tag to increase the ligand’s affinity and anti-PD-L1 activity by allowing additional protein–ligand interaction formation. Herein we present a systematic structure–activity study for newly synthesized and biologically tested compounds based on the recently discovered *m*-terphenyl core decorated with cyclic amino acid derivatives as one of the most reported solubilizer tags in anti-PD-L1 SMIs. Compounds were initially tested for their potency in disrupting the PD-1/PD-L1 complex using a standardized homogeneous time-resolved fluorescence (HTRF) assay followed by the cell-based immune checkpoint blockade (ICB) assay. The understanding of the protein–ligand interactions, including the role of the solubilizer tag, was assessed via computational methods and crystal structure analyses.

We started by developing further the scaffold described in ref (18) to investigate whether its biological properties can be improved by solubilizer tag modifications and how it influences their activity in cells. The synthesis of the *m*-terphenyl-based parent inhibitors presented herein is shown in Scheme 1. The initial *m*-terphenyl core was synthesized using Suzuki–Miyaura coupling reactions as described previously.^18^ An *m*-terphenyl precursor was then reacted with thionyl chloride, leading to a reactive benzyl chloride which is suitable for the subsequent nucleophilic substitution with one of the ester-protected cyclic amino acids: proline (**1**), β-proline (**2**), pipecolinic acid (**3**), nipecotic acid (**4**), and isonipecotic acid (**5**). The obtained esters were then directly transformed into final compounds using an aminolysis reaction or were hydrolyzed and coupled with different amines. All of the final structures are reported in Table S1 in the SMILES format.

**Scheme 1:**
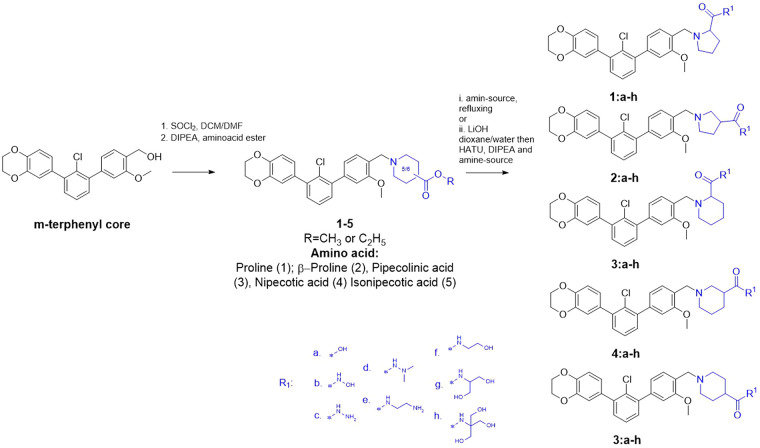
Overview of the Compound Synthesis

The synthesized compounds were tested for their potency in disrupting the PD-1/PD-L1 complex by using the HTRF assay (Table 1). The IC_50_ of reference compound **BMS-1166** in the HTRF assay, with a value of 3.89 ± 0.19 nM, was reported in our previous paper.^18^ The vast majority of the tested compounds effectively disrupted the PD-1/PD-L1 complex in the subnanomolar range, similar to one of the most prominent small-molecule inhibitors of PD-L1, **compound A**.^28^ In our research, we tested the impact of the ring size, solubilizer tag position, and type on the inhibitory activity. Furthermore, we conducted a comparative analysis of the activity exhibited by the tested compounds considering both their calculated and experimentally determined solubilities (Figure 1). The most prominent solubilizing tags among all the tested amino acid fragments are proline (**1a**–**1h**), β-proline (**2a**–**2h**), and isonipecotic acid (**5a**–**5h**), while pipecolinic acid (**3a**–**3h**) and nipecotic acid (**4a**–**4h**) derivatives, which are often used as PD-L1 SMI solubilizers, were generally the worst-performing. This effect correlates with the solubilities of the compounds, with the β-proline series demonstrating optimal activity and solubility. As expected, the acidic forms (series “a”) of the evaluated inhibitors were characterized with enhanced solubility and activity compared to their less acidic counterparts, namely, hydroxamic acids (series “b”), hydrazides (series “c”), and *N*,*N*-dimethylhydrazides (series “d”). Additionally, the introduction of amides as solubilizing tags maintained excellent activity for the tested derivatives, both in ethylenediamine (series “e”) and ethanolamine (series “f”) derivatives. However, an increase in the molecular size of the solubilizer, achieved by incorporating serinol (series “g”) and TRIS (series “h”), resulted in a slight weakening of compound activity. Nevertheless, the comparison of activity among the mentioned amides remains uncertain due to the detection limit of the HTRF method. It is noteworthy, though, that the solubilities of amides are not as favorable as those of their smaller counterparts. In general, the activity of the tested compounds seems to be correlated with the solubility rather than the type and size of the solubilizing tag. Interestingly, some compounds present a strong correlation between experimental and calculated solubilities (the same color of triangles within a square in Figure 1), showing the progress in the solubility determination algorithms for small molecules and therefore justifying their application.

**Table 1:**
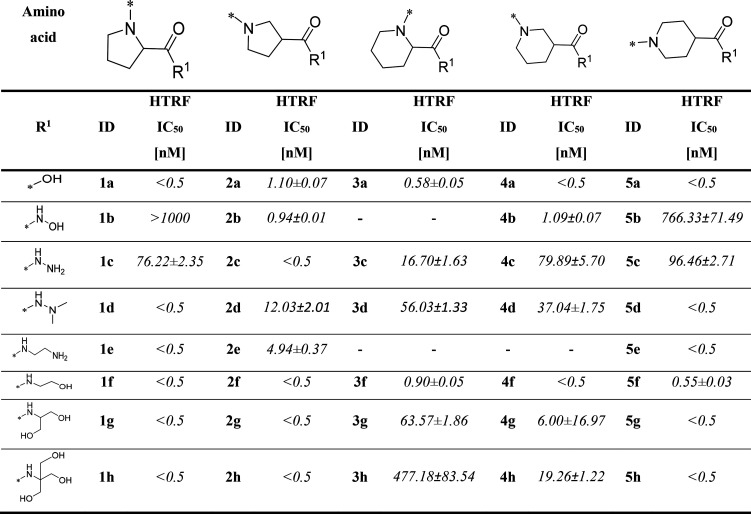
Potencies of Tested Compounds in Disrupting the PD-1/PD-L1 Complex Using HTRF (*n* = 2)

**Figure 1:**
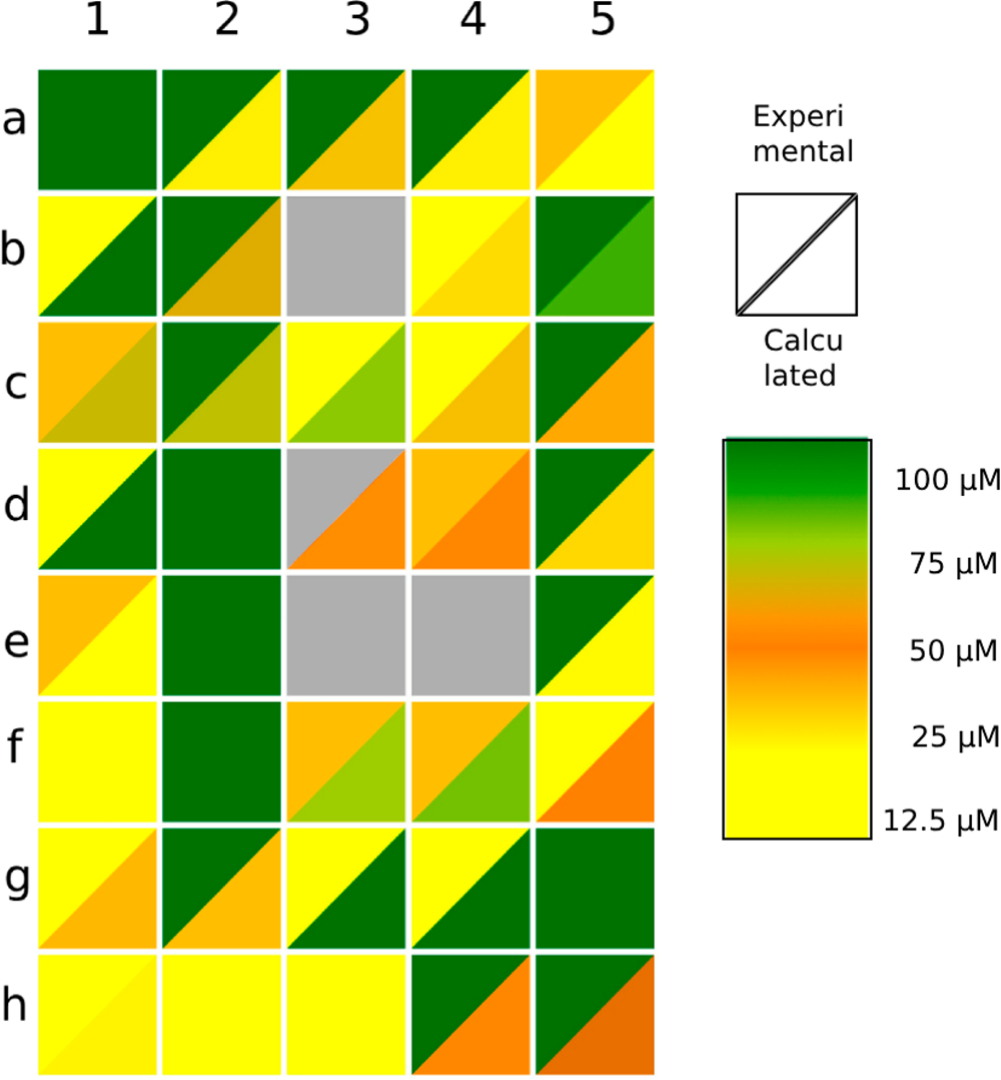
Comparison of the experimental and calculated solubilities of the tested compounds. Solubility levels are visually represented on a color scale, ranging from optimal (green) to weak (yellow) solubility. Each square on the display corresponds to a specific compound, with its position indicated by a combination of a number (column) and a letter (row) in alignment with Table 1. The upper triangle of each square displays the experimental solubility, while the lower part reflects the calculated value. Gray coloring represents unavailable data.

Following the HTRF analysis, the activities of the selected molecules were verified in the well-exploited cell-based PD-1/PD-L1 ICB assay.^16−18,30^ For the analysis, compounds **2a**–**2h** were chosen as a group displaying the most striking activity in the HTRF analysis and favorable solubility profile. All the analyzed compounds increased the activation of effector Jurkat T cells (Jurkat-ECs) in the assay, where the activation thereof is blocked by the PD-1/PD-L1 immune checkpoint (Figure 2A). This bioactivity was observed at a concentration of 1 μM for the β-proline derivatives **2b**, **2e**, **2g**, and **2h** (Figure 2B). For compounds **2d** and **2g**, this activity was retained at the concentration of 6.4 μM, while for compounds **2e** and **2h** the T cell activation dropped down, most probably due to toxic effects on the cells used in the assay. A more detailed view into the activation of Jurkat-ECs revealed the highest dose-dependent activity and lowest toxicity of the compounds **2d**, **2f**, and **2g**, which make these molecules the best candidates for further optimization (Figure S1). The observation proves the PD-L1-blocking activity of the compounds in the cellular context, although it has to be acknowledged that the observed effect is considerably lower than that observed for the control anti-PD-L1 antibody durvalumab (Figure 2B).

**Figure 2:**
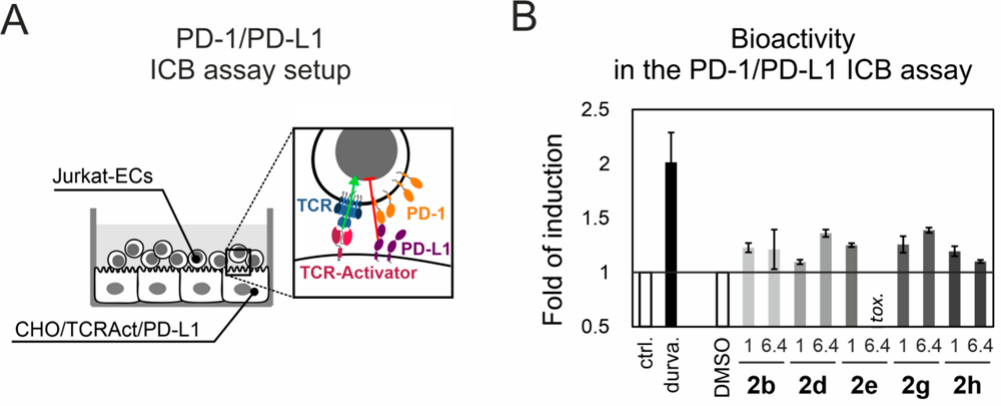
Bioactivities of the molecules in the *in vitro* PD-1/PD-L1 immune checkpoint blockade (ICB) assay. (A) Schematic representation of the assay, in which the effector Jurkat T cells (Jurkat-ECs) were incubated with the stimulator CHO/TCRAct/PD-L1 cells in the presence of the tested molecules. (B) Fold induction of the activation of Jurkat-ECs in the presence of either the indicated compounds (1 μM or 6.4 μM) or therapeutic anti-PD-L1 antibody (1 μg/mL), relative to controls. Untreated cells (ctrl.) served as controls for the durvalumab treatment and DMSO-treated cells as controls for the compound treatments. Data points represent mean ± SD values from duplicates.

Diffraction-quality crystals of the PD-L1/**2f** complex were obtained by using a sitting-drop setup. The final resolution of the obtained cocrystal structure was 2.1 Å (crystallographic parameters are shown in Table S2). The asymmetric unit contains one molecule of inhibitor **2f** and two molecules of PD-L1, which form a homodimer (Figure 3A). This type of dimerization upon the interaction with the inhibitor has been previously observed for biphenyl-based scaffold inhibitors of PD-L1.^32^ The terphenyl moiety of **2f** provides a strong stabilizing π interaction with _A_Tyr56 as well as numerous hydrophobic interactions with, both PD-L1 subunits’ amino acids, including _A_Tyr56, _A_Met115, _B_Met115, _A_Ala121, _B_Ala121, and _B_Tyr123 (Figure 3B). A strong salt bridge between the _B_Asp122 carboxylic group and the protonated amine of the **2f** molecule is also observed. Additionally, a hydrogen bond between _B_Arg125 and the terminal hydroxyl group of **2f** is observed. However, it should be noted that the electron density of this terminal part of the inhibitor is poor, suggesting a high flexibility of the **2f** solubilizer tag (Figure 3B). Therefore, the presented spatial orientation of the solubilizer tag was based on possible protein–ligand interactions.

**Figure 3:**
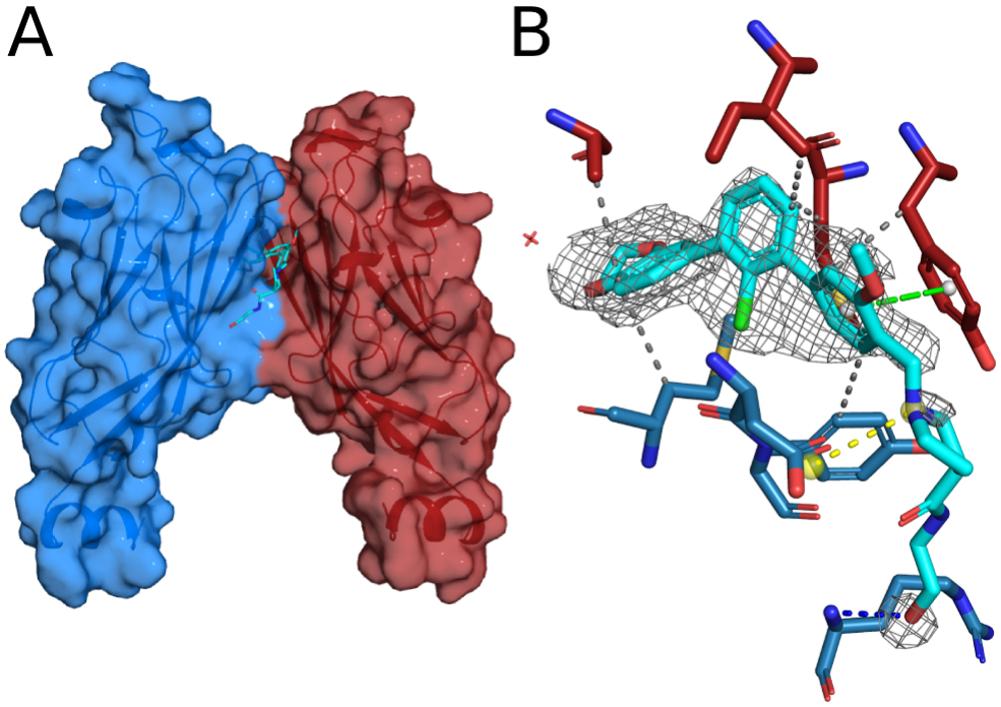
Cocrystal structure of PD-L1 and inhibitor **2f**. (A) Surface/cartoon representation of PD-L1 dimer with the A subunit in red and the B subunit in blue. Inhibitor **2f** is presented in a cyan stick style with atoms color-coded. (B) The **2f** molecule and its protein surroundings, showing interactions with PD-L1 amino acids, superimposed on the 2*F*_o_ – *F*_c_ electron density difference Fourier map leveled at 3σ (gray isomesh). The inhibitor **2f** is colored cyan, _A_PD-L1 amino acids are colored red, and _B_PD-L1 amino acids are colored blue. The hydrogen bond is indicated with a blue dashed line. Hydrophobic interactions are indicated with gray dashed lines. The π-stacking interaction is shown as a green dashed line with gray spheres. The salt bridge is shown as a yellow dashed line with yellow spheres. Waters are indicated as red crosses.

Compound **2a** crystallizes in the centrosymmetric space group *I*2/*a* (Table S3). The asymmetric unit consists of one molecule in the zwitterionic form (Figures 4A and S2–S4). Additionally, there are four water molecules, from which two (namely, O3W and O4W) are located at a special position and represent two alternative molecules’ locations. Water molecule O2W is disordered and refined in two positions with site occupancies of 55% and 45%. All water molecules form a network of hydrogen bonds propagating in a channel along [100]. The presence of the water channels in the proximity of the solubilizing tag confirms the hydrophilic properties of this molecular fragment. The fluctuating water molecules’ positions lead to disorder within the solubilizing tag (β-proline and its carboxylic substituent), with refined site occupancies of 54% and 46%. The two alternative positions are shown in Figure 4B (the less abundant conformation is shown in green).

**Figure 4:**
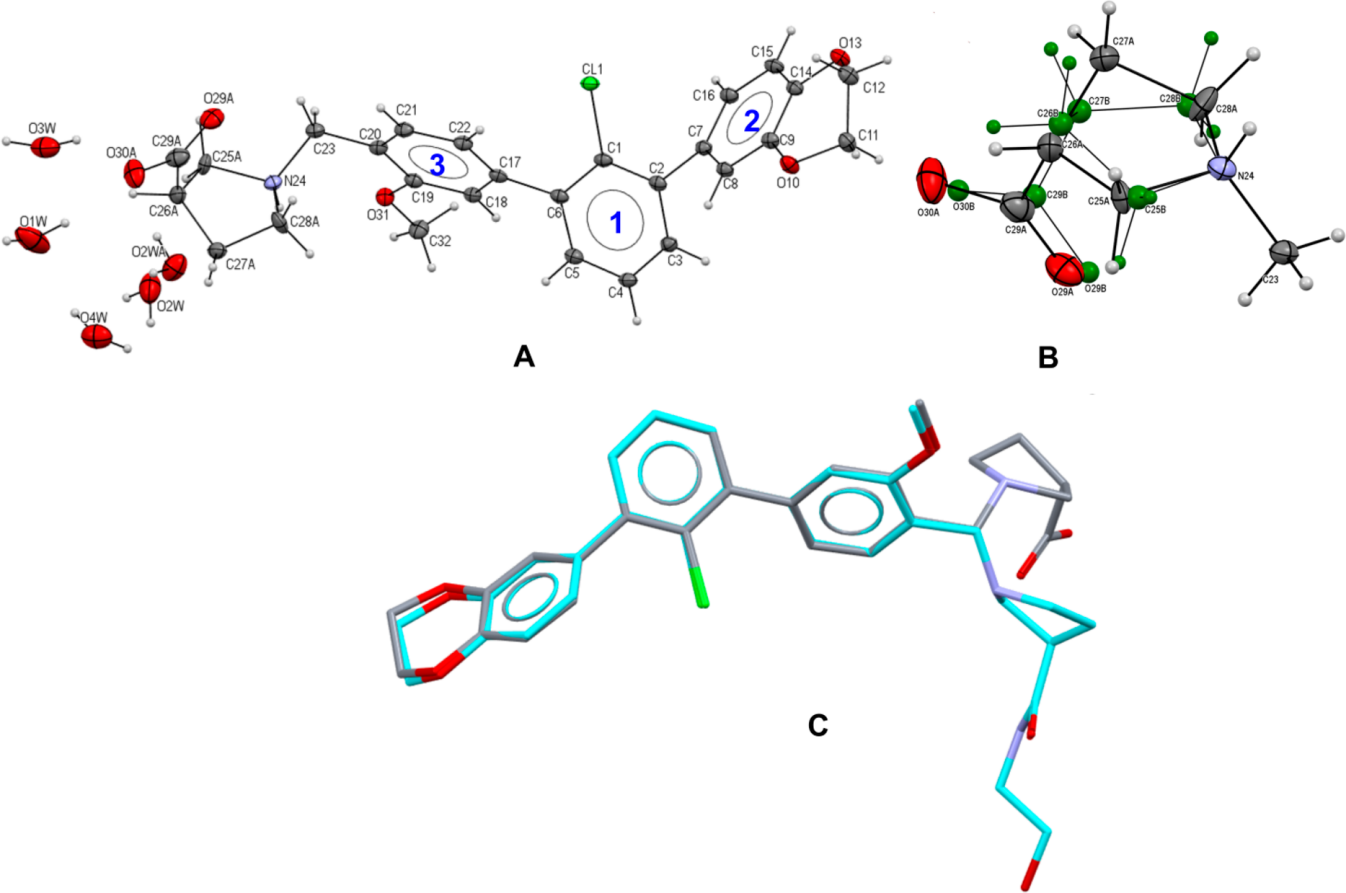
(A) Asymmetric unit of the **2a** crystal structure. Here the more abundant conformation of the organic compound is shown with aromatic rings marked with a numerical tag. (B) Four disordered water molecules are located in the proximity of the solubilizing tag, leading to disorder within β-proline and its carboxylic substituent. The less abundant conformation shown as green small spheres. (C) Superposed molecule **2a** (gray) in the geometry observed in the small-molecule crystal structure and molecule **2f** (cyan) in the conformation observed in the protein–ligand complex crystal, showing good agreement within the main terphenyl core with RMSD ∼ 0.11 Å for aromatic C atoms. Displacement ellipsoids of non-hydrogen atoms are drawn at the 30% probability level. H atoms are presented as small spheres with an arbitrary radius. The superposed molecules are shown in stick representation, with H atoms removed for figure clarity.

Apart from hydrogen bonds involving water molecules, the strongest observed intermolecular interaction is a charge-assisted hydrogen bond (salt bridge) formed between the protonated amine of the β-proline and the carboxylate anion of the neighboring molecule. This interaction propagates parallel to the water channels. The corresponding salt-bridge interaction is also observed in the protein–ligand crystal structure presented here, where the protonated amine of **2f** can interact with the anionic form of _B_Asp122. In the crystal of **2a**, several C–H···O interactions are observed (Table S4), which additionally stabilize the crystal structure.

The molecular conformation of the main aromatic *m*-terphenyl core is well conserved for the small-molecule and protein–ligand crystal structures. The superposition of the *m*-terphenyl fragment for compound **2a** in its crystal form (Figure 4C, the molecule with carbon atoms in gray) on the one observed for compound **2f** in the binding cavity of the PD-L1 dimer (Figure 4C, the molecule with cyan carbon atoms) shows that these fragments are almost identical, with a root-mean-square deviation (RMSD) for aromatic ring carbon atoms of ∼0.11 Å. For structure **2a**, the torsion angles C1–C2–C7–C16 (TOR1) and C1–C6–C17–C22 (TOR2) are 56.25° and 46,64°, respectively. The mutual aromatic fragments’ orientation may be defined also by angles between planes of phenyl rings 1–3 (marked with blue numbers in Figure 4A) with angles 1/2 (ANG1), 1/3 (ANG2), and 2/3 (ANG3) being 53.85°, 46.06°, and 87.44°, respectively. Such a spatial orientation of π-electron-rich fragments may be the main characteristic responsible for binding to the PD-L1 dimer, as it matches the corresponding *m*-terphenyl angles in the cocrystal structure. Therefore, we postulate that a correct preorientation of the core *m*-terphenyl scaffold in our inhibitors is primarily responsible for its strength in dissociating the PD-1/PD-L1 complex, as it avoids a thermodynamic penalty because no “torsion adjustments” are required for the inhibitor. Interestingly, such mutual aromatic rings’ arrangement is not very strictly defined and conserved for different *m*-terphenyl-containing structures and strongly depends on substituents. The Cambridge Structural Database (CSD) (ver. 5.43, November 2021)^33^ search revealed a wide range of values for all the analyzed geometrical parameters (the histograms showing the statistical distribution of TOR1–2 and ANG1–3 are presented in Figures S5–S9), with maximum counts for TOR1–2 in the range ±(80–100)°, ANG1–2 in the range 75–90°, and ANG3 in the range 45–60°. From the statistical point of view, the *m*-terphenyl derivatives presented here adopt a peculiar geometry not strongly represented in CSD results, which can be defined as a strong pharmacophore feature for the PD-1/PD-L1 inhibitors, perhaps justifying why such scaffolds were not reported previously.

The solubilizing tags of **2a** and **2f** in the crystal structures presented here are oriented differently. When a ligand is bound to PD-L1, the geometry of this molecular fragment is the most sensitive to the environment, as it is exposed to the solvent and therefore may display a high disorder level, which can be confirmed by the low coverage of the 2*F*_o_ – *F*_c_ electron density map (at contour level 3σ) from Figure 4B. The mobility of this fragment may suggest the formation of interactions with both protein and solvent in a competitive and even interchanging manner.

The docking procedure was performed based on the PD-L1/**2f** cocrystal structure. The protein structure was thoughtfully screened against all the available PD-L1/SMI complexes deposited in the Protein Data Bank^34^ to search for structural differences that appeared to be negligible. All compounds presented in this paper were docked onto the homodimer-formed binding pocket, along with **compound A**,^28^**BMS-1166**,^25,26^ and the *m*-terphenyl analog from the 7NLD crystal structure^18^ used as reference ligands. The summarized graphical representation of the obtained results is shown in Figure 5A (numerical results with ChemPLP scoring function values are included in the Supporting Information).

**Figure 5:**
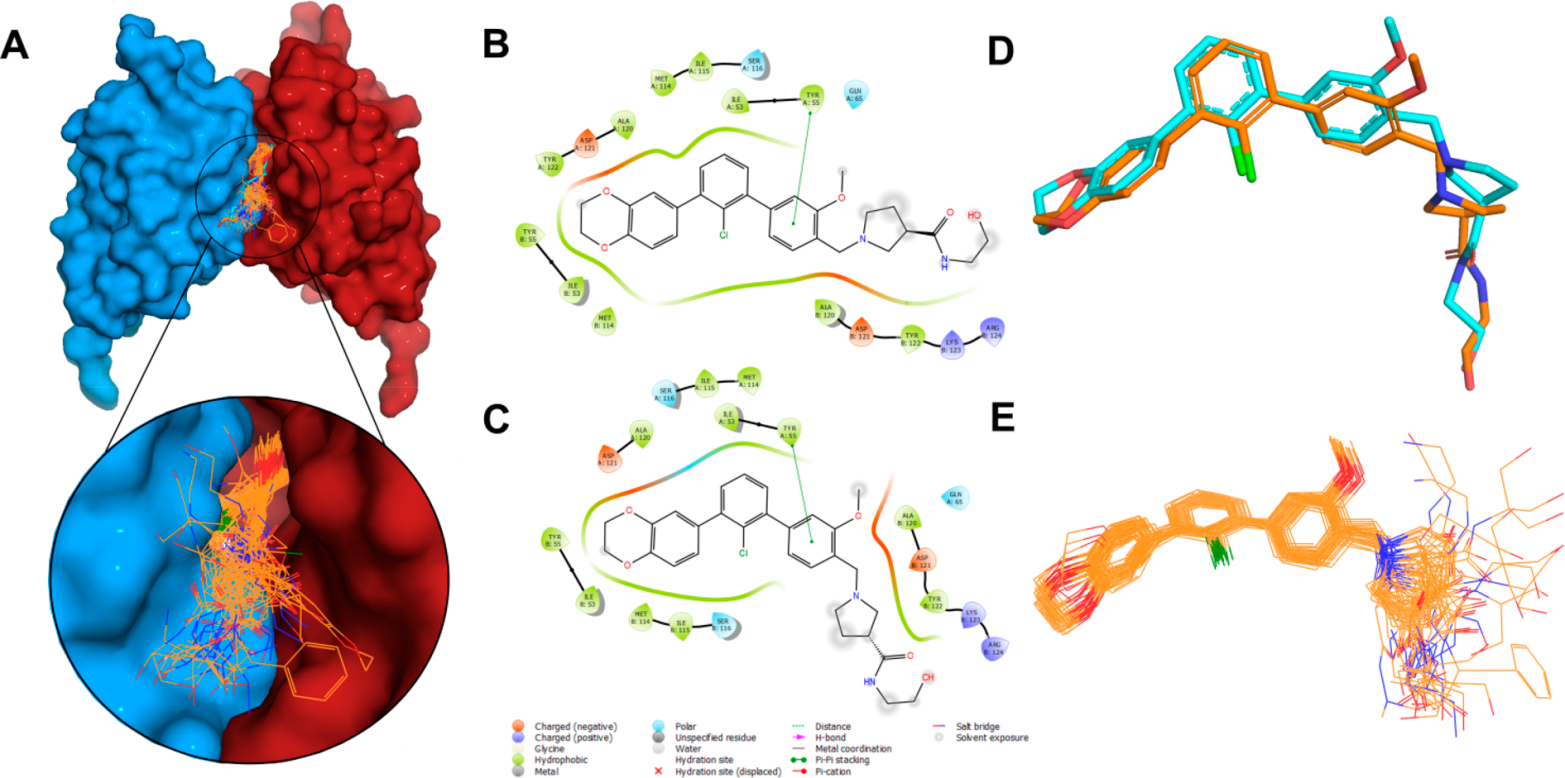
(A) Summarized representation of docking results for all the described *m*-terphenyl derivatives, including the reference PD-L1 ligands **compound A**,^28^**BMS-1166**,^25,26^ and the *m*-terphenyl analog from the 7NLD crystal structure.^18^ (B, C) 2D ligand interaction plots (Schrödinger release 2020-3: Maestro, Schrödinger, LLC, New York) for the **2f** conformer observed in the protein–ligand crystal structure and the **2f** redocking result, respectively. (D) Superposition of native **2f** conformer and its redocking result. (E) Superposition of all docked *m*-terphenyl derivatives (the best-scored poses).

The docking results revealed that the positive charge generated on the protonated amine corresponds to a higher scoring function result compared with the neutral form of the ligand. This is related to the N^+^–H···_B_Asp122 salt bridge formation that strongly stabilizes the protein–ligand complex.^35^

The redocking procedure resulted in well-reconstructed **2f** conformation and protein–ligand interactions (Figure 5B,C). The calculated RMSD based on all non-hydrogen atoms in **2f** is 0.84 Å. The discrepancy between the structure and predicted pose was observed for the 2,3-dihydro-1,4-benzodioxine moiety, which is related to the translational shift of the docked compound, lacking the native ligand’s position by ca. 0.5 Å but still preserving all key interactions (Figure 5D). The deeper penetration of the ligand is a consequence of removal of a water molecule from the binding site prior to the docking procedure. The major divergence is observed for the solubilizer tag region, further confirming that this fragment is in fact highly mobile in the protein/ligand complex (Figure 5D).

The comparison of all of the best-scored poses for each of the investigated ligands revealed a highly conserved location and orientation of the *m*-terphenyl fragment with a strong diversity in the solubilizer tag geometry (Figure 5E). This result hints that the role of this highly mobile terminal fragment is not trivial. The externally exposed terminal tail of the ligand may be less involved in PD-L1/SMI complex stabilization but rather competitively forms interactions with polar amino acids in the cavity’s entrance and environmental water molecules. This dynamic exchange may prevent water molecules from entering the hydrophobic pocket and additionally increase the entropy of the system. The analysis of the available PD-L1/SMI complexes shows that for the majority of the investigated crystal structures, the electron density of this terminal molecular fragment is poorly defined, and therefore, the presented protein/ligand stabilizing interactions are doubtful (e.g., PDB IDs 5J89, 5N2D, 5N2F, 6NM8, 7BEA, 7DY7, and 7NLD). The well-defined density is observed when the solubilizer tag is arborescent (e.g., PDB IDs 5NIU, 6R3K, and 6RPG). By the more stable conformation of this bifurcated tail, the deeper region of the binding cavity is better shielded from water molecules. It is worth noting that for the mentioned crystal structures, the observed protein/ligand contacts are either water-mediated or mostly weak interactions.

The solubility study and log *S* prediction results show that all of the studied compounds in their neutral form are only moderately soluble (Figure 1). However, the majority of the presented compounds are in their cationic forms due to the protonation of the amine group within the solubilizer tag at physiological pH, which affects the resulting compounds’ final solubility. Additionally, the predicted log *P* seems to be optimal only for **compound A**, whereas for most of the tested compounds, this important pharmacological parameter is in the range of 4–5, implying high lipophilicity of the *m*-terphenyl derivatives and hindering their accessibility in a water environment.

The weak correlation between the docking results and the biological tests can be related to the still not fully understood mechanism of action of PD-L1 ligands, which may be based on the synergistic effect of cell-surface PD-L1 dimerization as well as influencing some intracellular processes.^28^ Thus, all of the considered physicochemical parameters, such as ionization of the compound, low solubility, and high lipophilicity, can be treated as limiting factors which cooperatively influence the biological activity of PD-L1 ligands.

The success of cancer therapy by inhibition of negative immune regulation was awarded the 2018 Nobel Prize in Physiology or Medicine jointly to James P. Allison and Tasuku Honjo. It fueled the development of SMIs disrupting the PD-1/PD-L1 immune checkpoint. Despite the great interest resulting in numerous patents and publications on the PD-L1-targeted SMIs, the understanding of the mode of action of SMIs on PD-L1 at a molecular level is still not well-established. Classically, anti-PD-L1 SMIs’ scaffolds are divided into the biaryl core responsible for the PD-L1 dimerization, followed by the aryl moiety with an ether-linked group to increase the number of “binding anchors”, and terminated by the solubilizer tag accounting primarily for the enhancement of the compound solubility index (nowadays, many deviations from this classical outline are reported, such as mirrored compounds, etc.).^22^ The question also arises whether the solubilizing fragment influences ligand/protein binding and may be rationally designed to increase the potency of SMIs to stabilize the ligand-induced PD-L1 homodimerization. This led us to the formulation of guidelines for anti-PD-L1 SMIs. Continuing the work on the *m*-chloroterphenyl scaffold, we found that its characteristic preorientation of the aromatic rings in the inhibitor’s scaffold to engage PD-L1’s _A_Tyr56, _A_Met115, and _B_Tyr123 in strong hydrophobic/π interactions favors subnanomolar inhibitory constants. Therefore, the correct terphenyl substitution (with, *e.g.*, a halogen or a methyl at the *ortho* position) leading to steric hindrance and lowering the resonance effect as it was shown in the ligand’s crystal structure and the following CSD search presented in this article is a valid and straightforward strategy for the development of strong conformational scaffolds and pharmacophore models.

Solubilizer tags are often considered to increase the solubility of anti-PD-L1 compounds, which are usually quite hydrophobic. However, based on the performed *in silico* modeling routine and the obtained experimental results, we did not find a correlation. Clearly, poor inhibitor solubility can lead to its aggregation in the polar environment (such as buffers) and lower its effective concentration. Moreover, the connection between anti-PD-L1 SMIs and their lipophilicity is a convoluted process that we do not understand fully yet. Also, the second most postulated argument that solubilizers provide additional stabilizing contacts with PD-L1, such as the hydrogen bond between _B_Arg125 and the terminal hydroxyl group of compound **2f** reported here, seems not very obvious, as the poor electron density around this terminal part of the inhibitor suggests a high degree of flexibility of this fragment. A more likely explanation of the “solubilizer tag” role is that due to its high degree of conformational changes in the PD-L1 dimer-formed binding cavity, it prevents water molecules from penetrating the hydrophobic core of the ligand/protein complex. This is illustrated in our docking routine, where resulting poses with similar predicted binding scores represent various solubilizer tag orientations. In our work, we decorated the *m*-chloroterphenyl scaffold with polar amino acid derivatives such as proline, pipecolinic acid, or isonipecotic acid conjugated with various terminal groups, including hydroxyl, amides, acyl hydrazides, and ethanolamine groups. Nearly half of the reported compounds were more potent in the disruption of PD-1/PD-L1 complex than the well-known compound **BMS-1166** and showed similar results as one of the most active inhibitors to date, **compound A**. Especially, the β-proline series (**2a**–**2h**) proved to be potent, as all terminal fragments of this group gave the best results with subnanomolar IC_50_ values.

The ionization/protonation state of anti-PD-L1 inhibitors is often neglected and/or not considered in the *in silico* design of SMIs. Nevertheless, this parameter is crucial, as it can affect the binding energy and complex stabilization by highly favorable salt bridge formation, which leads to an enhancement of the biological activity toward PD-L1 for both macrocyclic peptides and SMIs.^35^ Application of this information in the *in silico* approach can increase the predictive power of the molecular-docking-based method for the studied protein–ligand system. Additionally, the potential ionization of the putative drug molecule may be a critical factor influencing bioavailability and altering the properties of cell penetration. The latter would be especially important in the case of the dual surface–internal/cytoplasmic mode of action of anti-PD-L1 small inhibitors.

Guidelines formulated here for PD-L1 SMIs shed more light on the often-neglected subject of the importance of the solubilization tag. Through the extensive biological, biochemical, and structural analysis exemplified by the *m*-chloroterphenyl scaffold, we aimed to structure the current knowledge about the importance and complex function of the solubilizing tag in the design of PD-L1 SMIs.

## Data Availability

Data will be made available on request.

## Supporting Information Available

CSD search; Tables S1–S4; Figures S1–S9; experimental procedures; solubility measurements; results of the HTRF assay; PD-1/PD-L1 ICB assay; data collection and refinement statistics (molecular replacement) for the PD-L1 cocrystal structure; crystal structure determination for compound **2a**; molecular modeling; spectral data and purity of target compounds (PDF)
Molecular docking results (PDF)

## Accession Codes

Crystallographic data for compound **2a** have been deposited with the Cambridge Crystallographic Data Centre (CCDC) as supplementary publication no. CCDC 2231594. Copies of the data can be obtained free of charge on application to CCDC (email: deposit@ccdc.cam.ac.uk). The structure factors and final models of PD-L1 complexes with inhibitor **2f** were deposited into the Protein Data Bank with the accession number 8R6Q.

## Abbreviations

HTRF: homogeneous time-resolved fluorescence
ICB: immune checkpoint blockade
SMI: small-molecule inhibitor
PD-L1: programmed cell death protein ligand 1
PD-1: programmed cell death protein 1
PDB: Protein Data Bank

## References

[ref1] Keir M. E.; Butte M. J.; Freeman G. J.; Sharpe A. H. PD-1 and Its Ligands in Tolerance and Immunity. Annu. Rev. Immunol. 2008, 26, 677–704. 10.1146/annurev.immunol.26.021607.090331. 18173375 PMC10637733

[ref2] Han Y.; Liu D.; Li L. PD-1/PD-L1 Pathway: Current Researches in Cancer. Am. J. Cancer Res. 2020, 10 (3), 727–742. 32266087 PMC7136921

[ref3] Chen L.; Han X. Anti-PD-1/PD-L1 Therapy of Human Cancer: Past, Present, and Future. J. Clin. Invest. 2015, 125 (9), 3384–3391. 10.1172/JCI80011. 26325035 PMC4588282

[ref4] Sharpe A. H.; Wherry E. J.; Ahmed R.; Freeman G. J. The Function of Programmed Cell Death 1 and Its Ligands in Regulating Autoimmunity and Infection. Nat. Immunol. 2007, 8 (3), 239–245. 10.1038/ni1443. 17304234

[ref5] Alsaab H. O.; Sau S.; Alzhrani R.; Tatiparti K.; Bhise K.; Kashaw S. K.; Iyer A. K. PD-1 and PD-L1 Checkpoint Signaling Inhibition for Cancer Immunotherapy: Mechanism, Combinations, and Clinical Outcome. Front. Pharmacol. 2017, 8 (AUG), 1–15. 10.3389/fphar.2017.00561. 28878676 PMC5572324

[ref6] Ribas A.; Wolchok J. D. Cancer Immunotherapy Using Checkpoint Blockade. Science 2018, 359 (6382), 1350–1355. 10.1126/science.aar4060. 29567705 PMC7391259

[ref7] Sharma P.; Allison J. P. The Future of Immune Checkpoint Therapy. Science 2015, 348 (6230), 56–61. 10.1126/science.aaa8172. 25838373

[ref8] Sharma P.; Allison J. P. Dissecting the Mechanisms of Immune Checkpoint Therapy. Nat. Rev. Immunol. 2020, 20 (2), 75–76. 10.1038/s41577-020-0275-8. 31925406

[ref9] Sunshine J.; Taube J. M. PD-1/PD-L1 Inhibitors. Curr. Opin. Pharmacol. 2015, 23, 32–38. 10.1016/j.coph.2015.05.011. 26047524 PMC4516625

[ref10] Lin X.; Lu X.; Luo G.; Xiang H. Progress in PD-1/PD-L1 Pathway Inhibitors: From Biomacromolecules to Small Molecules. Eur. J. Med. Chem. 2020, 186, 111876 10.1016/j.ejmech.2019.111876. 31761384

[ref11] Sukari A.; Nagasaka M.; Al-Hadidi A.; Lum L. G. Cancer Immunology and Immunotherapy. Anticancer Res. 2016, 36 (11), 5593–5606. 10.21873/anticanres.11144. 27793882

[ref12] Twomey J. D.; Zhang B. Cancer Immunotherapy Update: FDA-Approved Checkpoint Inhibitors and Companion Diagnostics. AAPS J. 2021, 23 (2), 39 10.1208/s12248-021-00574-0. 33677681 PMC7937597

[ref13] Johansen A.; Christensen S. J.; Scheie D.; Højgaard J. L. S.; Kondziella D. Neuromuscular Adverse Events Associated with Anti-PD-1 Monoclonal Antibodies: Systematic Review. Neurology 2019, 92 (14), 663–674. 10.1212/WNL.0000000000007235. 30850443

[ref14] Hutchinson J. A.; Kronenberg K.; Riquelme P.; Wenzel J. J.; Glehr G.; Schilling H.-L.; Zeman F.; Evert K.; Schmiedel M.; Mickler M.; Drexler K.; Bitterer F.; Cordero L.; Beyer L.; Bach C.; Koestler J.; Burkhardt R.; Schlitt H. J.; Hellwig D.; Werner J. M.; Spang R.; Schmidt B.; Geissler E. K.; Haferkamp S. Virus-Specific Memory T Cell Responses Unmasked by Immune Checkpoint Blockade Cause Hepatitis. Nat. Commun. 2021, 12 (1), 1439 10.1038/s41467-021-21572-y. 33664251 PMC7933278

[ref15] Butera R.; Ważyńska M.; Magiera-Mularz K.; Plewka J.; Musielak B.; Surmiak E.; Sala D.; Kitel R.; De Bruyn M.; Nijman H. W.; Elsinga P. H.; Holak T. A.; Dömling A. Design, Synthesis, and Biological Evaluation of Imidazopyridines as PD-1/PD-L1 Antagonists. ACS Med. Chem. Lett. 2021, 12 (5), 768–773. 10.1021/acsmedchemlett.1c00033. 34055224 PMC8155249

[ref16] Konieczny M.; Musielak B.; Kocik J.; Skalniak L.; Sala D.; Czub M.; Magiera-Mularz K.; Rodriguez I.; Myrcha M.; Stec M.; Siedlar M.; Holak T. A.; Plewka J. Di-Bromo-Based Small-Molecule Inhibitors of the PD-1/PD-L1 Immune Checkpoint. J. Med. Chem. 2020, 63 (19), 11271–11285. 10.1021/acs.jmedchem.0c01260. 32936638 PMC7584369

[ref17] Magiera-Mularz K.; Skalniak L.; Zak K. M.; Musielak B.; Rudzinska-Szostak E.; Berlicki Ł.; Kocik J.; Grudnik P.; Sala D.; Zarganes-Tzitzikas T.; Shaabani S.; Dömling A.; Dubin G.; Holak T. A. Bioactive Macrocyclic Inhibitors of the PD-1/PD-L1 Immune Checkpoint. Angew. Chem., Int. Ed. 2017, 56 (44), 13732–13735. 10.1002/anie.201707707. PMC6400216 28881104

[ref18] Muszak D.; Surmiak E.; Plewka J.; Magiera-Mularz K.; Kocik-Krol J.; Musielak B.; Sala D.; Kitel R.; Stec M.; Weglarczyk K.; Siedlar M.; Dömling A.; Skalniak L.; Holak T. A. Terphenyl-Based Small-Molecule Inhibitors of Programmed Cell Death-1/Programmed Death-Ligand 1 Protein-Protein Interaction. J. Med. Chem. 2021, 64 (15), 11614–11636. 10.1021/acs.jmedchem.1c00957. 34313116 PMC8365601

[ref19] Rodriguez I.; Kocik-Krol J.; Skalniak L.; Musielak B.; Wisniewska A.; Ciesiołkiewicz A.; Berlicki Ł.; Plewka J.; Grudnik P.; Stec M.; Siedlar M.; Holak T. A.; Magiera-Mularz K. Structural and Biological Characterization of PAC65, a Macrocyclic Peptide That Blocks PD-L1 with Equivalent Potency to the FDA-Approved Antibodies. Mol. Cancer 2023, 22 (1), 150 10.1186/s12943-023-01853-4. 37679783 PMC10483858

[ref20] Shaabani S.; Huizinga H. P. S.; Butera R.; Kouchi A.; Guzik K.; Magiera-Mularz K.; Holak T. A.; Dömling A. A Patent Review on PD-1/PD-L1 Antagonists: Small Molecules, Peptides, and Macrocycles (2015–2018). Expert Opin. Ther. Pat. 2018, 28 (9), 665–678. 10.1080/13543776.2018.1512706. 30107136 PMC6323140

[ref21] Sun G.; Rong D.; Li Z.; Sun G.; Wu F.; Li X.; Cao H.; Cheng Y.; Tang W.; Sun Y. Role of Small Molecule Targeted Compounds in Cancer: Progress, Opportunities, and Challenges. Front. Cell. Dev. Biol. 2021, 9, 694363 10.3389/fcell.2021.694363. 34568317 PMC8455877

[ref22] Guzik K.; Tomala M.; Muszak D.; Konieczny M.; Hec A.; Błaszkiewicz U.; Pustuła M.; Butera R.; Dömling A.; Holak T. A. Development of the Inhibitors That Target the PD-1/PD-L1 Interaction—A Brief Look at Progress on Small Molecules, Peptides and Macrocycles. Molecules 2019, 24 (11), 2071 10.3390/molecules24112071. 31151293 PMC6600339

[ref23] Lin X.; Lu X.; Luo G.; Xiang H. Progress in PD-1/PD-L1 Pathway Inhibitors: From Biomacromolecules to Small Molecules. Eur. J. Med. Chem. 2020, 186, 111876 10.1016/j.ejmech.2019.111876. 31761384

[ref24] Yang J.; Hu L. Immunomodulators Targeting the PD-1/PD-L1 Protein-Protein Interaction: From Antibodies to Small Molecules. Med. Res. Rev. 2019, 39 (1), 265–301. 10.1002/med.21530. 30215856

[ref25] Chupak L. S.; Zheng X. (Bristol-Myers Squibb Company). Compounds Useful as Immunomodulators. WO 2015/034820 A1, 2015.

[ref26] Chupak L.; Ding M.; Martin S.; Zheng X.; Hewawasam P.; Connoly T.; Xu N.; Yeung K.; Zhu J.; Langley D.; Tenney D.; Scola P. (Bristol-Myers Squibb Company). Compounds Useful as Immunomodulators. WO 2015/160641 A2, 2015.

[ref27] Wu Q.; Jiang L.; Li S. C.; He Q.-J.; Yang B.; Cao J. Small molecule inhibitors targeting the PD-1/PD-L1 signaling pathway. Acta Pharmacol. Sin. 2021, 42, 1–9. 10.1038/s41401-020-0366-x. 32152439 PMC7921448

[ref28] Park J. J.; Thi E. P.; Carpio V. H.; Bi Y.; Cole A. G.; Dorsey B. D.; Fan K.; Harasym T.; Iott C. L.; Kadhim S.; Kim J. H.; Lee A. C. H.; Nguyen D.; Paratala B. S.; Qiu R.; White A.; Lakshminarasimhan D.; Leo C.; Suto R. K.; Rijnbrand R.; Tang S.; Sofia M. J.; Moore C. B. Checkpoint Inhibition through Small Molecule-Induced Internalization of Programmed Death-Ligand 1. Nat. Commun. 2021, 12 (1), 1222 10.1038/s41467-021-21410-1. 33619272 PMC7900207

[ref29] Surmiak E.; Magiera-Mularz K.; Musielak B.; Muszak D.; Kocik-Krol J.; Kitel R.; Plewka J.; Holak T. A.; Skalniak L. PD-L1 Inhibitors: Different Classes, Activities, and Mechanisms of Action. Int. J. Mol. Sci. 2021, 22 (21), 11797 10.3390/ijms222111797. 34769226 PMC8583776

[ref30] Cheng Z.-J. J.; Karassina N.; Grailer J.; Hartnett J.; Fan F.; Cong M. Abstract 5440: Novel PD-1 Blockade Bioassay to Assess Therapeutic Antibodies in PD-1 and PD-L1 Immunotherapy Programs. Cancer Res. 2015, 75 (15_Suppl.), 5440 10.1158/1538-7445.AM2015-5440.

[ref32] Guzik K.; Zak K. M.; Grudnik P.; Magiera K.; Musielak B.; Törner R.; Skalniak L.; Dömling A.; Dubin G.; Holak T. A. Small-Molecule Inhibitors of the Programmed Cell Death-1/Programmed Death-Ligand 1 (PD-1/PD-L1) Interaction via Transiently Induced Protein States and Dimerization of PD-L1. J. Med. Chem. 2017, 60 (13), 5857–5867. 10.1021/acs.jmedchem.7b00293. 28613862

[ref33] Groom C. R.; Bruno I. J.; Lightfoot M. P.; Ward S. C. The Cambridge Structural Database. Acta Crystallogr., Sect. B 2016, 72 (2), 171–179. 10.1107/S2052520616003954. PMC4822653 27048719

[ref34] Berman H. M.; Westbrook J.; Feng Z.; Gilliland G.; Bhat T. N.; Weissig H.; Shindyalov I. N.; Bourne P. E. The Protein Data Bank. Nucleic Acids Res. 2000, 28 (1), 235–242. 10.1093/nar/28.1.235. 10592235 PMC102472

[ref35] Riccio A.; Coletti A.; Dolciami D.; Mammoli A.; Cerra B.; Moretti S.; Gioiello A.; Ferlin S.; Puxeddu E.; Macchiarulo A. The Stone Guest: How Does PH Affect Binding Properties of PD-1/PD-L1 Inhibitors?. ChemMedChem 2021, 16 (3), 568–577. 10.1002/cmdc.202000760. 33085193

