# Supplementary material for: Solubilizer Tag Effect on PD-L1/Inhibitor Binding Properties for m-Terphenyl Derivatives
Source: ACS Med Chem Lett. 2023 Dec 14;15(1):36–44. doi: 10.1021/acsmedchemlett.3c00306 (PMC10788941; doi:10.1021/acsmedchemlett.3c00306)
Supplement: Supplementary file 1 — ml3c00306_si_001.pdf [file ml3c00306_si_001.pdf]

Solubilizer tag effect on PD-L1/inhibitor binding properties for m-terphenyl derivatives.

Ewa Surmiak[a*], Julia Ząber[a,b], Jacek Plewka [a], Grzegorz Wojtanowicz [a], Justyna Kocik-Król [a,b], Oskar Kruc [a,b], Damian Muszak [a], Ismael Rodríguez[a,b], Bogdan Musielak [a], Monica Viviano [c], Sabrina Castellano [c], Lukasz Skalniak [a], Katarzyna Magiera-Mularz [a], Tad A. Holak [a], Justyna Kalinowska-Tłuścik[a*]

[a] Jagiellonian University, Faculty of Chemistry, Gronostajowa St 2, 30-387 Cracow, Poland

[b] Jagiellonian University, Doctoral School of Exact and Natural Sciences, Prof. St. Łojasiewicz St 11, 30-348, Cracow, Poland

[c] University of Salerno, Departent of Pharmacy, Via Giovanni Paolo II 132, Fisciano, Italy

* corresponding authors: ewa.surmiak@uj.edu.pl, justyna.kalinowska-tluscik@uj.edu.pl

SUPPORTING INFORMATION

1. CAMBRIDGE STRUCTURAL DATABASE (CSD) SEARCH 3
2. SUPPORTING TABLES 4
Table S1. Tested compounds SMILES list 4
Table S2. Diffraction data collection and refinement statistics for PD-L1 complex with 2f. 5
Table S3. Crystal data and final refinement results for structure 2a 6
Table S4. Geometrical parameters of hydrogen bonds observed in the crystal structure of 2a [Å and °]. 7
3. SUPPORTING FIGURES 8
Figure S1. The dose-dependent bioactivity of the molecules in the in vitro PD-1/PD-L1 Immune Checkpoint Blockade (ICB) assay. 8
Figure S2. The asymmetric unit of 2a crystal structure with O2W, O3W, and O4W water molecules shown in the two alternative positions 9
Figure S3. Packing scheme in the crystal in the structure of 2a. View along [100]. 10
Figure S4. Packing scheme in the crystal in the structure of 2a. View along [100]. 11
Figure S5. Distribution of torsion angle corresponding to C1-C2-C7-C16 (TOR1) in structures deposited in CSD, which contain *meta*-terphenyl moiety 12
Figure S6. Distribution of torsion angle corresponding to C1-C6-C17-C22 (TOR2) in structures deposited in CSD, which contain *meta*-terphenyl moiety 13
Figure S7. Distribution of mutual angle between mean planes defined by carbon atoms of aromatic rings 1 and 2 (ANG1) in structures deposited in CSD, which contain *meta*-terphenyl moiety 14
Figure S8. Distribution of mutual angle between mean planes defined by carbon atoms of aromatic rings 1 and 3 (ANG2) in structures deposited in CSD, which contain *meta*-terphenyl moiety 15
Figure S9. Distribution of mutual angle between mean planes defined by carbon atoms of aromatic rings 2 and 3 (ANG3) in structures deposited in CSD, which contain *meta*-terphenyl moiety 16
4. EXPERIMENTAL DETAILS 17
4.1. Compounds synthesis 17
General protocol of esters 1-5 synthesis 17
General protocol of hydrolysis (1a-5a) 20
General procedure for aminolysis 23
General procedure for HATU coupling 32
4.2. Solubility measurements 42
4.3. Homogenous Time-Resolved Fluorescence 42
4.4. PD-1/PD-L1 immune checkpoint blockade (ICB) assay 42
4.5. Protein expression and crystallization 43
4.6. Crystal structure determination for compound 2a 43
4.7. Molecular modeling 44
5. SUPPLEMENTARY REFERENCES 46
6. COPIES OF THE NMR OF THE FINAL COMPOUNDS 48
7. COPIES OF THE LCMS OF THE FINAL COMPOUNDS 85

1. CAMBRIDGE STRUCTURAL DATABASE (CSD) SEARCH

The conformational preferences amongst compounds containing meta-terphenyl core were statistically studied by searching CSD Version 5.43 (November 2021)[1] with the ConQuest 2021.3.0 program.[2] The searched molecular fragment is shown below.

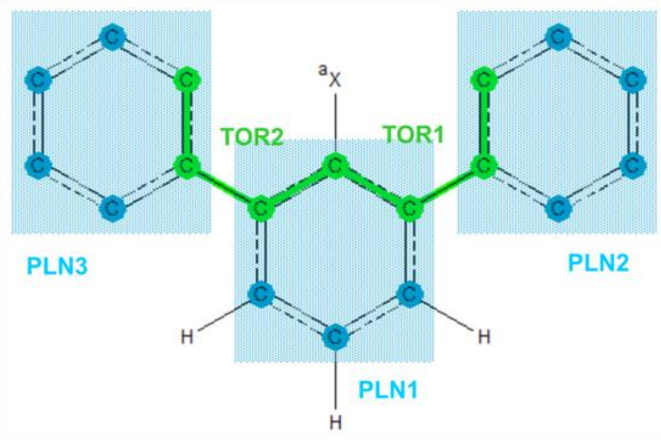

The defined geometrical parameters were angles between planes (PLN1 and PLN2 (ANG1), PLN1 and PLN3 (ANG2), PLN2 and PLN3 (ANG3)), and torsion angles TOR1 and TOR2. [a]X is an acyclic substituent. The search was performed only for single crystal structures of organic compounds, with 3D coordinates available. The search resulted in 683 structures. For 85 results the meta-terphenyl moiety was a fragment of a large cyclic compound. In such rigidified structures the numerical values of the studied geometrical parameters could be influenced by the internal ring stains. For this reason, only 598 structures were selected for the geometrical study. Histograms presenting the range of selected parameter values and their frequencies are shown in **Figures S4-S8**.

2. SUPPORTING TABLES

Table S1. Tested compounds SMILES list

No	SMILES
**1a**	C(O)(C1N(Cc2c(OC)cc(c3c(Cl)c(c5cc4c(OCCO4)cc5)ccc3)cc2)CCC1)O
**1b**	N1(Cc2c(OC)cc(c3c(Cl)c(c5cc4c(OCCO4)cc5)ccc3)cc2)C(C(NO)=O)CCC1
**1c**	N1(Cc2c(OC)cc(c3c(Cl)c(c5cc4c(OCCO4)cc5)ccc3)cc2)C(C(NN)=O)CCC1
**1d**	N1(Cc2c(OC)cc(c3c(Cl)c(c5cc4c(OCCO4)cc5)ccc3)cc2)C(C(NN(C)C)=O)CCC1
**1e**	N1(Cc2c(OC)cc(c3c(Cl)c(c5cc4c(OCCO4)cc5)ccc3)cc2)C(C(NCCN)=O)CCC1
**1f**	N1(Cc2c(OC)cc(c3c(Cl)c(c5cc4c(OCCO4)cc5)ccc3)cc2)C(C(NCCO)=O)CCC1
**1g**	N1(Cc2c(OC)cc(c3c(Cl)c(c5cc4c(OCCO4)cc5)ccc3)cc2)C(C(NC(CO)CO)=O)CCC1
**1h**	N1(Cc2c(OC)cc(c3c(Cl)c(c5cc4c(OCCO4)cc5)ccc3)cc2)C(C(NC(CO)(CO)CO)=O)CCC1
**2a**	N1(Cc2c(OC)cc(c3c(Cl)c(c5cc4c(OCCO4)cc5)ccc3)cc2)CC(C(O)O)CC1
**2b**	N1(Cc2c(OC)cc(c3c(Cl)c(c5cc4c(OCCO4)cc5)ccc3)cc2)CC(C(NO)=O)CC1
**2c**	N1(Cc2c(OC)cc(c3c(Cl)c(c5cc4c(OCCO4)cc5)ccc3)cc2)CC(C(NN)=O)CC1
**2d**	N1(Cc2c(OC)cc(c3c(Cl)c(c5cc4c(OCCO4)cc5)ccc3)cc2)CC(C(NN(C)C)=O)CC1
**2e**	N1(Cc2c(OC)cc(c3c(Cl)c(c5cc4c(OCCO4)cc5)ccc3)cc2)CC(C(NCCN)=O)CC1
**2f**	N1(Cc2c(OC)cc(c3c(Cl)c(c5cc4c(OCCO4)cc5)ccc3)cc2)CC(C(NCCO)=O)CC1
**2g**	N1(Cc2c(OC)cc(c3c(Cl)c(c5cc4c(OCCO4)cc5)ccc3)cc2)CC(C(NC(CO)CO)=O)CC1
**2h**	N1(Cc2c(OC)cc(c3c(Cl)c(c5cc4c(OCCO4)cc5)ccc3)cc2)CC(C(NC(CO)(CO)CO)=O)CC1
**3a**	c12c(OCCO2)cc(c3cccc(c4ccc(CN5CCCCC5C(O)O)c(OC)c4)c3Cl)cc1
**3c**	C1COc2c(O1)ccc(c3cccc(c4ccc(CN5CCCCC5C(NN)=O)c(OC)c4)c3Cl)c2
**3d**	C1COc2c(O1)ccc(c3cccc(c4ccc(CN5CCCCC5C(NN(C)C)=O)c(OC)c4)c3Cl)c2
**3f**	C1COc2c(O1)ccc(c3cccc(c4ccc(CN5CCCCC5C(NCCO)=O)c(OC)c4)c3Cl)c2
**3g**	C1COc2c(O1)ccc(c3cccc(c4ccc(CN5CCCCC5C(NC(CO)CO)=O)c(OC)c4)c3Cl)c2
**3h**	C1COc2c(O1)ccc(c3cccc(c4ccc(CN5CCCCC5C(NC(CO)(CO)CO)=O)c(OC)c4)c3Cl)c2
**4a**	c12c(OCCO2)cc(c3cccc(c4ccc(CN5CCCC(C(O)O)C5)c(OC)c4)c3Cl)cc1
**4b**	C1COc2c(O1)ccc(c3cccc(c4ccc(CN5CCCC(C(NO)=O)C5)c(OC)c4)c3Cl)c2
**4c**	C1COc2c(O1)ccc(c3cccc(c4ccc(CN5CCCC(C(NN)=O)C5)c(OC)c4)c3Cl)c2
**4d**	c12c(OCCO2)cc(c3cccc(c4ccc(CN5CCCC(C(NN(C)C)=O)C5)c(OC)c4)c3Cl)cc1
**4f**	C1COc2c(O1)ccc(c3cccc(c4ccc(CN5CCCC(C(NCCO)=O)C5)c(OC)c4)c3Cl)c2
**4g**	C1COc2c(O1)ccc(c3cccc(c4ccc(CN5CCCC(C(NC(CO)CO)=O)C5)c(OC)c4)c3Cl)c2
**4h**	C1COc2c(O1)ccc(c3cccc(c4ccc(CN5CCCC(C(NC(CO)(CO)CO)=O)C5)c(OC)c4)c3Cl)c2
**5a**	N1(CCC(C(NC(C(O)O)Cc6ccccc6)=O)CC1)Cc2c(OC)cc(c3c(Cl)c(c5cc4c(OCCO4)cc5)ccc3)cc2
**5b**	N1(CCC(C(NO)=O)CC1)Cc2c(OC)cc(c3c(Cl)c(c5cc4c(OCCO4)cc5)ccc3)cc2
**5c**	N1(CCC(C(NN)=O)CC1)Cc2c(OC)cc(c3c(Cl)c(c5cc4c(OCCO4)cc5)ccc3)cc2
**5d**	N1(CCC(C(NN(C)C)=O)CC1)Cc2c(OC)cc(c3c(Cl)c(c5cc4c(OCCO4)cc5)ccc3)cc2
**5e**	N1(CCC(C(NCCN)=O)CC1)Cc2c(OC)cc(c3c(Cl)c(c5cc4c(OCCO4)cc5)ccc3)cc2
**5f**	N1(CCC(C(NCCO)=O)CC1)Cc2c(OC)cc(c3c(Cl)c(c5cc4c(OCCO4)cc5)ccc3)cc2
**5g**	N1(CCC(C(NC(CO)CO)=O)CC1)Cc2c(OC)cc(c3c(Cl)c(c5cc4c(OCCO4)cc5)ccc3)cc2
**5h**	N1(CCC(C(NC(CO)(CO)CO)=O)CC1)Cc2c(OC)cc(c3c(Cl)c(c5cc4c(OCCO4)cc5)ccc3)cc2

Table S2. Diffraction data collection and refinement statistics for PD-L1 complex with 2f.

**Wavelength**	0.97926
**Space group**	P 21 21 2
**Unit cell**	a=52.09, b=109.99, c=51.82, alpha=90.00, beta=90.00, gamma=90.00
**Resolution range**	46.88 - 2.17 (2.24-2.17)
**Total reflections**	97447 (8682)
**Unique reflections**	16333 (1376)
**Multiplicity**	6.00 (6.30)
**Completeness**	99.70 (100.00)
**mean(I) / sig(I)**	6.80 (1.60)
**Wilson B-factor**	40.10
**Rmerge**	0.15 (0.79)
**Rmeas**	0.17 (0.86)
**Rpim**	0.07 (0.34)
**CChalf**	0.99 (0.73)
**R/Rfree**	0.25/0.28
**Ramachandran favoured (%)**	96.0
**Ramachandran allowed (%)**	3.6
**Ramachandran outliers (%)**	0.4
**Rotamer outliers**	0.9
**Clash score**	3.4
**MolProbity score**	1.41

Statistics for the last shell is given in parentheses.

Table S3. Crystal data and final refinement results for structure 2a

	**2a**
Empirical moiety formula	C_{27} H_{26} Cl N O_5, 3H_2 O
Formula weight [g/mol]	533.98
Crystal system	Monoclinic
Space group	I2/*a*
Unite cell dimensions	a = 11.0216(1) Å<br>b = 10.6260(1) Å<br>c = 43.5391(6) Å<br>α=90°<br>β=93.556(1)°<br>γ=90°
Volume [Å^3]	5089.29(10)
Z	8
D_{calc} [Mg/m^3]	1.394
μ [mm^{-1}]	1.775
F(000)	2256
Crystal size [mm^3]	0.6 x 0.2 x 0.02
Θ range	4.069° to 80.555°
Index ranges	-13 ≤ h ≤ 10,<br>-13 ≤ k ≤ 13,<br>-55 ≤ l ≤ 54
Refl. Collected	30432
Independent reflections	5532<br>[R(int) = 0.0447]
Completeness [%] to Θ	98.9 (Θ 80.555°)
Absorption correction	Multi-scan
Tmin. and Tmax.	0.944 and 1.000
Data/ restraints/parameters	5532 / 16 / 452
GooF on F2	1.063
Final R indices [I>2sigma(I)]	R1= 0.0488,<br>wR2= 0.1298
R indices (all data)	R1= 0.0512,<br>wR2= 0.1316
$\Delta\rho_{max}$, $\Delta\rho_{min}$ [e·Å^{-3}]	0.677 and -0.407

Table S4. Geometrical parameters of hydrogen bonds observed in the crystal structure of 2a [Å and °].

D-H...A	d(D-H)	d(H...A)	d(D...A)	<(DHA)
C(32)-H(32B)...O(2WA)#3	0.98	2.54	3.405(17)	147.2
C(23)-H(23A)...O(29B^b)	0.99	2.53	3.239(17)	128.1
C(11)-H(11A)...O(2W)#4	0.99	2.58	3.456(5)	147.5
C(11)-H(11A)...O(2WA)#4	0.99	2.50	3.438(6)	158.4
C(11)-H(11B)...O(31)#5	0.99	2.57	3.266(2)	127.6
C(25A^a)-H(25A^a)...O(1W)#6	0.99	2.60	3.497(17)	150.3
C(28A^a)-H(28B^a)...O(13)#5	0.99	2.45	3.151(17)	127.3
C(25B^b)-H(25D^b)...O(3W)#6	0.99	2.45	3.33(2)	146.6
C(25B^b)-H(25C^b)...O(29B^b)	0.99	2.54	3.05(3)	111.8
C(28B^b)-H(28D^b)...O(13)#5	0.99	2.37	3.161(17)	136.8
O(1W)-H(11W)...O(3W)	0.896(18)	2.61(4)	3.109(5)	116(3)
O(1W)-H(11W)...O(30A^a)	0.896(18)	1.98(2)	2.865(6)	169(4)
O(1W)-H(12W)...O(2W)#7	0.840(19)	1.78(2)	2.590(10)	163(4)
O(1W)-H(12W)...O(2WA)#7	0.840(19)	2.26(3)	3.024(12)	152(4)
O(2W)-H(23W)...O(30A^a)	1.19(5)	1.95(3)	2.992(11)	144(6)
O(2W)-H(23W)...O(30B^b)	1.19(5)	1.72(4)	2.892(13)	166(7)
O(2WA)-H(23W)...O(30A^a)	0.88(2)	1.95(3)	2.804(7)	164(7)
O(2WA)-H(23W)...O(30B^b)	0.88(2)	1.72(4)	2.527(10)	151(8)
O(2WA)-H(24W)...O(1W)#7	0.85(2)	2.24(5)	3.024(12)	152(9)
O(2W)-H(22W)...O(4W)	0.88(2)	1.88(3)	2.726(11)	161(7)
O(3W)-H(31W)...O(30A^a)	0.81(3)	1.80(5)	2.496(6)	143(7)
O(3W)-H(31W)...O(30B^b)	0.81(3)	2.04(4)	2.763(7)	147(7)
O(4W)-H(41W)...O(1W)	0.85(2)	1.91(5)	2.607(6)	138(7)
O(4W)-H(42W)...O(1W)#2	0.86(2)	2.09(5)	2.852(5)	147(7)
N(24)-H(24)...O(31)	0.90(3)	2.57(3)	3.033(2)	113(2)
N(24)-H(24)...O(29A^a)#3	0.90(3)	1.88(3)	2.722(16)	155(3)
N(24)-H(24)...O(29B^b)#3	0.90(3)	1.78(3)	2.636(18)	158(3)

Symmetry transformations used to generate equivalent atoms:

#1 -x+5/2,y,-z+1 #2 -x+3/2,y,-z+1 #3 x-1/2,-y+1,z

#4 -x+2,y-1/2,-z+3/2 #5 -x+2,y+1/2,-z+3/2 #6 -x+2,-y+1,-z+1

#7 -x+2,-y+2,-z+1

3. SUPPORTING FIGURES

Figure S1. The dose-dependent bioactivity of the molecules in the in vitro PD-1/PD-L1 Immune Checkpoint Blockade (ICB) assay. The graphs present the fold induction of the activation of Jurkat-ECs cells cocultured with the stimulator CHO/TCRAct/PD-L1 cells in the presence of different concentrations of the indicated compounds. DMSO-treated cells were used as a control for the compound treatments. Data points represent mean ± SD values from duplicates. The concentration range that evokes increasingly toxic effects on the cells is highlighted in red.

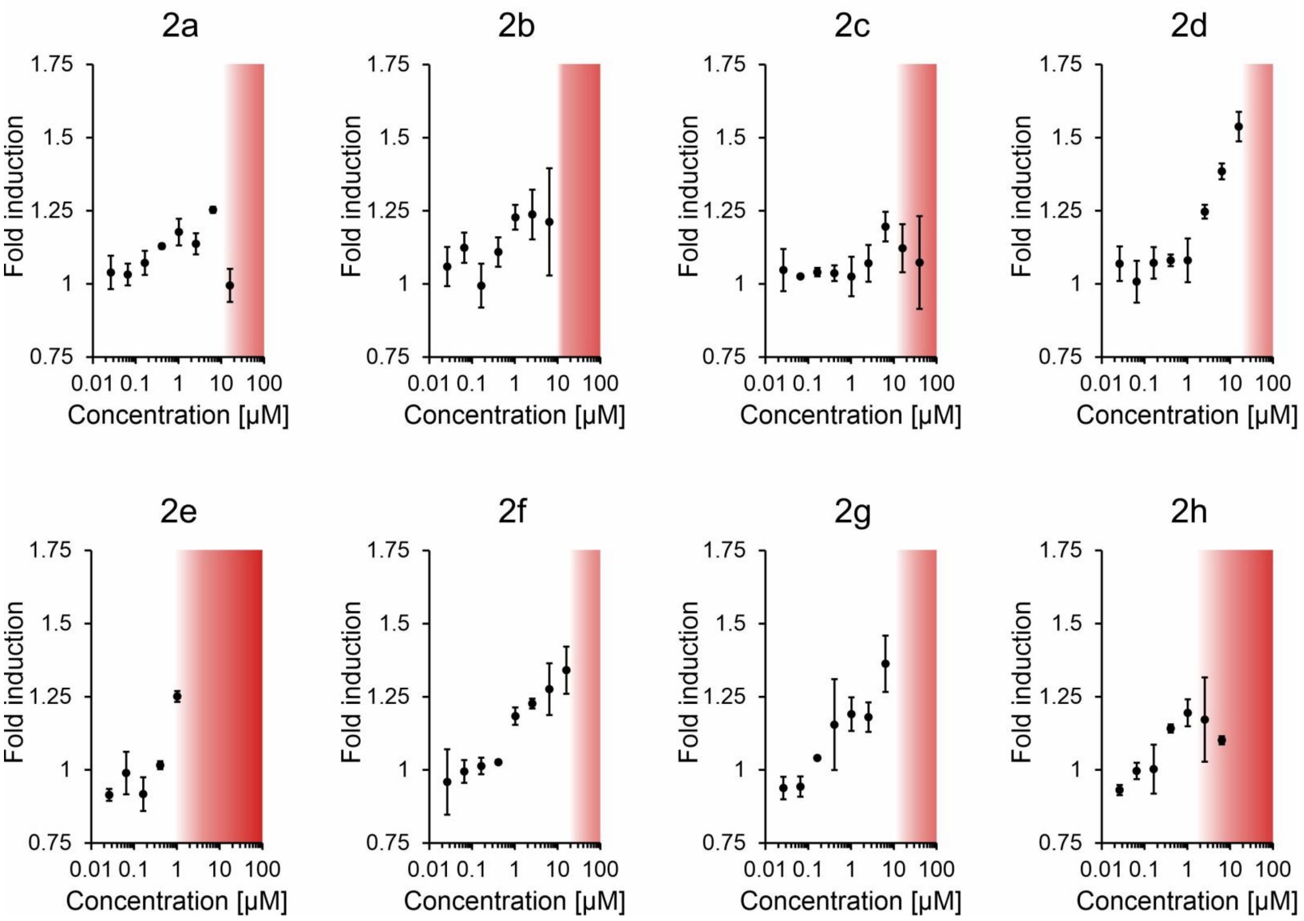

Figure S2. The asymmetric unit of 2a crystal structure with O2W, O3W, and O4W water molecules shown in the two alternative positions. The highly disordered water channel leads to disorder of the solubilizing-tag fragment with the refined site occupancies 54% and 46% (the last shown as green sphere representation).

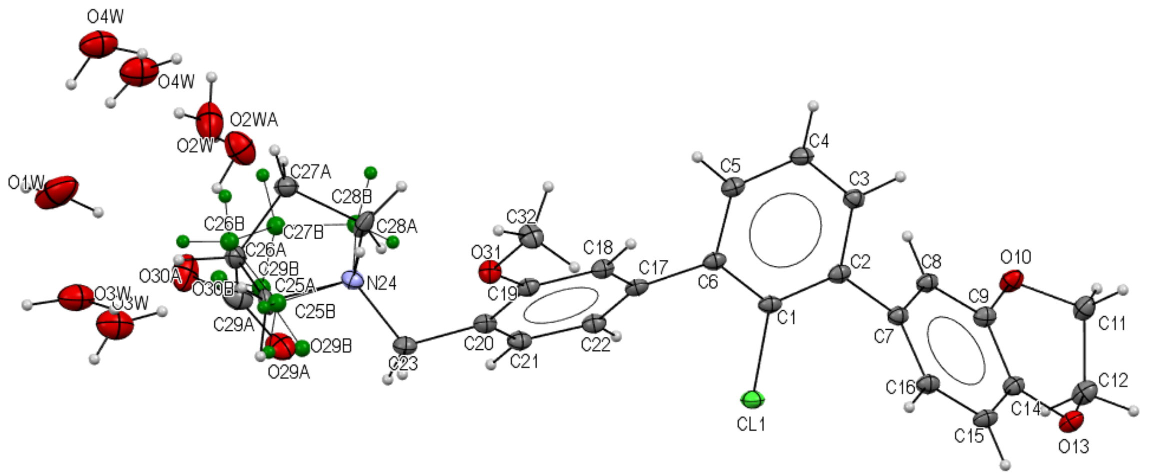

Figure S3. Packing scheme in the crystal in the structure of 2a. View along [100]. Water channels propagating in [100] are represented by cumulated red ellipsoids (oxygen atoms). Displacement ellipsoids of non-hydrogen atoms are drawn at the 30% probability level. Hydrogen atoms are not shown for figure clarity.

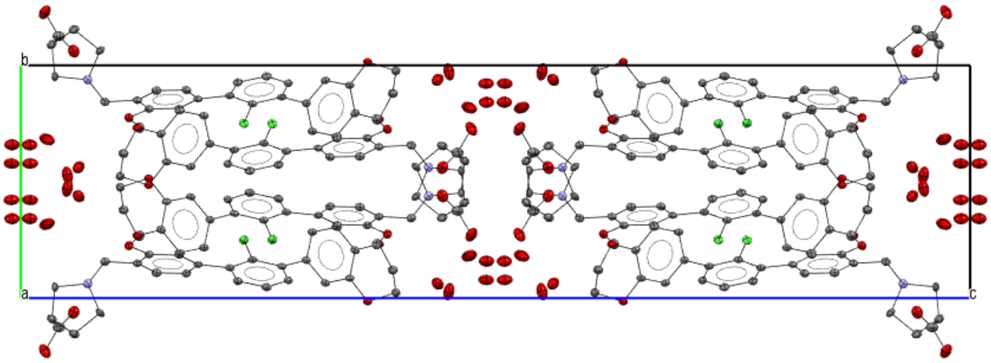

Figure S4. Packing scheme in the crystal in the structure of 2a. View along [100]. Displacement ellipsoids of non-hydrogen atoms are drawn at the 30% probability level. Hydrogen atoms are not shown for figure clarity.

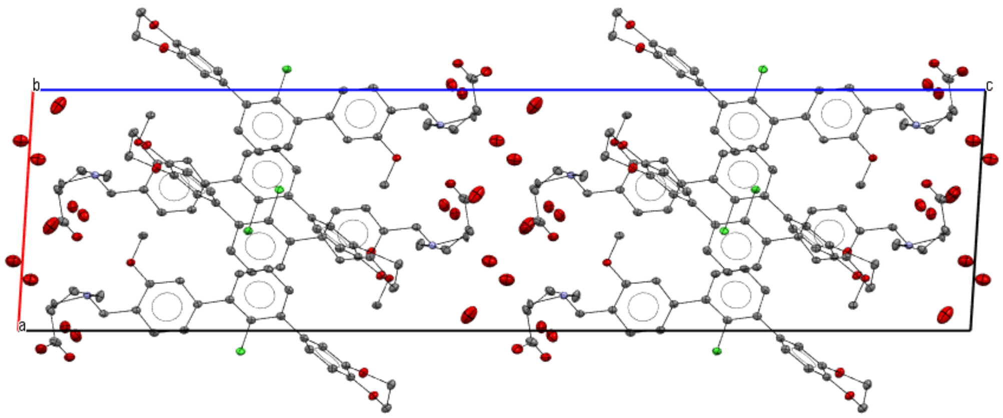

Figure S5. Distribution of torsion angle corresponding to C1-C2-C7-C16 (TOR1) in structures deposited in CSD, which contain *meta*-terphenyl moiety

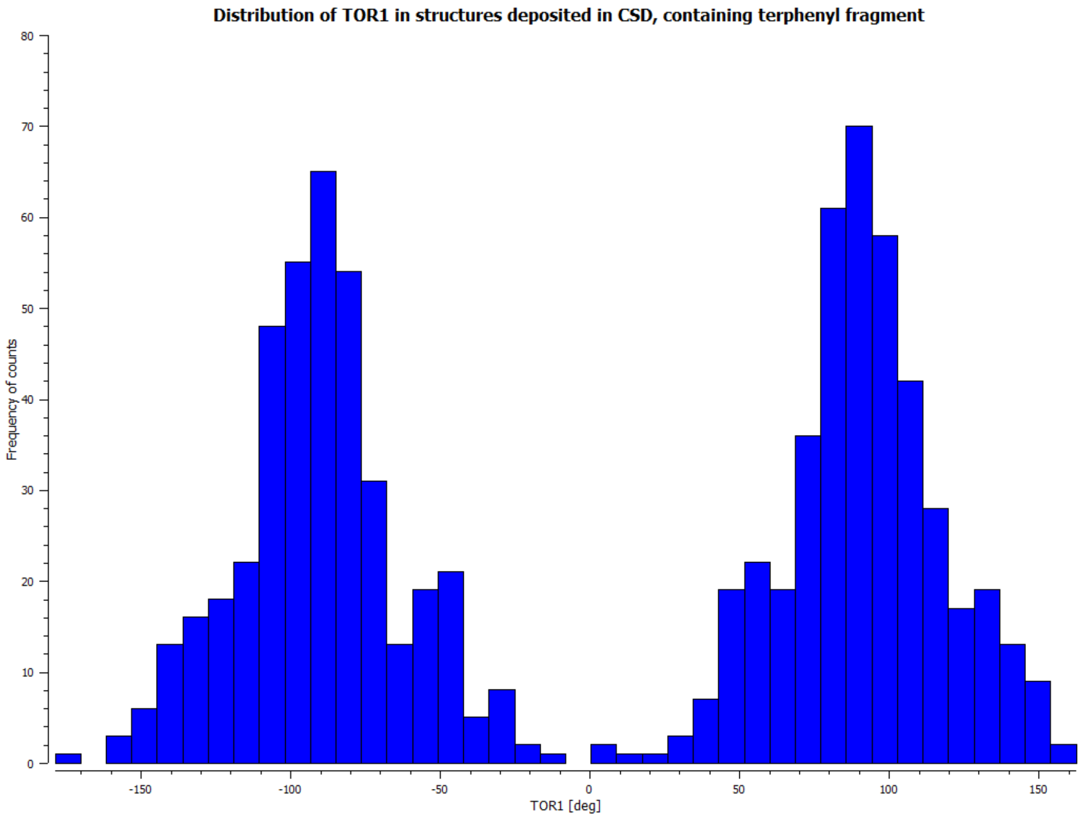

Figure S6. Distribution of torsion angle corresponding to C1-C6-C17-C22 (TOR2) in structures deposited in CSD, which contain *meta*-terphenyl moiety

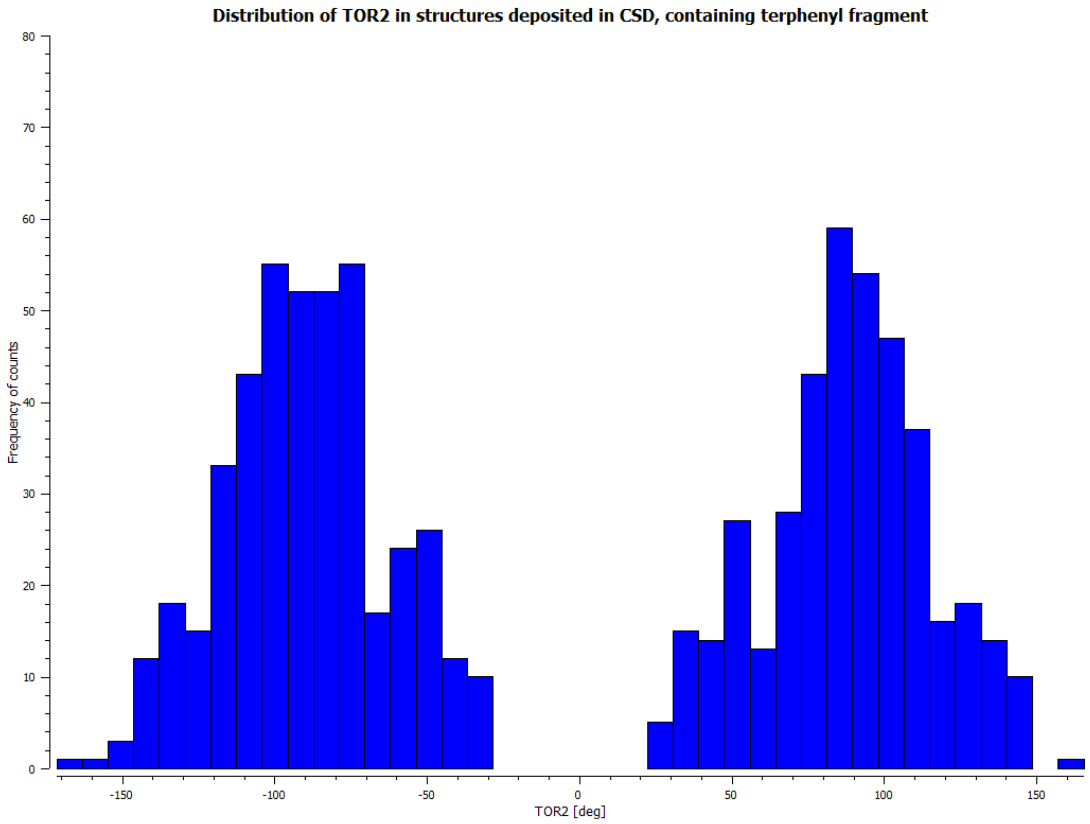

Figure S7. Distribution of mutual angle between mean planes defined by carbon atoms of aromatic rings 1 and 2 (ANG1) in structures deposited in CSD, which contain *meta*-terphenyl moiety

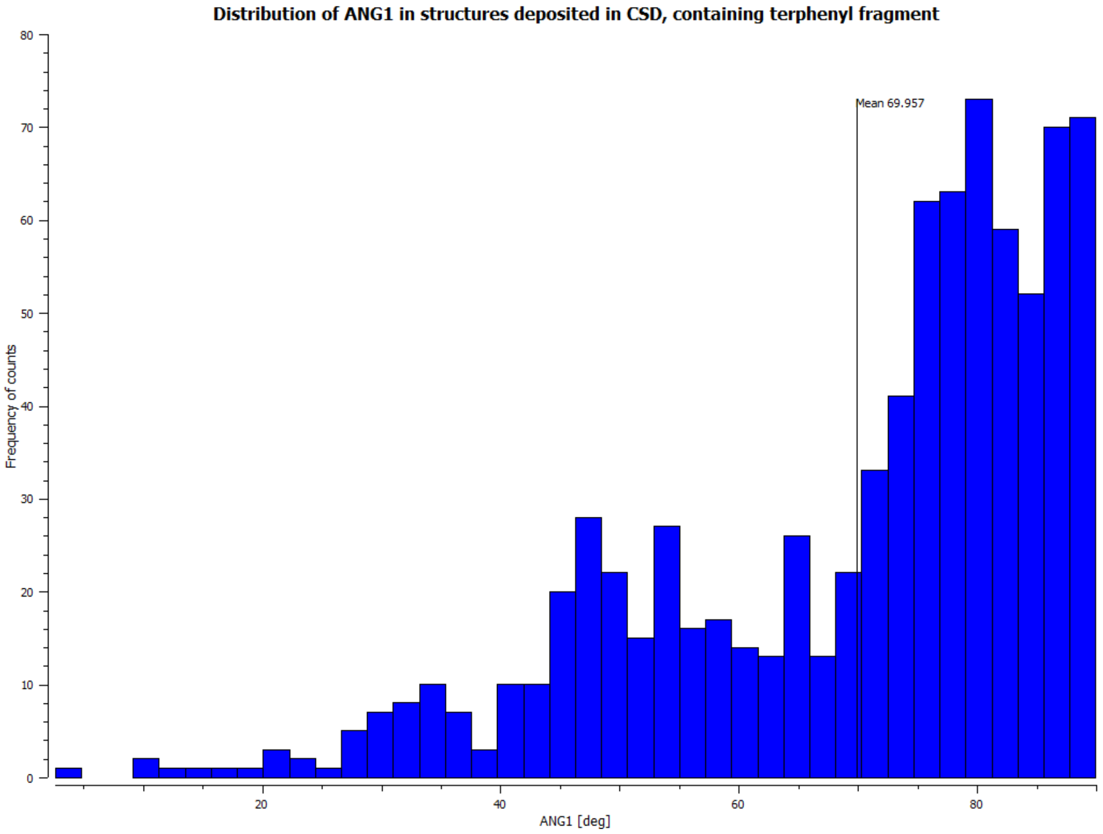

Figure S8. Distribution of mutual angle between mean planes defined by carbon atoms of aromatic rings 1 and 3 (ANG2) in structures deposited in CSD, which contain *meta*-terphenyl moiety

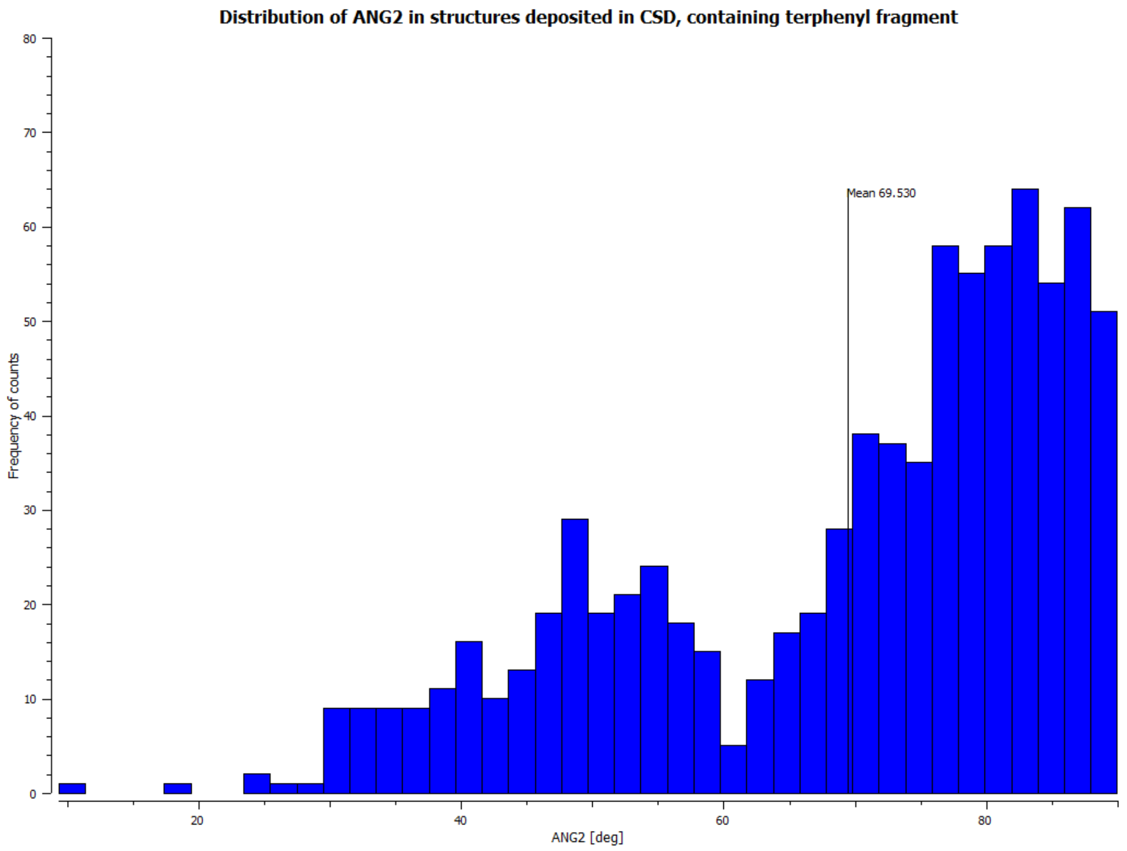

Figure S9. Distribution of mutual angle between mean planes defined by carbon atoms of aromatic rings 2 and 3 (ANG3) in structures deposited in CSD, which contain *meta*-terphenyl moiety.

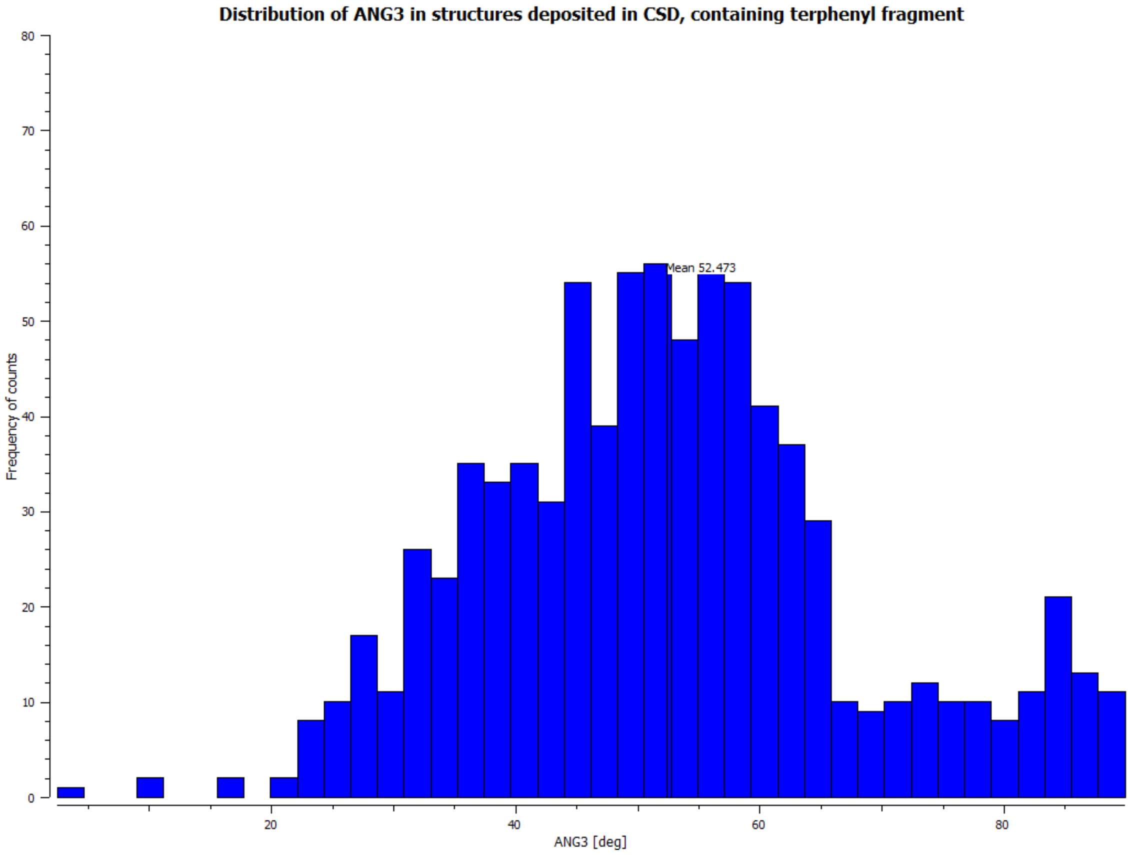

4. EXPERIMENTAL DETAILS

4.1. Compounds synthesis

All the reagents and solvents which were used for synthesis and characterization of the compounds were received from commercial suppliers such as Sigma Aldrich, Alfa-Aesar, Acros, Ambeed, and others, they were used without further purification. During experiments no unexpected or unusually high safety hazards were encountered. The NMR spectral data were recorded with Bruker Avance NMR spectrometer, ^{1}H NMR at 600, and ^{13}C NMR at 151 MHz, reporting chemical shifts (δ) in ppm and coupling constants (J) in Hz. All chemical shifts were analyzed in correspondence to the solvent peaks (DMSO-d_6, MeOD-d_4, $CDCl_3$). Thin-layer chromatography (TLC) was performed on aluminum sheets precoated with Silica Gel 60 F_{254} (Merck). Techniques of visualization of TLC plates included radiation with a UV lamp at 254 nm wavelength. Purification of compounds was conducted by flash chromatography on the Grace Reveleris X2 Flash Chromatoraphy System with the Grace Resolv Silica Cartridges. The LCMS measurements were recorded on the UPLC-MS/MS system consisting of a Waters ACQUITY UPLC coupled to a Waters TQD mass spectrometer (electrospray ionization mode ESI-tandem quadrupole). Chromatographic separations were performed with the use of the Acquity UPLC BEH C_{18} column; 2.1 × 100 mm, and 1.7 µm particle size, equipped with Acquity UPLC BEH C_{18} VanGuard pre-column; 2.1 × 5 mm, and 1.7 µm particle size. The column was maintained at 40°C, and eluted under gradient conditions using from 95% to 0% of eluent A over 10 min, at a flow rate of 0.3 mL min-1. Eluent A: water/formic acid (0.1%, v/v); eluent B: acetonitrile/formic acid (0.1%, v/v). All final compounds were determined to have at least 92% of purity. High-resolution mass spectrometry (HRMS) analyses were carried out with the microTOF-QII spectrometer using the ESI ionization technique.

General protocol of esters 1-5 synthesis

m-terphenyl core ((2'-chloro-3'-(2,3-dihydrobenzo[b][1,4]dioxin-6-yl)-3-methoxy-[1,1'-biphenyl]-4-yl)methanol; 1 equiv.) was placed in the round bottom flask in the anhydrous DCM/DMF mixture (50:1 v/v). The reaction was cooled to 0°C and the thionyl chloride was added dropwise (5 equiv.). The reaction mixture was stirred at room temperature for 2 hours and then poured into 1M NaOH and extracted with dichloromethane. The organic layers were collected, dried over anhydrous $MgSO_4$ then filtrated and evaporated. The resulting oil was placed with the round bottom flask together with appropriate amine (3 equiv.), DIPEA (2 equiv.) and anhydrous DMF. If the hydrochloride of the amine was used, the free amine was obtained in the reaction flask, prior to the addition of the crude chloride, by the reaction with triethylamine (3 equiv.). The reaction mixture was heated at 80°C for 16 hours. The reaction was poured on saturated $NaHCO_3$ solution and extracted with ethyl acetate. The organic layers were collected, washed with brine and dried with anhydrous $MgSO_4$, then filtrated and

evaporated. Crude product was purified by column chromatography giving compounds **1-5** with 51-99% yield. m-terphenyl core used in the reaction was prepared as described in Muszak et al. [3]

Methyl ((2'-chloro-3'-(2,3-dihydrobenzo[b][1,4]dioxin-6-yl)-3-methoxy-[1,1'-biphenyl]-4-yl)methyl)prolinate (1)

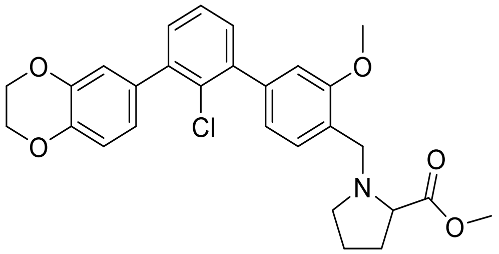

Reagent used: m-terphenyl core (1.58 g, 4.1 mmol) thionyl chloride (1.50 ml, 20.7 mmol), L-proline methyl ester hydrochloride (2.06 g, 12.4 mmol), triethylamine (1.73 ml, 12.4 mmol), DIPEA (1.44 ml, 8.3 mmol). Crude product was purified on flash chromatography (SiO_2, n-hexane:ethyl acetate 5:1) giving compound 1 as yellowish oil with 84% yield (1.72 g)

R_f = 0.47 (SiO_2, hexane/ethyl acetate 1:1); **^{1}H NMR** (600 MHz, $CDCl_3$) δ [ppm]: 7.36 (d, J = 7.6 Hz, 1H), 7.33-7.28 (m, 3H), 7.01-6.99 (m, 2H), 6.96-6.95 (m, 1H), 6.94-6.91 (m, 2H), 4.30 (s, 4H), 3.88 (q, J = 13.4 Hz, 2H), 3.82 (s, 3H), 3.67 (s, 3H), 3.33-3.28 (m, 1H), 3.22-3.16 (m, 1H), 2.51 (q, J = 8.3 Hz, 1H), 2.18-2.09 (m, 1H), 1.99-1.90 (m, 2H), 1.82-1.75 (m, 1H); **^{13}C NMR** (151 MHz, $CDCl_3$) δ [ppm]: 174.8, 157.3, 143.3, 143.1, 141.7, 141.2, 140.5, 133.5, 131.1, 130.5, 130.3, 126.4, 122.9, 121.6, 118.7, 117.0, 112.1, 65.1, 64.6, 64.5, 55.5, 53.4, 51.9, 51.5, 29.6, 23.0; **IR (ATR)** [cm^{-1}]: 3073, 2948, 1731, 1605, 1582, 1506, 1301, 1279, 1235, 1067, 1042, 849, 801, 745

Methyl 1-((2'-chloro-3'-(2,3-dihydrobenzo[b][1,4]dioxin-6-yl)-3-methoxy-[1,1'-biphenyl]-4-yl)methyl)pyrrolidine-3-carboxylate (2)

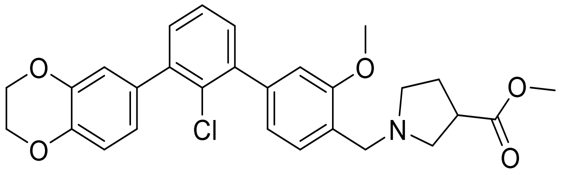

Reagent used: m-terphenyl core (2.00 g, 5.2 mmol) thionyl chloride (1.90 ml, 26.1 mmol), DL-β-proline methyl ester hydrochloride (1.77 g, 10.7 mmol), triethylamine (2.18 ml, 15.7 mmol), DIPEA (1.82 ml, 10.5 mmol). Crude product was purified on column chromatography (SiO_2, chloroform/methanol 10:1) giving compound **2** as brownish oil with 99% yield (2.55 g)

R_f = 0.47 (SiO_2, chloroform/methanol 10:1); **^{1}H NMR** (600 MHz, $CDCl_3$) δ [ppm]: 7.31 (d, J = 7.7 Hz, 1H), 7.24-7.19 (m, 3H), 6.94-6.91 (m, 2H), 6.88 (d, J = 1.5 Hz, 1H), 6.87-6.82 (m, 2H), 4.20 (s, 4H), 3.75 (s, 3H), 3.64 (s, 2H), 3.60 (s, 3H), 3.02-2.93 (m, 2H), 2.77-2.72 (m, 1H), 2.63 (dd, J = 8.8, 6.9 Hz, 1H), 2.53 (q, J = 7.9 Hz, 1H), 2.04 (q, J = 8.2 Hz, 2H); **^{13}C NMR** (151 MHz, $CDCl_3$) δ [ppm]: 175.7, 156.9, 143.2, 143.1, 141.7, 141.1, 140.1, 133.5, 131.1, 130.5, 130.3, 129.9, 126.4, 126.2, 122.9, 121.6, 118.6, 116.9, 112.1, 64.51, 64.47, 57.0 55.6, 54.0, 53.2, 51.9, 42.1, 27.8; **IR (ATR)** [cm^{-1}]: 2951, 2799, 1733, 1678, 1582, 1507, 1459, 1385, 1302, 1280, 1246, 1228, 1171, 1068, 1043, 871, 797, 736

Methyl 1-((2'-chloro-3'-(2,3-dihydrobenzo[b][1,4]dioxin-6-yl)-3-methoxy-[1,1'-biphenyl]-4-yl)methyl)piperidine-2-carboxylate (3)

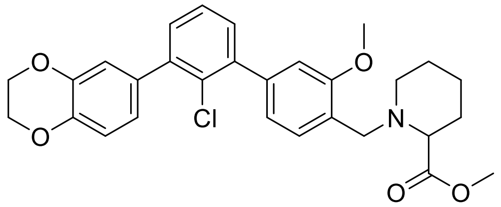

Reagent used: m-terphenyl core (1.0 g, 2.5 mmol) thionyl chloride (1.0 ml, 13.4 mmol), DL-methyl piperidine-2-carboxylate hydrochloride (1.34 g, 7.48 mmol), triethylamine (1.0 ml, 7.5 mmol), DIPEA (0.9 ml, 4.98 mmol). Crude product was purified on column chromatography (SiO_2, hexane/ethyl acetate 3:2) giving compound **3** as yellow solid with 71% yield (0.894 g).

R_f = 0.53 (SiO_2, chloroform/methanol 10:1); **^{1}H NMR** (600 MHz, $CDCl_3$) δ [ppm]: 7.44 (d, J = 5.9 Hz, 1H), 7.35 – 7.27 (m, 3H), 7.01 (dd, J = 9.0, 4.9 Hz, 2H), 6.98 – 6.89 (m, 3H), 5.30 (s, 1H), 4.31 (s, 4H), 3.81 (s, 3H), 3.75 (s, 4H), 3.25 (s, 1H), 3.08 (s, 1H), 2.28 (s, 1H), 1.86 (d, J = 33.0 Hz, 2H), 1.62 (s, 3H), 1.38 (s, 1H); **^{13}C NMR** (151 MHz, $CDCl_3$) δ [ppm]: 157.3, 143.2, 143.0, 141.0, 131.0, 130.4, 130.2, 126.3, 122.8, 121.5, 118.6, 116.8, 111.9, 64.5, 64.4, 55.4, 53.7, 51.6, 50.5, 29.8, 28.0, 25.3, 22.6; **IR (ATR)** [cm^{-1}]: 2936, 1726, 1611, 1582, 1506, 1457, 1302, 1279, 1225, 1067, 1043, 1029, 870, 795, 744

Methyl 1-((2'-chloro-3'-(2,3-dihydrobenzo[b][1,4]dioxin-6-yl)-3-methoxy-[1,1'-biphenyl]-4-yl)methyl)piperidine-3-carboxylate (4)

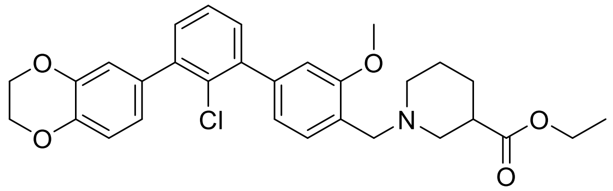

Reagent used: m-terphenyl core (2.30 g, 5.73 mmol), thionyl chloride (2.18 ml, 30.04 mmol), DL-ethyl piperidine-3-carboxylate hydrochloride (3.33 g, 17.2 mmol), triethylamine (2.4 ml, 17.2 mmol), DIPEA (2 ml, 11.46 mmol). Crude product was purified on column chromatography (SiO_2, hexane/ethyl acetate 3:2) giving compound **4** as pale yellow solid with 51% yield (1.52 g).

R_f = 0.55 (SiO_2, chloroform/methanol 10:1); **^{1}H NMR** (600 MHz, $CDCl_3$) δ [ppm]: 7.43 – 7.40 (m, J = 7.7 Hz, 1H), 7.34 – 7.28 (m, 3H), 7.01 (dd, J = 8.6, 1.7 Hz, 2H), 6.97 – 6.88 (m, J = 14.2, 4.7 Hz, 3H), 4.31 (s, 4H), 3.84 (s, 2H), 3.61 (s, 1H), 3.09 – 3.01 (m, J = 10.0 Hz, 1H), 2.86 – 2.79 (m, J = 9.6 Hz, 1H), 2.66 – 2.58 (m, J = 10.2 Hz, 1H), 2.35 – 2.28 (m, J = 12.5, 7.1 Hz, 1H), 2.19 – 2.10 (m, J = 10.2 Hz, 1H), 1.98 – 1.90 (m, J = 9.6 Hz, 1H), 1.79 – 1.70 (m, 1H), 1.60 (s, 2H), 1.52 – 1.44 (m, J = 9.0 Hz, 1H); **^{13}C NMR** (151 MHz, $CDCl_3$) δ [ppm]: 174.2, 171.1, 157.2, 143.2, 143.0, 141.6, 141.1, 133.4, 131.0, 130.4, 130.2, 126.3, 122.8, 121.6, 118.6, 116.8, 112.1, 64.5, 64.4, 60.3, 55.6, 41.7, 26.8, 24.4; **IR (ATR)** [cm^{-1}]: 2937, 1725, 1611, 1582, 1506, 1456, 1389, 1302, 1279, 1245, 1225, 1177, 1126, 1068, 1043, 1029, 870, 795, 745

Methyl 1-((2'-chloro-3'-(2,3-dihydrobenzo[b][1,4]dioxin-6-yl)-3-methoxy-[1,1'-biphenyl]-4-yl)methyl)piperidine-4-carboxylate (**5**)

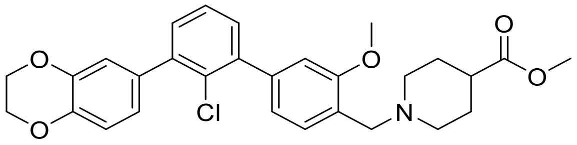

Reagent used: m-terphenyl core (1.15 g, 2.6 mmol) thionyl chloride (0.95 ml, 13.1 mmol), DL-piperidine-4-caboxylic acid ethyl ester (1.77 g, 10.7 mmol), triethylamine (1.21 ml, 7.9 mmol), DIPEA (5.24 ml, 0.91 mmol). Crude product was purified on column chromatography (SiO_2, hexane/ethyl acetate 1:1) giving compound **5** as white solid with 86% yield (1.18 g)

R_f = 0.23 (SiO_2, hexane/ethyl acetate 1:1); **^{1}H NMR** (600 MHz, $CDCl_3$) δ [ppm]: 7.43 (d, J = 8.6 Hz, 1H), 7.35-7.27 (m, 3H), 7.01 (dd, J = 7.6, 1.6 Hz, 2H), 7.00 (d, J = 1.8 Hz, 1H), 6.98-6.90 (m, 2H), 4.31 (s, 4H), 4.13 (q, J = 7.1 Hz, 2H), 3.84 (bs, 2H), 2.99-2.94 (m, 2H), 2.35-2.26 (m, 1H), 2.23-2.10 (m, 2H), 1.93-1.87 (m, 3H), 1.86-1.76 (m, 2H), 1.25 (t, J = 7.1 Hz, 3H); **^{13}C NMR** (151 MHz, $CDCl_3$) δ [ppm]: 157.3, 143.3, 143.1, 141.7, 141.2, 133.5, 131.1, 130.5, 130.3, 126.4, 122.9, 121.7, 118.7, 110.7, 112.2, 64.6, 64.5, 60.4, 56.3, 55.7, 53.3, 41.4, 28.5, 27.1, 14.4; **IR (ATR)** [cm^{-1}]: 2935, 1727, 1582, 1506, 1457, 1381, 1302, 1279, 1245, 1226, 1179, 1166, 1068, 1044, 1028, 919, 890, 870, 794, 744

General protocol of hydrolysis (1a-5a)

Appropriate ester **1**-**5** (1 equiv.) was placed in round-bottom flask with the LiOH monohydrate (1.5-4 equiv.) and dioxane/water (2:1 v/v) mixture. The reaction was heated at 60°C for 16 hours. After reaction completion, water was added and pH was adjusted to approx. 6.5 with 1M HCl, precipitated solid was collected by filtration or the mixture was extracted with ethyl acetate. Organic layers were collected, washed with brine, dried over anhydrous $MgSO_4$, filtered and evaporated. The crude product was obtained with sufficient purity or purified by column chromatography giving compounds **1a**-**5a** with 59-87% yield.

((2'-chloro-3'-(2,3-dihydrobenzo[b][1,4]dioxin-6-yl)-3-methoxy-[1,1'-biphenyl]-4-yl)methyl)proline (**1a**)

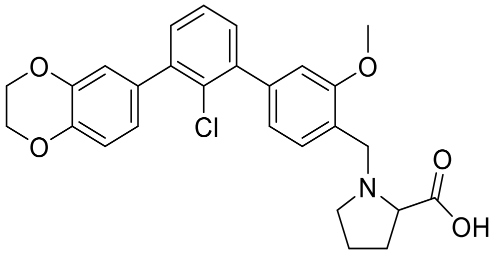

Reagent used: compound **1** (0.340 g, 0.69 mmol), LiOH monohydrate (0.043 g, 1.03 mmol). Crude product was purified by column chromatography (SiO_2 ethyl acetate/methanol 1:1) giving compound **1a** as white solid with 59% yield (0.195 g).

R_f = 0.24 (SiO_2, ethyl acetate/methanol 1:1); **^{1}H NMR** (600 MHz, $CDCl_3$) δ [ppm]: 7.35 (d, J = 8.1 Hz, 1H), 7.34-7.31 (m, 2H), 7.27-7.25 (m, 1H), 7.06-7.00 (m, 2H), 6.98 (t, J = 1.2 Hz, 1H), 6.92 (d, J

= 1.2 Hz, 2H), 4.38 (d, J = 12.6 Hz, 1H), 4.30 (s, 4H), 4.06 (d, J = 12.6 Hz, 1H), 3.94 (s, 3H), 3.84 (dd, J = 9.2, 4.2 Hz, 1H), 3.53-3.46 (m, 1H), 2.92-2.84 (m, 1H), 2.43-2.27 (m, 2H), 2.01-1.92 (m, 2H); **^{13}C NMR** (151 MHz, $CDCl_3$) δ [ppm]: 171.7, 157.6, 143.5, 143.4, 143.2, 141.3, 140.8, 133.2, 131.6, 131.1, 130.9, 130.1, 126.6, 122.9, 122.3, 119.2, 118.7, 117.0, 112.7, 68.5, 64.6, 64.5, 55.9, 54.4, 54.0, 29.4, 24.1; **IR (ATR)** [cm^{-1}]: 3391(broad), 2975, 2877, 1615, 1582, 1507, 1457, 1390, 1303, 1280, 1246, 1230, 1128, 1067, 1042, 872, 794; **LC-MS (DAD/ESI):** t_R = 5.66 min, calcd for. $C_{27}H_{26}ClNO_5$ (m/z) $[M+H]^+$ 480.16; found $[M+H]^+$ 480.25; **HRMS (ESI):** calcd for $C_{27}H_{26}ClNO_5$ [m/z] $[M+H]^+$ 480.1572; found, $[M+H]^+$ 480.1576

1-((2'-chloro-3'-(2,3-dihydrobenzo[b][1,4]dioxin-6-yl)-3-methoxy-[1,1'-biphenyl]-4-yl)methyl)pyrrolidine-3-carboxylic acid (**2a**)

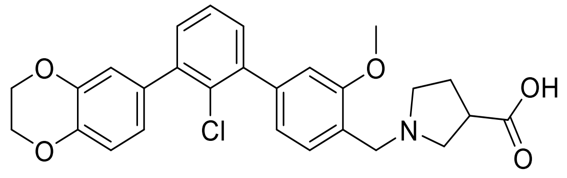

Reagent used: compound **2** (1.50 g, 3.0 mmol), LiOH monohydrate (0.19 g, 4.6 mmol). Crude product was collected by filtration giving compound **2a** as white solid with 77% yield (1.12 g).

R_f = 0.27 (SiO_2, ethyl acetate/methanol 4:1); **^{1}H NMR** (600 MHz, $CDCl_3$) δ [ppm]: 7.62 (d, J = 7.7 Hz, 1H), 7.35-7.25 (m, 3H), 7.03 (dd, J = 7.7, 1.6 Hz, 1H), 7.01-6.97 (m, 2H), 6.96-6.90 (m, 2H), 6.38 (bs, 1H), 4.35 (d, J = 12.7 Hz, 1H), 4.31 (s, 4H), 4.12 (d, J = 12.6 Hz, 1H), 3.97 (d, J = 10.4 Hz, 1H), 3.86 (s, 3H), 3.43-3.33 (m, 1H), 3.11-3.04 (m, 1H), 2.86 (t, J = 9.2 Hz, 1H), 2.79 (q, J = 9.4 Hz, 1H), 2.55-2.46 (m, 1H), 2.27-2.18 (m, 1H); **^{13}C NMR** (151 MHz, $CDCl_3$) δ [ppm]: 177.7, 157.4, 143.3, 143.1, 142.5, 141.23, 131.16, 133.3, 132.5, 131.0, 130.8, 130.2, 126.5, 122.9, 122.3, 120.1, 118.7, 117.0, 112.4, 64.6, 64.5, 56.9, 55.7, 51.3, 50.9, 43.3, 27.0; **IR (ATR)** [cm^{-1}]: 3382, 2970, 2876, 1613, 1583, 1507, 1457, 1389, 1303, 1280, 1246, 1230, 1128, 1068, 1043, 872, 795, 731; **LC-MS (DAD/ESI):** t_R = 5.48 min, calcd for. $C_{27}H_{26}ClNO_5$ (m/z) $[M+H]^+$ 480.16; found $[M+H]^+$ 480.25; **HRMS (ESI):** calcd for $C_{27}H_{26}ClNO_5$ [m/z] $[M+H]^+$ 480.1572; found, $[M+Na]^+$ 480.1578

1-((2'-chloro-3'-(2,3-dihydrobenzo[b][1,4]dioxin-6-yl)-3-methoxy-[1,1'-biphenyl]-4-yl)methyl)piperidine-2-carboxylic acid (3a)

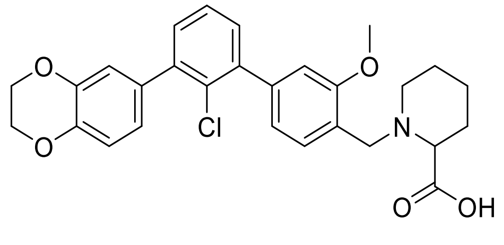

Reagent used: compound **3** (1.11 g, 2.2 mmol), LiOH monohydrate (0.08 g, 3.28 mmol). Crude product was collected by filtration giving compound **3a** as white solid with 87% yield (0.94 g).

R_f = 0.53, (SiO_2, chloroform/methanol 5:1); **^{1}H NMR** (600 MHz, MeOD-d_4) δ [ppm]: 7.53 (d, J = 7.5 Hz, 1H), 7.34 (t, J = 7.5 Hz, 1H), 7.29 (dd, J = 7.6, 1.9 Hz, 1H), 7.27 (dd, J = 7.4, 1.9 Hz, 1H), 6.97-6.92 (m, 2H), 6.89-6.78 (m, 3H), 4.26 (s, 4H), 3.87 (d, J =

13.3 Hz, 1H), 3.81 (s, 3H), 3.60 (d, J = 13.3 Hz, 1H), 2.96 (dt, J = 11.7, 3.7 Hz, 1H), 2.81 (dd, J = 10.5, 3.2 Hz, 1H), 2.03-1.98 (m, 1H), 1.93-1.89 (m, 1H), 1.75-1.68 (m, 2H), 1.56-1.54 (m, 2H), 1.31-1.26 (m, 1H); **^{13}C NMR** (151 MHz, MeOD-d_4) δ [ppm]: 181.9, 159.0, 144.7, 144.5, 143.1, 142.6, 141.7, 134.6, 132.7, 131.9, 131.5, 131.2, 127.6, 126.2, 123.6, 122.5, 119.4, 117.7, 113.0, 71.1, 65.71, 65.65, 56.0, 54.2, 52.5, 31.5, 26.4, 25.1; **IR (ATR)** [cm^{-1}]: 3354, 2935, 1582, 1507, 1417, 1323, 1303, 1280, 1246, 1228, 1126, 1067, 941, 920, 864, 793; **LC-MS (DAD/ESI):** t_R = 5.74 min, calcd for. $C_{28}H_{28}ClNO_5$ (m/z) $[M+H]^+$ 494.17; found $[M+H]^+$ 494.27; **HRMS (ESI):** calcd for $C_{28}H_{28}ClNO_5$ [m/z] $[M+H]^+$ 494.1729; found, $[M+H]^+$ 494.1728

1-((2'-chloro-3'-(2,3-dihydrobenzo[b][1,4]dioxin-6-yl)-3-methoxy-[1,1'-biphenyl]-4-yl)methyl)piperidine-3-carboxylic acid (4a)

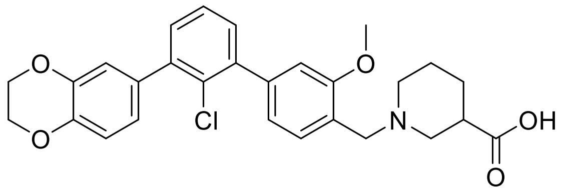

Reagent used: compound **4** (1.52 g, 3.0 mmol), LiOH monohydrate (0.1 g, 4.35 mmol). Crude product was collected by filtration giving compound **4a** as white solid with 81% yield (0.89 g).

R_f = 0.34, (SiO_2, chloroform/methanol 5:1); **^{1}H NMR** (600 MHz, $CDCl_3$) δ [ppm]: 7.73 (d, J = 7.7 Hz, 1H), 7.34 – 7.31 (m, 2H), 7.28 (dd, *J* = 6.7, 2.7 Hz, 1H), 7.08 (d, J = 7.7 Hz, 1H), 7.03 (d, J = 1.6 Hz, 1H), 6.97 (s, 1H), 6.91 (s, 2H), 4.37 (d, J = 12.9 Hz, 1H), 3.29 (s, 5H), 3.80 – 3.78 (m, 1H), 3.46 – 3.44 (m, 2H), 2.86 (t, *J* = 12.2 Hz, 1H), 2.66 (t, *J* = 13.5 Hz, 1H), 2.28 (s_b, 2H), 1.91 – 1.89 (m, 1H), 1.51 – 1.46 (m, 1H); **^{13}C NMR** (151 MHz, $CDCl_3$) δ [ppm]: 173.4, 158.0, 144.1, 143.4, 143.1, 141.3, 140.7, 133.9, 133.2, 131.1, 130.8, 130.1, 126.7, 122.9, 122.6, 118.7, 117.0, 115.5, 112.8, 64.6, 64.5, 56.0, 54.70, 53.2, 51.3, 39.1, 25.6, 22.3; **IR (ATR)** [cm^{-1}]: 3368, 2935, 2537, 1721, 1613, 1581, 1507, 1454, 1386, 1302, 1279, 1244, 1230, 1127, 1066, 1041, 1025, 942, 870, 795, 745; **LC-MS (DAD/ESI):** t_R = 5.65 min, calcd for. $C_{28}H_{28}ClNO_5$ (m/z) $[M+H]^+$ 494.17; found $[M+H]^+$ 494.27; **HRMS (ESI):** calcd for $C_{28}H_{28}ClNO_5$ [m/z] $[M+H]^+$ 494.1729; found, $[M+H]^+$ 494.1729

1-((2'-chloro-3'-(2,3-dihydrobenzo[b][1,4]dioxin-6-yl)-3-methoxy-[1,1'-biphenyl]-4-yl)methyl)piperidine-4-carboxylic acid (5a)

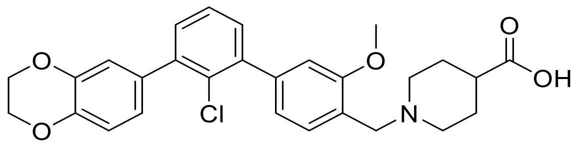

Reagent used: compound **5** (0.169 g, 0.32 mmol), LiOH monohydrate (0.055 g, 1.30 mmol). Crude product was purified by crystallization in cyclohexane/ethyl acetate giving **5a** as white solid with 78% yield (0.123 g).

R_f = 0.08, (SiO_2, ethyl acetate/methanol 1:1); **^{1}H NMR** (600 MHz, DMSO-d_6) δ [ppm]: 7.42 (t, J = 7.5 Hz, 1H), 7.39-7.31 (m, 3H), 7.02 (s, 1H), 6.99 (d, J = 7.6 Hz, 1H), 6.96-6.87 (m, 3H), 4.28 (s, 4H), 3.79 (s, 3H), 3.48 (s, 2H), 2.84-2.79 (m, 2H), 2.24-2.16 (m, 1H), 2.09-2.03 (m, 2H), 1.82-1.76 (m,

2H), 1.63-1.53 (m, 2H); **^{13}C NMR** (151 MHz, DMSO-d_6) δ [ppm]: 176.2, 156.8, 143.1, 142.9, 141.1, 140.5, 139.2, 132.5, 130.5, 130.3, 129.9, 129.2, 127.0, 125.5, 122.4, 121.2, 118.0, 116.7, 112.0, 64.15, 64.11, 55.51, 55.48, 52.7, 40.3, 28.1; **IR (ATR)** [cm^{-1}]: 3362, 2963, 1585, 1510, 1466, 1450, 1376, 1350, 1324, 1303, 1280, 1245, 1235, 1134, 1109, 1068, 1041, 1026, 973, 926, 890, 871, 789, 783, 747, 728, 668; **LC-MS (DAD/ESI):** t_R = 5.76 min, calcd for. $C_{28}H_{28}ClNO_5$ (m/z) $[M+H]^+$ 494.17; found $[M+H]^+$ 494.21; **HRMS (ESI):** calcd for $C_{28}H_{28}ClNO_5$ [m/z] $[M+H]^+$ 494.1723; found, $[M+H]^+$ 494.1735

General procedure for aminolysis

Ester **1-5** (1 equiv.) was placed in round bottom flask equipped with reflux condenser together with excess of appropriate amine component, potassium carbonate (1.5 equiv. if needed) and then heated in methanol or ethanol at 60°C for 24 hours. Then water was added and reaction mixture was extracted with ethyl acetate. Organic layers were collected, washed with brine and dried over anhydrous $MgSO_4$, then filtrated and evaporated. Crude product was purified on column chromatography giving final compounds: **1b**, **1c**, **1f, 1g**, **2c**, **2f**, **2g**, **3c**, **3f**, **3g**, **4b**, **4c**, **5b**, **5c**, **5f**, **5g** with 7-96% yield.

1-((2'-chloro-3'-(2,3-dihydrobenzo[b][1,4]dioxin-6-yl)-3-methoxy-[1,1'-biphenyl]-4-yl)methyl)-N-hydroxypyrrolidine-2-carboxamide (**1b**)

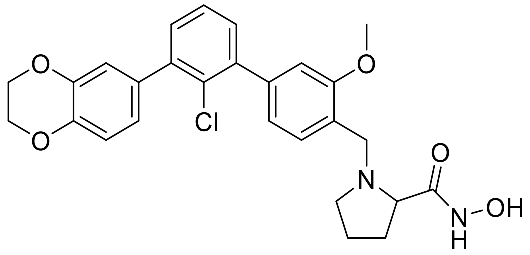

Reagent used: Ester **1** (0.200 g, 0,41 mmol), hydroxylamine 50% wt. in water (0.25 ml, 4.05 mmol), K_2CO_3 (0.084 g, 0.61 mmol), ethanol (10 ml). Crude product was purified on column chromatography (SiO_2, chloroform/methanol 20:1) giving final product **1b** as yellowish solid with 16% yield (0.032 g).

R_f = 0.61 (SiO_2, chloroform/methanol 10:1); **^{1}H NMR** (600 MHz, $CDCl_3$) δ [ppm]: 7.47 (d, J = 7.7 Hz, 1H), 7.37-7.33 (m, 2H), 7.29-7.26 (m, 1H), 7.11 (dd, J = 7.7, 1.4 Hz, 1H), 7.07 (d, J = 1.2 Hz, 1H), 6.98 (s, 1H), 6.93 (d, J = 1.1 Hz, 2H), 4.84 (d, J = 13.0 Hz, 1H), 4.70 (d, J = 13.0 Hz, 1H), 4.31 (s, 4H), 4.01 (t, J = 9.8 Hz, 1H), 3.91 (s, 3H), 3.61-3.55 (m, 1H), 3.46 (q, J = 9.8 Hz, 1H), 2.59-2.49 (m, 1H), 2.46-2.36 (m, 1H), 2.36-2.26 (m, 1H), 2.06 (s_b, 1H); **^{13}C NMR** (151 MHz, $CDCl_3$) δ [ppm]: 170.3, 158.0, 144.8, 143.4, 143.2, 141.4, 140.5, 134.5, 133.1, 131.3, 130.8, 130.0, 126.7, 122.9, 122.6, 118.6, 117.1, 115.3, 113.0, 70.9, 64.6, 64.5, 63.4, 63.1, 55.9, 26.4, 20.6; **LC-MS (DAD/ESI): t_R** = 6.26 min, calcd for. $C_{27}H_{27}ClN_2O_5$ (m/z) $[M+H]^+$ 495.17; found $[M+H]^+$ 496.26; **HRMS (ESI):** calcd for $C_{27}H_{27}ClN_2O_5$ [m/z] $[M]^+$ 495.1681; found, $[M]^+$ 495.1680

1-((2'-chloro-3'-(2,3-dihydrobenzo[b][1,4]dioxin-6-yl)-3-methoxy-[1,1'-biphenyl]-4-yl)methyl)pyrrolidine-2-carbohydrazide (1c)

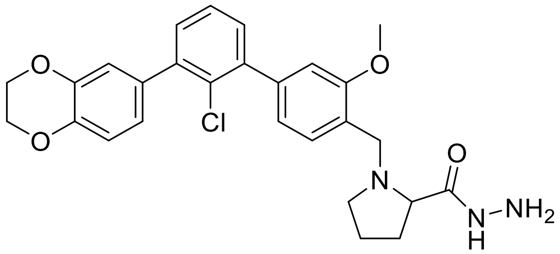

Reagent used: Ester **1** (0.200 g, 0,41 mmol), hydrazine monohydrate (0.79 ml, 4.1 mmol), methanol (8 ml). Crude product was purified on column chromatography (SiO_2, chloroform/methanol 20:1) giving final product **1c** as white solid with 96% yield (0.192 g).

R$_f$ = 0.36 (SiO_2, chloroform/methanol 20:1); **^{1}H NMR** (600 MHz, $CDCl_3$) δ [ppm]: 9.01 (s, 1H), 7.34-7.28 (m, 3H), 7.23 (d, J = 7.5 Hz, 1H), 7.02-6.97 (m, 3H), 6.96-6.91 (m, 2H), 4.30 (s, 4H), 4.04 (d, J = 12.1 Hz, 1H), 3.97 (s, 3H), 3.82 (s$_b$, 2H), 3.35 (d, J = 12.1 Hz, 1H), 3.27 (dd, J = 10.3, 5.0 Hz, 1H), 2.96 (t, J = 7.3 Hz, 1H), 2.39-2.32 (m, 1H), 2.30-2.20 (m, 1H), 1.96-1.90 (m, 1H), 1.78-1.73 (m, 1H), 1.72-1.68 (m, 1H); **^{13}C NMR** (151 MHz, $CDCl_3$) δ [ppm]: 174.9, 157.3, 143.3, 143.1, 141.4, 141.2, 121.0, 133.4, 131.0, 130.65, 130.57, 130.2, 126.5, 126.1, 122.9, 121.7, 118.6, 116.9, 112.2, 66.5, 64.52, 64.48, 55.8, 55.6, 54.0, 30.6, 24.0; **IR (ATR)** [cm^{-1}]: 3335, 2935, 2874, 1662, 1613, 1581, 1506, 1458, 1387, 1302, 1279, 1245, 1228, 1126, 1067, 1041, 1026, 919, 890, 871, 793, 744; **LC-MS (DAD/ESI):** t_R = 5.02 min, calcd for. $C_{27}H_{28}ClN_3O_4$ (m/z) $[M+H]^+$ 494.18; found $[M+H]^+$ 494.14; **HRMS (ESI):** calcd for $C_{27}H_{28}ClN_3O_4$ [m/z] $[M+H]^+$ 494.1841; found, $[M+H]^+$ 494.1847

1-((2'-chloro-3'-(2,3-dihydrobenzo[b][1,4]dioxin-6-yl)-3-methoxy-[1,1'-biphenyl]-4-yl)methyl)-N-(2-hydroxyethyl)pyrrolidine-2-carboxamide (1f)

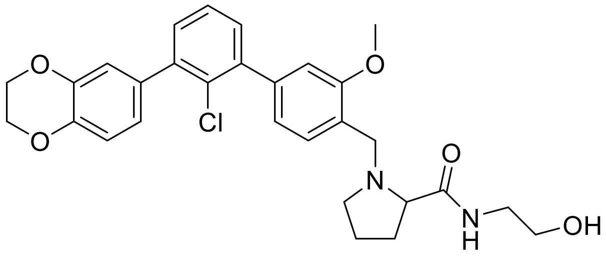

Reagent used: Ester **1** (0.200 g, 0.40 mmol), ethanolamine (0.098 ml, 1.60 mmol), K_2CO_3 (0.055 g, 0.40 mmol) methanol (3 ml). Crude product was purified on column chromatography (SiO_2, ethyl acetate/methanol 2:1) giving final product **1f** as a colorless solid with 81% yield (0.169 g).

R$_f$ = 0.65 (SiO_2, ethyl acetate/methanol 1:1); **^{1}H NMR** (600 MHz, $CDCl_3$) δ [ppm]: 8.16 (t, J = 6.0 Hz, 1H), 7.35-7.24 (m, 4H), 7.01-6.94 (m, 3H), 6.95-6.89 (m, 2H), 4.28 (s, 4H), 3.98 (d, J = 12.3 Hz, 1H), 3.88 (s, 3H), 3,73 (bs, 1H), 3.66 (t, J = 5.2 Hz, 2H), 3.49 (d, J = 12.3 Hz, 1H), 3.46-3.31 (m, 2H), 3.26-3.20 (m, 1H), 3.02 (t, J = 7.5 Hz, 1H), 2.46-2.39 (m, 1H), 2.29-2.19 (m, 1H), 1.94-1.86 (m, 1H), 1.79-1.63 (m, 2H); **^{13}C NMR** (151 MHz, $CDCl_3$) δ [ppm]: 176.6, 157.2, 143.2, 143.0, 141.3, 141.1, 140.9, 133.3, 130.9, 130.7, 130.6, 130.1, 126.4, 125.9, 122.8, 121.7, 118.6, 116.9, 112.3, 67.0, 64.5, 64.4, 62.8, 55.8, 54.9, 54.3, 42.6, 30.8, 23.9; **IR (ATR)** [cm^{-1}]: 3543, 3355, 2930, 1882, 1667, 1532, 1510, 1074, 860, 785; **LC-MS (DAD/ESI):** t_R = 5.52 min, calcd for $C_{29}H_{31}ClN_2O_5$ (m/z) $[M+H]^+$

523.20; found [M+H]$^+$ 523.25; **HRMS (ESI):** calcd for $C_{29}H_{31}ClN_2O_5$ [m/z] [M+H]$^+$ 523.1994; found, [M+H]$^+$ 523.2000

1-((2'-chloro-3'-(2,3-dihydrobenzo[b][1,4]dioxin-6-yl)-3-methoxy-[1,1'-biphenyl]-4-yl)methyl)-N-(1,3-dihydroxypropan-2-yl)pyrrolidine-2-carboxamide (**1g**)

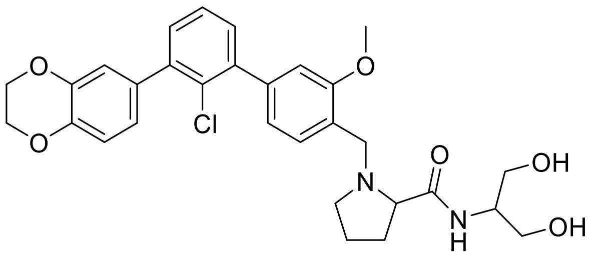

Reagent used: Ester **1** (0.200 g, 0,41 mmol), serinol (0.79 ml, 4.1 mmol), K_2CO_3 (0.084 g, 0.61 mmol) methanol (10 ml). Crude product was purified on column chromatography (SiO_2, ethyl acetate/methanol 10:1) giving final product **1g** as white solid with 61% yield (0.136 g).

R$_f$ = 0.60 (SiO_2, ethyl acetate/methanol 4:1); **^{1}H NMR** (600 MHz, $CDCl_3$) δ [ppm]: 8.11 (s, 1H), 7.32-7.28 (m, 4H), 7.01-6.99 (m, 3H), 6.95-6.91 (m, 2H), 4.30 (s, 4H), 3.88 (d, J = 14.6 Hz, 1H), 3.87 (s, 3H), 3.76-3.72 (m, 3H), 3.69-3.66 (m, 3H), 3.38 (bs, 2H), 3.31-3.28 (m, 1H), 3.12 (t, J = 7.4 Hz, 1H), 2.53-2.48 (m, 1H), 2.28-2.23 (m, 1H), 2.06 (bs, 1H), 1.93-1.89 (m, 1H), 1.79-1.70 (m, 2H); **^{13}C NMR** (151 MHz, $CDCl_3$) δ [ppm]: 176.4, 157.4, 143.3, 141.3, 141.2, 141.1, 133.4, 131.0, 130.8, 130.7, 130.2, 126.6, 125.9, 122.9, 121.8, 118.7, 117.0, 112.5, 67.3, 64.6, 64.5, 63.1, 63.0, 55.9, 54.8, 54.5, 53.5, 31.1, 24.1; **IR (ATR)** [cm^{-1}]: 3308, 2932, 2875, 1643, 1581, 1507, 1457, 1386, 1302, 1280, 1245, 1228, 1126, 1067, 1042, 919, 891, 871, 793, 744; **LC-MS (DAD/ESI):** t_R = 4.90 min, calcd for. $C_{30}H_{33}ClN_2O_6$ (m/z) [M+H]$^+$ 553.21; found [M+H]$^+$ 553.22; **HRMS (ESI):** calcd for $C_{30}H_{33}ClN_2O_6$ [m/z] [M+H]$^+$ 553.2100; found, [M+H]$^+$ 553.2104

1-((2'-chloro-3'-(2,3-dihydrobenzo[b][1,4]dioxin-6-yl)-3-methoxy-[1,1'-biphenyl]-4-yl)methyl)pyrrolidine-3-carbohydrazide (**2c**)

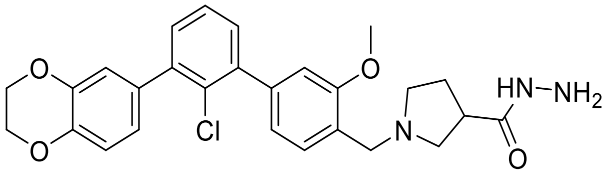

Reagent used: Ester **2** (0.200 g, 0,41 mmol), hydrazine monohydrate (0.79 ml, 4.1 mmol), methanol (8 ml). Crude product was purified on column chromatography (SiO_2, dichloromethane/methanol 10:1) giving final product **2c** as brown solid with 7% yield (0.015 g).

R$_f$ = 0.16 (SiO_2, dichloromethane/methanol 1:1); **^{1}H NMR** (600 MHz, DMSO-d_6) δ [ppm]: 7.44-7.40 (m, 1H), 7.38-7.33 (m, 3H), 7.01-6.97 (m, 2H), 6.93-6.88 (m, 3H), 4.28 (s, 4H), 3.80 (s, 3H), 3.59 (s, 2H), 2.93-2.75 (m, 2H), 2.75-2.66 (m, 1H), 2.47-2.36 (m, 2H), 2.02-1.92 (m, 2H); **^{13}C NMR** (151 MHz, DMSO-d_6) δ [ppm]: 173.3, 156.5, 143.1, 142.9, 141.0, 140.5, 139.2, 132.5, 130.5, 130.3, 129.9, 129.3, 127.0, 126.2, 122.4, 121.1, 118.0, 116.7, 111.9, 64.1, 57.3, 55.4, 53.8, 52.8, 40.8, 27.7; **IR (ATR)** [cm^{-1}]: 3320(broad), 2923, 2852, 1661, 1582, 1507, 1458, 1388, 1303, 1280, 1246, 1229, 1068,

794; **LC-MS (DAD/ESI):** t_R = 5.47 min, calcd for. $C_{27}H_{28}ClN_3O_4$ (m/z) $[M+H]^+$ 494.18; found $[M+H]^+$ 493.28; **HRMS (ESI):** calcd for $C_{27}H_{28}ClN_3O_4$ [m/z] $[M+H]^+$ 494.1841; found, $[M+H]^+$ 494.1847

1-((2'-chloro-3'-(2,3-dihydrobenzo[b][1,4]dioxin-6-yl)-3-methoxy-[1,1'-biphenyl]-4-yl)methyl)-N-(2-hydroxyethyl)pyrrolidine-3-carboxamide (**2f**)

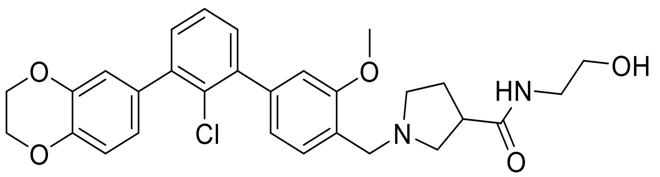

Reagent used: Ester **2** (0.200 g, 0,41 mmol), ethanolamine (0.25 ml, 4.1 mmol), K_2CO_3 (0.084 g, 0.61 mmol) ethanol (10 ml). Crude product was purified on column chromatography (SiO_2, ethyl acetate/methanol/7M ammonia in methanol 90:45:0.5) giving final product **2f** as white solid with 86% yield (0.182 g).

R_f = 0.22 (SiO_2, ethyl acetate/methanol 1:1); **^{1}H NMR** (600 MHz, $CDCl_3$) δ [ppm]: 7.81 (t, J = 5.5 Hz, 1H), 7.36 (d, J = 7.6 Hz, 1H), 7.34-7.27 (m, 3H), 7.02 (dd, J = 7.6, 1.6 Hz, 1H), 7.01-6.87 (m, 2H), 6.96-6.89 (m, 2H), 5.19 (s, 1H), 4.30 (s, 4H), 3.96-3.85 (m, 2H), 3.85 (m, 3H), 3.66 (t, J = 5.0 Hz, 2H), 3.41-3.29 (m, 2H), 3.12-2.97 (m, 3H), 2.89 (dd, J = 10.0, 7.2 Hz, 1H), 2.74-2.70 (m, 1H), 2.26-2.20 (m, 1H), 2.09-2.05 (m, 1H); **^{13}C NMR** (151 MHz, $CDCl_3$) δ [ppm]: 176.6, 157.3, 143.3, 143.1, 141.5, 141.2, 141.2, 133.3, 131.0, 130.9, 130.7, 130.2, 126.5, 123.3, 122.9, 121.9, 118.6, 117.0, 112.4, 64.5, 64.5, 62.3, 55.7, 52.6, 43.9, 42.8, 28.9; **IR (ATR)** [cm^{-1}]: 3283, 2933, 2874, 1651, 1581, 1507, 1459, 1392, 1303, 1280, 1247, 1230, 1068, 872, 796, 745; **LC-MS (DAD/ESI):** t_R = 5.29 min, calcd for. $C_{29}H_{31}ClN_2O_5$ (m/z) $[M+H]^+$ 523.20; found $[M+H]^+$ 523.32; **HRMS (ESI):** calcd for $C_{29}H_{31}ClN_2O_5$ [m/z] $[M+H]^+$ 523.2001; found, $[M+H]^+$ 523.1994

1-((2'-chloro-3'-(2,3-dihydrobenzo[b][1,4]dioxin-6-yl)-3-methoxy-[1,1'-biphenyl]-4-yl)methyl)-N-(1,3-dihydroxypropan-2-yl)pyrrolidine-3-carboxamide (**2g**)

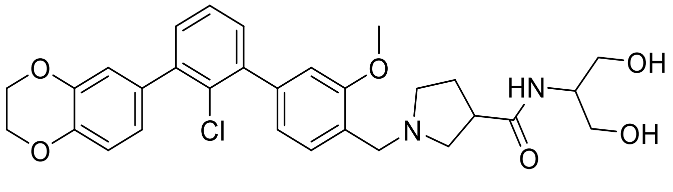

Reagent used: Ester **2** (0.200 g, 0,41 mmol), serinol (0.369 g, 4.1 mmol), K_2CO_3 (0.084 g, 0.61 mmol) methanol (10 ml). Crude product was purified on column chromatography (SiO_2, chloroform/7M ammonia in methanol 25:2) giving final product **2g** as yellowish solid with 33% yield (0.073 g).

R_f = 0.57 (SiO_2, chloroform/7M ammonia solution in methanol 5:1); **^{1}H NMR** (600 MHz, $CDCl_3$) δ [ppm]: 7.72 (d, J = 7.3 Hz, 1H), 7.37 (d, J = 7.6 Hz, 1H), 7.33-7.27 (m, 3H), 7.01 (dd, J = 7.7, 1.2 Hz, 1H), 6.99-6.98 (m, 2H), 6.94-6.91 (m, 2H), 4.30 (s, 4H), 3.84 (bs, 6H), 3.81 (s, 2H), 3.76-3.65 (m, 4H), 3.05-2.98 (m, 2H), 2.97-2.91 (m, 1H), 2.75 (t, J = 8.5 Hz, 1H), 2.62 (q, J = 8.7 Hz, 1H), 2.24-

2.16 (m, 1H), 2.07-2.01 (m, 1H); **^{13}C NMR** (151 MHz, $CDCl_3$) δ [ppm]: 177.0, 157.2, 143.3, 143.1, 141.4, 141.2, 141.1, 133.4, 130.7, 130.6, 130.2, 126.5, 124.6, 122.9, 121.9, 118.7, 117.0, 112.4, 64.6, 64.5, 63.2, 57.2, 55.8, 53.1, 52.8, 44.1, 29.0; **IR (ATR)** [cm^{-1}]: 3293(broad), 2933, 2875, 1648, 1581, 1507, 1459, 1387, 1303, 1280, 1247, 1229, 1068, 1043, 795; **LC-MS (DAD/ESI):** t_R = 5.15 min, calcd for. $C_{30}H_{33}ClN_2O_6$ (m/z) $[M+H]^+$ 553.21; found $[M+H]^+$ 553.29; **HRMS (ESI):** calcd for $C_{30}H_{33}ClN_2O_6$ [m/z] $[M+H]^+$ 553.2100; found, $[M+H]^+$ 553.2102

1-((2'-chloro-3'-(2,3-dihydrobenzo[b][1,4]dioxin-6-yl)-3-methoxy-[1,1'-biphenyl]-4-yl)methyl)piperidine-2-carbohydrazide (3c)

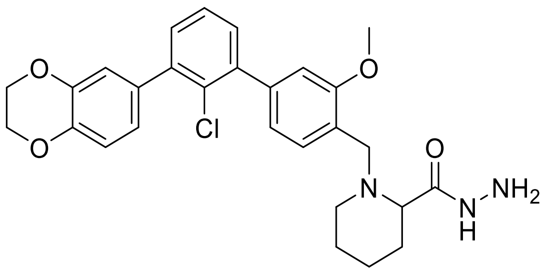

Reagent used: Ester **3** (0.234 g, 0.46 mmol), hydrazine monohydrate (1.0 ml, 18.4 mmol), methanol (6 ml). Crude product was purified on column chromatography (SiO_2, chloroform/methanol 10:1) giving final product **3c** as white solid with 20% yield (0.047 g).

R_f = 0.82 (SiO_2, chloroform/methanol 5:1); **^{1}H NMR** (600 MHz, $CDCl_3$) δ [ppm]: 8.58 (bs, 1H), 7.35-7.27 (m, 3H), 7.23 (d, J = 7.4 Hz, 1H), 7.03-6.97 (m, 3H), 6.95-6.90 (m, 2H), 4.30 (s, 4H), 3.95 (s, 3H), 3.94 (d, J = 12.9 Hz, 1H), 3.85 (bs, 2H), 3.01 (d, J = 13.0 Hz, 1H), 3.00-2.96 (m, 1H), 2.87 (m, 1H), 2.11-2.05 (m, 1H), 1.97-1.88 (m, 1H), 1.77-1.69 (m, 1H), 1.64-1.56 (m, 1H), 1.55-1.46 (m, 1H), 1.45-1.35 (m, 1H), 1.33-1.24 (m, 1H); **^{13}C NMR** (151 MHz, DMSO-d_6) δ [ppm]:172.6, 156.7, 143.1, 142.8, 141.0, 140.5, 139.1, 132.5, 130.4, 130.3, 129.9, 129.3, 126.9, 125.7, 122.3, 121.2, 118.0, 116.7, 111.9, 66.1, 64.1, 55.5, 53.5, 51.4, 30.0, 24.8, 23.1; **IR (ATR)** [cm^{-1}]: 3325, 2935, 1732, 1668, 1612, 1582, 1506, 1457, 1383, 1302, 1279, 1244, 1228, 1067, 1042, 1026, 890, 868, 793, 745; **LC-MS (DAD/ESI):** t_R = 5.27 min, calcd for. $C_{28}H_{30}ClN_3O_4$ (m/z) $[M+H]^+$ 508.20; found $[M+H]^+$ 508.34; **HRMS (ESI):** calcd for $C_{28}H_{30}ClN_3O_4$ [m/z] $[M+H]^+$ 508.1998; found, $[M+H]^+$ 508.1996

1-((2'-chloro-3'-(2,3-dihydrobenzo[b][1,4]dioxin-6-yl)-3-methoxy-[1,1'-biphenyl]-4-yl)methyl)-N-(2-hydroxyethyl)piperidine-2-carboxamide (3f)

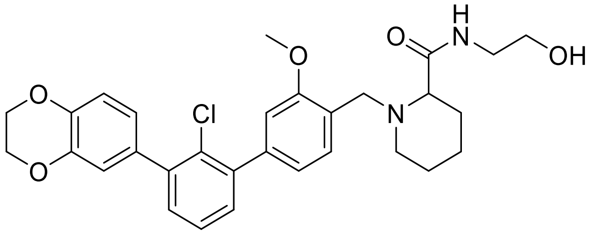

Reagent used: Ester **3** (0.200 g, 0.39 mmol),), ethanolamine (0.25 ml, 3.9 mmol), K_2CO_3 (0.082 g, 0.59 mmol) methanol (6 ml). Crude product was purified on column chromatography (SiO_2, chloroform/methanol 10:1) giving final product **3f** as white solid with 39% yield (0.083 g).

R_f = 0.78, (SiO_2, chloroform/methanol 5:1); **^{1}H NMR** (600 MHz, $CDCl_3$) δ [ppm]: 7.70 (s, 1H), 7.34-7.28 (m, 4H), 7.01-6.99 (m, 3H), 6.95-6.91 (m, 2H), 4.30 (s, 4H), 3.96 (d, J = 13.3 Hz, 1H), 3.87 (s, 3H), 3.73-3.69 (m, 2H), 3.48-3.45 (m, 2H), 3.18-3.16 (m, 1H), 2.98-2.96 (m, 1H), 2.86-2.84 (m, 1H), 2.86 (bs, 1H), 2.06-2.01 (m, 1H), 1.76-1.74 (m, 1H), 1.63-1.61 (m, 1H), 1.54-1.52 (m, 1H), 1.45-1.42 (m, 1H), 1.32-1.30 (m, 1H), 1.25-1.23 (m, 1H); **^{13}C NMR** (151 MHz, $CDCl_3$) δ [ppm]: 171.3, 157.4, 143.3, 143.1, 141.4, 141.2, 133.4, 131.0, 130.7, 130.6, 130.2, 126.5, 122.9, 121.8, 118.7, 117.0, 112.6, 64.6, 64.5, 63.5, 63.1, 55.9, 52.1, 42.7, 39.0, 25.0, 23.4, 21.0; **IR (ATR)** [cm^{-1}]: 3345, 2934, 1736, 1650, 1507, 1457, 1380, 1302, 1279, 1244, 1227, 1066, 1043, 891, 869, 795, 744; **LC-MS (DAD/ESI):** t_R = 5.13 min, calcd for. $C_{30}H_{33}ClN_2O_5$ (m/z) $[M+H]^+$ 537.21; found $[M+H]^+$ 537.14; **HRMS (ESI):** calcd for $C_{30}H_{33}ClN_2O_5$ [m/z] $[M+H]^+$ 537.2151; found, $[M+H]^+$ 537.2151

1-((2'-chloro-3'-(2,3-dihydrobenzo[b][1,4]dioxin-6-yl)-3-methoxy-[1,1'-biphenyl]-4-yl)methyl)-N-(1,3-dihydroxypropan-2-yl)piperidine-2-carboxamide (3g)

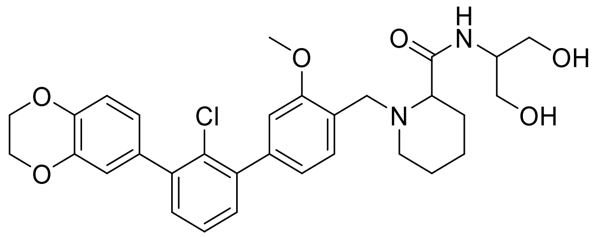

Reagent used: Ester **3** (0.200 g, 0.39 mmol), serinol (0.35 ml, 3.9 mmol), K_2CO_3 (0.082 g, 0.59 mmol) methanol (6 ml). Crude product was purified on column chromatography (SiO_2, chloroform/methanol 10:1) giving final product **3g** as white solid with 14% yield (0.032 g).

R_f = 0.71, (SiO_2, chloroform/methanol 5:1); **^{1}H NMR** (600 MHz, DMSO-d_6) δ [ppm]: 7.44 (m, 2H), 7.36 (m, 3H), 7.02 (s, 1H), 6.98 (d, J = 7.6 Hz, 1H), 6.95-6.92 (m, 2H), 6.89 (dd, J = 8.3, 2.0 Hz, 1H), 4.76 (t, J = 5.4 Hz, 1H), 4.67 (t, J = 5.3 Hz, 1H), 4.28 (s, 4H), 4.67 (s, 3H), 3.80-3.73 (m, 1H), 3.68 (d, J = 13.6 Hz, 1H), 3.53-3.48 (m, 1H), 3.46-3.41 (m, 4H), 3.29 (d, J = 13.5 Hz, 1H), 2.79-2.73 (m, 2H) 2.00-1.92 (m, 1H), 1.82-1.76 (m, 1H), 1.69-1.62 (m, 1H), 1.56-1.50 (m, 2H), 1.38-1.34 (m, 1H); **^{13}C NMR** (151 MHz, DMSO-d_6) δ [ppm]: 173.2, 157.1, 143.1, 142.9, 141.1, 140.5, 139.4, 132.6, 130.5, 129.9, 127.0, 125.4, 122.5, 121.3, 118.1, 116.8, 114.3, 112.1, 109.5, 67.7, 64.2, 64.2, 59.8, 55.6, 53.6, 52.0, 51.1, 29.1, 24.5, 23.2; **IR (ATR)** [cm^{-1}]: 3351, 2933, 1649, 1582, 1507, 1457, 1385, 1302, 1279, 1246, 1227, 891, 869, 793, 745; **LC-MS (DAD/ESI):** t_R = 5.82, calcd for. $C_{31}H_{35}ClN_2O_6$ (m/z) $[M+H]^+$ 567.23; found $[M+H]^+$ 567.31; **HRMS (ESI):** calcd for $C_{31}H_{35}ClN_2O_6$ [m/z] $[M+H]^+$ 567.2257; found, $[M+H]^+$ 567.2257

1-((2'-chloro-3'-(2,3-dihydrobenzo[b][1,4]dioxin-6-yl)-3-methoxy-[1,1'-biphenyl]-4-yl)methyl)-N-hydroxypiperidine-3-carboxamide (4b)

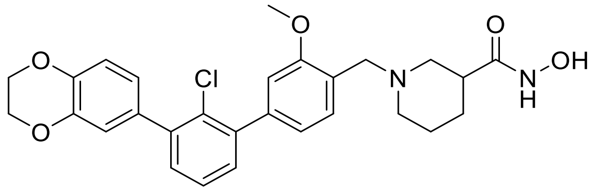

Reagent used: Ester **4** (0.174 g, 0,33 mmol), hydroxylamine 50% wt. in water (0.82 ml, 13.3 mmol), ethanol (6 ml). Crude product was purified on column chromatography (SiO_2, chloroform/methanol 10:1) giving final product **4b** as yellowish solid with 21% yield (0.036 g).

R_f = 0.73, (SiO_2, chloroform/methanol 5:1); **^{1}H NMR** (600 MHz, DMSO-d_6) δ [ppm]: 10.46 (s, 1H), 8.70 (s, 1H), 7.43 (t, J = 7.5 Hz, 1H), 7.38 (dd, J = 7.6, 1.8 Hz, 1H), 7.02 (s, 1H), 6.98 (dd, J = 7.7, 1.5 Hz, 1H), 6.95-6.91 (m, 2H), 6.89 (dd, J = 8.3, 2.0 Hz, 1H), 4.28 (s, 4H), 3.80 (s, 3H), 3.53-3.51 (m, 2H), 2.75-2.74 (m, 2H), 2.28-2.25 (m, 1H), 2.15-2.12 (m, 1H), 2.01-1.98 (m, 1H), 1.64-1.61 (m, 2H), 1.48-1.40 (m, 2H); **^{13}C NMR** (151 MHz, DMSO-d_6) δ [ppm]: 170.6, 157.0, 143.2, 142.9, 141.1, 140.5, 139.5, 132.6, 130.6, 130.5, 129.9, 129.7, 127.1, 125.3, 122.5, 121.2, 118.1, 116.9, 112.2, 64.23, 64.19, 55.9, 55.6, 53.3, 40,2, 40,1, 27.0, 24.3; **IR (ATR)** [cm^{-1}]: 2938, 1732, 1649, 1613, 1582, 1507, 1457, 1387, 1302, 1280, 1244, 1228, 1067, 1043, 891, 871, 793, 745; **LC-MS (DAD/ESI):** t_R = 5.37 min, calcd for. $C_{28}H_{29}ClN_2O_5$ (m/z) $[M+H]^+$ 509.18; found $[M+H]^+$ 509.16; **HRMS (ESI):** calcd for $C_{28}H_{29}ClN_2O_5$ [m/z] $[M+H]^+$ 509.1838; found, $[M+H]^+$ 509.1839

1-((2'-chloro-3'-(2,3-dihydrobenzo[b][1,4]dioxin-6-yl)-3-methoxy-[1,1'-biphenyl]-4-yl)methyl)piperidine-3-carbohydrazide (4c)

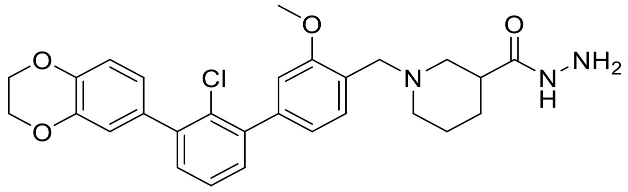

Reagent used: Ester **4** (0.220 g, 0.42 mmol), hydrazine monohydrate (0.9 ml, 16.9 mmol), ethanol (6 ml). Crude product was purified on column chromatography (SiO_2, chloroform/methanol 10:1) giving final product **4c** as white solid with 52% yield (0.112 g).

R_f = 0.50, (SiO_2, chloroform/methanol 5:1); **^{1}H NMR** (600 MHz, DMSO-d_6) δ [ppm]: 9.03 (s, 1H), 7.43 (t, J = 7.5 Hz, 1H), 7.38 (dd, J = 7.6, 1.9 Hz, 1H), 7.35-7.32 (m, 2H), 7.02 (d, J = 1.5 Hz, 1H), 6.98 (dd, J = 7.6, 1.6 Hz, 1H), 6.95-6.92 (m, 2H), 6.89 (dd, J = 8.3, 2.1 Hz, 1H), 4.28 (s, 4H), 4.14 (bs, 2H), 3.80 (s, 3H), 3.51 (d, J = 13.9 Hz, 1H), 3.43 (d, J = 14.0 Hz, 1H), 2.78-2.70 (m, 2H), 2.36-2.31 (m, 1H), 2.12 (t, J = 10.5 Hz, 1H), 2.02 (t, J = 10.1 Hz, 1H), 1.67-1.58 (m, 2H), 1.49-1.35 (m, 2H); **^{13}C NMR** (151 MHz, DMSO-d_6) δ [ppm]: 173.0, 157.0, 143.1, 142.9, 141.1, 140.5, 139.3, 132.6, 130.5, 130.4, 129.9, 129.6, 127.1, 125.4, 122.5, 121.2, 118.1, 116.8, 112.1, 64.2, 64.2, 56.0, 56.0, 55.6, 53.4, 41.1, 27.1, 24.3; **IR (ATR)** [cm^{-1}]: 3272, 2935, 1731, 1612, 1582, 1506, 1457, 1385, 1302, 1280, 1245, 1227, 1067, 1042, 1028, 890, 870, 793, 745; **LC-MS (DAD/ESI):** t_R = 5.32 min, calcd for. $C_{28}H_{30}ClN_3O_4$ (m/z) $[M+H]^+$ 508.20; found $[M+H]^+$ 508.16; **HRMS (ESI):** calcd for $C_{28}H_{30}ClN_3O_4$ [m/z] $[M+H]^+$ 508.1998; found, $[M+H]^+$ 508.1998

1-((2'-chloro-3'-(2,3-dihydrobenzo[b][1,4]dioxin-6-yl)-3-methoxy-[1,1'-biphenyl]-4-yl)methyl)-N-hydroxypiperidine-4-carboxamide (5b)

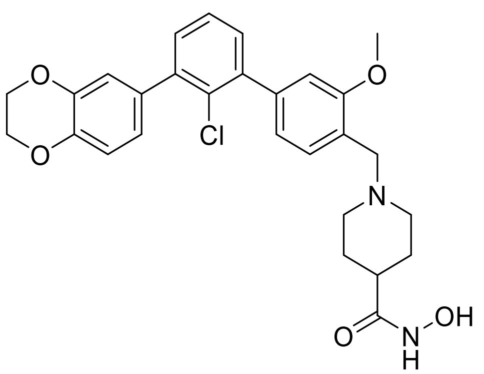

Reagent used: Ester **5** (0.200 g, 0.38 mmol), hydroxylamine 50% v/v in water (0.23 ml, 3.80 mmol), K_2CO_3 (0.080 g, 0.57 mmol), ethanol (6 ml). Crude product was purified on column chromatography (SiO_2, ethyl acetate/methanol 4:1) giving final product **5b** as yellowish solid with 23% yield (0.045 g).

R_f = 0.10 (SiO_2, ethyl acetate/methanol 1:1); **^{1}H NMR** (600 MHz, DMSO-d_6) δ [ppm]: 10.45 (s, 1H), 7.42 (t, J = 7.6 Hz, 1H), 7.37 (d, J = 8.0 Hz, 2H), 7.34 (dd, J = 7.4, 1.4 Hz, 1H), 7.01 (s_b, 1H), 6.99 (d, J = 7.6 Hz, 1H), 6.96-6.91 (m, 2H), 6.89 (dd, J = 8.3, 1.8 Hz, 1H), 4.28 (s, 4H), 2.79 (s, 3H), 3.47 (s, 2H), 2.93-2.83 (m, 2H), 2.04-1.82 (m, 3H), 1.69-1.55 (m, 4H); **^{13}C NMR** (151 MHz, DMSO-d_6) δ [ppm]: 171.6, 156.8, 143.1, 142.9, 141.1, 139.1, 132.5, 130.5, 130.3, 129.9, 129.1, 127.0, 125.6, 122.4, 121.2, 118.0, 116.7, 112.0, 64.14, 64.11, 55.6, 55.5, 52.9, 28.5, 21.5; **IR (ATR)** [cm^{-1}]: 3382, 2926, 1650, 1613, 1568, 1507, 1454, 1410, 1302, 1279, 1245, 1231, 1126, 1066, 1042, 1025, 939, 920, 891, 871, 795, 744; **LC-MS (DAD/ESI):** t_R = 5.24 min, calcd for. $C_{28}H_{29}ClN_2O_5$ (m/z) $[M+H]^+$ 509.18; found $[M+H]^+$ 509.16; **HRMS (ESI):** calcd for $C_{28}H_{29}ClN_2O_5$ [m/z] $[M+H]^+$ 509.1838; found, $[M+H]^+$ 509.1844

1-((2'-chloro-3'-(2,3-dihydrobenzo[b][1,4]dioxin-6-yl)-3-methoxy-[1,1'-biphenyl]-4-yl)methyl)piperidine-4-carbohydrazide (**5c**)

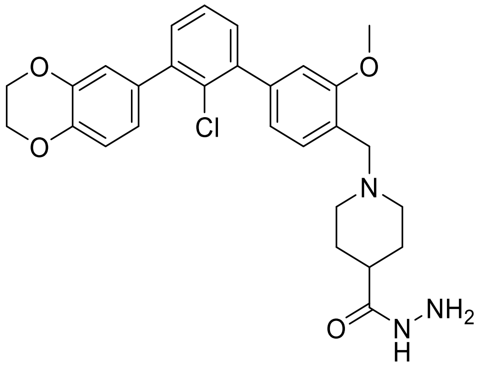

Reagent used: Ester **5** (0.100 g, 0.21 mmol), hydrazine hydrate (0.40 ml, 8.0 mmol), ethanol (6 ml). Crude product was purified on column chromatography (SiO_2, ethyl acetate/methanol 1:1) giving final product **5c** as colorless oil with 72% yield (0.073 g).

R_f = 0.25 (SiO_2, ethyl acetate/methanol 4:1); **^{1}H NMR** (600 MHz, DMSO-d_6) δ [ppm]: 8.94 (s, 2H), 7.43 (t, J = 7.5 Hz, 1H), 7.39-7.36 (m, 2H), 7.34 (dd, J = 7.4; 1.9 Hz, 1H), 7.02 (d, J = 1.5 Hz, 1H), 6.99 (dd, J = 7.7; 1.6 Hz, 1H); 6.95-6.92 (m, 2H); 6.89 (dd, J = 8.3; 2.0 Hz, 1H); 4.28 (s, 6H); 3.80 (s, 3H); 3.47 (s, 2H); 2.91-2.85 (m, 2H); 2.07-2.02 (m, 1H); 1.98-1.94 (m, 2H); 1.64-1.58 (m, 4H); **^{13}C NMR** (151 MHz, DMSO-d_6) δ [ppm]: 168.6, 156.8, 143.1, 142.9, 141.1, 140.5, 139.1, 132.5, 130.4, 130.3, 129.9, 127.0, 125.6, 122.4, 121.2, 118.0, 116.8, 112.0, 64.1, 64.1, 55.6, 55.5, 53.0, 40.3, 28.6; **IR (ATR)** [cm^{-1}]: 3279, 1613, 1582, 1507, 1455, 1379, 1302, 1280, 1245, 1228, 1067, 1042, 1027, 891, 871, 794, 744; **LC-MS (DAD/ESI):** t_R = 5.14 min, calcd for. $C_{28}H_{30}ClN_3O_4$

(m/z) $[M+H]^+$ 508.20; found $[M+H]^+$ 508.16; **HRMS (ESI):** calcd for $C_{28}H_{30}ClN_3O_4$ [m/z] $[M+H]^+$ 508.2000; found, $[M+H]^+$ 508.2003

1-((2'-chloro-3'-(2,3-dihydrobenzo[b][1,4]dioxin-6-yl)-3-methoxy-[1,1'-biphenyl]-4-yl)methyl)-N-(2-hydroxyethyl)piperidine-4-carboxamide (**5f**)

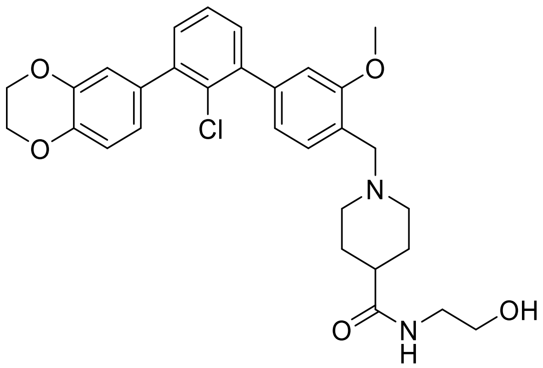

Reagent used: Ester **5** (0.200 g, 0.38 mmol), ethanolamine (0.30 ml, 3.80 mmol), K_2CO_3 (0.080 g, 0.57 mmol), ethanol (10 ml). Crude product was purified on column chromatography (SiO_2, ethyl acetate/methanol 1:1) giving final product **5f** as white solid with 83% yield (0.169 g).

$\mathbf{R_f}$ = 0.30 (SiO_2, ethyl acetate/methanol 1:1); **^{1}H NMR** (600 MHz, DMSO-d_6) δ [ppm]: 7.73 (t, J = 5.6 Hz, 1H), 7.43 (t, J = 7.5 Hz, 1H); 7.39-7.37 (m, 2H), 7.34 (dd, J = 7.4, 1.8 Hz, 1H), 7.02 (d, J = 1.4 Hz, 1H), 6.99 (dd, J = 7.7, 1.5 Hz, 1H), 6.94-6.93 (m, 2H), 6.89 (dd, J = 8.3, 2.0 Hz, 1H), 4.28 (s, 4H), 3.80 (s, 3H), 3.47 (s, 2H), 3.37 (t, J = 6.2 Hz, 2H), 3.09 (q, J = 6.1 Hz, 2H), 2.87-2.84 (m, 2H), 2.12-2.07 (m, 1H), 1.99-1.94 (m, 2H), 1.64-1.58 (m, 4H); **^{13}C NMR** (151 MHz, DMSO-d_6) δ [ppm]: 174.6, 156.8, 143.1, 142.9, 141.1, 140.5, 139.1, 132.5, 130.4, 130.3, 129.9, 129.1, 127.0, 125.7, 122.4, 121.2, 118.0, 116.7, 112.0, 64.13, 64.10, 59.9, 55.6, 55.5, 53.1, 42.0, 41.4, 28.7; **IR (ATR)** [cm^{-1}]: 3295, 2936, 1641, 1567, 1506, 1455, 1387, 1302, 1279, 1245, 1227, 1067, 1042, 1028, 871, 794, 744; **LC-MS (DAD/ESI):** t_R = 5.21 min, calcd for. $C_{30}H_{33}ClN_2O_5$ (m/z) $[M+H]^+$ 537.21; found $[M+H]^+$ 537.20; **HRMS (ESI):** calcd for $C_{30}H_{33}ClN_2O_5$ [m/z] $[M+H]^+$ 537.2151; found, $[M+H]^+$ 537.2157

1-((2'-chloro-3'-(2,3-dihydrobenzo[b][1,4]dioxin-6-yl)-3-methoxy-[1,1'-biphenyl]-4-yl)methyl)-N-(1,3-dihydroxypropan-2-yl)piperidine-4-carboxamide (**5g**)

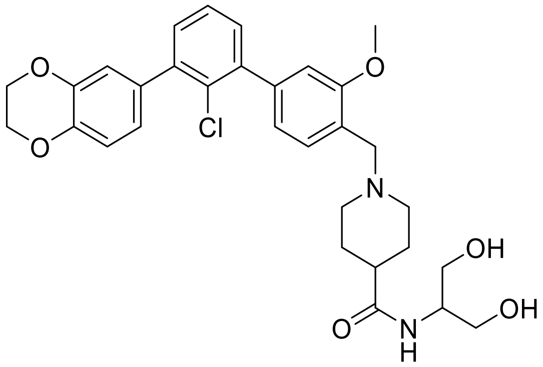

Reagent used: Ester **5** (0.200 g, 0.38 mmol), serinol (0.346 g, 3.80 mmol), K_2CO_3 (0.080 g, 0.57 mmol), ethanol (6 ml). Crude product was purified on column chromatography (SiO_2, ethyl acetate/methanol 4:1) giving final product **5g** as colorless oil with 33% yield (0.072 g).

$\mathbf{R_f}$ = 0.43 (SiO_2, ethyl acetate/methanol 1:1); **^{1}H NMR** (600 MHz, DMSO-d_6) δ [ppm]: 7.46 (d, J = 8.1 Hz, 1H), 7.41 (t, J = 7.5 Hz, 1H), 7.39-7.35 (m, 2H), 7.33 (dd, J = 7.4, 1.7 Hz, 1H), 7.01 (d, J = 1.1 Hz, 1H), 6.98 (d, J = 7.6 Hz, 1H), 6.94-6.91 (m, 2H), 6.89 (dd, J = 8.3, 2.0 Hz, 1H), 4.27 (s, 4H), 3.79 (s, 3H), 3.71-3.66 (m, 1H), 3.47 (s, 2H), 3.40 (d, J = 5.6 Hz, 4H), 2.91-2.85 (m, 2H), 2.18-2.11 (m, 1H), 2.01-1.94 (m, 2H), 1.63-1.59 (m, 4H); **^{13}C NMR** (151 MHz, DMSO-d_6) δ [ppm]: 174.7, 156.9, 143.1, 142.9,

141.1, 140.5, 139.1, 132.6, 130.5, 130.4, 129.9, 129.2, 127.0, 125.7, 122.4, 121.2, 118.1, 116.8, 112.0, 64.2, 64.2, 60.3, 55.7, 55.5, 53.1, 52.7, 42.1, 28.8; **IR (ATR)** [cm^{-1}]: 3278, 2936, 1642, 1613, 1567, 1506, 1455, 1394, 1302, 1279, 1245, 1228, 1067, 1042, 871, 794, 744; **LC-MS (DAD/ESI):** t_R = 5.02 min, calcd for. $C_{31}H_{35}ClN_2O_6$ (m/z) $[M+H]^+$ 567.23; found $[M+H]^+$ 567.24; **HRMS (ESI):** calcd for $C_{31}H_{35}ClN_2O_6$ [m/z] $[M+H]^+$ 567.2256; found, $[M+H]^+$ 567.2263

General procedure for HATU coupling

Method A

Appropriate esters **1-5** (1 equiv.) and LiOH monohydrate (1.5 equiv) were placed with round bottom flask together with dioxane/water (2:1) mixture. The reaction was heated at 60°C 16 hours. After this time solvents were removed and the resulting solid dried in high vacuum. The dried solid was dissolved in anhydrous DMF in round bottom flask. To this mixture the HATU (1.5 equiv.) and DIPEA (2-3 equiv.) were added. The reaction was stirred at room temperature for 30 minute and then excess of the appropriate amine was added. The mixture was then stirred at room temperature 16 h. After reaction was complete the water was added and mixture was extracted with ethyl acetate. Organic layers were collected, dried over anhydrous $MgSO_4$, then filtrated and evaporated. Crude products were purified by column chromatography giving final compounds **1d**, **1e**, **1h**, **2d**, **5d**, **5e**, **5h** with 17-77% yield.

Method B

Appropriate acid **1a-5a** (1 equiv.) was placed in round bottom flask with anhydrous DMF. To the mixture HATU (1 equiv.) and DIPEA (5 equiv.) were added and the reaction was stirred at room temperature for 30 minute. After this time the excess of appropriate amine was added. The reaction was further stirred at room temperature for 16 h. After reaction was complete the water was added and mixture was extracted with ethyl acetate. Organic layers were collected, dried over anhydrous $MgSO_4$, then filtrated and evaporated. Crude products were purified by column chromatography giving final compounds **2b**, **2e**, **2h**, **3h**, **4d**, **4g**, **4f**, **4h** with 32-60% yield.

1-((2'-chloro-3'-(2,3-dihydrobenzo[b][1,4]dioxin-6-yl)-3-methoxy-[1,1'-biphenyl]-4-yl)methyl)-N',N'-dimethylpyrrolidine-2-carbohydrazide (**1d**)

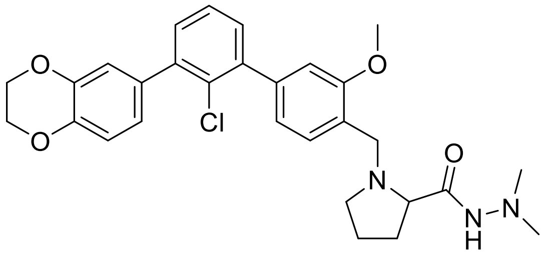

Method A. Reagent used: ester **1** (0.200 g, 0.41 mmol), LiOH monohydrate, (0.026 g, 0.61 mmol), HATU (0.232 g, 0.61 mmol), DIPEA, (0.14 ml, 0.81 mmol), 1,1-dimethylhydrazine (0.308 ml, 4.1 mmol). Crude product

was purified on column chromatography (SiO_2, ethyl acetate/methanol 4:1) giving final product **1d** as light green oil with 77% yield (0.163 g).

R_f = 0.41 (SiO_2, ethyl acetate/methanol 4:1); **^{1}H NMR** (600 MHz, $CDCl_3$) δ [ppm]: 8.33 (s_b, 1H), 7.26-7.18 (m, 4H), 6.94-6.90 (m, 3H), 6.87-6.82 (m, 2H), 4.22 (s, 4H), 3.86 (d, J = 12.5 Hz, 1H), 3.84 (s, 3H), 3.48 (d, J = 12.4 Hz, 1H), 3.19 (dd, J = 10.2, 5.0 Hz, 1H), 2.97 (t, J = 7.5 Hz, 1H), 2.48 (s, 6H), 2.37-2.34 (m, 1H), 2.20-2.16 (m, 1H), 1.87-1.83 (m, 1H), 1.70-1.63 (m, 1H), 1.63-1.57 (m, 1H); **^{13}C NMR** (151 MHz, $CDCl_3$) δ [ppm]: 172.0, 157.3, 143.2, 143.0, 141.3, 141.1, 141.0, 133.3, 130.9, 130.7, 130.6, 130.1, 126.4, 125.7, 122.8, 121.7, 118.6, 116.9, 112.4, 66.8, 64.5, 64.4, 55.6, 54.8, 54.5, 47.2, 30.8, 23.8; **LC-MS (DAD/ESI):** t_R = 5.55 min, calcd for. $C_{29}H_{32}ClN_3O_4$ (m/z) $[M+H]^+$ 522.12; found $[M+H]^+$ 522.19; **HRMS (ESI):** calcd for $C_{29}H_{32}ClN_3O_4$ [m/z] $[M+H]^+$ 522.2154; found, $[M+H]^+$ 522.2157

N-(2-aminoethyl)-1-((2'-chloro-3'-(2,3-dihydrobenzo[b][1,4]dioxin-6-yl)-3-methoxy-[1,1'-biphenyl]-4-yl)methyl)pyrrolidine-2-carboxamide (**1e**)

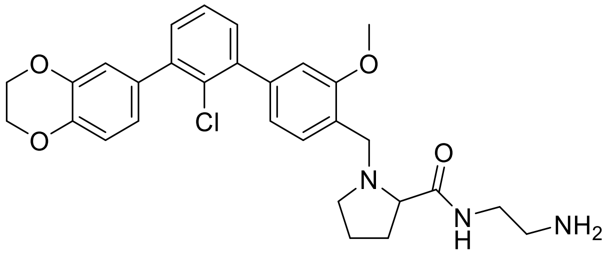

Method A. Reagent used: ester **1** (0.200 g, 0.41 mmol), LiOH monohydrate, (0.026 g, 0.61 mmol), HATU (0.232 g, 0.61 mmol), DIPEA, (0.14 ml, 0.81 mmol), N-Boc-ethylenediamine (0.64 ml, 4.10 mmol). Crude Boc-protected product was purified on column chromatography (SiO_2, chloroform/methanol 10:1). Resulting oil was then deprotected by the reaction with 1M HCl with dioxane at room temperature for 16 hours. Then the reaction mixture was poured into 1M NaOH and extracted with ethyl acetate. Organic layers were collected, dried over anhydrous $MgSO_4$, filtrated and evaporated giving final product **1e** with sufficient purity as white soli with 57% yield (0.121 g).

R_f = 0.33 (SiO_2, chloroform/methanol 20:1); **^{1}H NMR** (600 MHz, $CDCl_3$) δ [ppm]: 7.86 (t, J = 6.0 Hz, 1H), 7.27-7.18 (m, 4H), 6.94-6.91 (m, 3H), 6.89-6.83 (m, 2H), 4.22 (s, 4H), 3.90 (d, J = 12.4 Hz, 1H), 3.81 (s, 3H), 3.43 (d, J = 12.5 Hz, 1H), 3.29-3.23 (m, 1H), 3.22-3.17 (m, 1H), 3.15 (dd, J = 10.2, 5.0 Hz, 1H), 2.96 (t, J = 7.4 Hz, 1H), 2.72 (t, J = 6.2 Hz, 2H), 2.38-2.33 (m, 1H), 2.22-2.18 (m, 1H), 1.82-1.80 (m, 1H), 1.73-1.61 (m, 4H); **^{13}C NMR** (151 MHz, $CDCl_3$) δ [ppm]: 175.5, 157.2, 143.3, 143.1, 141.4, 141.2, 140.9, 133.4, 130.7, 130.6, 130.2, 126.5, 126.2, 122.9, 121.7, 118.6, 116.9, 112.3, 67.4, 64.53, 64.48, 55.8, 55.0, 54.4, 42.2, 42.0, 31.0, 24.0; **IR (ATR)** [cm^{-1}]: 3325, 2936, 2874, 1650, 1581, 1507, 1459, 1387, 1302, 1280, 1247, 1228, 1126, 1068, 794; **LC-MS (DAD/ESI): t_R** = 4.14 min, calcd for. $C_{29}H_{32}ClN_3O_4$ (m/z) $[M+H]^+$ 522.21; found $[M+H]^+$ 522.38; **HRMS (ESI):** calcd for $C_{29}H_{32}ClN_3O_4$ [m/z] $[M+H]^+$ 522.2154; found, $[M+H]^+$ 522.2163

1-((2'-chloro-3'-(2,3-dihydrobenzo[b][1,4]dioxin-6-yl)-3-methoxy-[1,1'-biphenyl]-4-yl)methyl)-N-(1,3-dihydroxy-2-(hydroxymethyl)propan-2-yl)pyrrolidine-2-carboxamide (1h)

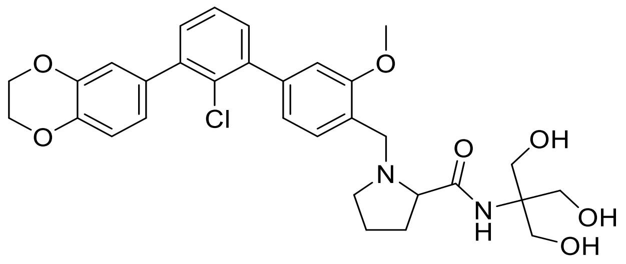

Method A. Reagent used: ester **1** (0.200 g, 0.41 mmol), LiOH monohydrate, (0.026 g, 0.61 mmol), HATU (0.232 g, 0.61 mmol), DIPEA, (0.14 ml, 0.81 mmol), tris(hydroxymethyl)aminomethane (0.491 g, 4.10 mmol). Crude product was purified on column chromatography (SiO_2, chloroform/methanol 10:1) giving final product **1h** as white solid with 47% yield (0.111 g).

R_f = 0.42 (SiO_2, chloroform/methanol 10:1); **^{1}H NMR** (600 MHz, $CDCl_3$) δ [ppm]: 8.17 (s, 1H), 7.30 (d, J = 7.6 Hz, 1H), 7.26-7.20 (m, 3H), 6.95-6.91 (m, 3H), 6.88-6.84 (m, 2H), 4.23 (s, 4H), 4.14 (s_b, 3H), 3.80 (d, J = 13.1 Hz, 1H), 3.78 (s, 3H), 3.65 (d, J = 13.0 Hz, 1H), 3.42 (s_b, 6H), 3.22 (dd, J = 10.2, 4.2 Hz, 1H), 3.16 (t, J = 7.3 Hz, 1H), 2.50-2.46 (m, 1H), 2.22-2.15 (m, 1H), 1.79-1.65 (m, 3H); **^{13}C NMR** (151 MHz, $CDCl_3$) δ [ppm]: 177.2, 157.3, 143.3, 143.1, 141.4, 141.2, 140.8, 133.4, 131.0, 130.7, 130.3, 126.6, 126.0, 122.9, 121.8, 118.7, 117.0, 112.4, 67.3, 64.6, 64.5, 62.7, 61.0, 55.7, 55.2, 53.7, 31.5, 24.3; **IR (ATR)** [cm^{-1}]: 3295, 2935, 2875, 1643, 1507, 1460, 1302, 1280, 1246, 1229, 1125, 1068, 1043, 1027, 785; **LC-MS (DAD/ESI): t_R** = 4.98 min, calcd for. $C_{31}H_{35}ClN_2O_7$ (m/z) $[M+H]^+$ 583.22; found $[M+H]^+$ 583.39; **HRMS (ESI):** calcd for $C_{31}H_{35}ClN_2O_7$ [m/z] $[M+H]^+$ 583.2205 found, $[M+H]^+$ 583.2211

1-((2'-chloro-3'-(2,3-dihydrobenzo[b][1,4]dioxin-6-yl)-3-methoxy-[1,1'-biphenyl]-4-yl)methyl)-N-hydroxypyrrolidine-3-carboxamide (2b)

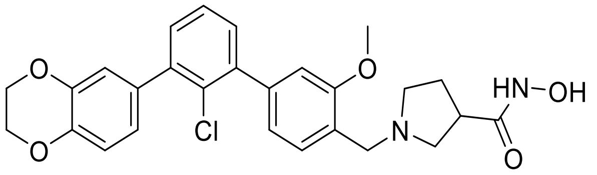

Method B. Reagent used: acid **2a** (0.200 g, 0.42 mmol), HATU (0.160 g, 0.42 mmol), DIPEA, (0.29 ml, 2.10 mmol), hydroxylamine 50% v/v water solution (1.00 ml, 16.2 mmol). Crude product was purified on column chromatography (SiO_2, chloroform/methanol/2M ammonia solution in chloroform 10:2:1) giving final product **2b** as white solid with 33% yield (0.069 g).

R_f = 0.20 (SiO_2, chloroform/2M ammonia in chloroform/methanol 20:2:1); **^{1}H NMR** (600 MHz, $CDCl_3$) δ [ppm]: 7.36-7.26 (m, 1H), 7.24 (d, J = 7.5 Hz, 1H), 7.02-6.96 (m, 3H), 6.96-6.91 (m, 2H), 4.31 (s, 4H), 3.92 (s, 3H), 3.87 (d, J = 12.5 Hz, 1H), 3.64 (d, H = 12.5 Hz, 1H), 3.03-2.94 (m, 2H), 2.93-2.87 (m, 1H), 2.47-2.42 (m, 1H), 2.36-2.29 (m, 1H), 2.22-2.13 (m, 1H), 2.02-1.93 (m, 1H); **^{13}C NMR** (151 MHz, $CDCl_3$) δ [ppm]: 174.0, 157.5, 143.3, 143.1, 141.5, 141.20, 141.16, 133.5, 131.5, 130.7, 130.32, 130.27, 126.5, 122.9, 121.7, 118.7, 117.0, 112.3, 64.6, 64.5, 56.6, 55.8, 54.1, 51.2, 42.3, 28.9; **IR (ATR)** [cm^{-1}]: 3188(broad), 2929, 2873, 1650, 1613, 1582, 1507, 1459, 1387, 1302, 1280,

1246, 1228, 1126, 1068, 1043, 872, 794, 745; **LC-MS (DAD/ESI):** t_R = 5.34 min, calcd for. $C_{27}H_{27}ClN_2O_5$ (m/z) $[M+H]^+$ 495.17; found $[M+H]^+$ 493.20; **HRMS (ESI):** calcd for $C_{27}H_{27}ClN_2O_5$ [m/z] $[M+H]^+$ 495.1681; found, $[M+H]^+$ 495.1688

1-((2'-chloro-3'-(2,3-dihydrobenzo[b][1,4]dioxin-6-yl)-3-methoxy-[1,1'-biphenyl]-4-yl)methyl)-N',N'-dimethylpyrrolidine-3-carbohydrazide (2d)

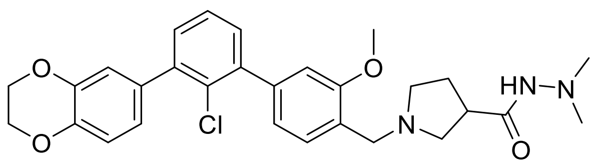

Method A. Reagent used: ester **2** (0.200 g, 0.41 mmol), LiOH monohydrate, (0.026 g, 0.61 mmol), HATU (0.232 g, 0.61 mmol), DIPEA, (0.14 ml, 0.81 mmol), 1,1-dimethylhydrazine (0.308 ml, 4.1 mmol). Crude product was purified by radial chromatography (SiO_2, dichloromethane/methanol 10:1) giving final product **2d** as colorless solid with 17% yield (0.035 g).

$\mathbf{R_f}$ = 0.54 (chloroform/ 7M ammonia in methanol 10:1); **^{1}H NMR** (600 MHz, DMSO-d_6, two rotamers are visible, the predominant rotameter signals are considered) δ [ppm]: 9.08 (bs, 1H), 7.53 (t, J = 8.4 Hz, 1H), 7.45 (t, J = 7.45 Hz, 1H), 7.39-7.37 (m, 2H), 7.17 (d, J = 7.7 Hz, 1H), 7.10 (t, J = 7.2 Hz, 1H), 6.95-6.89 (m, 3H), 4.34-4.28 (m, 6H), 3.89 (d, J = 4.8 Hz, 3H), 3.65 (bs, 1H), 3.25-3.15 (m, 3H), 2.98 (bs, 1H), 2.46 (s, 6H), 2.29-1.98 (m, 2H); **^{13}C NMR** (151 MHz, DMSO-d_6) δ [ppm]: 173.8, 157.3, 143.2, 142.9, 140.6, 140.5, 132.4, 130.9, 130.7, 130.3, 129.8, 127.1, 122.4, 121.6, 118.1, 116.8, 115.4, 112.6, 64.2, 64.2, 55.9, 52.1, 51.7, 47.7, 46.4, 38.0, 23.4; **IR (ATR)** [cm^{-1}]: 3640, 3571, 3343, 1660, 1628, 1532, 1508, 1456, 1304, 1247, 1178, 1068, 834; **LC-MS (DAD/ESI):** t_R = 5.47 min, calcd for $C_{29}H_{32}ClN_3O_4$ (m/z) $[M+H]^+$ 522.21; found $[M+H]^+$ 522.32; **HRMS (ESI):** calcd for $C_{29}H_{32}ClN_3O_4$ [m/z] $[M+H]^+$ 522.2154; found, $[M+H]^+$ 522.2162

N-(2-aminoethyl)-1-((2'-chloro-3'-(2,3-dihydrobenzo[b][1,4]dioxin-6-yl)-3-methoxy-[1,1'-biphenyl]-4-yl)methyl)pyrrolidine-3-carboxamide (**2e**)

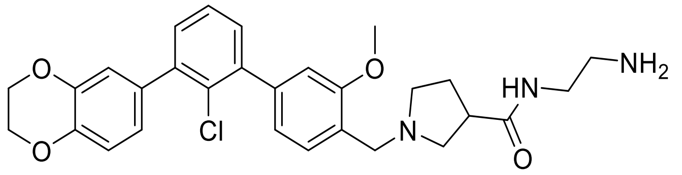

Method B. Reagent used: acid **2a** (0.200 g, 0.42 mmol) HATU (0.232 g, 0.61 mmol), DIPEA, (0.14 ml, 0.81 mmol), N-Boc-ethylenediamine (0.64 ml, 4.10 mmol). Crude Boc-protected product was purified on column chromatography (SiO_2, chloroform/methanol/2M ammonia solution in chloroform 100:10:4). Resulting oil was then deprotected by the reaction with 1M HCl with dioxane at room temperature for 16 hours. Then the reaction mixture was poured into 1M NaOH and extracted with ethyl acetate. Organic layers were collected, dried over anhydrous $MgSO_4$, filtrated and evaporated giving final product **2e** with sufficient purity as white soli with 41% yield (0.086 g).

R_f = 0.14 (SiO_2, 2M ammonia in chloroform/methanol 1:1); **^{1}H NMR** (600 MHz, $CDCl_3$) δ [ppm]: 7.58 (t, J = 5.9 Hz, 1H), 7.34 (d, J = 7.6 Hz, 1H), 7.33-7.27 (m, 3H), 7.03-6.95 (m, 3H), 6.95-6.90 (m, 2H), 4.30 (s, 4H), 3.85 (s, 3H), 3.81-3.73 (m, 2H), 3.36-3.21 (m, 2H), 3.01-2.94 (m, 2H), 2.93-2.86 (m, 1H), 2.90-2.81 (m, 4H), 2.63 (dd, J = 9.6, 6.8 Hz, 1H), 2.52 (q, J = 8.6 Hz, 1H), 2.25-2.15 (m, 1H), 2.05-1.95 (m, 1H); **^{13}C NMR** (151 MHz, $CDCl_3$) δ [ppm]: 177.1, 157.2, 143.3, 143.1, 141.4, 141.2, 140.8, 133.4, 131.0, 130.6, 130.3, 130.2, 126.5, 126.5, 125.5, 122.9, 121.7, 118.7, 117.0, 112.3, 64.6, 64.5, 57.5, 55.7, 53.2, 52.9, 44.2, 41.6, 41.6, 29.1; **IR (ATR)** [cm^{-1}]: 3272, 2929, 2873, 1650, 1582, 1507, 1459, 1386, 1303, 1280, 1247, 1228, 1068, 871, 795, 745; **LC-MS (DAD/ESI):** t_R = 5.03 min, calcd for. $C_{29}H_{32}ClN_3O_4$ (m/z) $[M+H]^+$ 522.12; found $[M+H]^+$ 522.25; **HRMS (ESI):** calcd for $C_{29}H_{32}ClN_3O_4$ [m/z] $[M+H]^+$ 522.2154; found, $[M+H]^+$ 522.2162

1-((2'-chloro-3'-(2,3-dihydrobenzo[b][1,4]dioxin-6-yl)-3-methoxy-[1,1'-biphenyl]-4-yl)methyl)-N-(1,3-dihydroxy-2-(hydroxymethyl)propan-2-yl)pyrrolidine-3-carboxamide (**2h**)

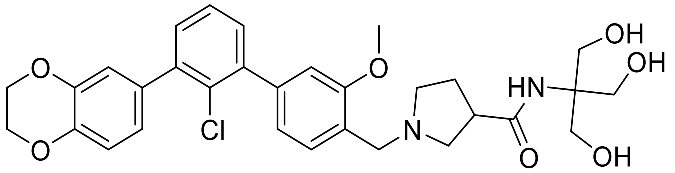

Method B. Reagent used: acid **2a** (0.200 g, 0.42 mmol), HATU (0.160 g, 0.42 mmol), DIPEA, (0.29 ml, 2.10 mmol), tris(hydroxymethyl)aminomethane (0.491 g, 4.10 mmol). Crude product was purified on column chromatography (SiO_2, chloroform/methanol/2M ammonia solution in chloroform 10:1.5:1) giving final product **2h** as yellowish solid with 48% yield (0.113 g).

R_f = 0.32 (SiO_2, chloroform/2M ammonia solution in chloroform/methanol 10:1:10); **^{1}H NMR** (600 MHz, $CDCl_3$) δ [ppm]: 7.73 (s, 1H), 7.40 (d, J = 7.6 Hz, 1H), 7.35-7.27 (m, 3H), 7.02 (dd, J = 7.6, 1.6 Hz, 1H), 7.01-6.98 (m, 2H), 6.96-6.90 (m, 2H), 4.31 (s, 4H), 3.85 (s, 3H), 3.81-3.72 (m, 2H), 3.63 (d, J = 2.2 Hz, 6H), 3.07-2.98 (m, 2H), 2.94-2.87 (m, 1H), 2.62 (dd, J = 9.7, 7.0 Hz, 1H), 2.52 (q, J = 8.3 Hz, 1H), 2.28-2.19 (m, 1H), 2.04-1.96 (m, 1H); **^{13}C NMR** (151 MHz, $CDCl_3$) δ [ppm]: 178.6, 157.1, 143.3, 143.1, 141.4, 141.2, 140.8, 133.4, 131.0, 130.7, 130.5, 130.3, 126.5, 125.4, 122.9, 121.9, 118.7, 117.0, 112.5, 64.6, 64.5, 63.5, 61.3, 57.8, 55.9, 53.1, 52.9, 44.7, 29.2; **IR (ATR)** [cm^{-1}]: 3307(broad), 2926, 1648, 1507, 1459, 1386, 1302, 1280, 1247, 1229, 1068, 1043, 871, 795, 745; **LC-MS (DAD/ESI):** t_R = 5.25 min, calcd for. $C_{31}H_{35}ClN_2O_7$ (m/z) $[M+H]^+$ 583.22; found $[M+H]^+$ 583.26; **HRMS (ESI):** calcd for $C_{31}H_{35}ClN_2O_7$ [m/z] $[M+H]^+$ 583.2205; found, $[M+H]^+$ 583.2210

1-((2'-chloro-3'-(2,3-dihydrobenzo[b][1,4]dioxin-6-yl)-3-methoxy-[1,1'-biphenyl]-4-yl)methyl)-N',N'-dimethylpiperidine-2-carbohydrazide (3d)

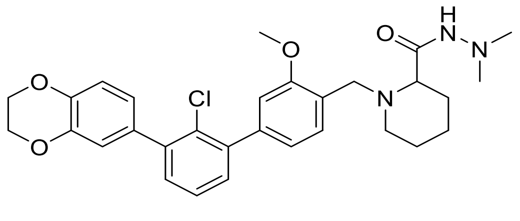

Method B. Reagent used: acid **4a** (0.180 g, 0.36 mmol), HATU (0.208 g, 0.55 mmol), DIPEA, (0.19 ml, 0.72 mmol), 1,1-dimethylhydrazine (0.28 ml, 3.6 mmol). Crude product was purified on column chromatography (SiO_2, chloroform/methanol 10:1) giving final product **3d** as light yellow solid with 40% yield (0.068 g).

R_f = 0.68, (SiO_2, chloroform/methanol 5:1); **^{1}H NMR** (600 MHz, $CDCl_3$) δ [ppm]: 8.02-7.98 (m, 1H), 7.34-7.29 (m, 4H), 7.03 (d, J = 7.4 Hz, 1H), 7.00-6.99 (m, 2H), 6.96-6.92 (m, 2H), 4.31 (s, 4H), 3.95 (d, J = 13.5 Hz, 1H), 3.89 (s, 3H), 3.24 (d, J = 13.3 Hz, 1H), 2.95-2.94 (m, 1H), 2.88-2.85 (m, 1H), 2.58 (s, 6H), 2.10-2.08 (m, 1H), 2.03-1.99 (m, 1H), 1.75-1.74 (m, 1H), 1.63-1.61 (m, 1H), 1.54-1.46 (m, 2H), 1.33-1.29 (m, 1H); **^{13}C NMR** (151 MHz, $CDCl_3$) δ [ppm]: 172.3, 157.4, 143.4, 143.2, 141.5, 141.3, 140.7, 133.4, 131.1, 130.7, 130.2, 130.0, 126.5, 125.4, 122.9, 121.7, 118.7, 117.0, 112.4, 68.1, 64.60, 64.6, 56.1, 55.6, 52.3, 47.2, 31.1, 25.1, 23.7; **LC-MS (DAD/ESI):** t_R = 6.44 min, calcd for. $C_{30}H_{34}ClN_3O_4$ (m/z) [M+H]+ 536.23; found [M+H]+ 536.35; **HRMS (ESI**): calcd for $C_{30}H_{34}ClN_3O_4$ [m/z] [M+H]+ 536.2311; found, [M+H]+ 536.2310

1-((2'-chloro-3'-(2,3-dihydrobenzo[b][1,4]dioxin-6-yl)-3-methoxy-[1,1'-biphenyl]-4-yl)methyl)-N-(1,3-dihydroxy-2-(hydroxymethyl)propan-2-yl)piperidine-2-carboxamide (3h)

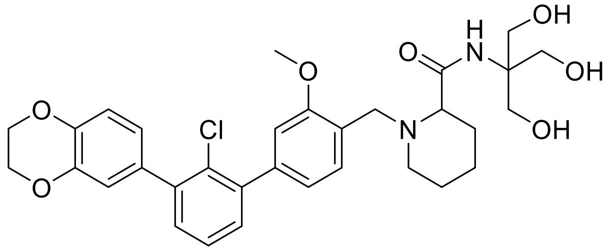

Method B. Reagent used: acid **3a** (0.200 g, 0.40 mmol), HATU (0.228 g, 0.60 mmol), DIPEA, (0.15 ml, 0.80 mmol), tris(hydroxymethyl)aminomethane (0.491 g, 4.05 mmol). Crude product was purified on column chromatography (SiO_2, chloroform/methanol/2M ammonia solution in chloroform 10:1.5:1) giving final product **3h** as yellowish solid with 50% yield (0.120 g).

R_f = 0.78, (SiO_2, chloroform/methanol 5:1); **^{1}H NMR** (600 MHz, $CDCl_3$) δ [ppm]: 7.87 (s, 1H), 7.50 (d, J = 7.7 Hz, 1H), 7.33-7.28 (m, 3H), 7.02 (dd, J = 7.7, 1.4 Hz, 1H), 6.99 (d, J = 1.6 Hz, 1H), 6.95 (m, 1H), 6.93-6.91 (m, 2H), 4.30 (s, 4H), 3.82 (s, 3H), 3.72 (d, J = 15.0 Hz, 1H), 3.62-3.53 (m, 7H), 3.02-2.98 (m, 1H), 2.94-2.93 (m, 1H), 2.19-2.13 (m, 1H), 2.05-2.01 (m, 1H), 1.76-1.63 (m, 1H), 1.65-1.49 (m, 3H), 1.39-1.29 (m, 1H); **^{13}C NMR** (151 MHz, $CDCl_3$) δ [ppm]: 176.8, 157.1, 143.3, 143.1, 141.5, 141.2, 140.3, 133.4, 131.0, 130.6, 130.3, 128.9, 126.5, 125.5, 122.9, 121.8, 118.7, 117.0, 112.1, 68.1, 64.6, 64.5, 63.1, 61.2, 55.6, 54.4, 52.0, 30.6, 24.7, 23.5; **IR (ATR)** [cm^{-1}]: 3317, 2935, 1734, 1649, 1505, 1457, 1302, 1279, 1243, 1228, 1067, 1043, 1026, 796, 744; **LC-MS (DAD/ESI):** t_R = 5.37 min, calcd for. $C_{32}H_{37}ClN_2O_7$ (m/z) $[M+H]^+$ 597.24; found $[M+H]^+$ 597.35; **HRMS (ESI):** calcd for $C_{32}H_{37}ClN_2O_7$ [m/z] $[M+H]^+$ 597.2362; found, $[M+H]^+$ 597.2361

1-((2'-chloro-3'-(2,3-dihydrobenzo[b][1,4]dioxin-6-yl)-3-methoxy-[1,1'-biphenyl]-4-yl)methyl)-N',N'-dimethylpiperidine-3-carbohydrazide (4d)

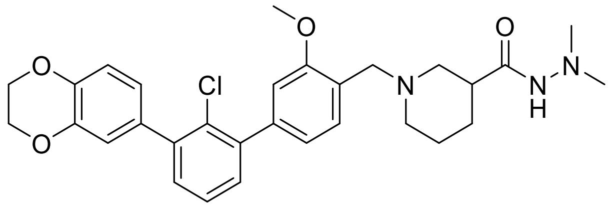

Method B. Reagent used: acid **4a** (0.154 g, 0.31 mmol), HATU (0.178 g, 0.47 mmol), DIPEA, (0.16 ml, 0.62 mmol), 1,1-dimethylhydrazine (0.24 ml, 3.1 mmol). Crude product was purified on column chromatography (SiO_2, chloroform/methanol 10:1) giving final product **4d** as light yellow solid with 32% yield (0.054 g).

R_f = 0.67, (SiO_2, chloroform/methanol 5:1); **^{1}H NMR** (600 MHz, $CDCl_3$) δ [ppm]: 9.12 (bs, 1H), 7.34-7.27 (m, 4H), 7.03-6.99 (m, 3H), 6.95-6.91 (m, 2H), 4.30 (s, 4H), 3.88 (s, 3H), 3.71-3.70 (m, 1H), 3.65 (d, J = 12.5 Hz, 1H), 3.51 (bs, 1H), 2.99 (bs, 2H), 2.55 (s_b, 1H) 2.48 (s, 6H), 2.32 (bs, 1H), 1.98 (bs, 1H), 1.74-1.68 (m, 1H), 1.63-1.57 (m, 2H); **^{13}C NMR** (151 MHz, $CDCl_3$) δ [ppm]: 172.8, 161.3, 157.6, 143.4, 143.1, 141.4, 141.3, 141.1, 133.4, 131.2, 130.7, 130.2, 126.5, 122.9, 121.6, 118.7, 117.0, 112.6, 67.0, 64.6, 57.1, 55.9, 54.4, 54.2, 53.4, 47.7, 41.0, 26.9, 22.6; **IR (ATR)** [cm^{-1}]: 2936, 1726, 1650, 1613, 1582, 1507, 1456, 1387, 1302, 1280, 1302, 1280, 1245, 1227, 1175, 1126, 1067, 1042, 1028, 941, 920, 891, 834, 796, 744; **LC-MS (DAD/ESI):** t_R = 5.50 min, calcd for. $C_{30}H_{34}ClN_3O_4$ (m/z) $[M+H]^+$ 536.23; found $[M+H]^+$ 536.36; **HRMS (ESI):** calcd for $C_{30}H_{34}ClN_3O_4$ [m/z] $[M+H]^+$ 536.2311; found, $[M+H]^+$ 536.2311

1-((2'-chloro-3'-(2,3-dihydrobenzo[b][1,4]dioxin-6-yl)-3-methoxy-[1,1'-biphenyl]-4-yl)methyl)-N-(2-hydroxyethyl)piperidine-3-carboxamide (4f)

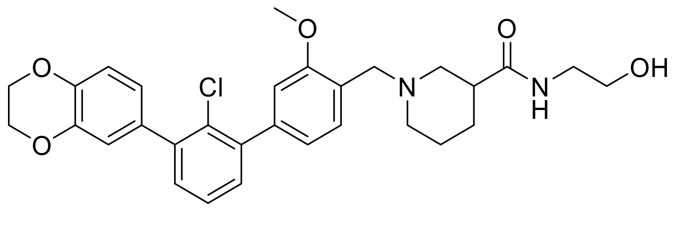

Method B. Reagent used: acid **4a** (0.200 g, 0.40 mmol), HATU (0.231 g, 0.61 mmol), DIPEA, (0.21 ml, 0.80 mmol), ethanolamine (0.25 ml, 4.1 mmol). Crude product was purified on column chromatography (SiO_2, chloroform/methanol 10:1) giving final product **4f** as light yellow solid with 54% yield (0.117 g).

R_f = 0.35 (SiO_2, chloroform/methanol 10:1); **^{1}H NMR** (600 MHz, $CDCl_3$) δ [ppm]: 7.94 (s, 1H), 7-32-7.21 (m, 4H), 7.11-6.98 (m, 2H), 6.91 (m, 1H), 6.86 (m, 2H), 4.24 (s, 4H), 4.10 (d, J = 4.10 Hz, 1H), 3.91 (s, 3H), 3.71-3.58 (m, 3H), 3.50-3.47 (m, 1H), 3.41-3.31 (m, 3H), 3.20-3.07 (m, 2H), 3.05-3.03 (m, 1H), 2.02-1.76 (m, 4H); **^{13}C NMR** (151 MHz, $CDCl_3$) δ [ppm]: 157.3, 144.2, 143.4, 143.2, 141.3, 140.5, 133.1, 131.9, 131.1, 130.7, 130.1, 126.7, 122.8, 122.7, 118.6, 117.0, 116.2, 112.5, 64.6, 61.6, 57.8, 55.9, 55.6, 54.6, 43.5, 42.5, 25.1, 20.2; **IR (ATR)** [cm^{-1}]: 3420, 2943, 1650, 1508, 1456, 1388, 1303, 1281, 1246, 1228, 1067, 828, 741; **LC-MS (DAD/ESI):** t_R = 5.46 min, calcd for.

$C_{30}H_{33}ClN_2O_5$ (m/z) $[M+H]^+$ 537.21; found $[M+H]^+$ 537.34; **HRMS (ESI):** calcd for $C_{30}H_{33}ClN_2O_5$ [m/z] $[M+H]^+$ 537.2151; found, $[M+H]^+$ 537.2151

1-((2'-chloro-3'-(2,3-dihydrobenzo[b][1,4]dioxin-6-yl)-3-methoxy-[1,1'-biphenyl]-4-yl)methyl)-N-(1,3-dihydroxypropan-2-yl)piperidine-3-carboxamide (4g)

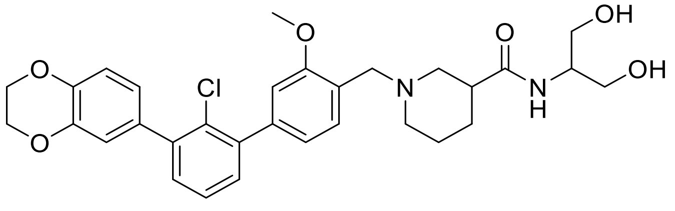

Method B. Reagent used: acid **4a** (0.154 g, 0.31 mmol), HATU (0.178 g, 0.47 mmol), DIPEA, (0.16 ml, 0.62 mmol), 1,1-dimethylhydrazine (0.24 ml, 3.1 mmol). Crude product was purified on column chromatography (SiO_2, chloroform/methanol 10:1) giving final product **4g** as light yellow solid with 32% yield (0.054 g).

R_f = 0.13 (SiO_2, chloroform/methanol 10:1); **^{1}H NMR** (600 MHz, $CDCl_3$) δ [ppm]: 8.79 (bs, 1H), 7.36-7.27 (m, 4H), 7.03 (d, J = 1.5 Hz, 1H), 7.02 (m, 1H), 6.99 (d, J = 1.4 Hz, 1H), 6.96-6.91 (m, 2H), 4.31 (s, 4H), 3.87 (s, 3H), 3.85-3.81 (m, 1H), 3.80-3.65 (m, 4H), 3.63 (d, J = 12.6 Hz, 1H), 3.55 (d, J = 12.5 Hz, 1H), 3.02-2.93 (m, 2H), 2.60 (bs, 1H), 2.44-2.29 (m, 2H), 1.92 (m, 1H), 1.81-1.72 (m, 1H), 1.64 (bs, 2H); **^{13}C NMR** (151 MHz, $CDCl_3$) δ [ppm]: 157.6, 143.3, 143.1, 141.4, 141.3, 141.3, 133.4, 131.3, 131.0, 130.8, 130.2, 126.6, 122.9, 121.9, 118.7, 117.0, 112.7, 77.2, 64.6, 64.5, 63.2, 63.1, 56.7, 54.9, 53.8, 53.6, 41.6, 26.9, 22.6; **IR (ATR)** [cm^{-1}]: 3290, 2936, 1640, 1568, 1507, 1456, 1386, 1302, 1280, 1245, 1228, 1067, 1042, 870, 794, 744; **LC-MS (DAD/ESI):** t_R = 5.28 min, calcd for. $C_{31}H_{35}ClN_2O_6$ (m/z) $[M+H]^+$ 567.23; found $[M+H]^+$ 567.31; **HRMS (ESI):** calcd for $C_{31}H_{35}ClN_2O_6$ [m/z] $[M+H]^+$ 567.2257; found, $[M+H]^+$ 567.2258

1-((2'-chloro-3'-(2,3-dihydrobenzo[b][1,4]dioxin-6-yl)-3-methoxy-[1,1'-biphenyl]-4-yl)methyl)-N-(1,3-dihydroxy-2-(hydroxymethyl)propan-2-yl)piperidine-3-carboxamide (4h)

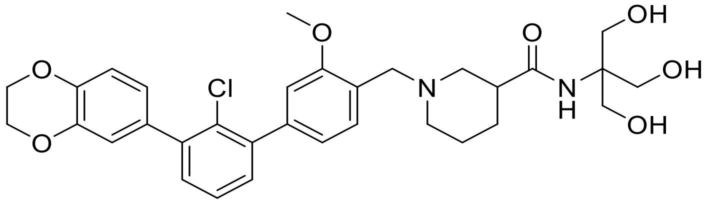

Method B. Reagent used: acid **4a** (0.200 g, 0.40 mmol), HATU (0.231 g, 0.61 mmol), DIPEA, (0.21 ml, 0.80 mmol), tris(hydroxymethyl)aminomethane (0.49 g, 4.0 mmol).Crude product was purified on column chromatography (SiO_2, chloroform/methanol/2M ammonia solution in chloroform 10:1:1) giving final product **4h** as a white solid with 60% yield (0.145 g).

R_f = 0.31, (SiO_2, chloroform/methanol 5:1); **^{1}H NMR** (600 MHz, $CDCl_3$) δ [ppm]: 9.76 (bs, 1H), 7.47-7.43 (m, 1H), 7.35-7.28 (m, 3H), 7.09-6.97 (m, 3H), 6.95-6.92 (m, 2H), 4.31 (s, 4H), 3.99-3.82 (m, 4H), 3.74 (bs, 1H), 3.32 (bs, 1H), 3.17 (bs, 2H), 3.06 (s, 3H), 2.97-2.88 (m, 3H), 2.50 (bs, 1H), 2.31 (bs, 1H), 1.96-1.73 (m, 3H), 1.54 (bs, 1H); **^{13}C NMR** (151 MHz, $CDCl_3$) δ [ppm]: 162.7, 157.4, 143.4, 143.1, 141.6, 141.2, 133.4, 131.1, 130.7, 130.2, 126.5, 122.9, 121.8, 121.6, 118.7, 117.0, 112.4,

64.6, 64.5, 56.3, 56.1, 55.8, 37.4, 36.6, 35.6, 31.6, 29.8, 26.9; **IR (ATR)** [cm^{-1}]: 3382, 2940, 1650, 1581, 1508, 1456, 1387, 1303, 1281, 1247, 1228, 1067, 1042, 833, 742; **LC-MS (DAD/ESI):** t_R = 5.64 min, calcd for. $C_{32}H_{37}ClN_2O_7$ (m/z) $[M+H]^+$ 597.24; found $[M+H]^+$ 597.29; **HRMS (ESI):** calcd for $C_{32}H_{37}ClN_2O_7$ [m/z] $[M+H]^+$ 597.2362; found, $[M+H]^+$ 597.2362

1-((2'-chloro-3'-(2,3-dihydrobenzo[b][1,4]dioxin-6-yl)-3-methoxy-[1,1'-biphenyl]-4-yl)methyl)-N',N'-dimethylpiperidine-4-carbohydrazide (**5d**)

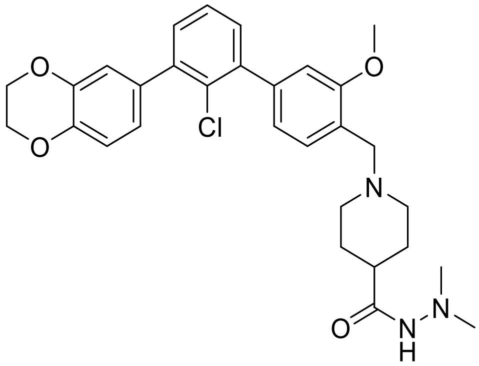

Method A. Reagent used: ester **5** (0.200 g, 0.38 mmol), LiOH monohydrate, (0.024 g, 0.57 mmol), HATU (0.220 g, 0.57 mmol), DIPEA, (0.20 ml, 1.14 mmol), 1,1-dimethylhydrazine (0.308 ml, 4.1 mmol). Crude product was purified on column chromatography (SiO_2, ethyl acetate/methanol 4:1) giving final product **5d** as colorless oil with 72% yield (0.147 g).

R_f = 0.21 (SiO_2, ethyl acetate/methanol 1:1); **^{1}H NMR** (600 MHz, DMSO-d_6) δ [ppm]: 8.68 (s, 1H), 7.43 (t, J = 7.5 Hz, 1H), 7.39-7.36 (m, 2H), 7.34 (dd, J = 7.4, 1.8 Hz, 1H), 7.02 (d, J = 1.4 Hz, 1H), 6.99 (d, J = 7.7 Hz, 1H), 6.95-6.92 (m, 2H0, 6.90 (dd, J = 8.3, 2.0 Hz, 1H), 4.28 (s, 4H), 3.80 (s, 3H), 3.47 (s, 2H), 2.91-2.85 (m, 2H), 2.43 (s, 6H), 2.03-1.99 (m, 1H), 1.98-1.92 (m, 2H), 1.62-1.56 (m, 4H); **^{13}C NMR** (151 MHz, DMSO-d_6) δ [ppm]: 171.8, 156.8, 143.1, 142.9, 141.1, 140.5, 139.1, 132.5, 130.4, 130.3, 129.9, 129.1, 127.0, 125.6, 122.4, 121.2, 118.0, 116.7, 112.0, 64.13, 64.10, 55.6, 55.5, 53.0, 48.1, 46.3, 28.5, 21.3; **IR (ATR)** [cm^{-1}]: 3212, 2936, 1660, 1613, 1573, 1507, 1454, 1393, 1302, 1279, 1245, 1229, 1067, 1042, 871, 795, 745; **LC-MS (DAD/ESI):** t_R = 5.36 min, calcd for. $C_{30}H_{34}ClN_3O_4$ (m/z) $[M+H]^+$ 536.23; found $[M+H]^+$ 536.21; **HRMS (ESI):** calcd for $C_{30}H_{34}ClN_3O_4$ [m/z] $[M+H]^+$ 536.2311; found, $[M+H]^+$ 536.2315

N-(2-aminoethyl)-1-((2'-chloro-3'-(2,3-dihydrobenzo[b][1,4]dioxin-6-yl)-3-methoxy-[1,1'-biphenyl]-4-yl)methyl)piperidine-4-carboxamide (**5e**)

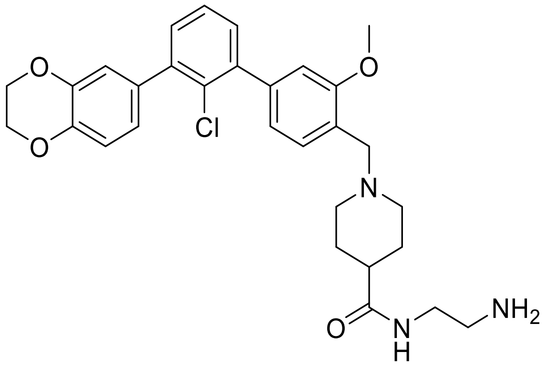

Method A. Reagent used: ester **5** (0.200 g, 0.38 mmol), LiOH monohydrate, (0.024 g, 0.57 mmol), HATU (0.21 g, 0.57 mmol), DIPEA, (0.20 ml, 1.14 mmol), N-Boc-ethylenediamine (0.60 ml, 3.80 mmol). Crude Boc-protected product was purified on column chromatography (SiO_2, ethyl acetate/methanol 4:1). Resulting oil was then deprotected by the reaction with 1M HCl with dioxane at room temperature for 16 hours. Then the reaction mixture was poured into 1M NaOH and extracted with

ethyl acetate. Organic layers were collected, dried over anhydrous $MgSO_4$, filtrated and evaporated giving final product **5e** with sufficient purity as yellowish soli with 59% yield (0.120 g).

R_f = 0.62, (SiO_2, ethyl acetate/methanol 1:1); **^{1}H NMR** (600 MHz, DMSO-d_6) δ [ppm]: 7.79 (t, J = 5.4 Hz, 1H), 7.42 (t, J = 7.5 Hz, 1H), 7.37 (d, J = 7.5 Hz, 2H), 7.34 (dd, J = 7.5, 1.8 Hz, 1H), 7.02 (d, J = 1.3 Hz, 1H), 7.00 (dd, J = 7.7, 1.4 Hz, 1H), 6.94-6.92 (m, 2H), 6.89 (dd, J = 8.3, 2.0 Hz, 2H), 4.28 (s, 4H), 3.79 (s, 3H), 3.47 (s, 2H), 3.06 (q, J = 6.2 Hz, 2H), 2.88-2.86 (m, 2H), 2.58 (t, J = 6.4 Hz, 2H), 2.11-2.07 (m, 1H), 1.99-1.95 (m, 2H), 1.65-1.59 (m, 4H); **^{13}C NMR** (151 MHz, DMSO-d_6) δ [ppm]: 174.7, 156.8, 143.1, 142.9, 141.1, 140.5, 139.1, 132.5, 130.5, 130.3, 129.9, 129.1, 127.0, 125.7, 122.4, 121.2, 118.0, 116.8, 112.0, 64.1, 64.1, 55.6, 55.5, 53.1, 42.1, 41.3, 40.9, 28.7; **IR (ATR)** [cm^{-1}]: 3285, 2933, 1642, 1568, 1505, 1456, 1387, 1302, 1279, 1245, 1226, 1067, 1042, 1029, 871, 794, 732; **LC-MS (DAD/ESI):** t_R = 4.49 min, calcd for. $C_{30}H_{34}ClN_3O_4$ (m/z) $[M+H]^+$ 536.23; found $[M+H]^+$ 536.21 **HRMS (ESI):** calcd for $C_{30}H_{34}ClN_3O_4$ [m/z] $[M+H]^+$ 536.2311; found, $[M+H]^+$ 536.2316

1-((2'-chloro-3'-(2,3-dihydrobenzo[b][1,4]dioxin-6-yl)-3-methoxy-[1,1'-biphenyl]-4-yl)methyl)-N-(1,3-dihydroxy-2-(hydroxymethyl)propan-2-yl)piperidine-4-carboxamide (**5h**)

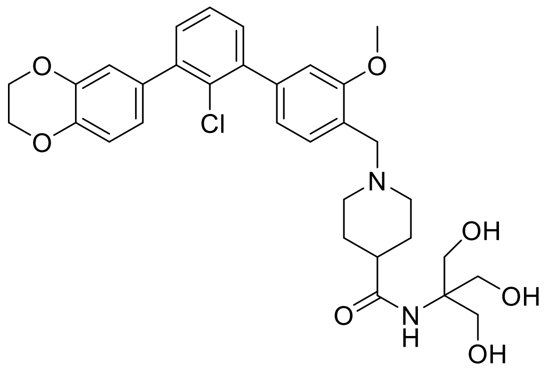

Method A. Reagent used: ester **5** (0.200 g, 0.38 mmol), LiOH monohydrate, (0.024 g, 0.57 mmol), HATU (0.220 g, 0.57 mmol), DIPEA, (0.20 ml, 1.14 mmol), tris(hydroxymethyl)aminomethane (0.460 g, 3.80 mmol). Crude product was purified on column chromatography (SiO_2, ethyl acetate/methanol 1:1) giving final product **5h** as yellowish oil with 48% yield (0.108 g).

R_f = 0.34 (SiO_2, ethyl acetate/methanol 1:1); **^{1}H NMR** (600 MHz, DMSO-d_6) δ [ppm]: 7.42 (t, J = 7.5 Hz, 1H), 7.39-7.35 (m, 2H), 7.33 (dd, J = 7.4, 1.5 Hz, 1H), 7.14 (s, 1H), 7.01 (s, 1H), 6.99 (d, J = 7.6 Hz, 1H), 6.95-6.91 (m, 2H), 6.89 (dd, J = 8.3, 1.8 Hz, 1H), 4.28 (s, 4H), 3.79 (s, 3H), 3.52 (s, 6H), 3.47 (s, 2H), 2.91-2.84 (m, 2H), 2.26-2.21 (m, 1H), 2.01-1.91 (m, 2H), 1.70-1.54 (m, 4H); **^{13}C NMR** (151 MHz, DMSO-d_6) δ [ppm]: 176.2, 156.8, 143.1, 142.9, 141.1, 140.5, 139.1, 132.6, 130.5, 130.4, 129.9, 129.2, 127.0, 125.6, 122.4, 121.2, 118.1, 116.8, 112.0, 64.2, 64.1, 62.1, 60.8, 55.6, 55.5, 53.0, 42.4, 28.8; **IR (ATR)** [cm^{-1}]: 3290, 2938, 1645, 1565, 1507, 1455, 1398, 1303, 1280, 1245, 1231, 1066, 1042, 1025, 871, 794, 744; **LC-MS (DAD/ESI):** t_R = 5.09 min, calcd for. $C_{32}H_{37}ClN_2O_7$ (m/z) $[M+H]^+$ 597.24; found $[M+H]^+$ 597.22; **HRMS (ESI):** calcd for $C_{32}H_{37}ClN_2O_7$ [m/z] $[M+H]^+$ 597.2362; found, $[M+H]^+$ 597.2368

4.2. Solubility measurements

Solubility of the compounds was determined using Nepheloskan Ascent® (Labsystems). The experiments were performed at room temperature in 96-well plates in a final volume of 300 μL. Each compound was tested in triplicate at the concentrations of 12.5, 25, 50 and 100 μM in PBS1X with 0.2% DMSO. The measurements were performed at 3 different times (0 h, 12 h, 24 h) from the preparation of the samples. Data obtained for each sample have been normalized with respect to the control sample (PBS with 0.2% DMSO). The compounds with normalized signal average below 3-fold of control were considered soluble in tested conditions.

4.3. Homogenous Time-Resolved Fluorescence

HTRF (Homogeneous Time Resolved Fluorescence) is a widely used technology for homogenous analyte measurement, particularly in high-throughput screening for drug target studies. This technology combines fluorescence resonance energy transfer (FRET) with time-resolved measurement. In TR-FRET assays, a signal is generated when a donor and an acceptor molecule are in proximity (so-called FRET distance), transfer energy through FRET. Both fluorophores are attached to the monoclonal antibodies that are then specifically detecting particular tags on target proteins.

The commercially available Cis-Bio assay kit was used to perform the HTRF assay. Following the standard protocol, the measurement was carried out at a 20 μL final volume with a 5 nM concentration of hPD-1 and 50 nM concentration of hPD-L1 in the final formulation. Separate dilution series were performed to calculate the half-maximal inhibitory concentration (IC_{50}) of the most potent compounds. Analyte and detection reagents (anti-analyte conjugated donor and acceptor conjugated antibody) were placed in the proper wells on the microplate as suggested in the Cis-Bio protocol. The plate was left for 1 h of incubation and it was read on an HTRF certified microplate reader Tecan Spark 20M. Output data were subjected to background subtraction on negative control (no PD-1), and normalization in correlation to the positive control (no inhibitor) and averaged. Resulting data points were then fitted with Hill's equation to generate IC_{50} using Mathematica 12.

4.4. PD-1/PD-L1 immune checkpoint blockade (ICB) assay

For the in vitro analysis of the bioactivity of the molecules an Immune Checkpoint Blockade assay was performed[4] CHO/TCRAct/PD-L1 cells (Promega), overexpressing an artificial TCR-activator construct and PD-L1, were seeded on 96-well white plates at the density of 10 000 cells/well. The next day, the cells were overlaid with the effector Jurkat T cells (Jurkat-ECs, Promega, 20 000 cells/well), overexpressing PD-1 and containing construct assuring an NFAT-induced expression of luciferase, either in the presence of the indicated concentrations of the compounds with DMSO-only as a control (the concentration of DMSO was kept constant at 0.1%) or durvalumab (Selleckchem), as a positive control, with untreated cells as controls. Activation of the Jurkat-ECs, reflected by luciferase activity, was monitored by luminescence measurements after 6 h of incubation (37°C, 5%CO_2), and

20 min of additional incubation with the Bio-GloTM Assay reagent (Promega) at room temperature. The luminescence was read on the Spark microplate reader (Tecan). The data is presented as fold induction of the luminescence signal relative to either untreated (for durvalumab) or DMSO-treated (for compounds) cells. Data points represent mean ± SD values from duplicates.

4.5. Protein expression and crystallization

The plasmid encoding of the PD-L1 protein (amino acids 18 – 134) was used to transform E. coli BL21 strain. The bacteria carrying the plasmid were then grown at 37 ºC until reaching an OD600nm of 0.6 – 0.8. The protein expression was then induced by adding a final concentration of 1 mM IPTG and the expression was then carried overnight at the same temperature. The PD-L1 protein was then isolated in the form of inclusion bodies, which were subsequently washed using a protocol previously described.[5] After washing the inclusion bodies, the protein was refolded by slowly dropping around 50 mg of inclusion bodies into a solution containing 0.1 M Tris-HCl pH 8.0, 1 M L-arginine-HCl, 0.25 mM oxidized glutathione, and 0.25 mM reduced glutathione. After the refolding step, the protein was dialyzed 3 times against buffer containing 10 mM Tris-HCl pH 8.0 and 20 mM NaCl. The final step of purification consisted of size exclusion chromatography using a Superdex 75 column and 10 mM Tris- HCl, 20 mM NaCl based buffer for crystallization or PBS buffer for NMR based experiments. The correctly folded state and purity of the PD-L1 protein were checked by NMR and SDS-PAGE respectively.

The freshly purified protein was concentrated to 5 mg/mL and then mixed in a 1:3 molar ratio with an excess of the 2f inhibitor, finally the solution was centrifuged for 1 minute at max speed. The clarified solution was then used for screening using multiple available commercial screens. The diffraction quality crystals were obtained from a sitting drop set up at room temperature with a buffer containing 27 % w/v PEG 3350, 0.1 M Bis-Tris propane, pH 7.0 and 0.2 M Lithium sulfate. Crystals were cryo-protected using glycerol and flash frozen with liquid nitrogen.

The X-ray diffraction data were collected at the BL13 - XALOC beamline at ALBA (Barcelona, Spain).[6]The data were indexed, integrated, and scaled using XDS, SCALE, and Aimless.[7–9] The initial phase estimates was obtained by molecular replacement calculated in Phaser using PDB:5C3T as a model.[10] The refinement was performed using CCP4cloud[11] and verified using Coot.[12–14] The structure was deposited in the Protein Data Bank with accession number 8R6Q. The protein-ligand interactions were assessed using PLIP server[15] and visualized using PyMol (The PyMOL Molecular Graphics System, Version 2.0 Schrödinger, LLC).

4.6. Crystal structure determination for compound 2a

X-ray diffraction data for single crystals of compound **2a** was collected using XtalLAB Synergy-S four-circle diffractometer with a mirror monochromator and a microfocus CuKα radiation source (λ = 1.5418 Å). The CryoStream cryostat system was used to allow low-temperature

experiments, performed at 100(2) K. The obtained data sets were processed with CrysAlisPro software (Rigaku-Oxford Diffraction; CrysAlisPro Oxford Diffraction Ltd, Abingdon, England V 1. 171. 36. 2.; release 27-06-2012 CN, 2006.). The phase problem was solved with direct methods using SIR2014. [16] Parameters of the obtained model were refined by full-matrix least-squares on F2 using SHELXL 2014/6.[17] Calculations were performed using WinGX integrated system (ver. 2014.1).[18] Figures were prepared with Mercury 4.0 software.[19]

All non-hydrogen atoms were refined anisotropically. All hydrogen atoms attached to carbon atoms were positioned with the idealized geometry and refined using the riding model with the isotropic displacement parameter Uiso[H] = 1.2 Ueq[C] for all but the methyl group, for which Uiso[H] = 1.5 Ueq[C] was applied. The hydrogen atom at N24, being a result of the tertiary amine protonation, was located on the Fourier difference map and refined with no restraints on Uiso parameter. In the structure water channels are observed, propagating along [100] axis. The four water molecules in the asymmetric unit are highly disordered, with two of them located in the proximity of the crystallographic 2-fold axis, leading to two alternative positions of O3w and O4w water molecules. Due to the observed disorder of water molecules, their hydrogen atoms' coordinates were obtained by the Fourier difference map inspection, supported by CALC-OH predictive algorithm[20] available in the WinGX suite.[18] Additionally, those hydrogen atoms were refined using the riding model with the isotropic displacement parameter Uiso[H] = 1.5 Ueq[O], with restrains on distances and angles (DFIX and DANG commands, respectively) to maintain relatively good water molecule geometry. The disordered water channels lead to the partial, positional disorder within the β-proline fragment which is involved in interactions with the mentioned water molecules. This disorder was modeled based on the Fourier difference map inspection and the site occupancies were determined during the refinement procedure.

Crystal structure data of **2a** has been deposited with the Cambridge Crystallographic Data Centre - accession no. CCDC 2231594

4.7. Molecular modeling

Protein preparation procedure

Prior to the molecular docking simulations, the PD-L1 dimer was prepared using Maestro (Version 12.5.139) (Schrödinger Release 2020-3: Maestro, Schrödinger, LLC, New York). The hydrogen atoms were added in the idealized positions. The missing side chains were added using the conformers' library. The ligand originally bound to the protein as well as water molecules were removed for the next step of the experiment.

Ligands preparation procedure

The 3D geometries of the investigated m-terphenyl derivatives were obtained from SMILES with OpenBabel (version 2.4.1).[21] Additionally, the possible protonation at pH=7.4 was predicted with

Calculator Plugins in Marvin 19.12.0, 2019, ChemAxon (http://www.chemaxon.com). The ionic form was applied if 60 % or more molecules were ionized at the set pH. For the range of 40-60 % both forms (neutral & ionized) were considered. Otherwise, the compound was kept in the neutral form. The ionization was introduced in Maestro 3D Builder. The initial geometries were minimized with the OPLS3 force field.[22]

Molecular Docking Procedure

Molecular docking experiments were performed using GOLD 2021.3.0 (Genetic Optimisation for Ligand Docking) software.[23] The binding region was defined based on the location of the native ligand, including all atoms within 8 Å, to prevent binding to the corresponding, mirror-like site formed in the homodimer. During the semi-flexible docking process, ligands were allowed flexibility to find the most probable binding pose, using a genetic algorithm (GA) implemented in GOLD [M4]. The empirical ChemPLP scoring function was applied to evaluate the obtained results. Three top-scored results have been recorded for each docked molecule. Compound A,[24] BMS-1166[25,26] m-terphenly analog from 7NLD crystal structure[3] and **2f** compound have been used as reference ligands in the docking procedure, after their geometry randomization (conversion of SMILES to .mol2 followed by energy minimization), and ionization state prediction as described in "Ligand preparation procedure" section.

5. SUPPLEMENTARY REFERENCES

(1) Groom, C. R.; Bruno, I. J.; Lightfoot, M. P.; Ward, S. C. The Cambridge Structural Database. *Acta Crystallographica Section B* **2016**, *72* (2), 171–179. https://doi.org/10.1107/S2052520616003954.

(2) Bruno, I. J.; Cole, J. C.; Edgington, P. R.; Kessler, M.; Macrae, C. F.; McCabe, P.; Pearson, J.; Taylor, R. New Software for Searching the Cambridge Structural Database and Visualizing Crystal Structures. *Acta Crystallographica Section B* **2002**, *58* (3–1), 389–397. https://doi.org/10.1107/S0108768102003324.

(3) Muszak, D.; Surmiak, E.; Plewka, J.; Magiera-Mularz, K.; Kocik-Krol, J.; Musielak, B.; Sala, D.; Kitel, R.; Stec, M.; Weglarczyk, K.; Siedlar, M.; Dömling, A.; Skalniak, L.; Holak, T. A. Terphenyl-Based Small-Molecule Inhibitors of Programmed Cell Death-1/Programmed Death-Ligand 1 Protein-Protein Interaction. *J Med Chem* **2021**, *64* (15), 11614–11636. https://doi.org/10.1021/acs.jmedchem.1c00957.

(4) Cheng, Z.-J. J.; Karassina, N.; Grailer, J.; Hartnett, J.; Fan, F.; Cong, M. Abstract 5440: Novel PD-1 Blockade Bioassay to Assess Therapeutic Antibodies in PD-1 and PD-L1 Immunotherapy Programs. In *Cancer research*; American Association for Cancer Research, **2015**; Vol. 75, pp 5440–5440. https://doi.org/10.1158/1538-7445.AM2015-5440.

(5) Magiera-Mularz, K.; Skalniak, L.; Zak, K. M.; Musielak, B.; Rudzinska-Szostak, E.; Berlicki, Ł.; Kocik, J.; Grudnik, P.; Sala, D.; Zarganes-Tzitzikas, T.; Shaabani, S.; Dömling, A.; Dubin, G.; Holak, T. A. Bioactive Macrocyclic Inhibitors of the PD-1/PD-L1 Immune Checkpoint. *Angewandte Chemie International Edition* **2017**, *56* (44), 13732–13735. https://doi.org/10.1002/ANIE.201707707.

(6) Juanhuix, J.; Gil-Ortiz, F.; Cuní, G.; Colldelram, C.; Nicolás, J.; Lidón, J.; Boter, E.; Ruget, C.; Ferrer, S.; Benach, J. Developments in Optics and Performance at BL13-XALOC, the Macromolecular Crystallography Beamline at the Alba Synchrotron. *J Synchrotron Radiat* **2014**, *21* (4), 679–689. https://doi.org/10.1107/S160057751400825X.

(7) Evans, P. R.; Murshudov, G. N. How Good Are My Data and What Is the Resolution? *Acta Crystallographica Section D* **2013**, *69* (7), 1204–1214. https://doi.org/10.1107/S0907444913000061.

(8) Kabsch, W. XDS. *Acta Crystallogr D Biol Crystallogr* **2010**, *66* (Pt 2), 125–132. https://doi.org/10.1107/S0907444909047337.

(9) Krug, M.; Weiss, M. S.; Heinemann, U.; Mueller, U. {\it XDSAPP}: A Graphical User Interface for the Convenient Processing of Diffraction Data Using {\it XDS}. *J Appl Crystallogr* **2012**, *45* (3), 568–572. https://doi.org/10.1107/S0021889812011715.

(10) McCoy, A. J.; Grosse-Kunstleve, R. W.; Adams, P. D.; Winn, M. D.; Storoni, L. C.; Read, R. J. Phaser Crystallographic Software. *J Appl Crystallogr* **2007**, *40* (4), 658–674. https://doi.org/10.1107/S0021889807021206.

(11) Winn, M. D.; Ballard, C. C.; Cowtan, K. D.; Dodson, E. J.; Emsley, P.; Evans, P. R.; Keegan, R. M.; Krissinel, E. B.; Leslie, A. G. W.; McCoy, A.; McNicholas, S. J.; Murshudov, G. N.; Pannu, N. S.; Potterton, E. A.; Powell, H. R.; Read, R. J.; Vagin, A.; Wilson, K. S. Overview of the CCP4 Suite and Current Developments. *Acta Crystallogr D Biol Crystallogr* **2011**, *67* (Pt 4), 235–242. https://doi.org/10.1107/S0907444910045749.

(12) Adams, P. D.; Afonine, P. V.; Bunkóczi, G.; Chen, V. B.; Davis, I. W.; Echols, N.; Headd, J. J.; Hung, L. W.; Kapral, G. J.; Grosse-Kunstleve, R. W.; McCoy, A. J.; Moriarty, N. W.; Oeffner, R.; Read, R. J.; Richardson, D. C.; Richardson, J. S.; Terwilliger, T. C.; Zwart, P. H. PHENIX: A Comprehensive Python-Based System for Macromolecular Structure Solution. *Acta Crystallogr D Biol Crystallogr* **2010**, *66* (Pt 2), 213–221. https://doi.org/10.1107/S0907444909052925.

(13) Emsley, P.; Cowtan, K. Coot: Model-Building Tools for Molecular Graphics. *Acta Crystallogr D Biol Crystallogr* **2004**, *60* (Pt 12 Pt 1), 2126–2132. https://doi.org/10.1107/S0907444904019158.

(14) Joosten, R. P.; Long, F.; Murshudov, G. N.; Perrakis, A. The PDB_REDO Server for Macromolecular Structure Model Optimization. *IUCrJ* **2014**, *1* (Pt 4), 213–220. https://doi.org/10.1107/S2052252514009324.

(15) Adasme, M. F.; Linnemann, K. L.; Bolz, S. N.; Kaiser, F.; Salentin, S.; Haupt, V. J.; Schroeder, M. PLIP 2021: Expanding the Scope of the Protein–Ligand Interaction Profiler to DNA and RNA. *Nucleic Acids Res* **2021**, *49* (W1), W530–W534. https://doi.org/10.1093/NAR/GKAB294.

(16) Burla, M. C.; Caliandro, R.; Carrozzini, B.; Cascarano, G. L.; Cuocci, C.; Giacovazzo, C.; Mallamo, M.; Mazzone, A.; Polidori, G. Crystal Structure Determination and Refinement via SIR2014. *J Appl Crystallogr* **2015**, *48* (1), 306–309. https://doi.org/10.1107/S1600576715001132.

(17) Sheldrick, G. M. A Short History of SHELX. *Acta Crystallographica Section A* **2008**, *64* (1), 112–122. https://doi.org/10.1107/S0108767307043930.

(18) Farrugia, L. J. WinGX Suite for Small-Molecule Single-Crystal Crystallography. *J Appl Crystallogr* **1999**, *32* (4), 837–838. https://doi.org/10.1107/S0021889899006020.

(19) MacRae, C. F.; Sovago, I.; Cottrell, S. J.; Galek, P. T. A.; McCabe, P.; Pidcock, E.; Platings, M.; Shields, G. P.; Stevens, J. S.; Towler, M.; Wood, P. A. Mercury 4.0: From Visualization to Analysis, Design and Prediction. *J Appl Crystallogr* **2020**, *53*, 226–235. https://doi.org/10.1107/S1600576719014092.

(20) Nardelli, M. Modeling Hydroxyl and Water H Atoms. *J Appl Crystallogr* **1999**, *32* (3), 563–571. https://doi.org/10.1107/S0021889899002666.

(21) O'Boyle, N. M.; Banck, M.; James, C. A.; Morley, C.; Vandermeersch, T.; Hutchison, G. R. Open Babel: An Open Chemical Toolbox. *J Cheminform* **2011**, *3* (10). https://doi.org/10.1186/1758-2946-3-33.

(22) Harder, E.; Damm, W.; Maple, J.; Wu, C.; Reboul, M.; Xiang, J. Y.; Wang, L.; Lupyan, D.; Dahlgren, M. K.; Knight, J. L.; Kaus, J. W.; Cerutti, D. S.; Krilov, G.; Jorgensen, W. L.; Abel, R.; Friesner, R. A. OPLS3: A Force Field Providing Broad Coverage of Drug-like Small Molecules and Proteins. *J Chem Theory Comput* **2016**, *12* (1), 281–296. https://doi.org/10.1021/acs.jctc.5b00864.

(23) Verdonk, M. L.; Cole, J. C.; Hartshorn, M. J.; Murray, C. W.; Taylor, R. D. Improved Protein-Ligand Docking Using GOLD. *Proteins: Structure, Function and Genetics* **2003**, *52* (4), 609–623. https://doi.org/10.1002/prot.10465.

(24) Park, J. J.; Thi, E. P.; Carpio, V. H.; Bi, Y.; Cole, A. G.; Dorsey, B. D.; Fan, K.; Harasym, T.; Iott, C. L.; Kadhim, S.; Kim, J. H.; Lee, A. C. H.; Nguyen, D.; Paratala, B. S.; Qiu, R.; White, A.; Lakshminarasimhan, D.; Leo, C.; Suto, R. K.; Rijnbrand, R.; Tang, S.; Sofia, M. J.; Moore, C. B. Checkpoint Inhibition through Small Molecule-Induced Internalization of Programmed Death-Ligand 1. *Nature Communications 2021 12:1* **2021**, *12* (1), 1–11. https://doi.org/10.1038/s41467-021-21410-1.

(25) Chupak, L.; Ding, M.; Martin, S.; Zheng, X.; Hewawasam, P.; Connoly, T.; Xu, N.; Yeung, K.; Zhu, J.; Langley, D.; Tenney, D.; Scola, P. Compounds Useful as Immunomodulators, Bristol-Myers Squibb, WO 2015/160641 A2., 2015.

(26) Chupak, L. S.; Zheng, X. Compounds Useful as Immunomodulators, Bristol-Myers Squibb Company, WO 2015/034820 A1. WO 2015/034820 A1, 2015.

6. COPIES OF THE NMR OF THE FINAL COMPOUNDS

1a: ^{1}H NMR, 600 MHz, $CDCl_3$

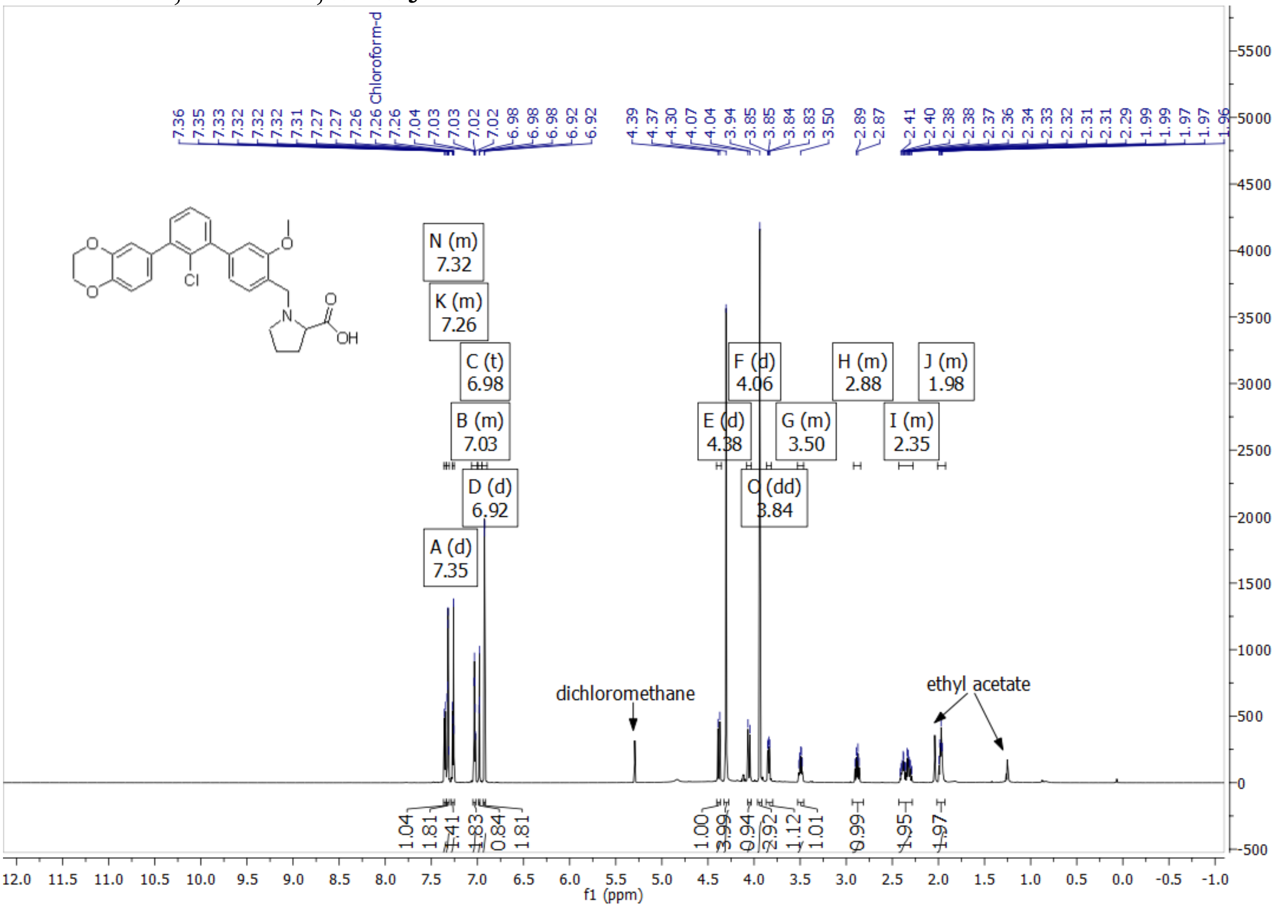

1a: ^{13}C NMR, 151 MHz, $CDCl_3$

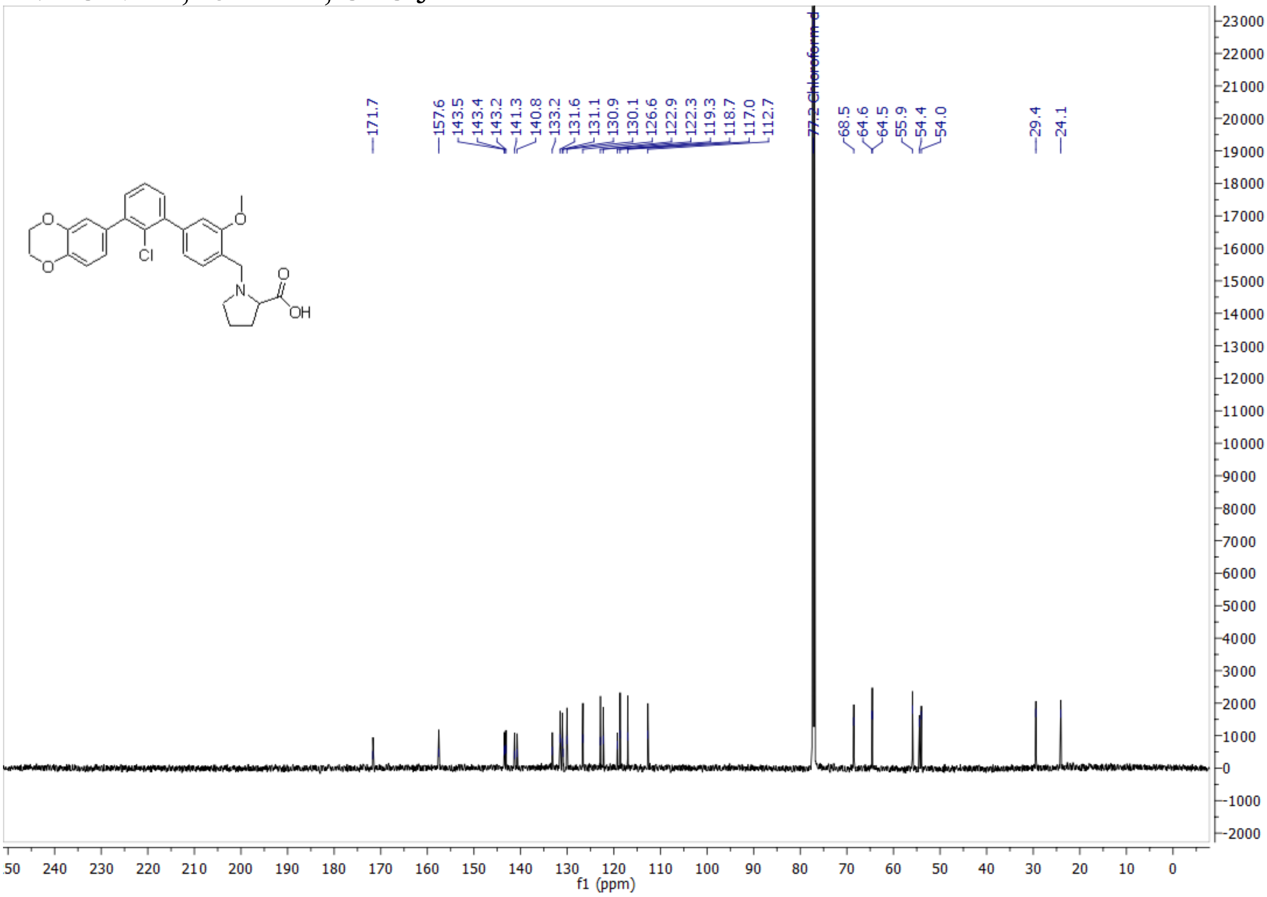

1b: ^{1}H NMR, 600 MHz, $CDCl_3$

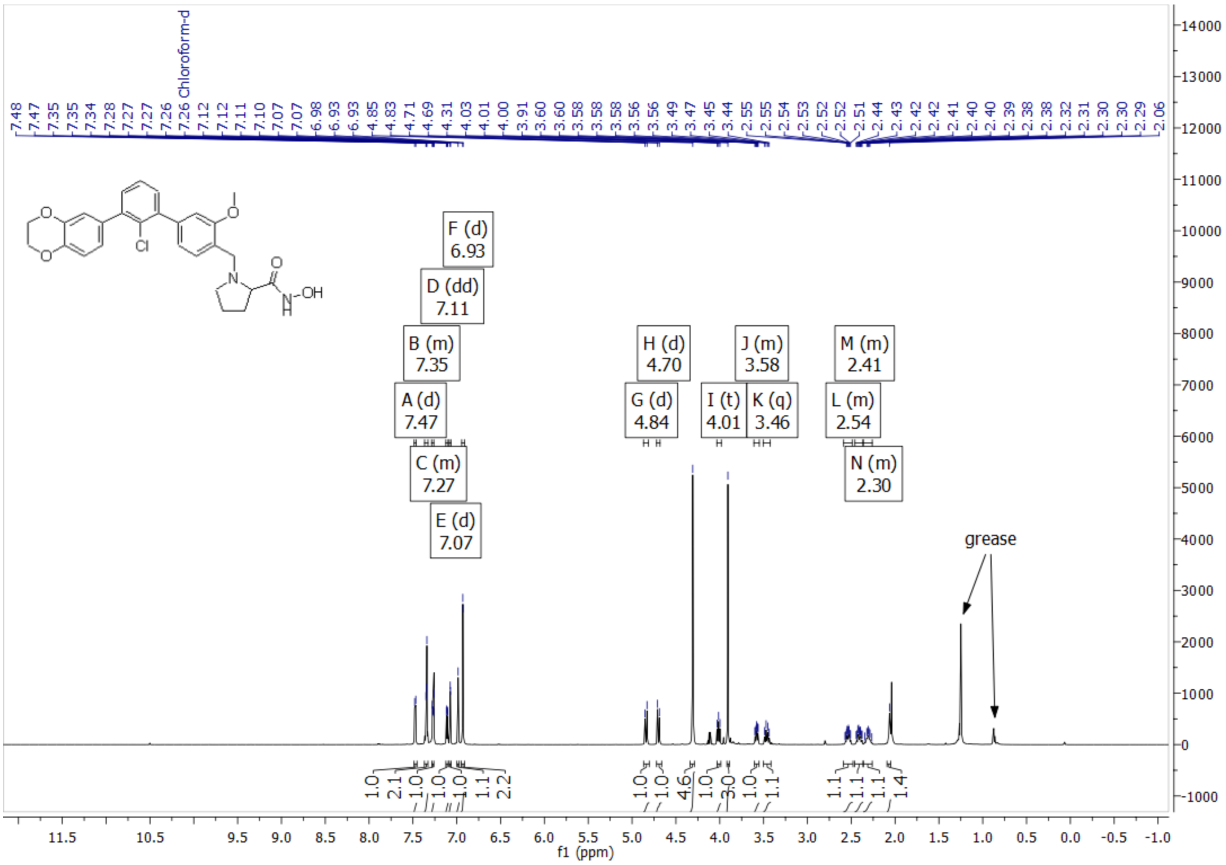

1b: ^{13}C NMR, 600 MHz, $CDCl_3$

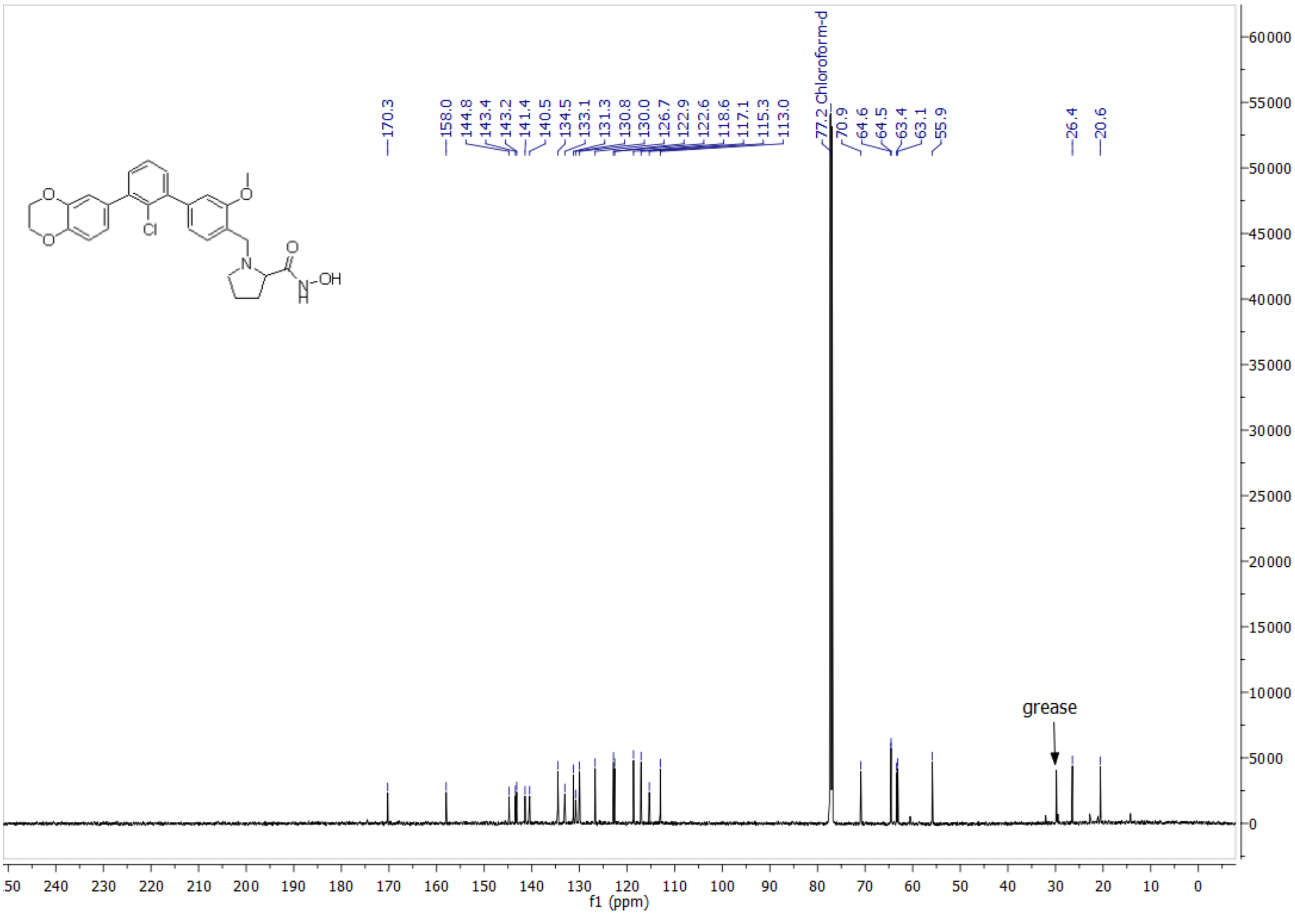

1c: ^{1}H NMR, 600 MHz, CDCl$_3$

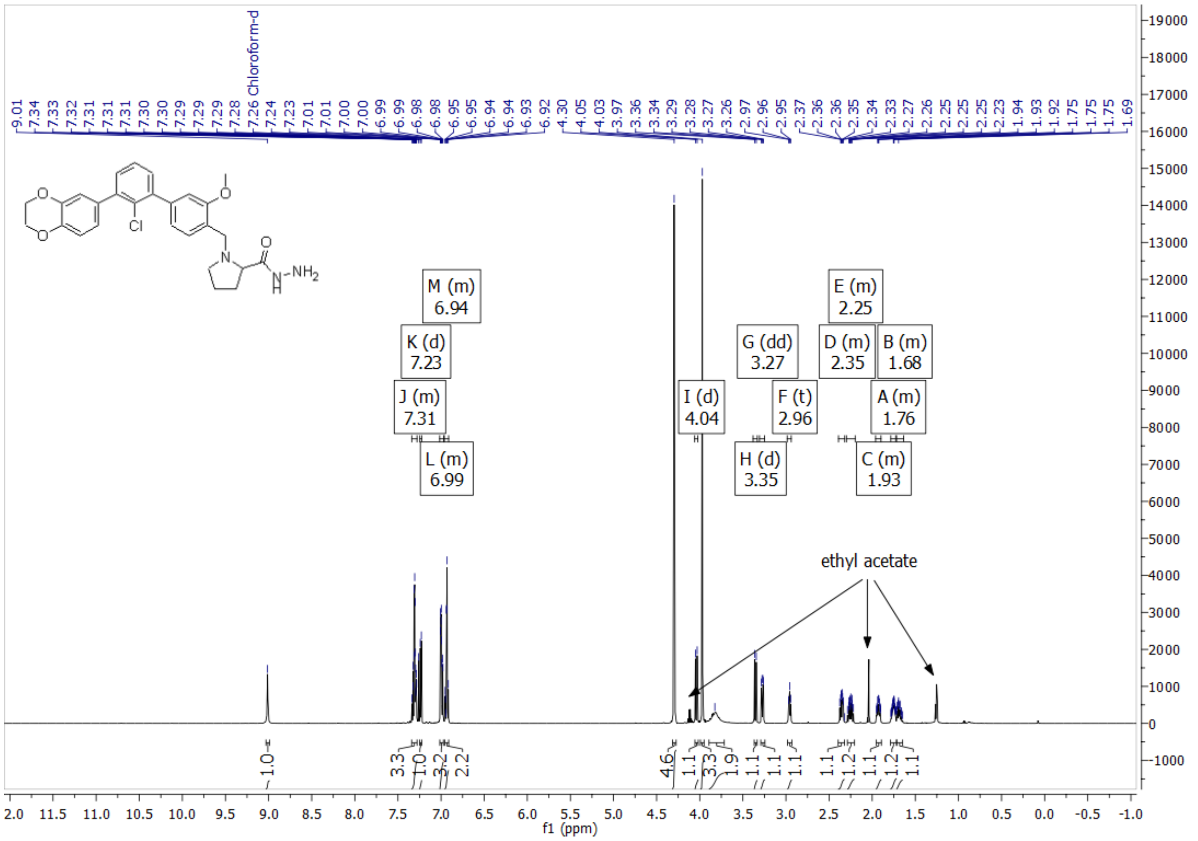

1c: ^{1}H NMR, 151 MHz, CDCl$_3$

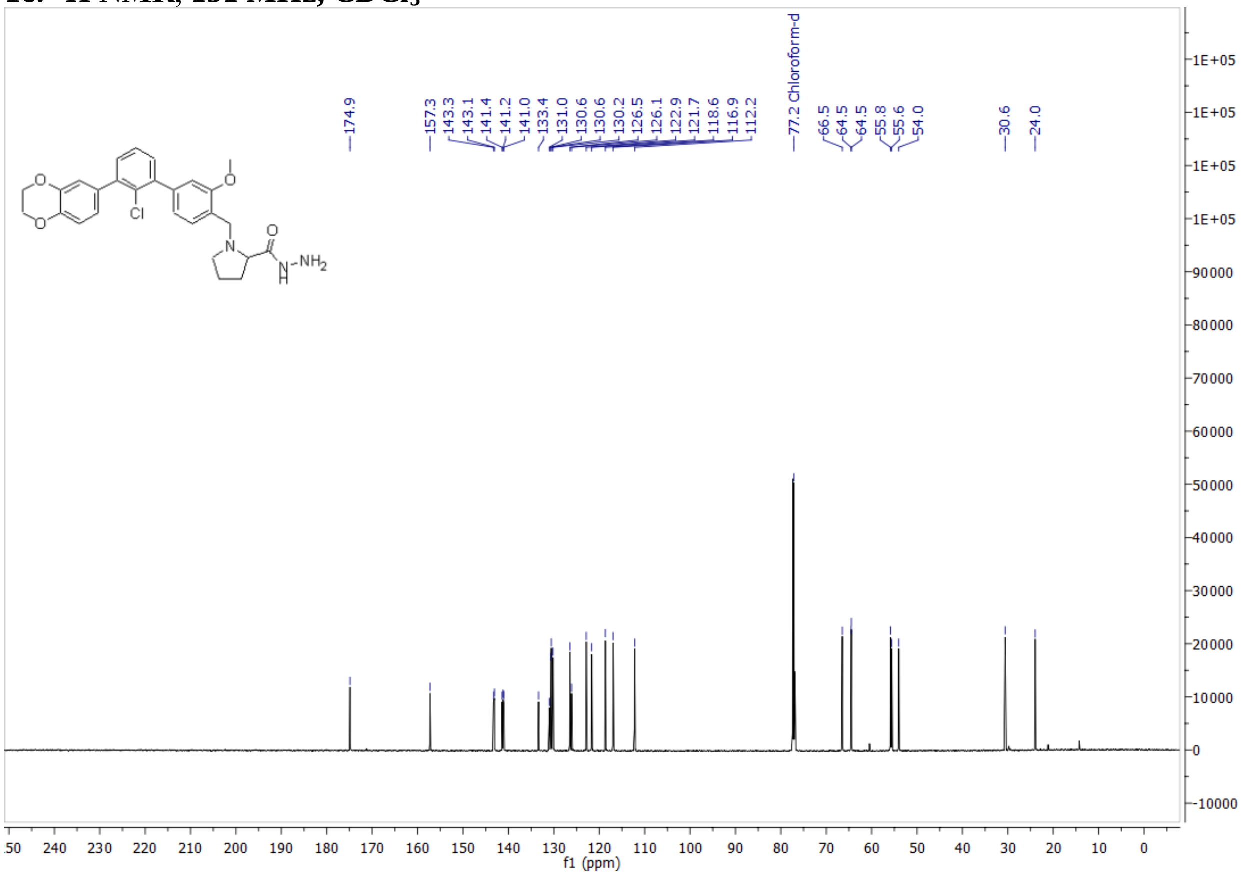

1d: ^{1}H NMR, 600 MHz, CDCl$_3$

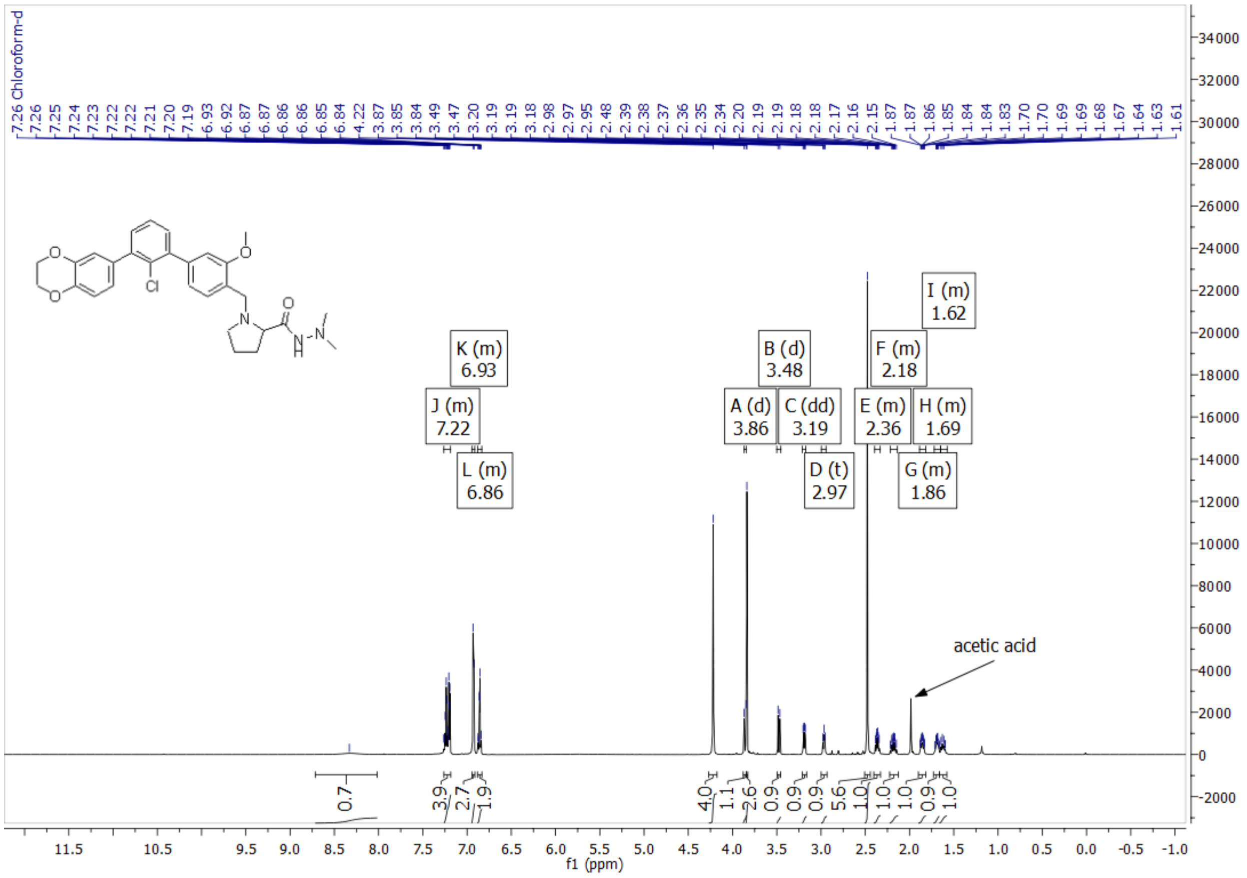

1d: ^{13}C NMR, 151 MHz, CDCl$_3$

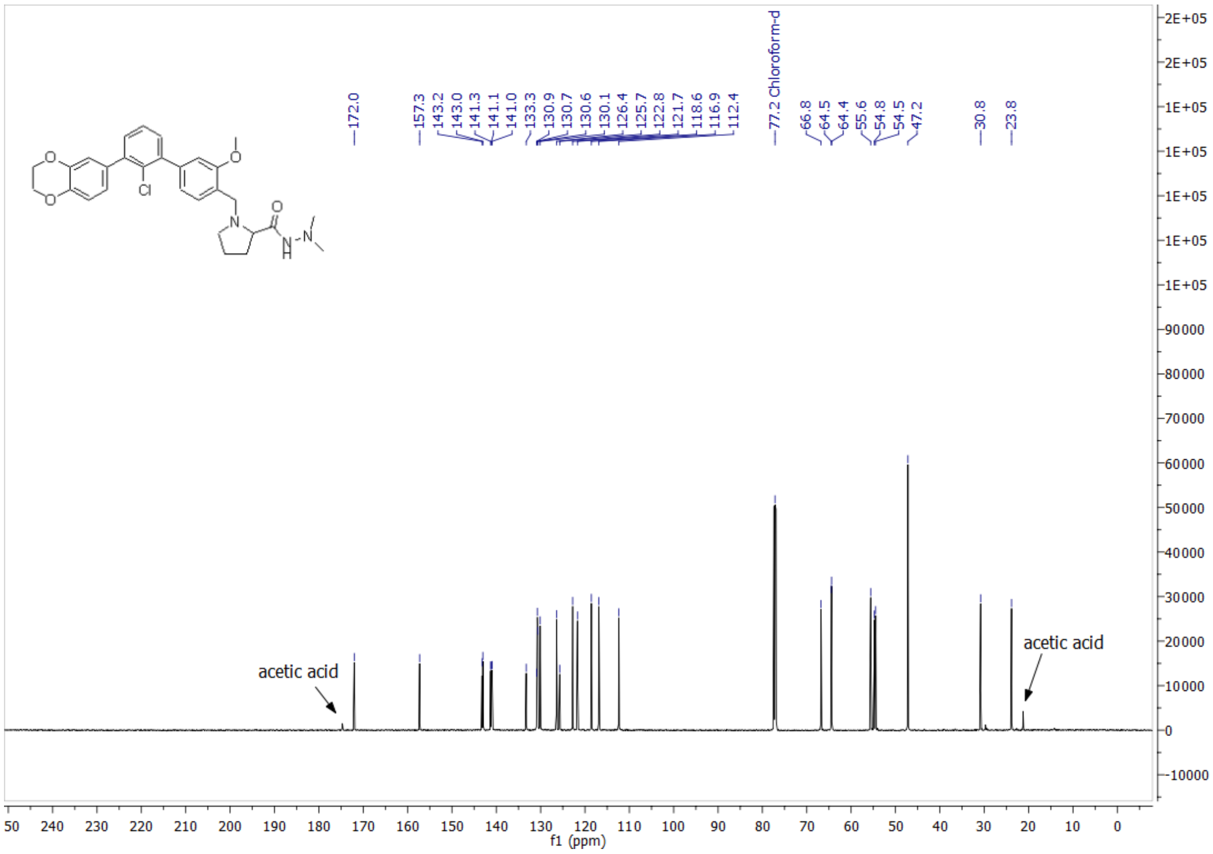

1e: ^{1}H NMR, 600 MHz, $CDCl_3$

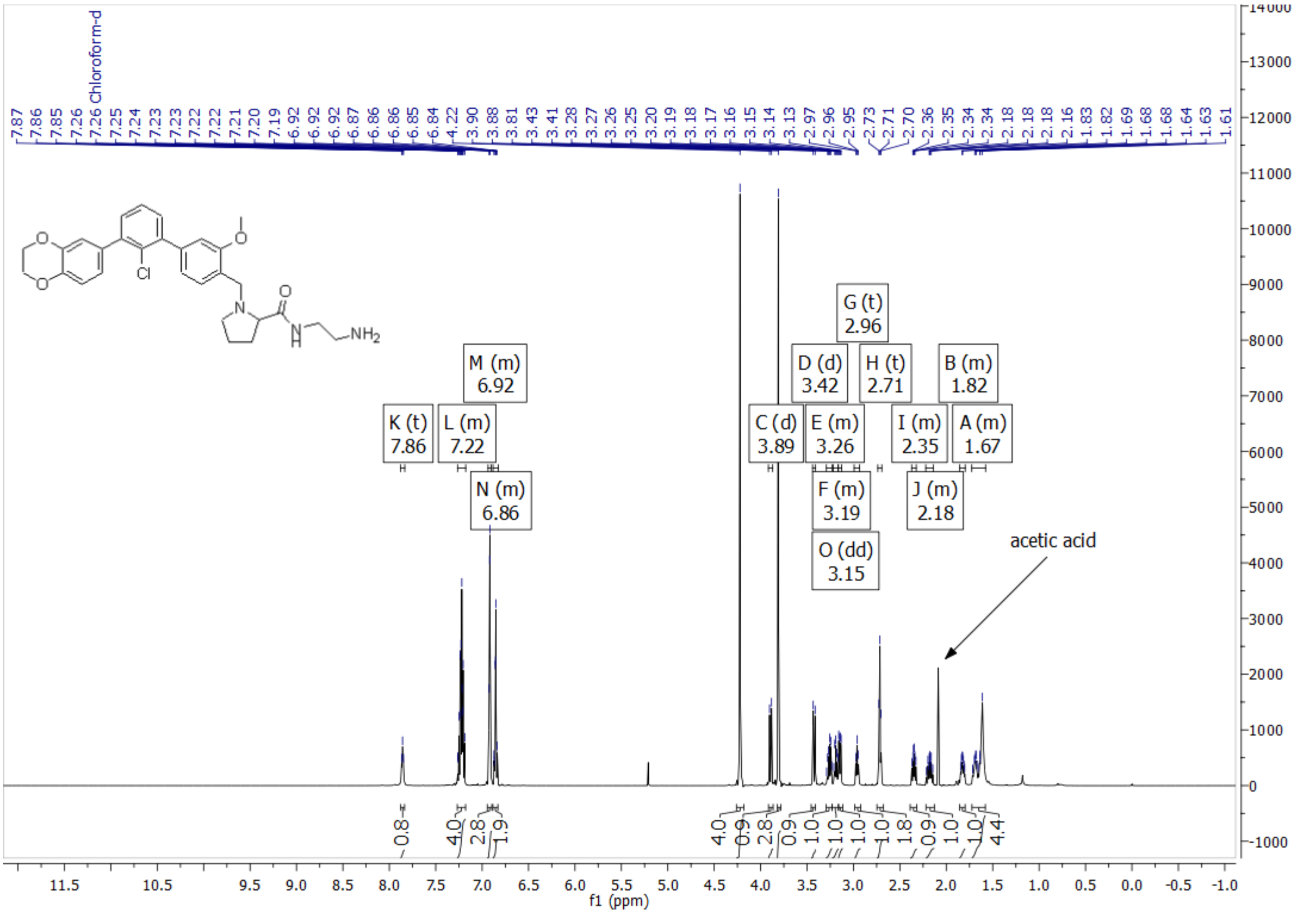

1e: ^{13}C NMR, 151 MHz, $CDCl_3$

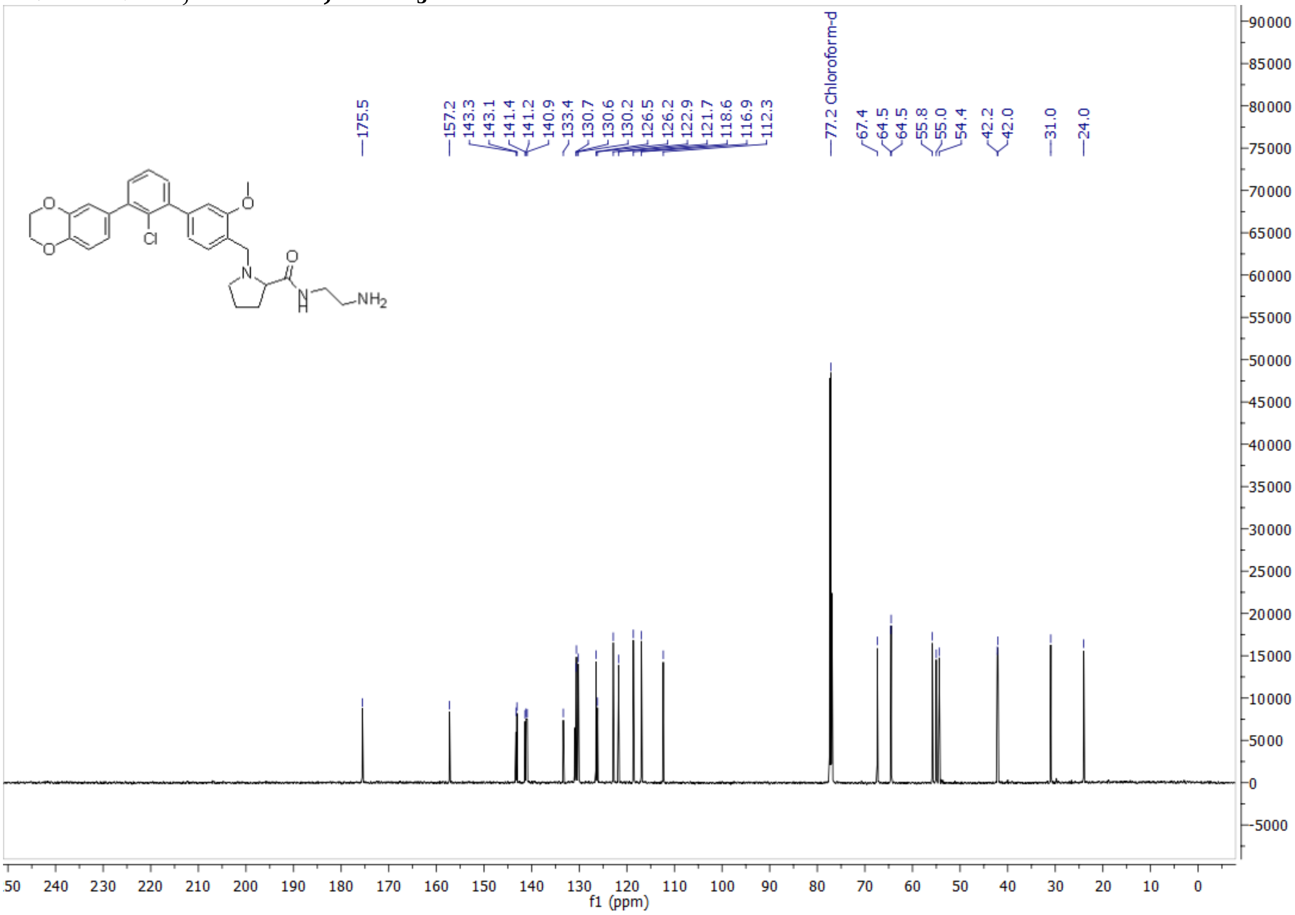

1f: ^{1}H NMR, 600 MHz, $CDCl_3$

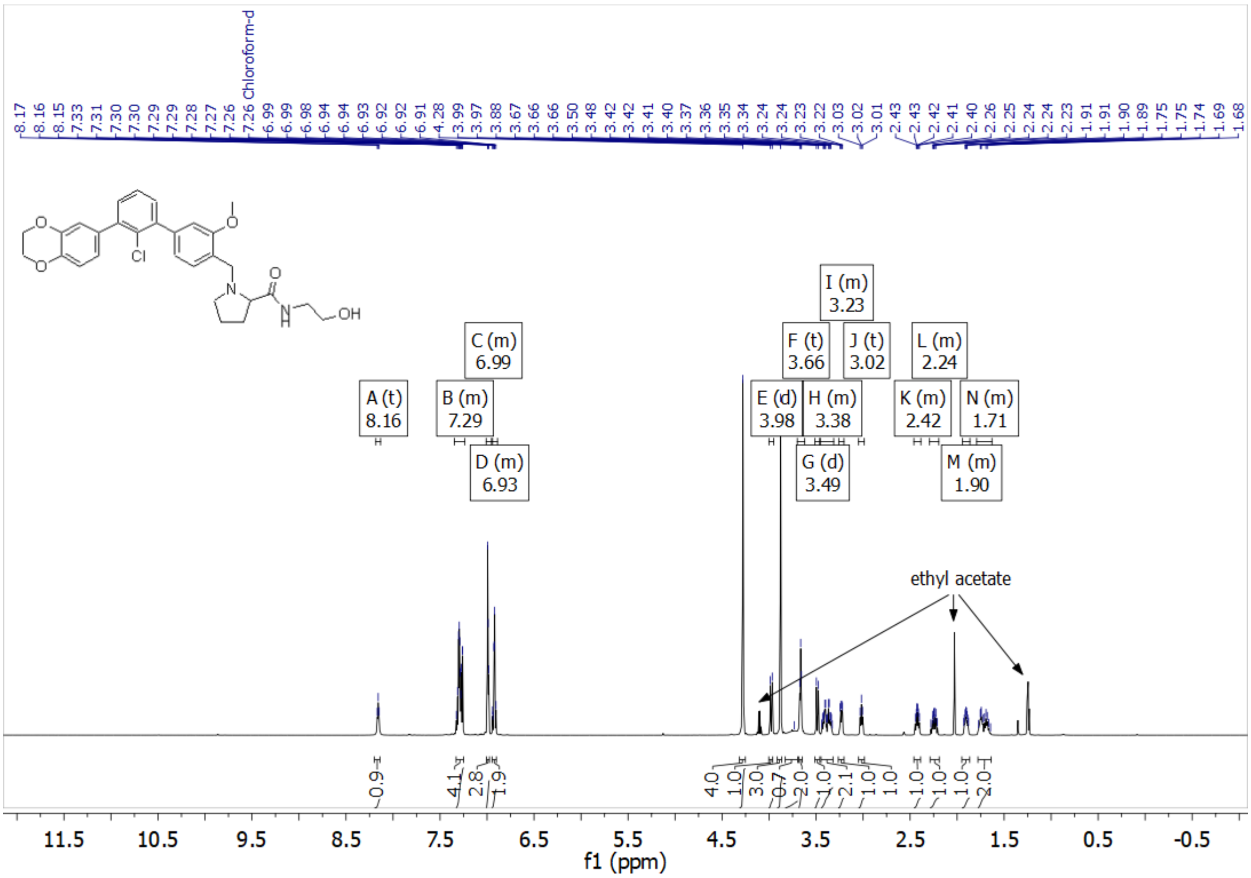

1f: ^{13}C NMR, 151 MHz, $CDCl_3$

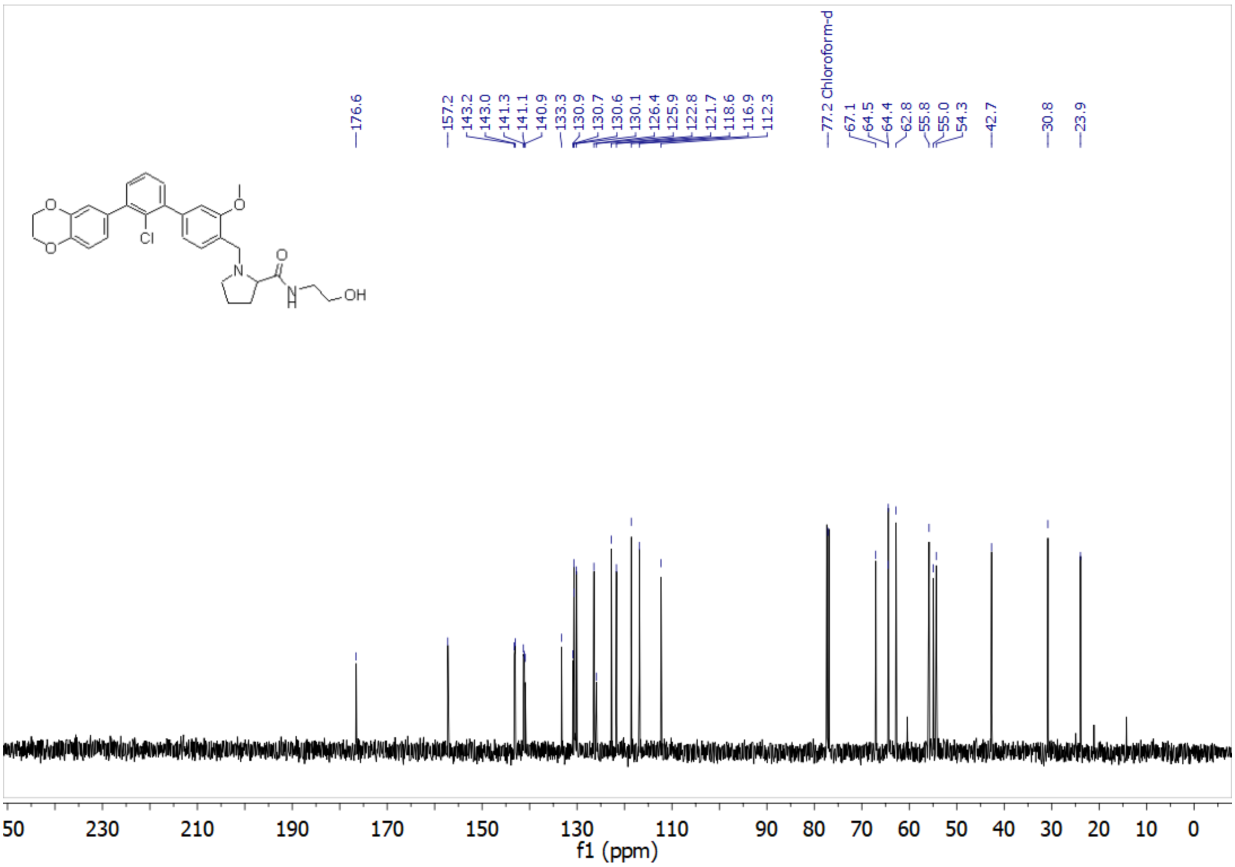

1g: ^{1}H NMR, 600 MHz, $CDCl_3$

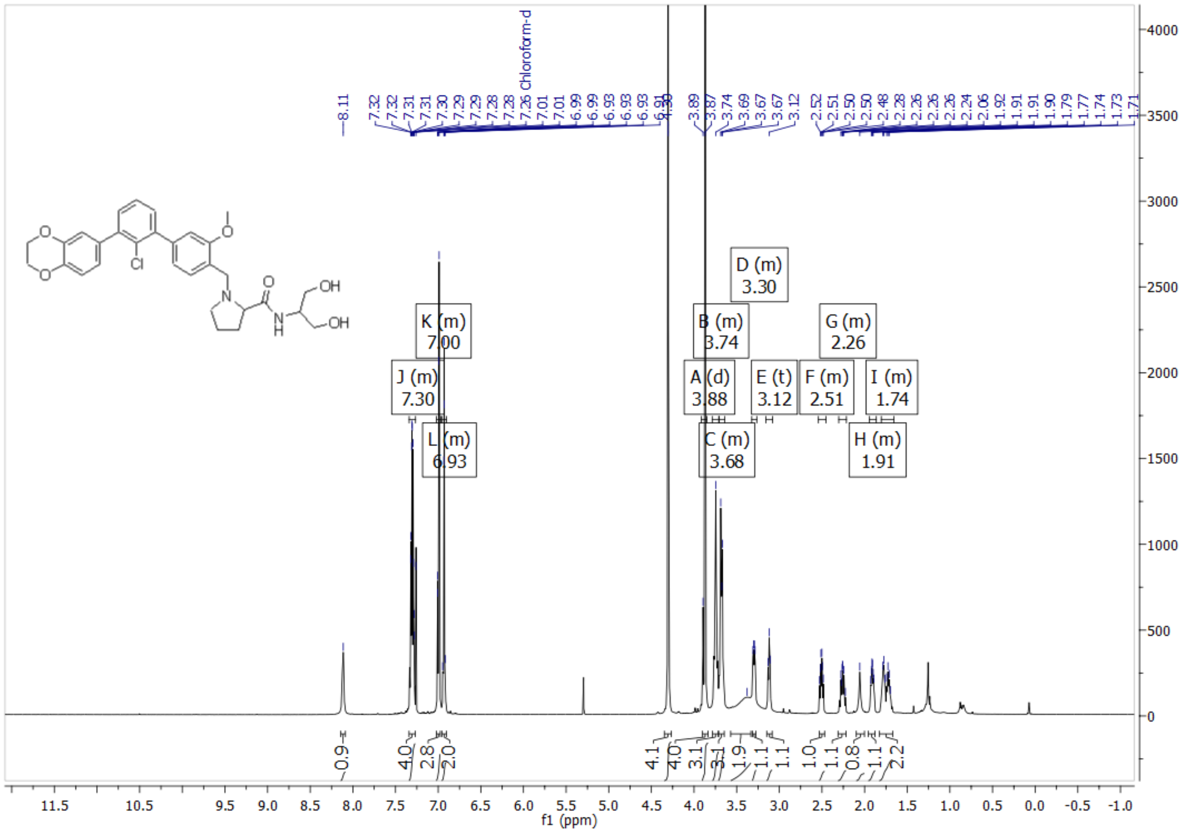

1g: ^{13}C NMR, 151 MHz, $CDCl_3$

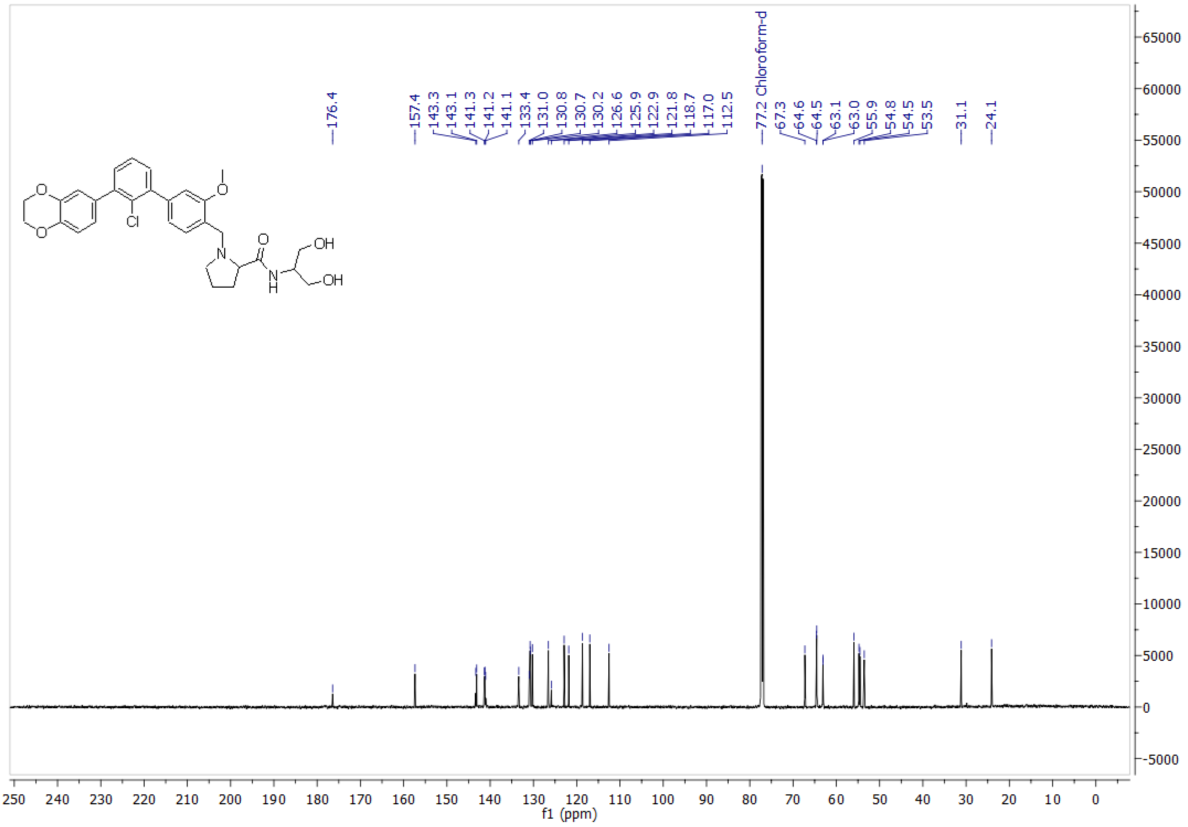

1h: ^{1}H NMR, 600 MHz, $CDCl_3$

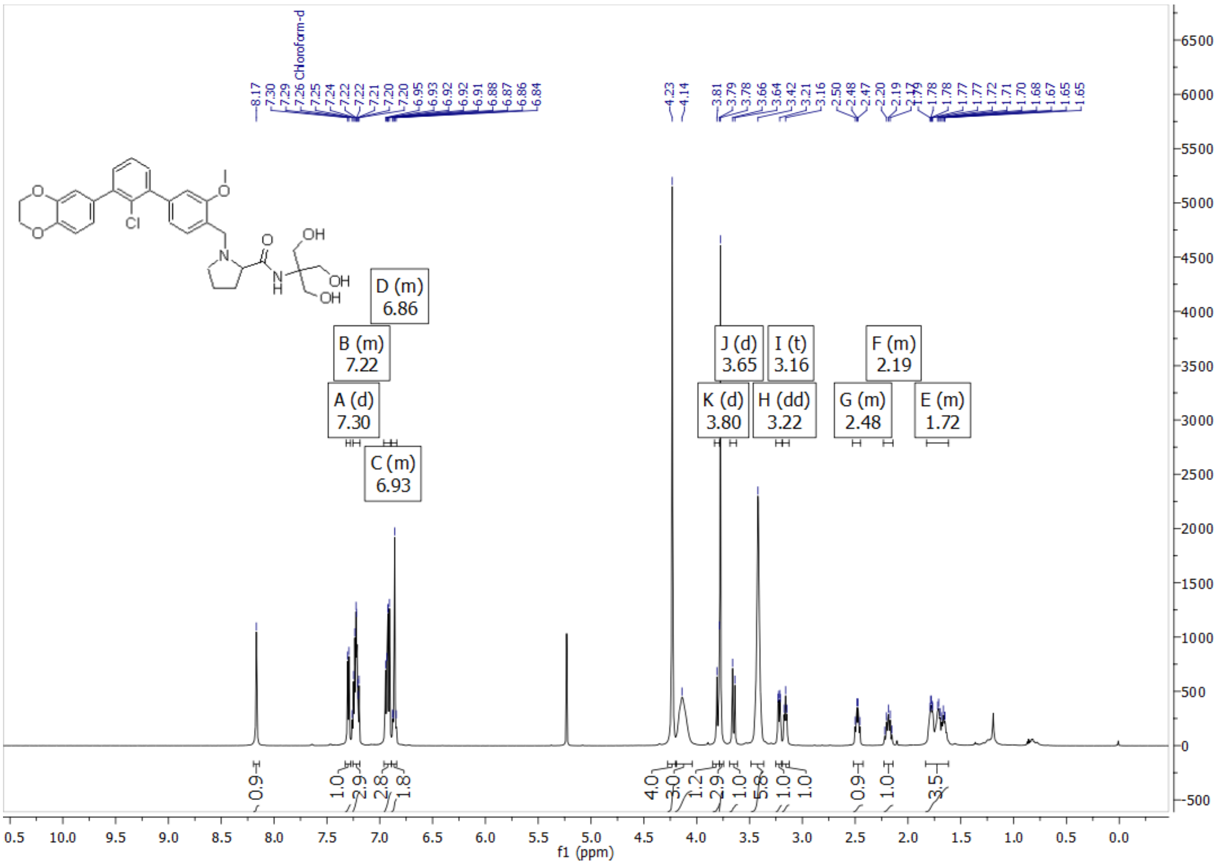

1h: ^{13}C NMR, 151 MHz, $CDCl_3$

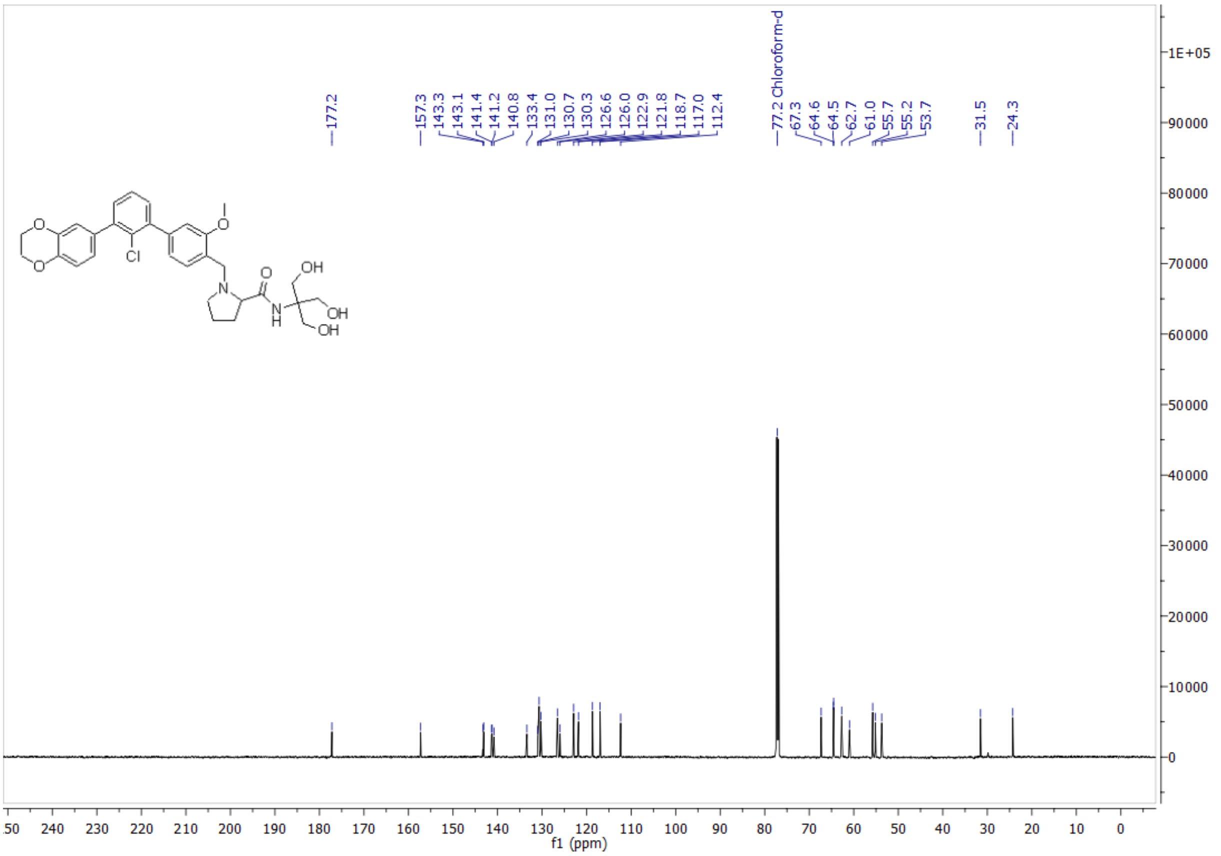

2a: ^{1}H NMR, 600 MHz, $CDCl_3$

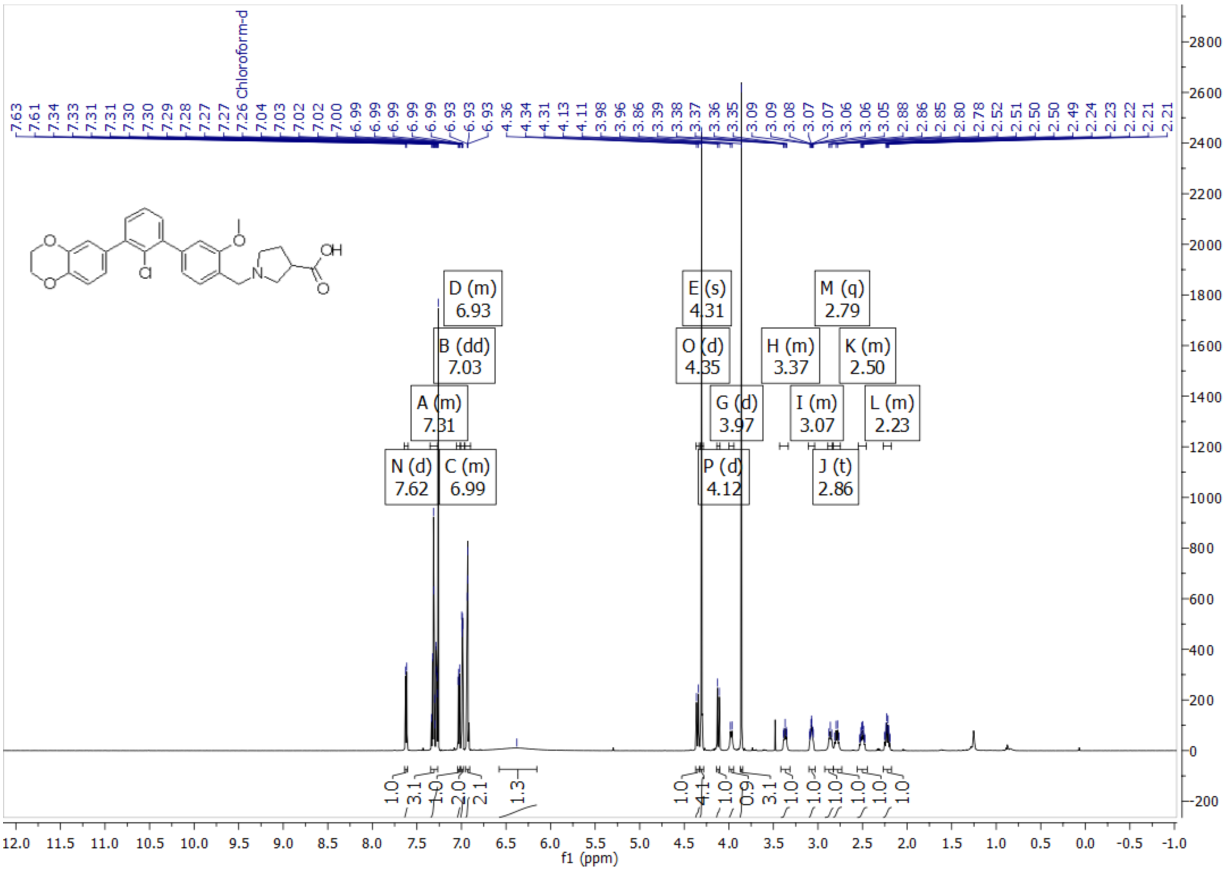

2a: ^{13}C NMR, 151 MHz, $CDCl_3$

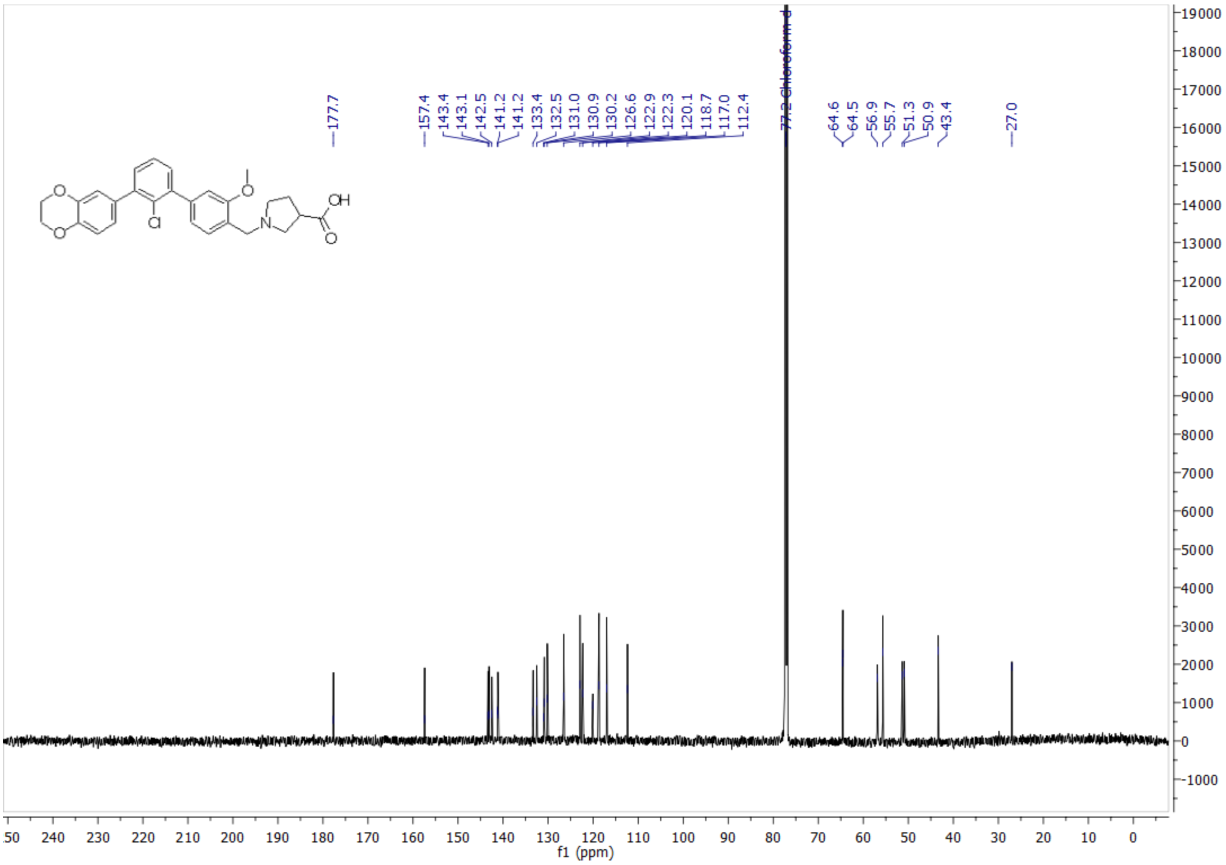

2b: ^{1}H NMR, 600 MHz, $CDCl_3$

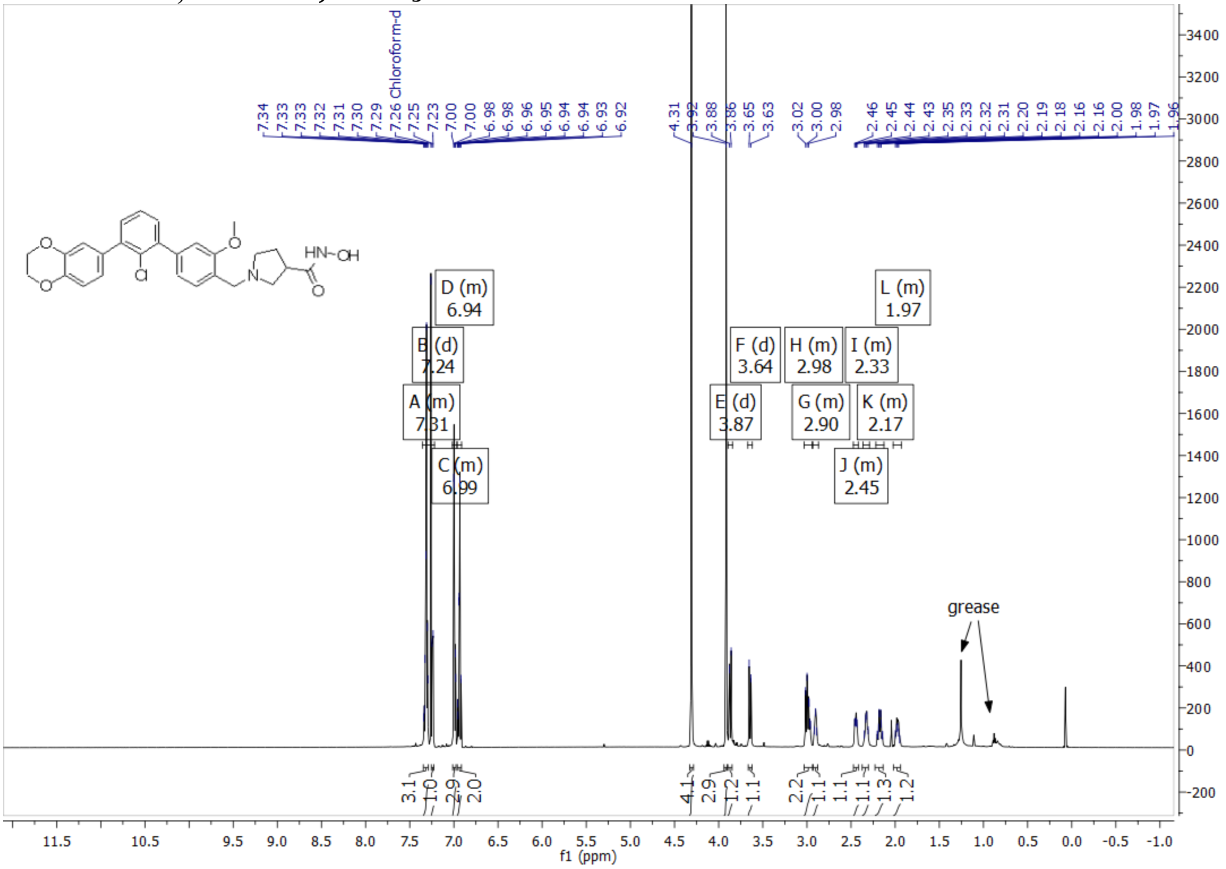

2b: ^{13}C NMR, 151 MHz, $CDCl_3$

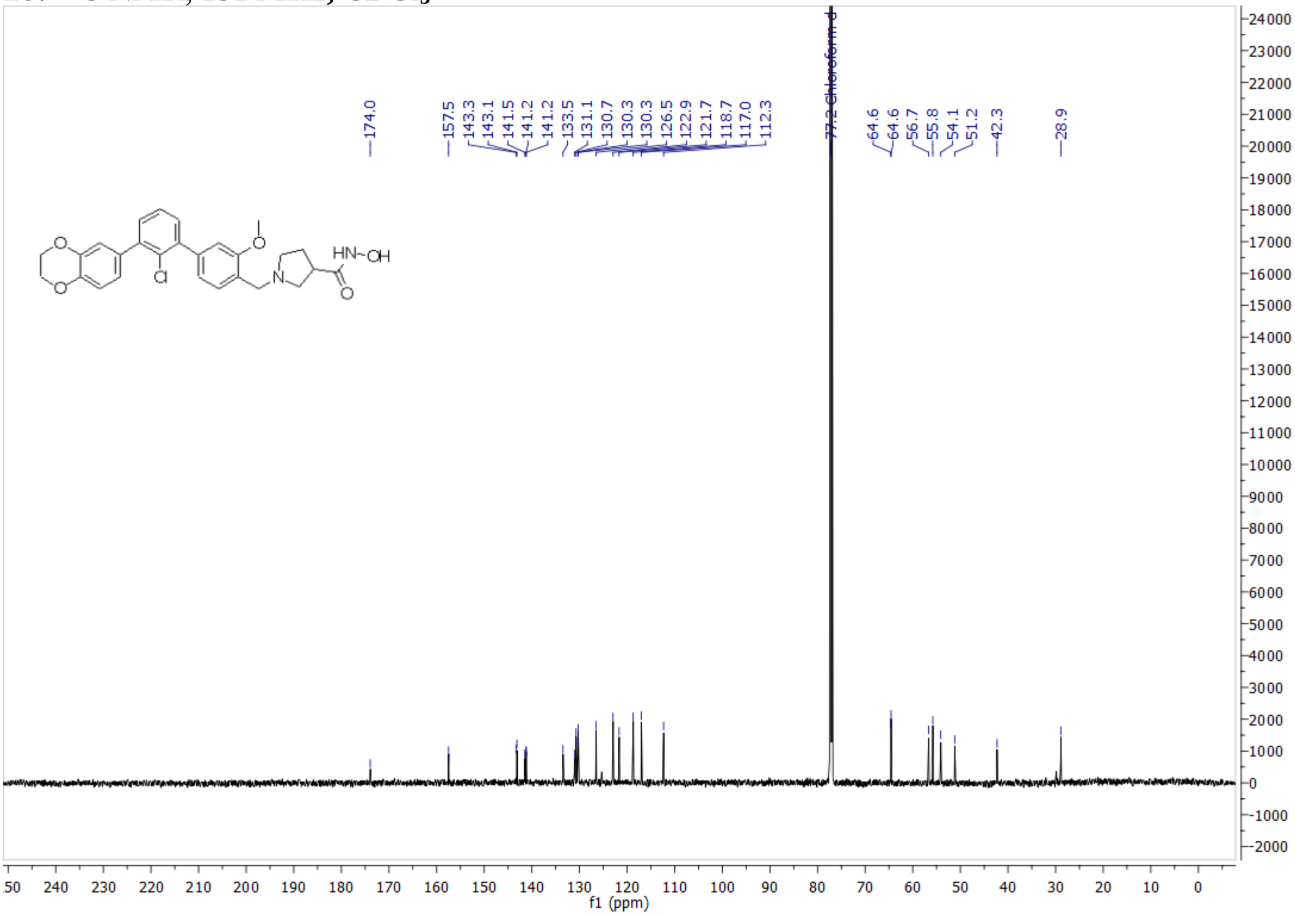

2c: ^{1}H NMR, 600 MHz, DMSO-d_6

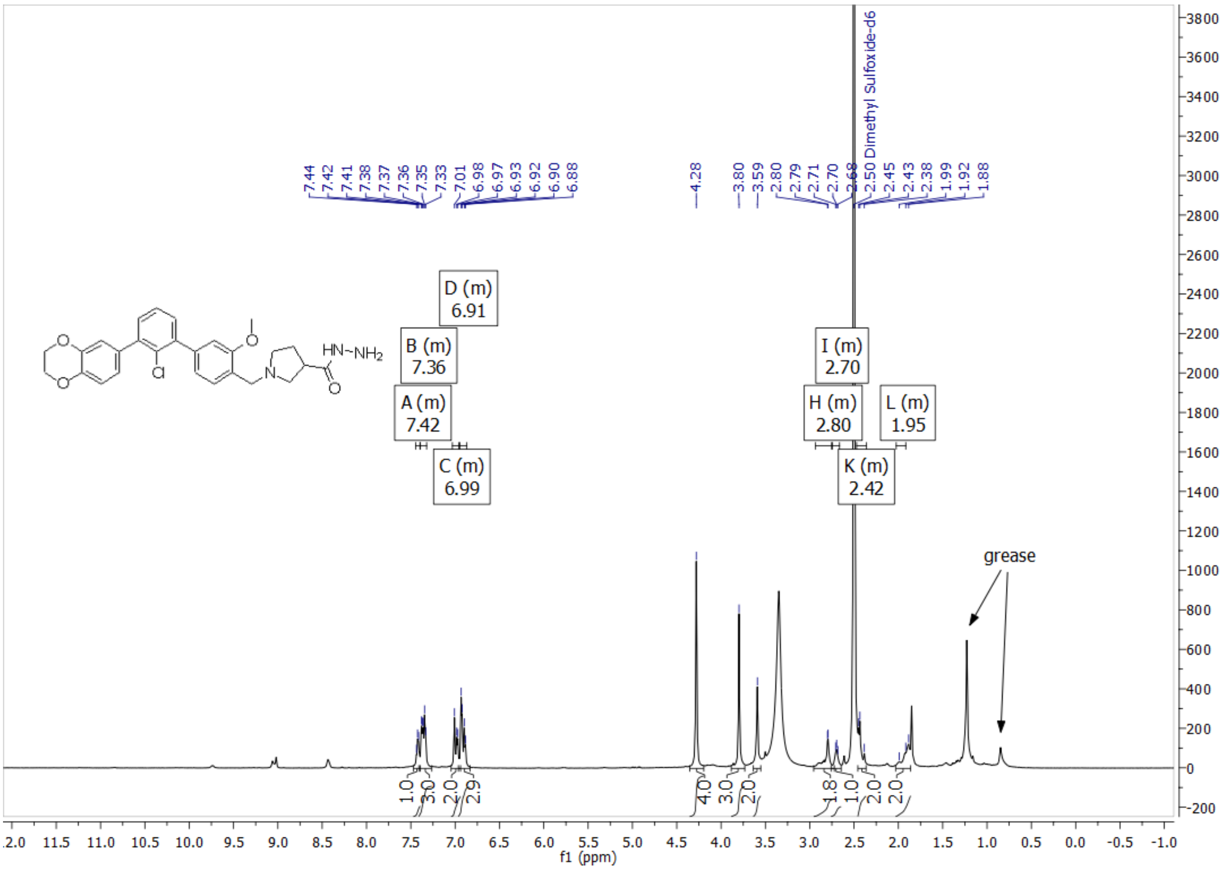

2c: ^{13}C NMR, 151 MHz, DMSO-d_6

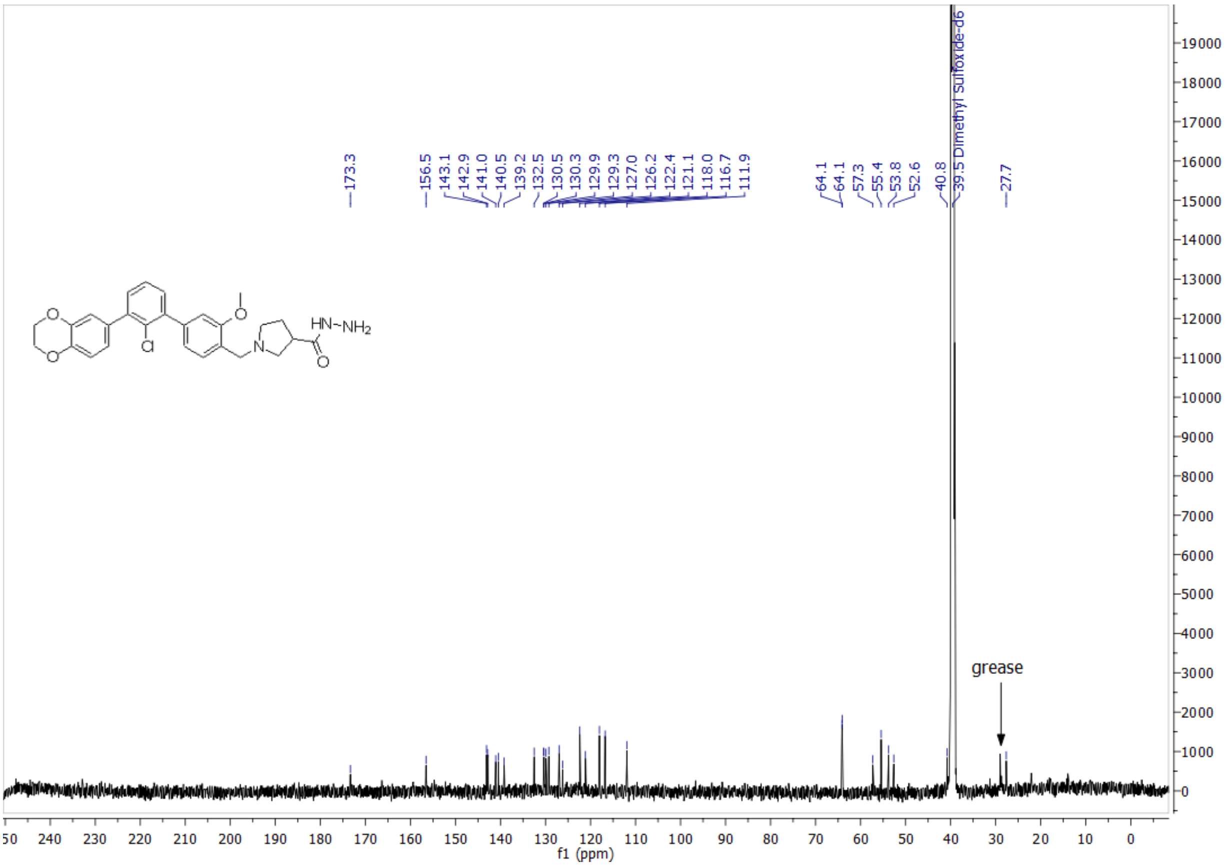

2d: ^{1}H NMR, 600 MHz, DMSO-d_6

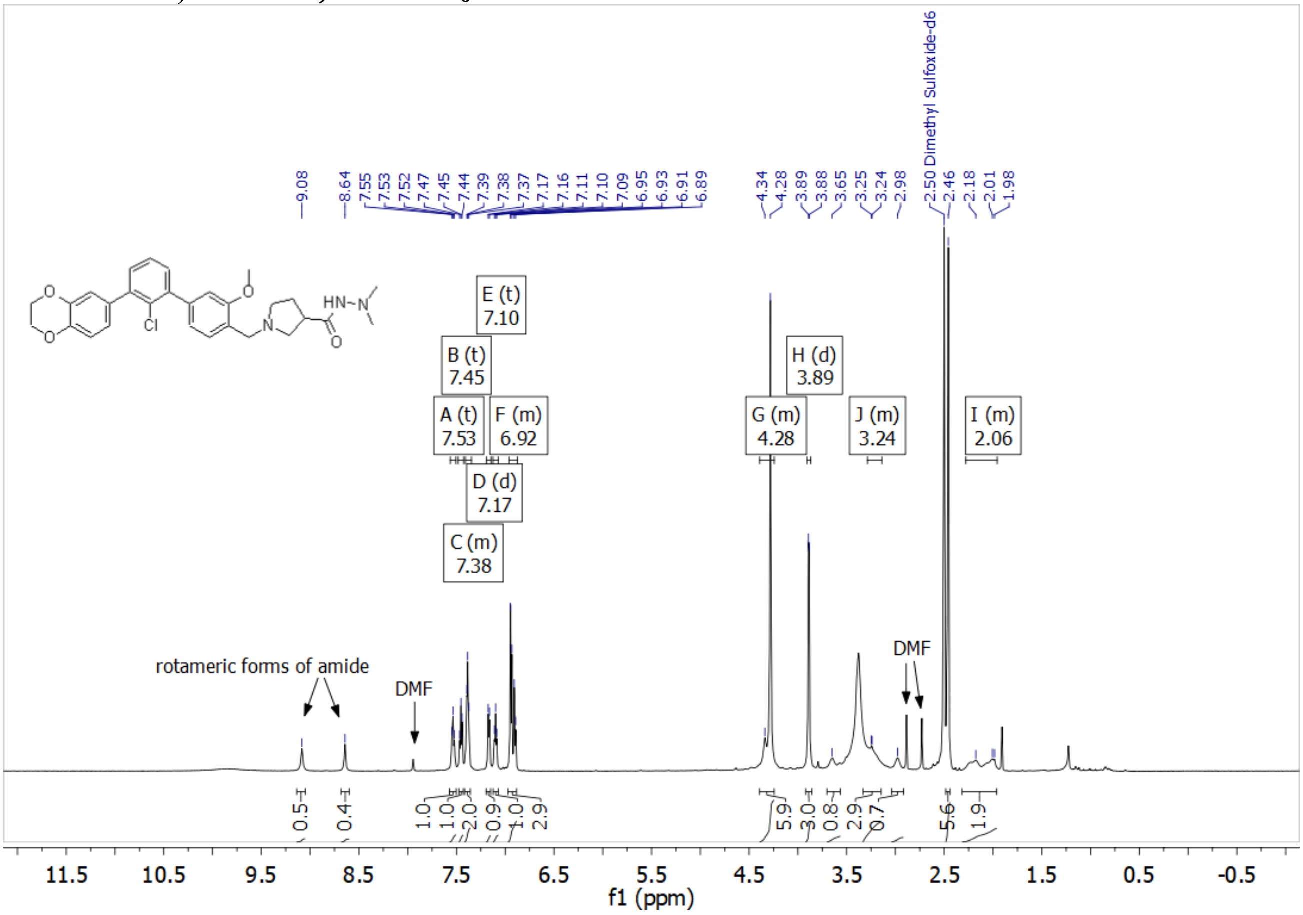

2d: ^{13}C NMR, 151 MHz, DMSO-d_6

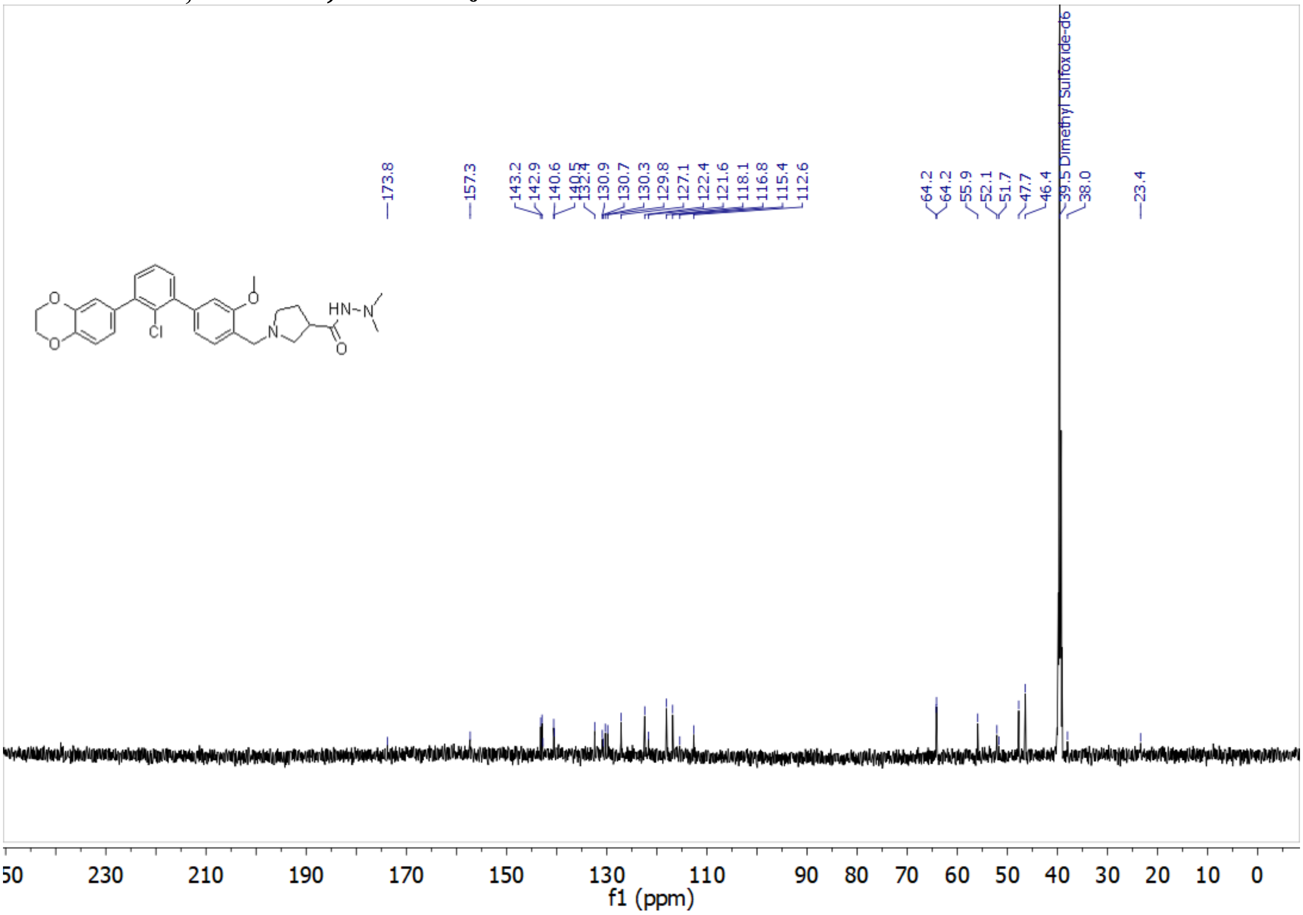

2e: ^{1}H NMR, 600 MHz, $CDCl_3$

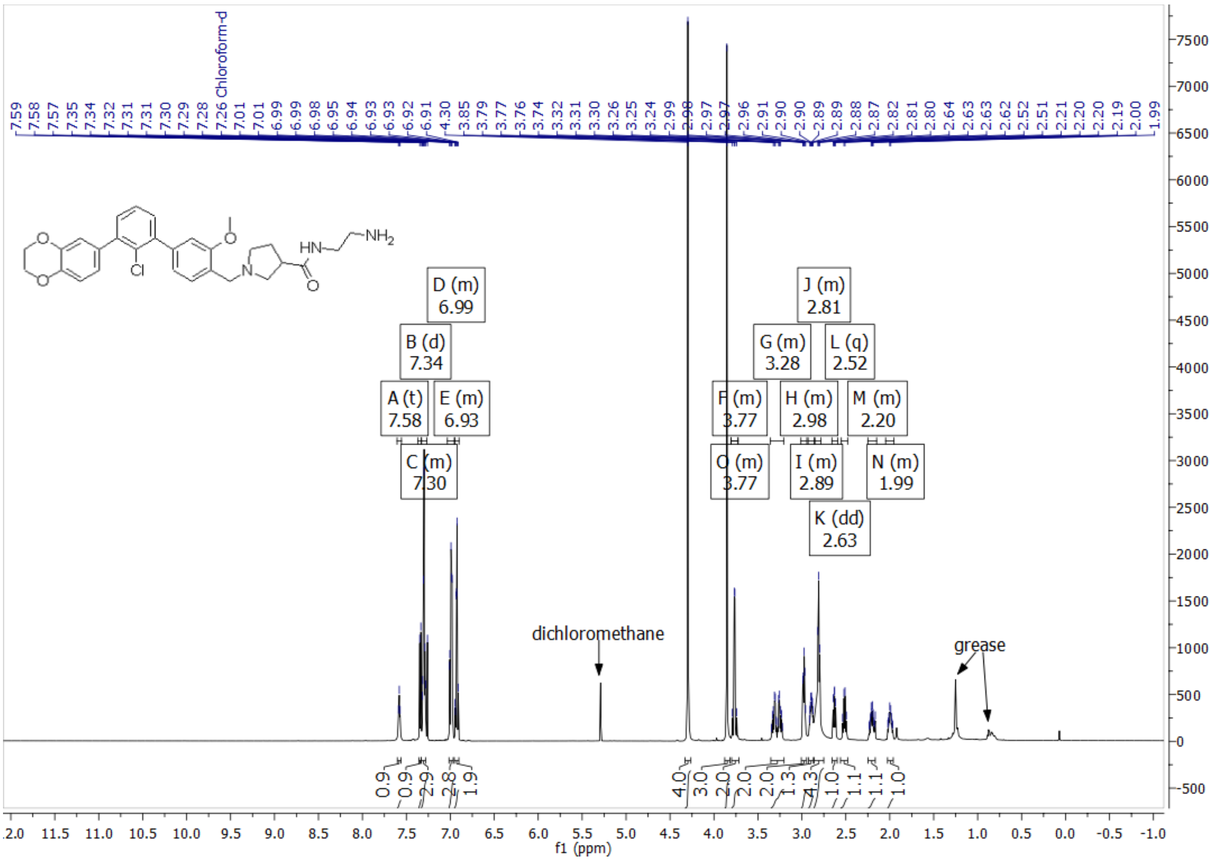

2e: ^{13}C NMR, 151 MHz, $CDCl_3$

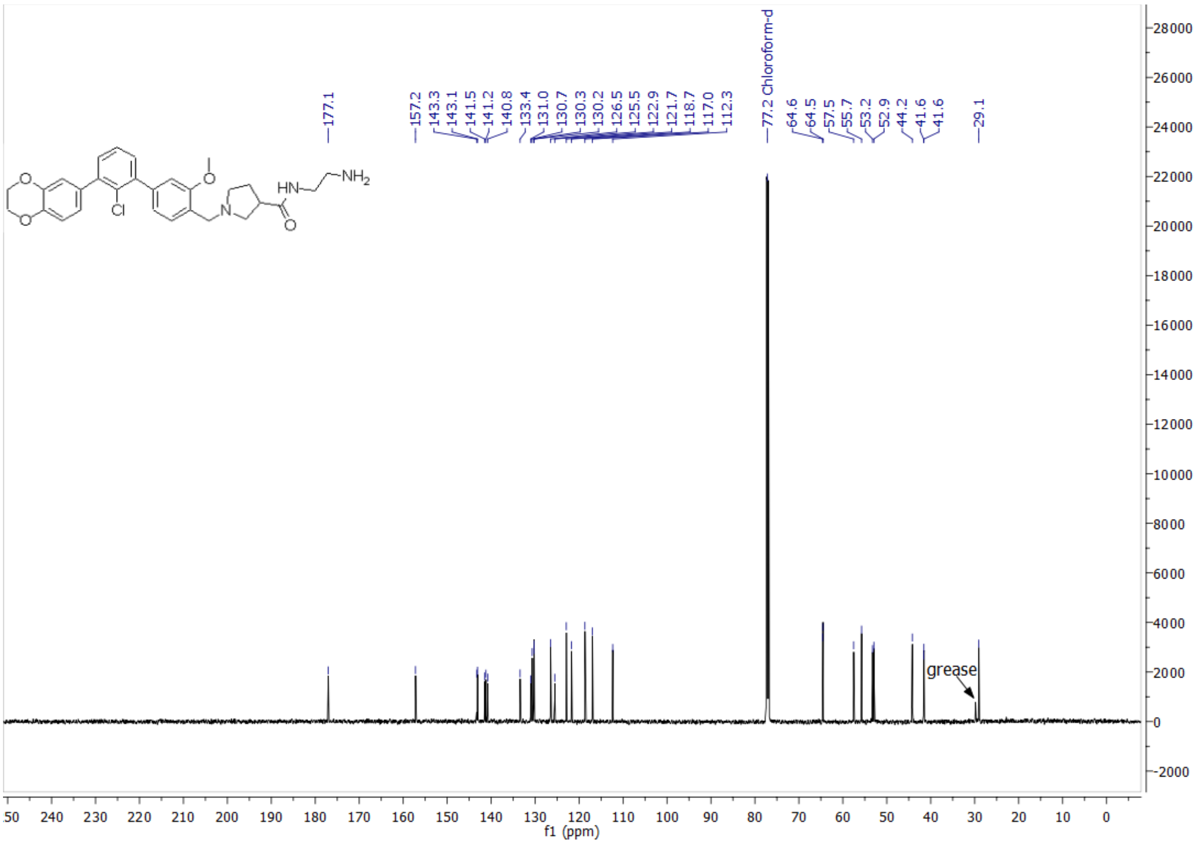

2f: ^{1}H NMR, 600 MHz, $CDCl_3$

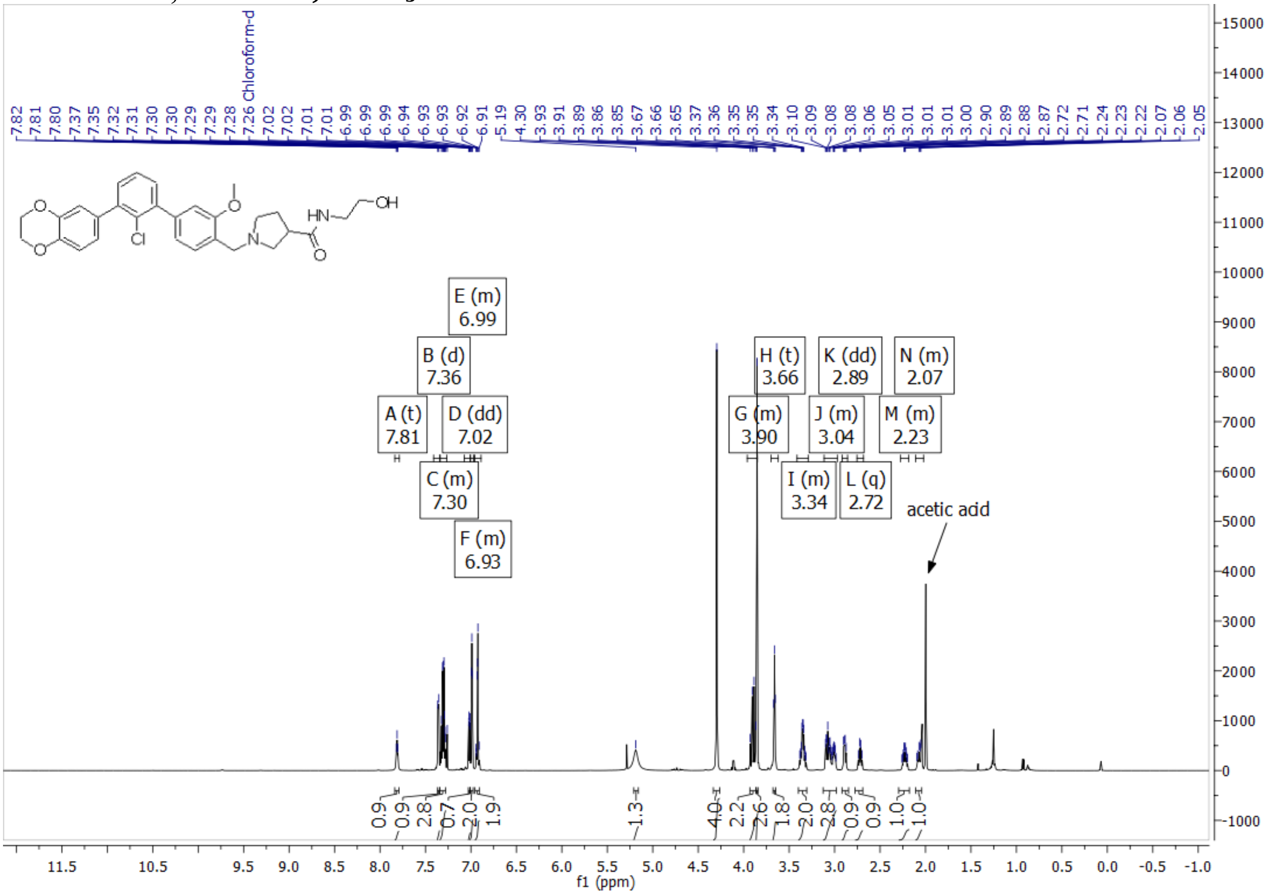

2f: ^{13}C NMR, 151 MHz, $CDCl_3$

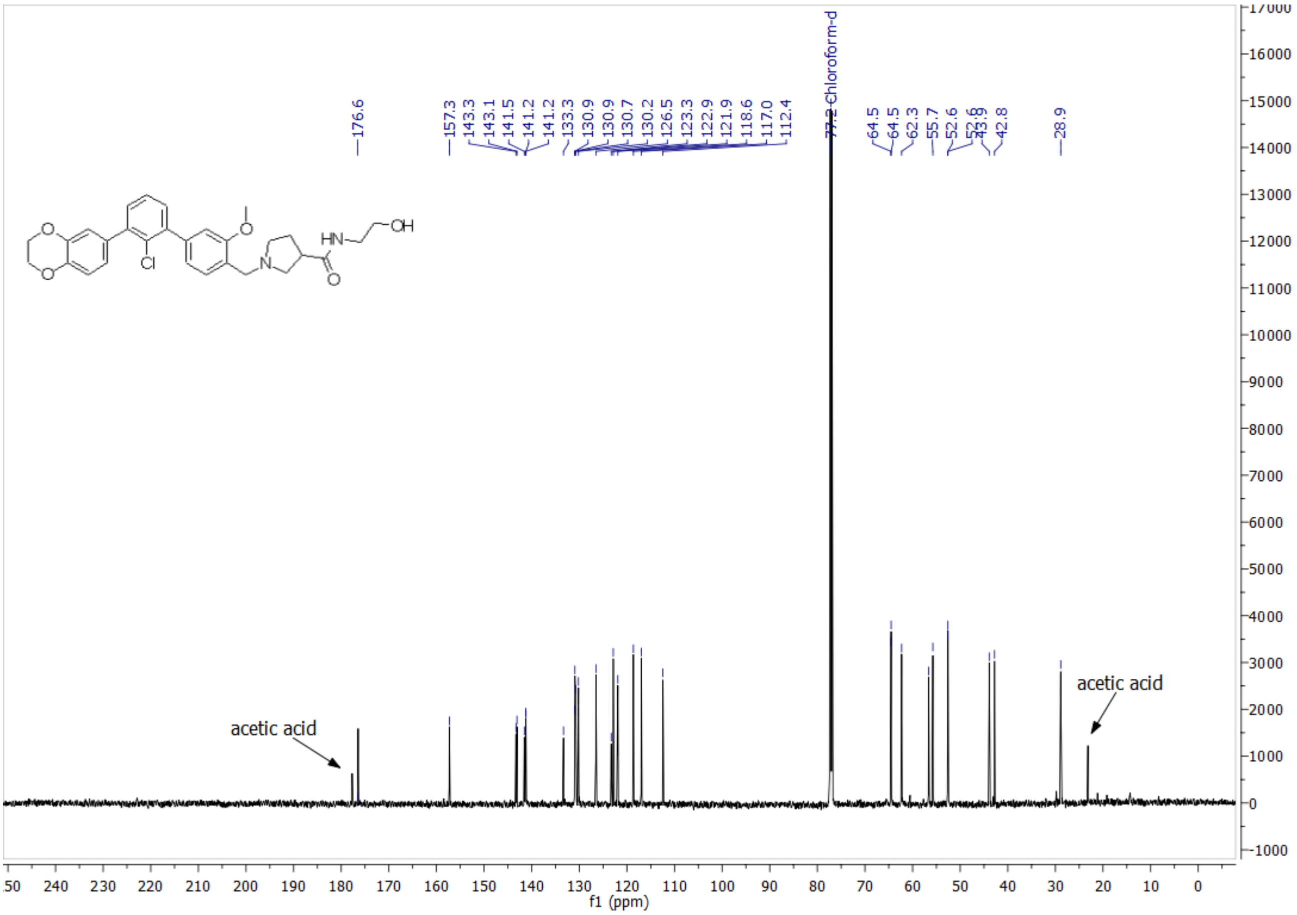

2g: ^{1}H NMR, 600 MHz, $CDCl_3$

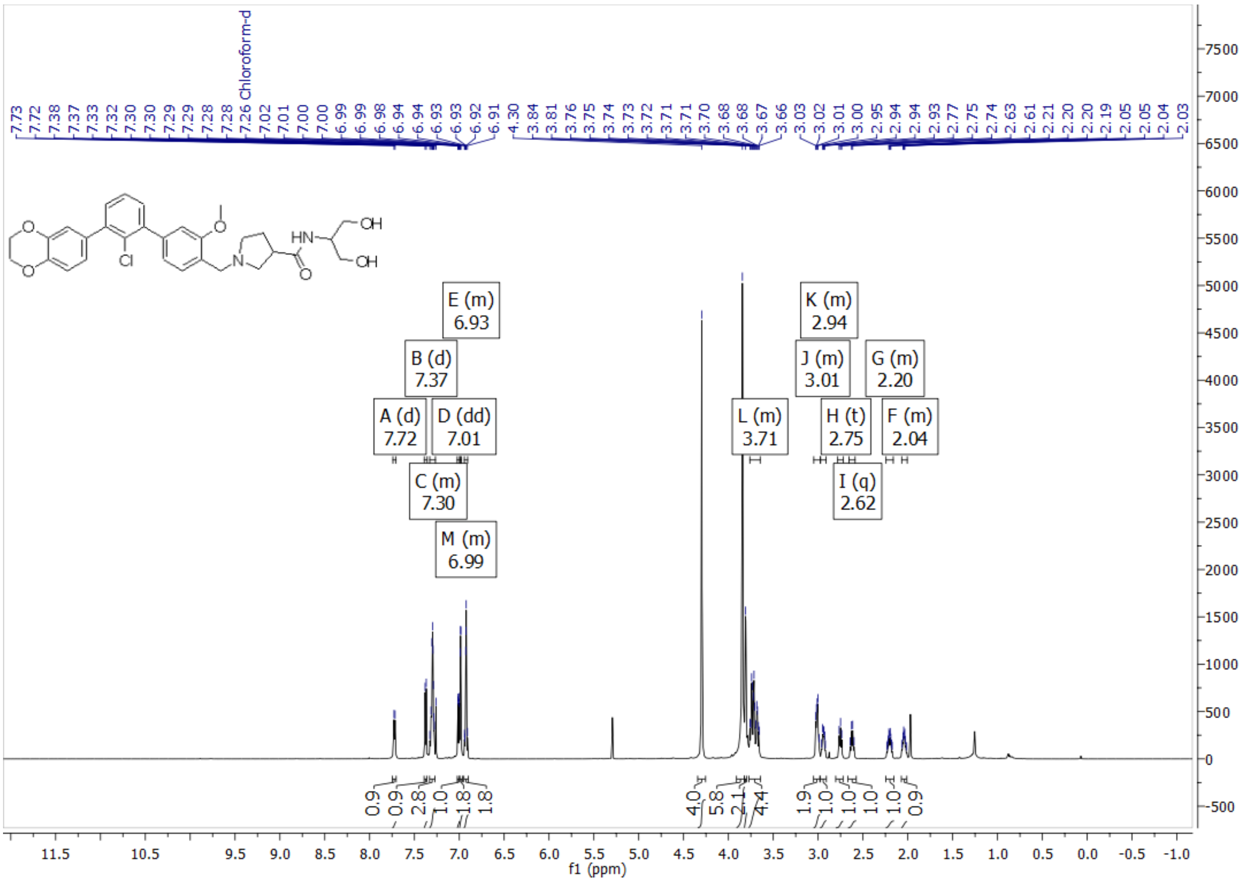

2g: ^{13}C NMR, 151 MHz, $CDCl_3$

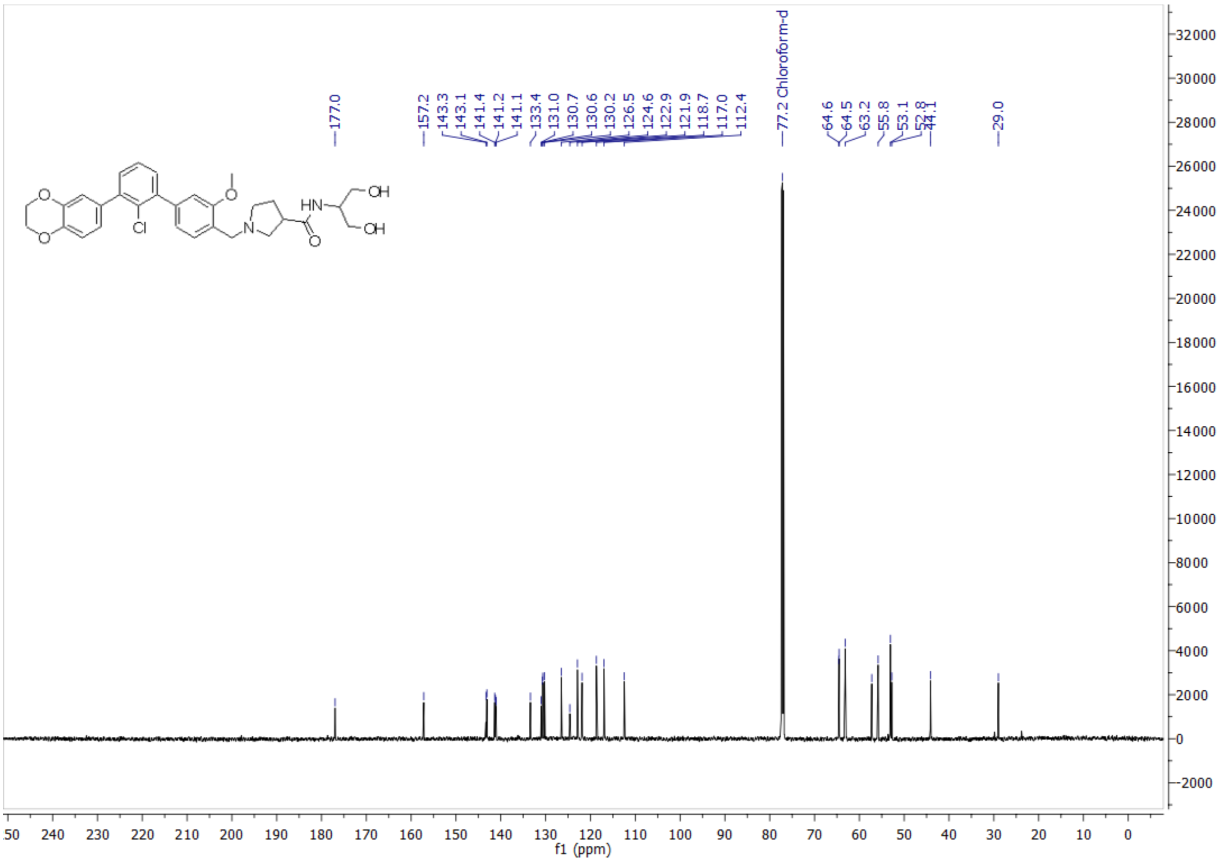

2h: ^{1}H NMR, 600 MHz, $CDCl_3$

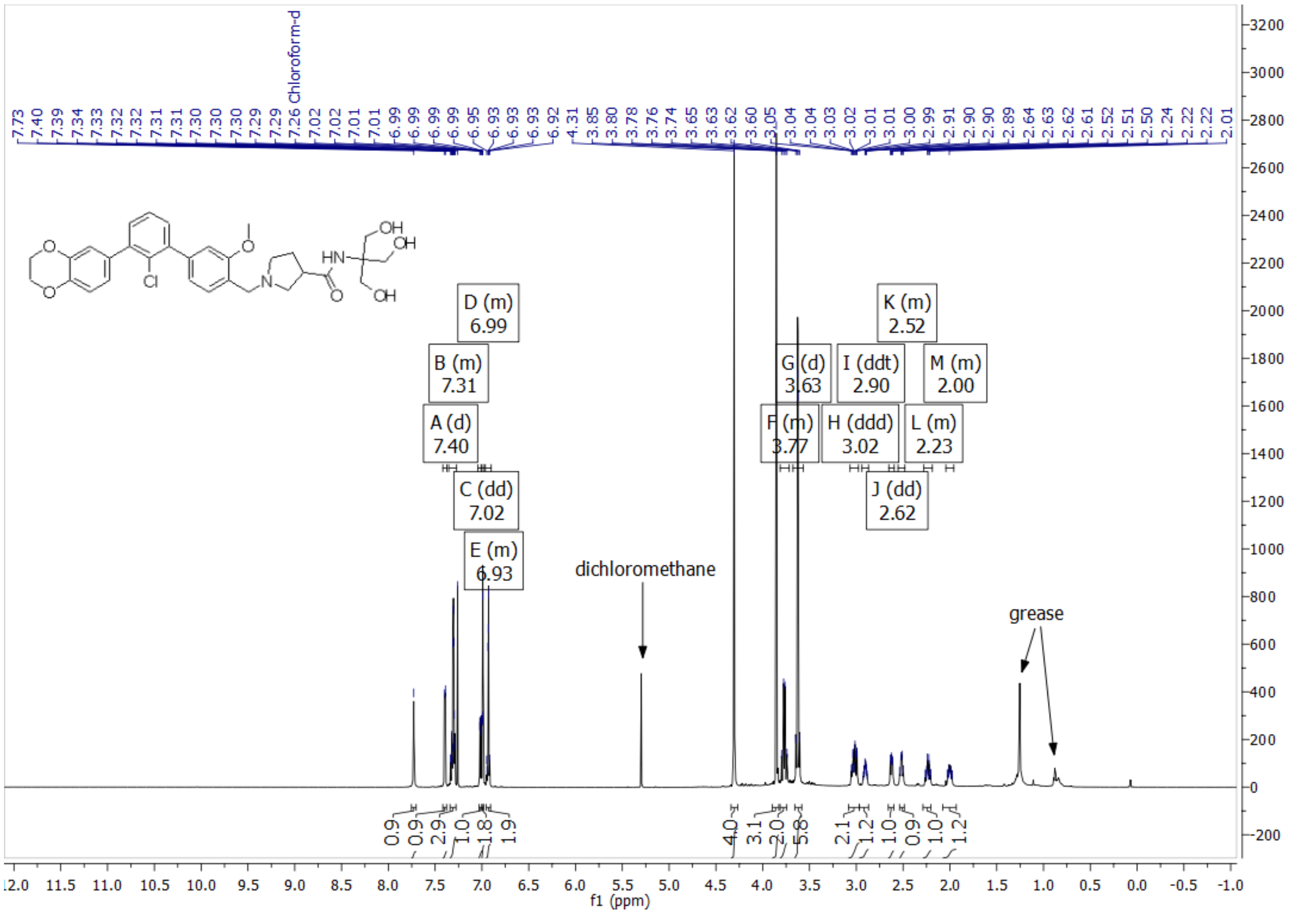

2h: ^{13}C NMR, 151 MHz, $CDCl_3$

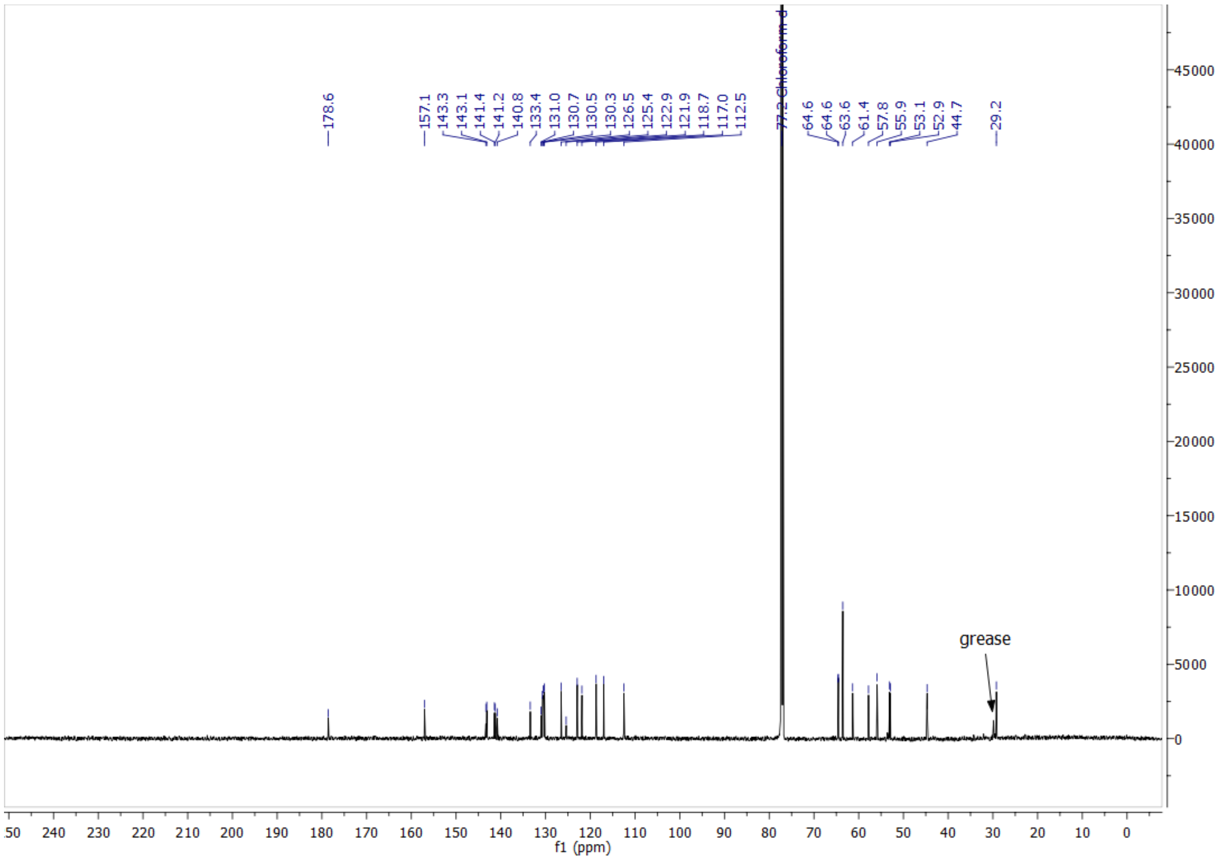

3a: ^{1}H NMR, 600 MHz, MeOD-d$_4$

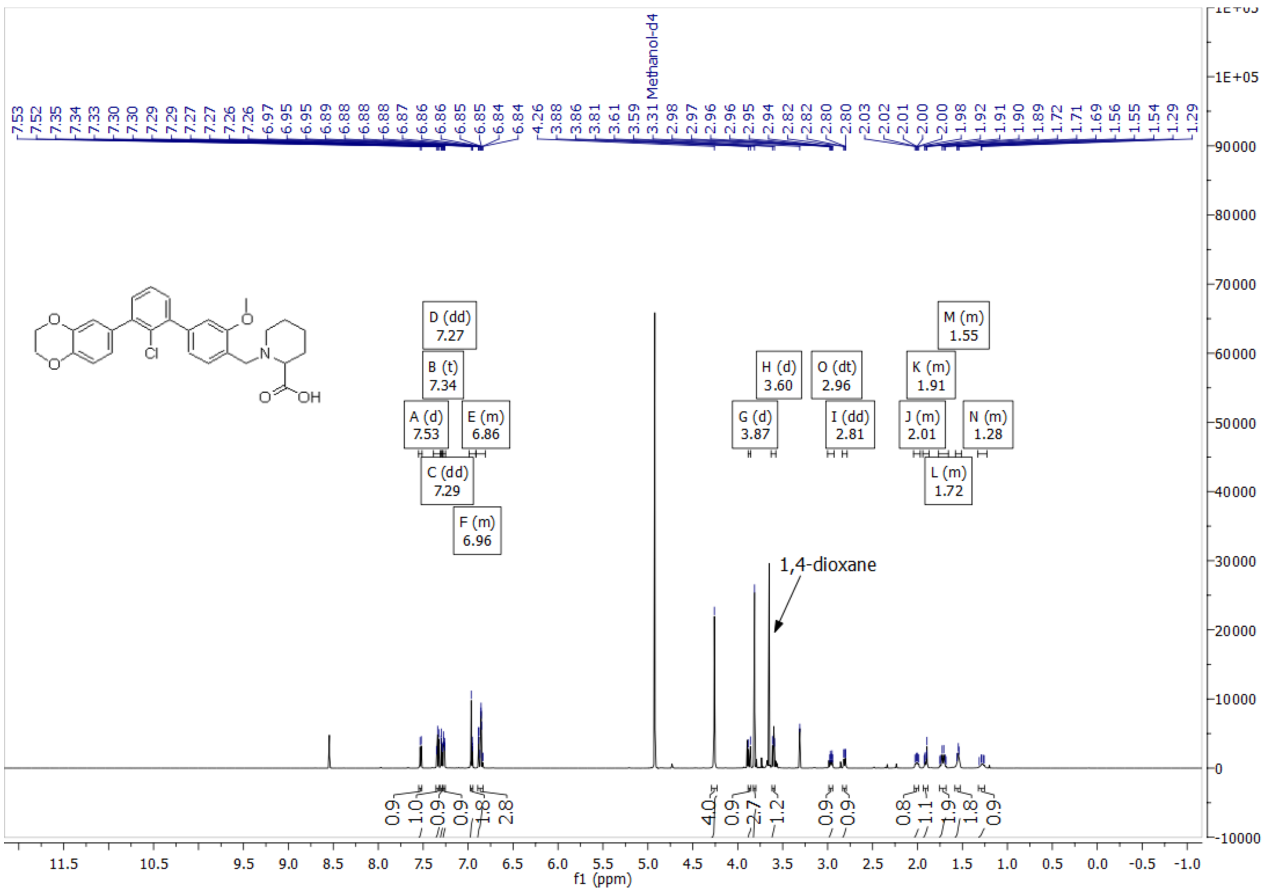

3a: ^{13}C NMR, 151 MHz, MeOD-d$_4$

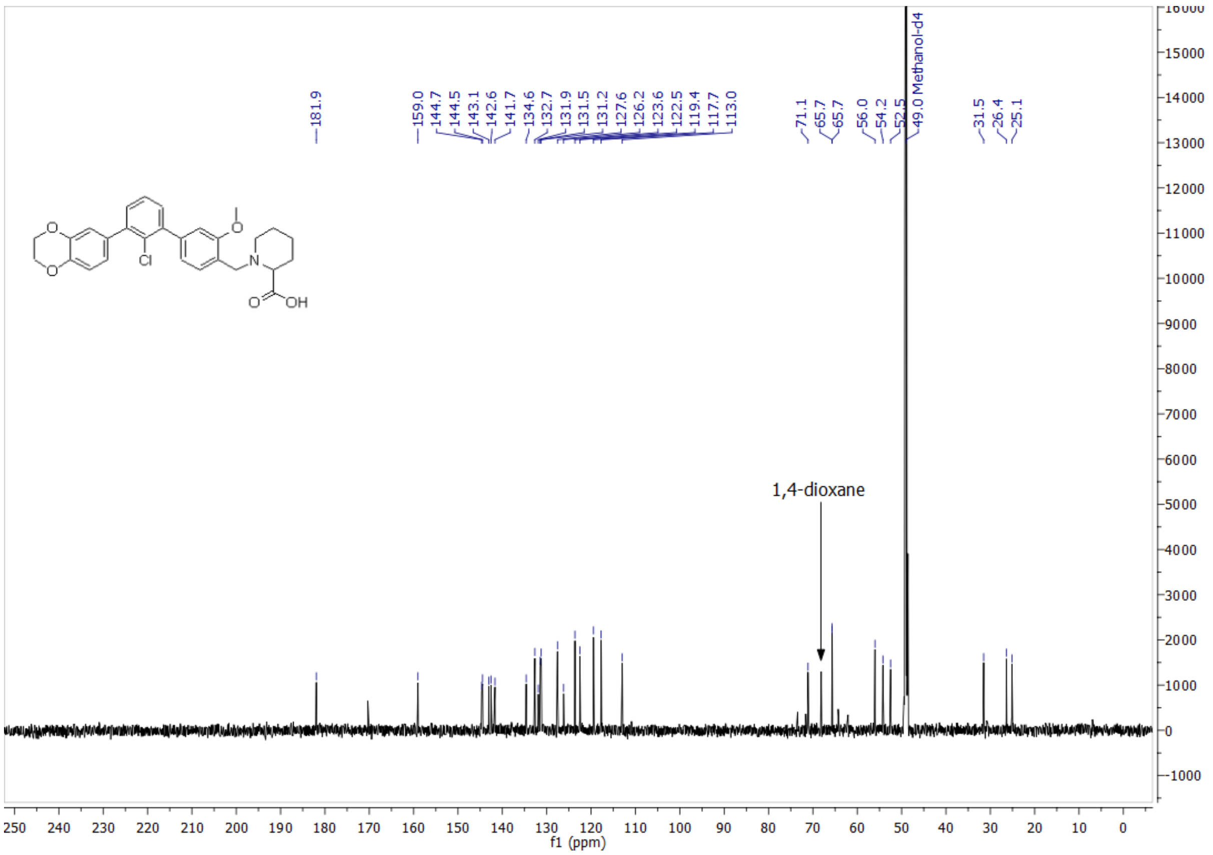

3c: ^{1}H NMR, 600 MHz, $CDCl_3$

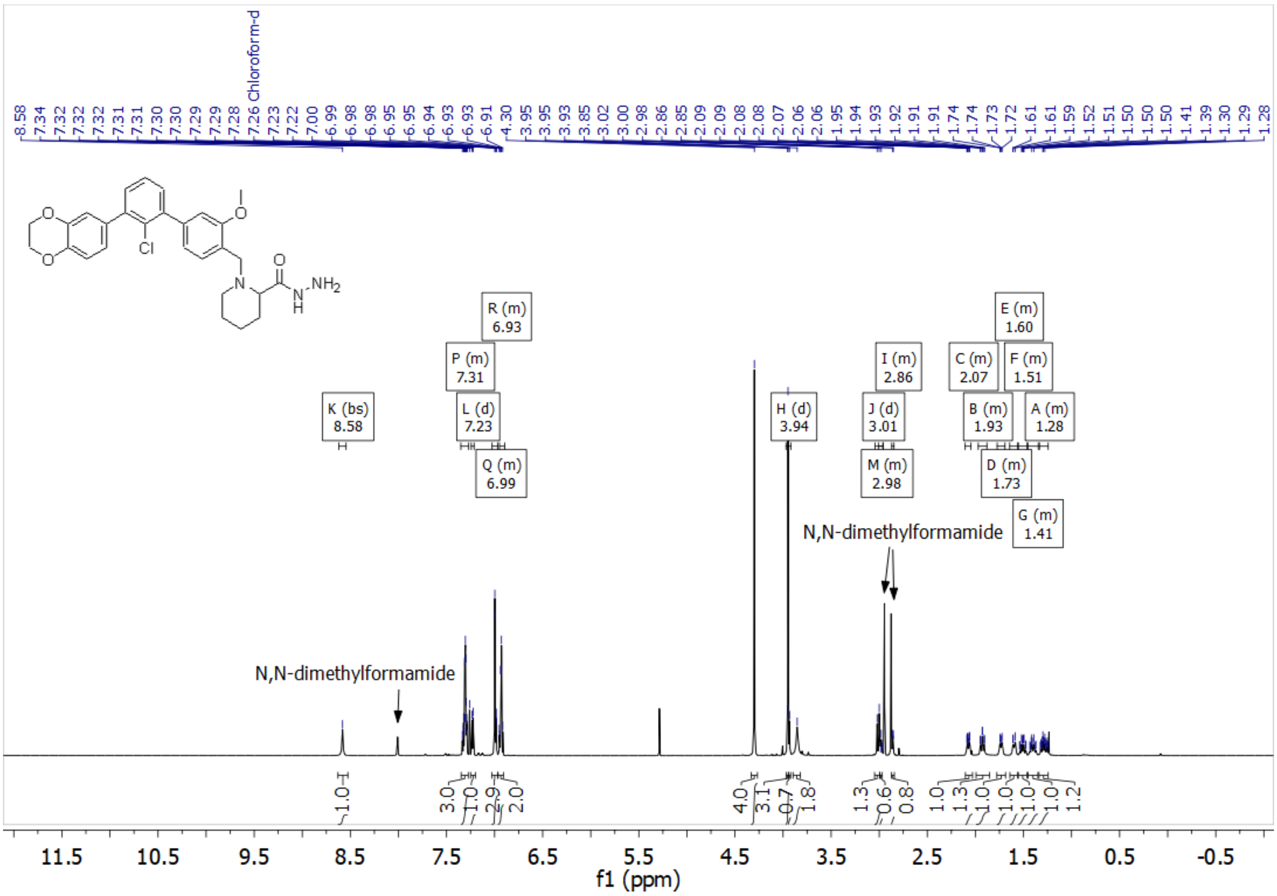

3c: ^{13}C NMR, 151 MHz, DMSO-d_6

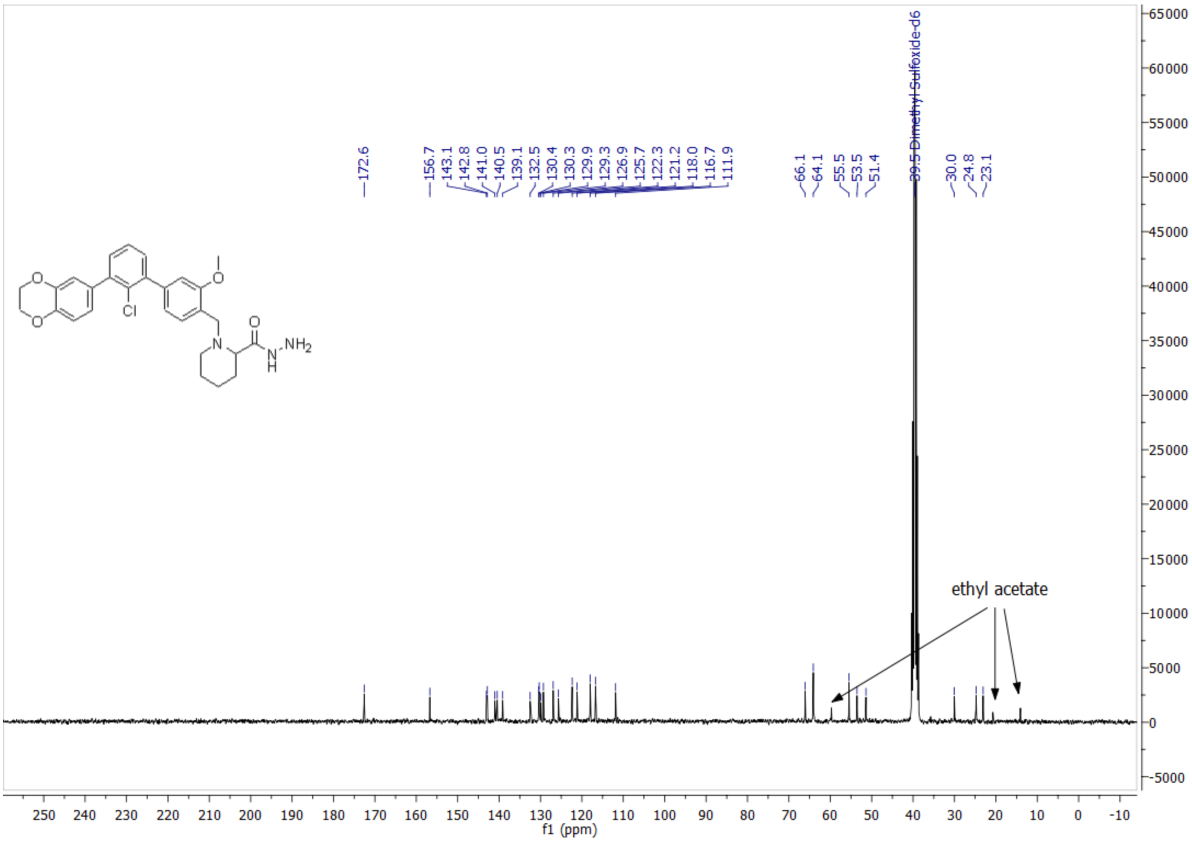

3d: ^{1}H NMR, 600 MHz, $CDCl_3$

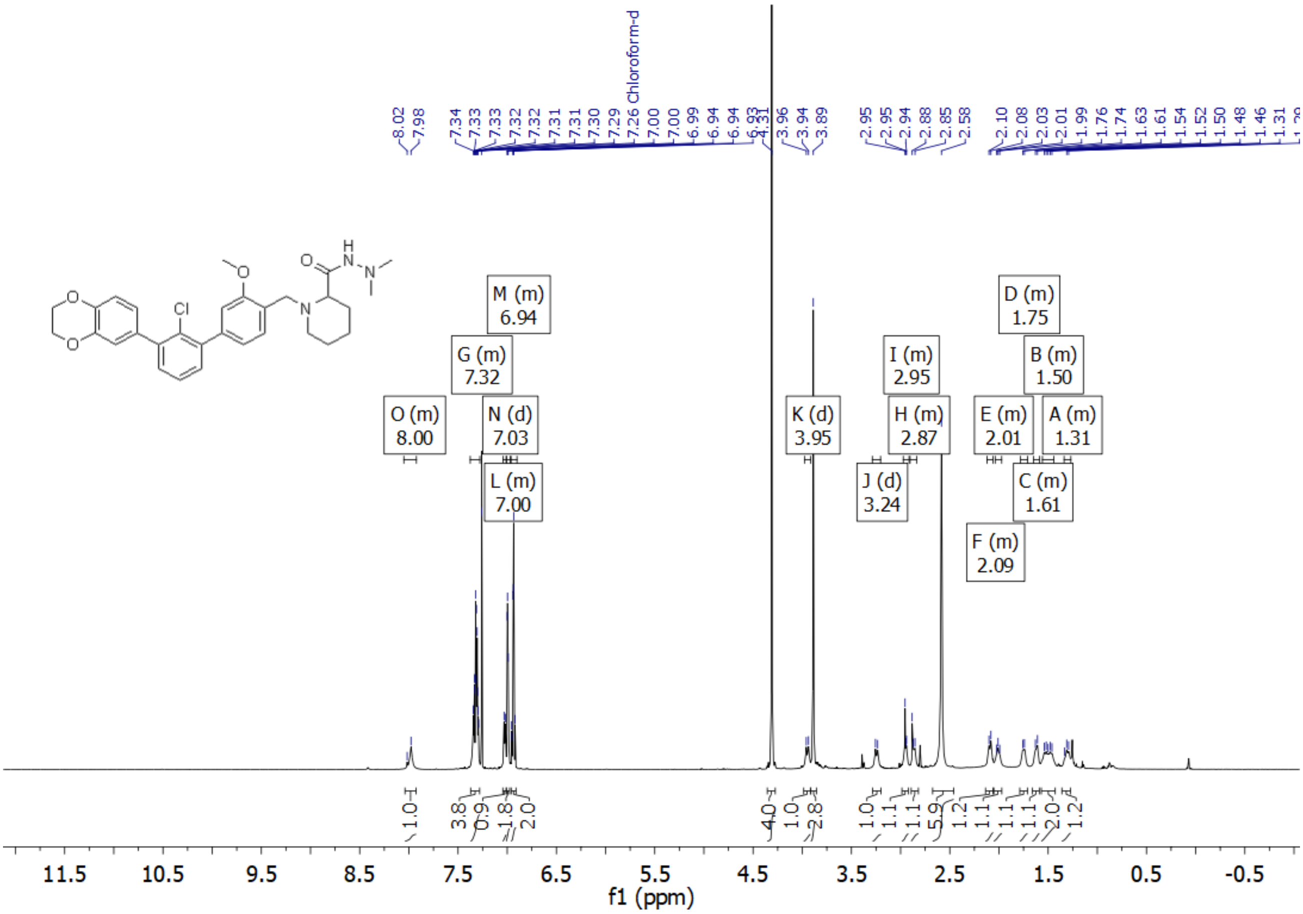

3d: ^{13}C NMR, 151 MHz, $CDCl_3$

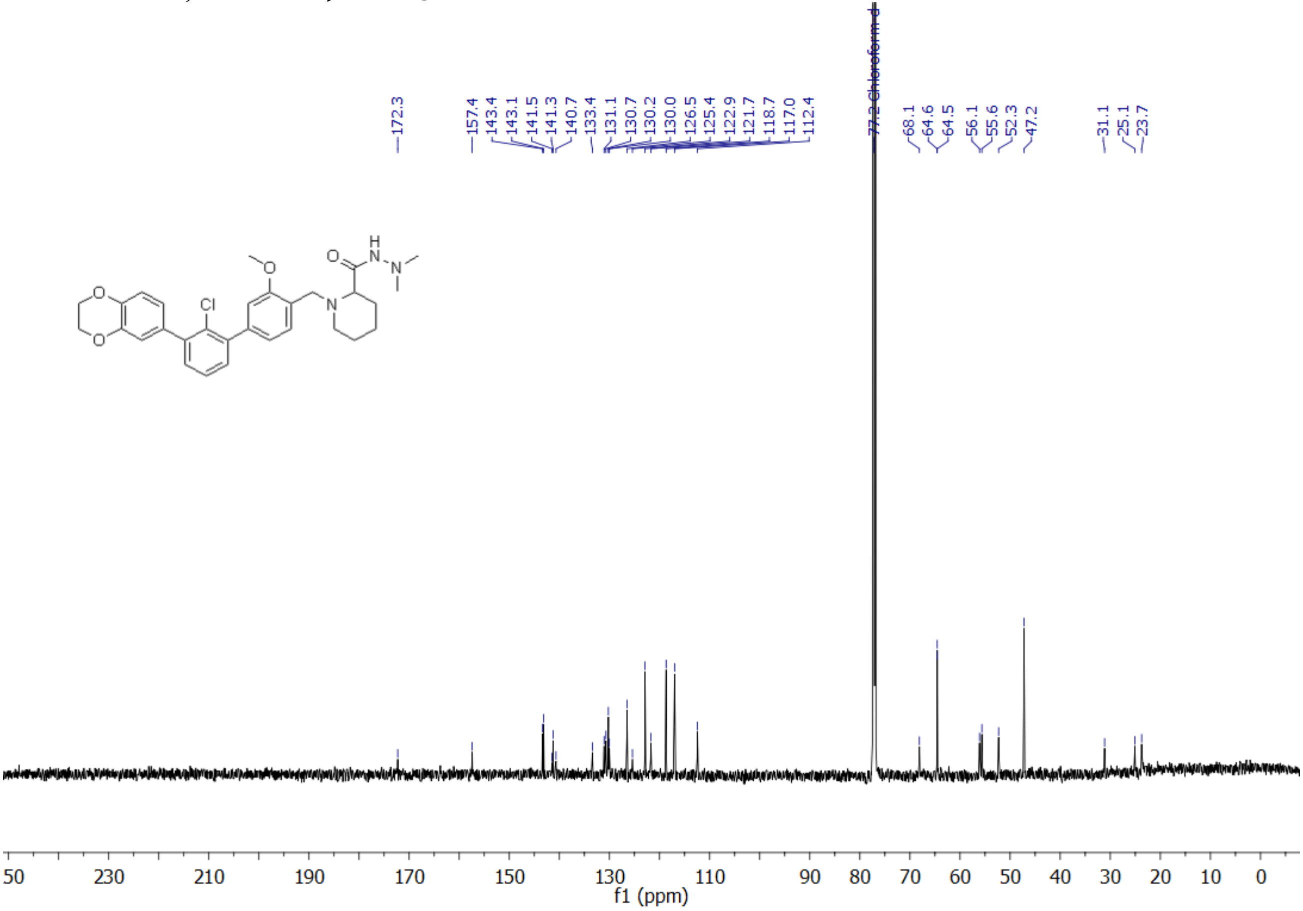

3f: ^{1}H NMR, 600 MHz, $CDCl_3$

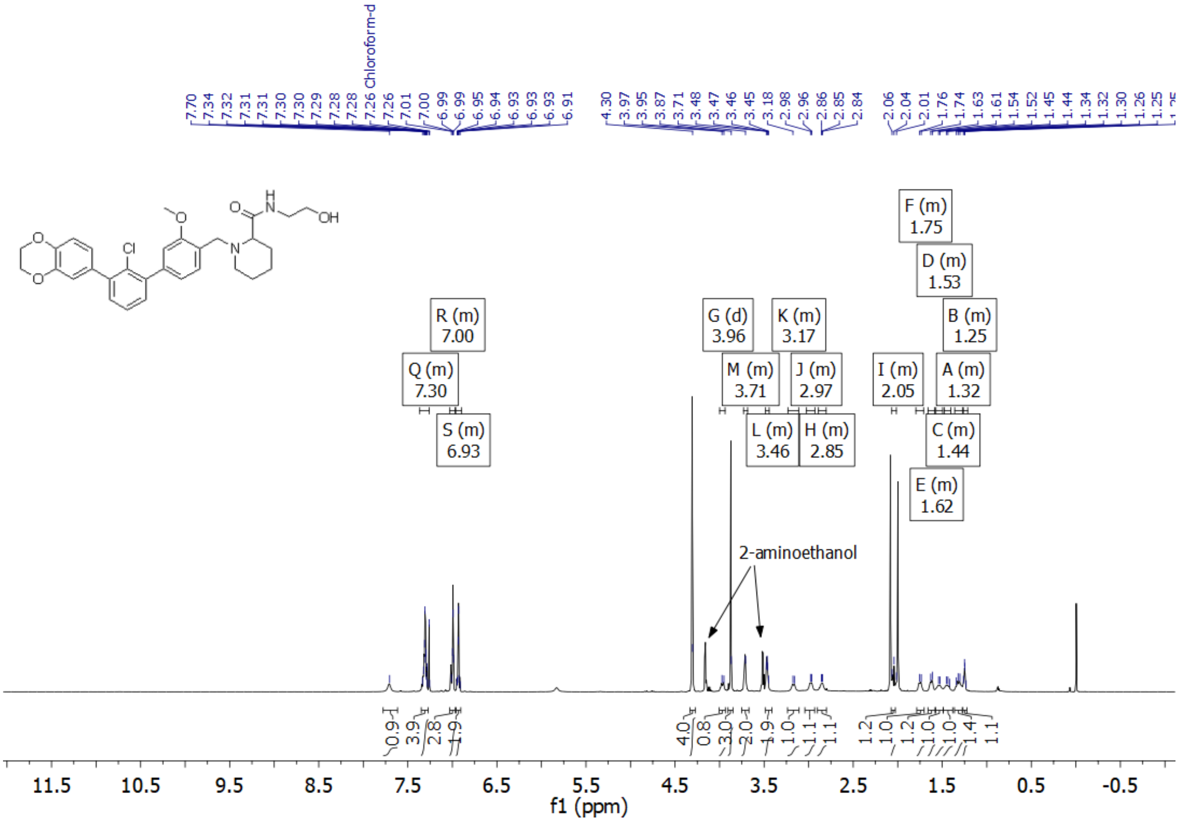

3f: ^{13}C NMR, 151 MHz, $CDCl_3$

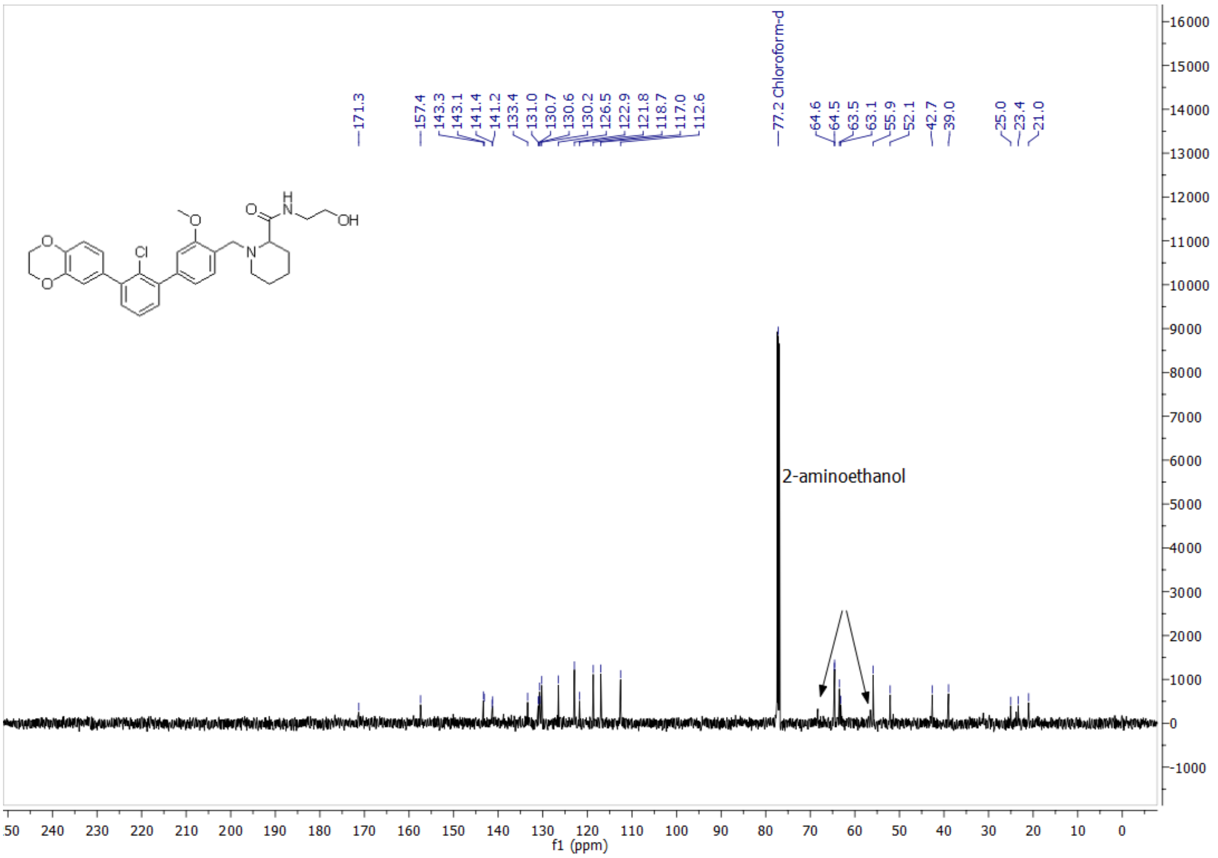

3g: ^{1}H NMR, 600 MHz, DMSO-d_6

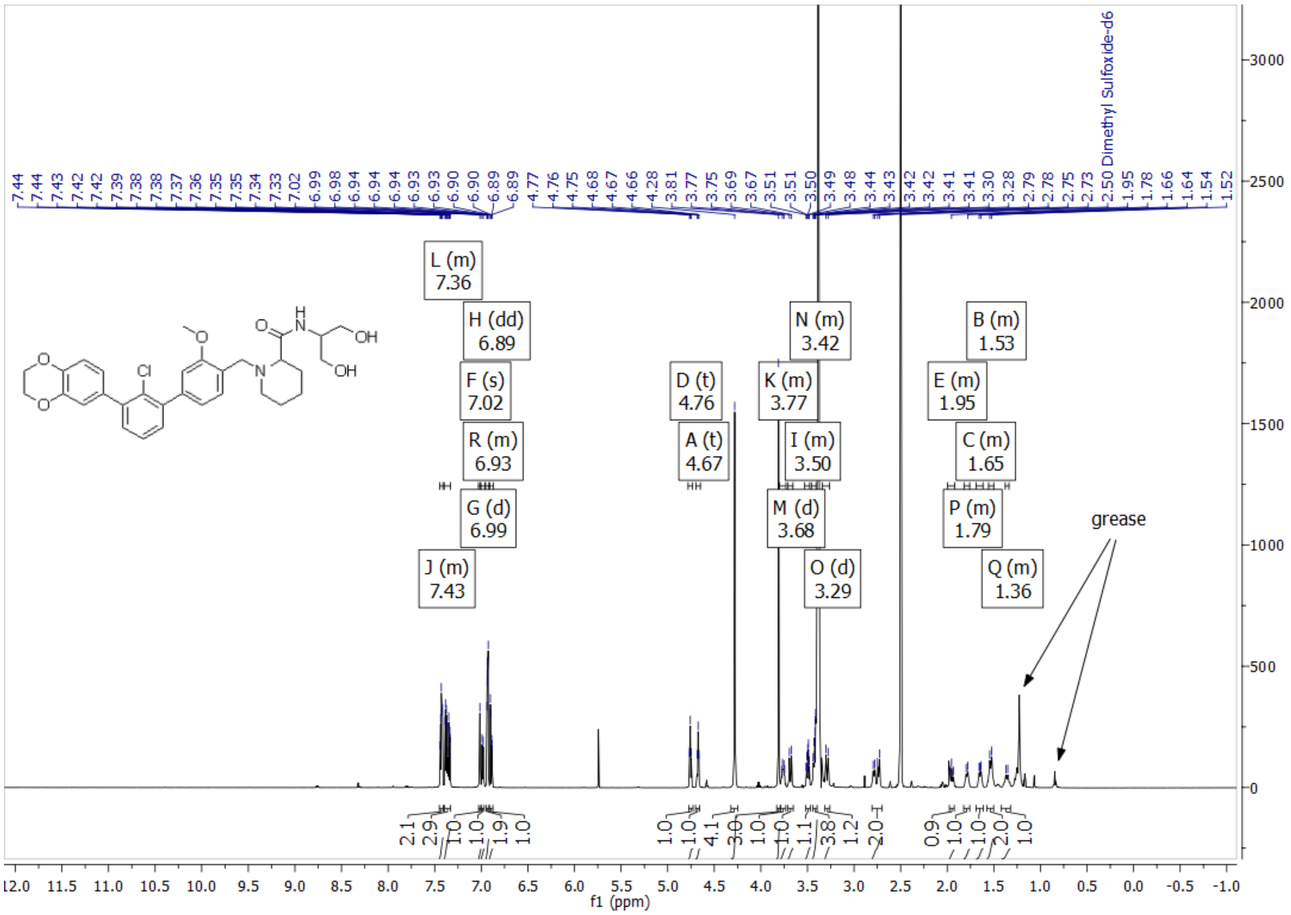

3g: ^{13}C NMR, 151 MHz, DMSO-d_6

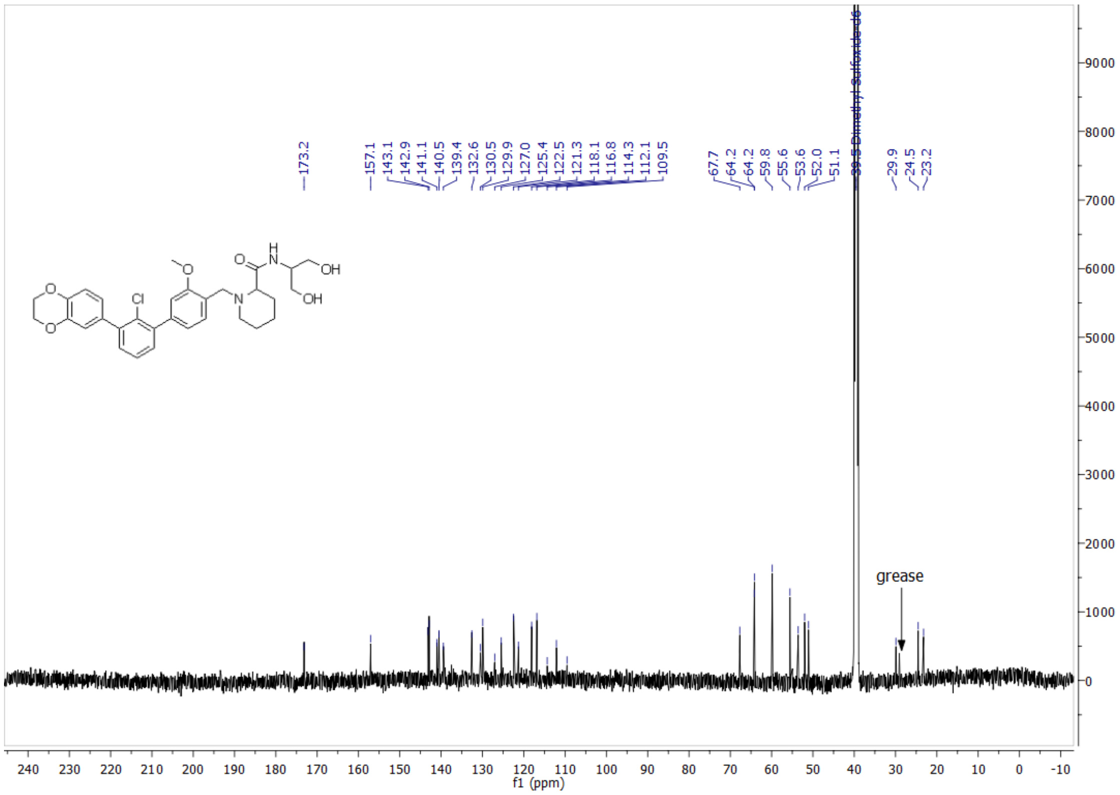

3h: ^{1}H NMR, 600 MHz, CDCl$_3$

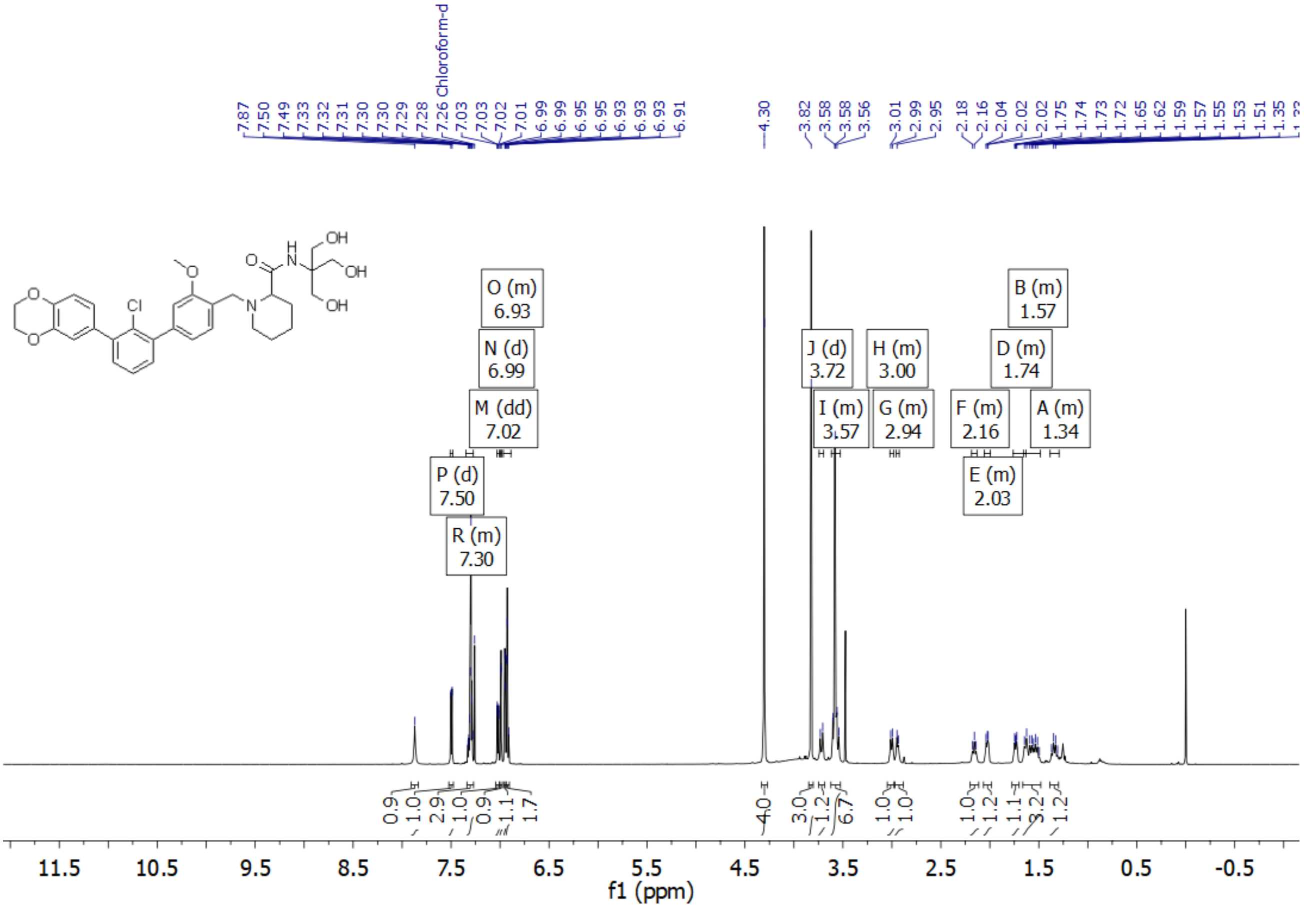

3h: ^{13}C NMR, 151 MHz, CDCl$_3$

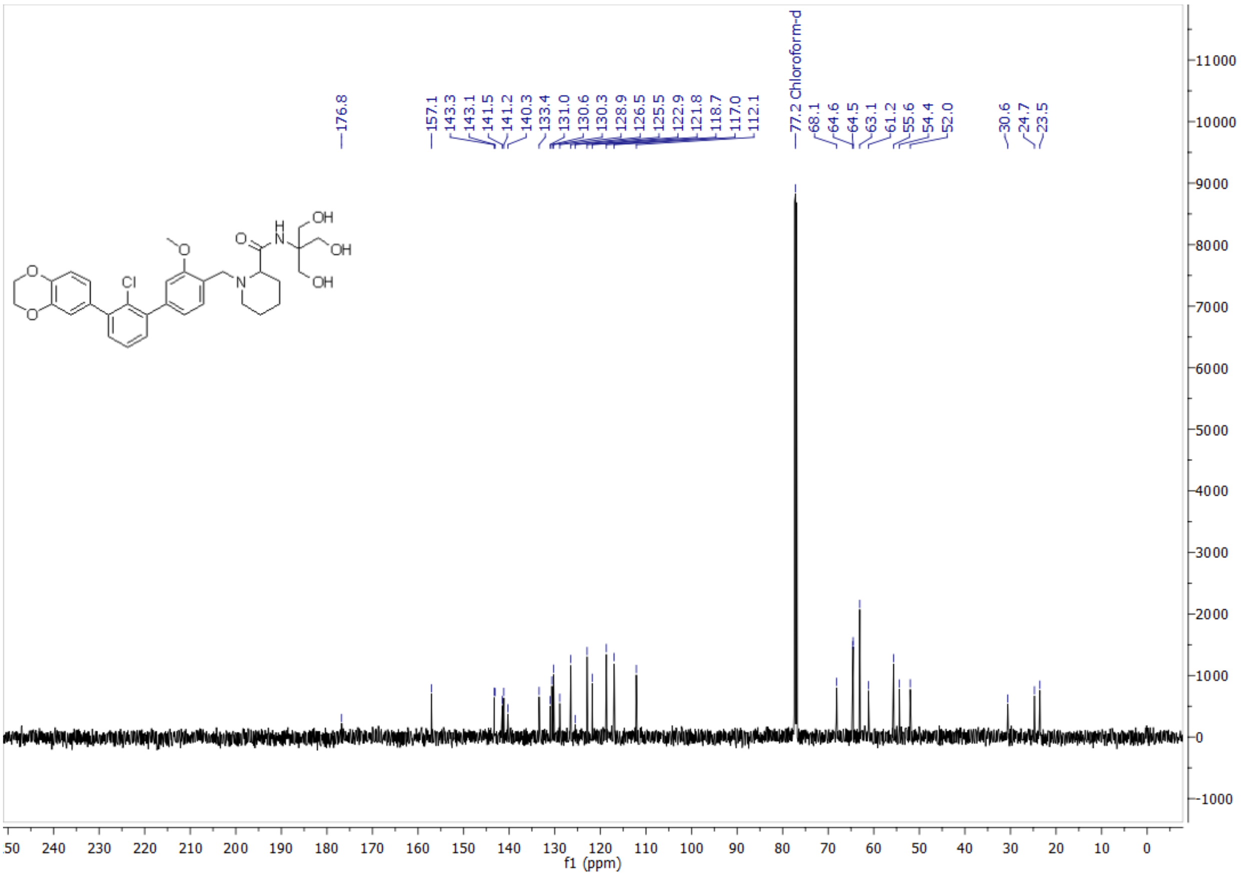

4a: ^{1}H NMR, 600 MHz, $CDCl_3$

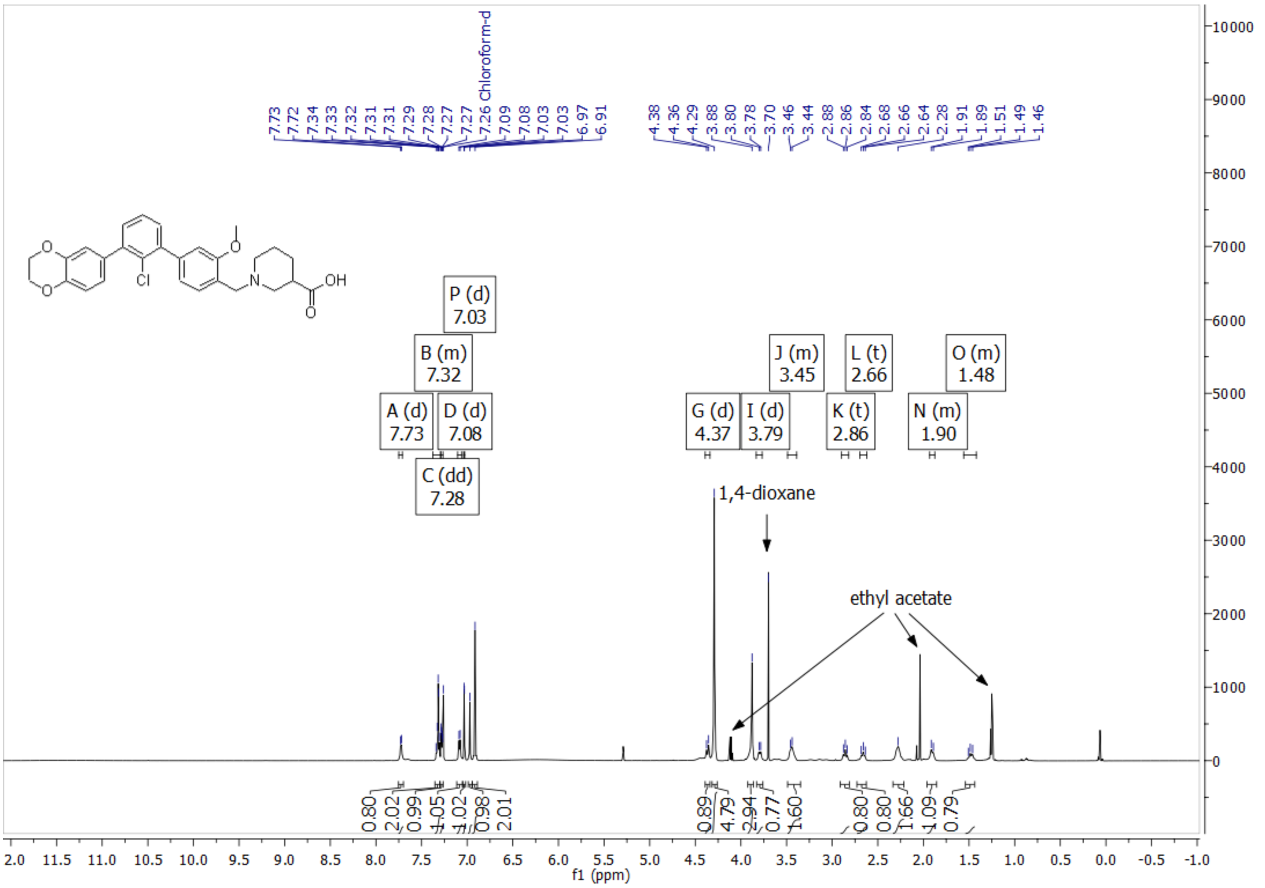

4a: ^{13}C NMR, 151 MHz, $CDCl_3$

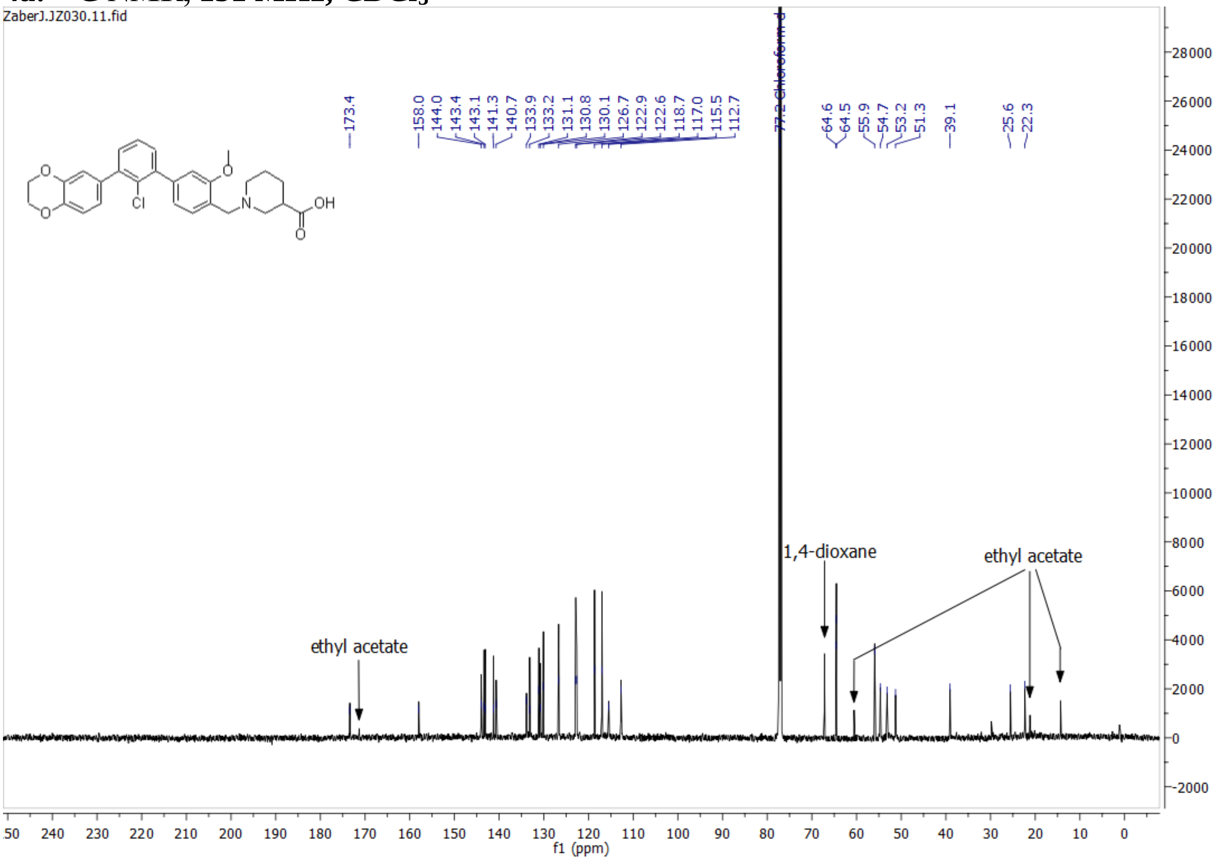

4b: ^{1}H NMR, 600 MHz, DMSO-d$_6$

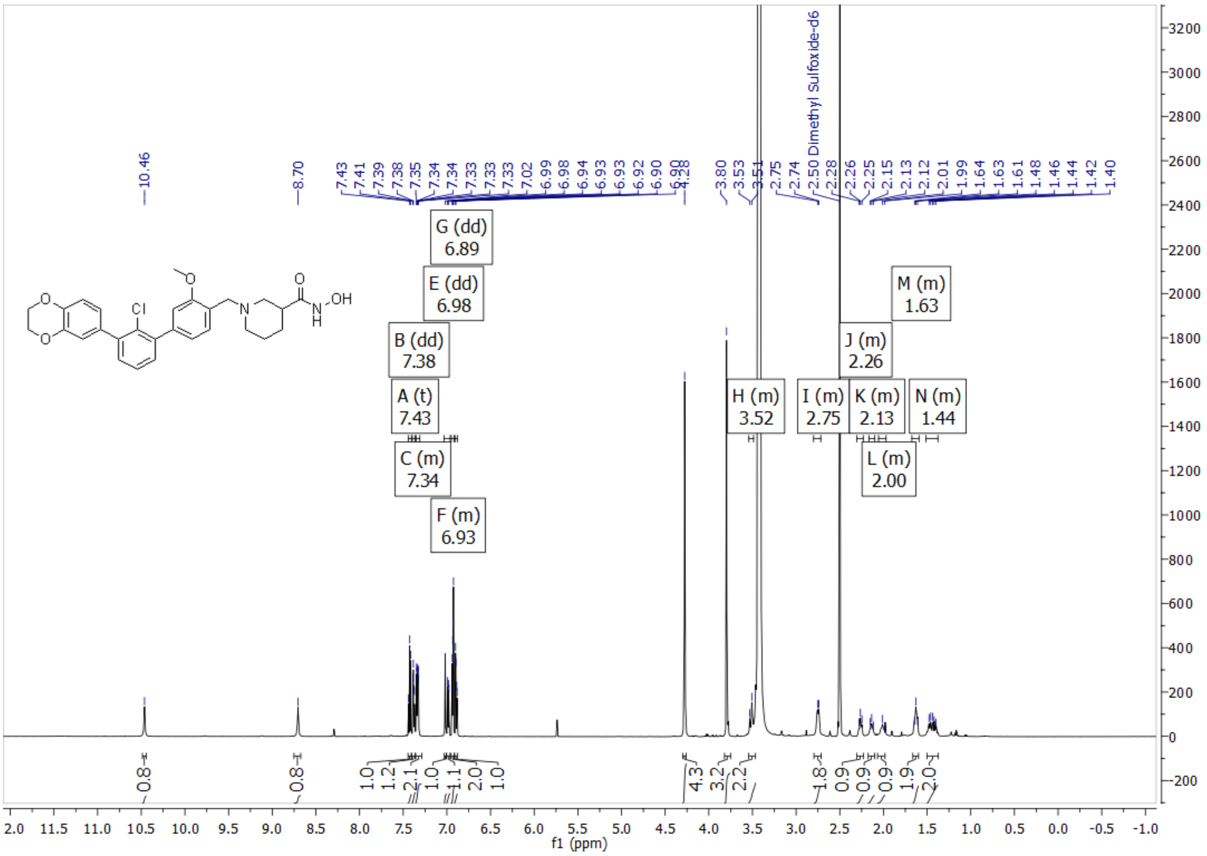

4b: ^{13}C NMR, 151 MHz, DMSO-d$_6$

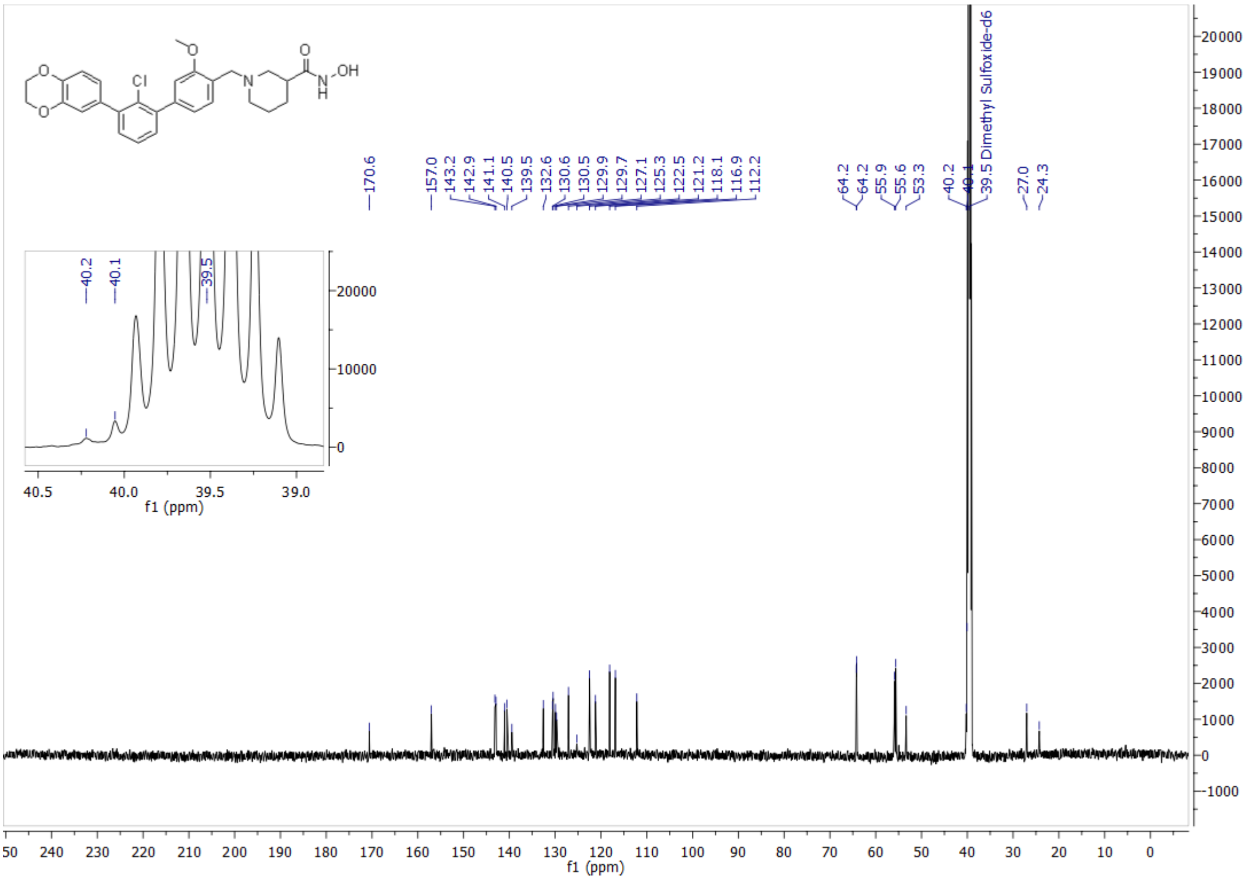

4c: ^{1}H NMR, 600 MHz, DMSO-D$_6$

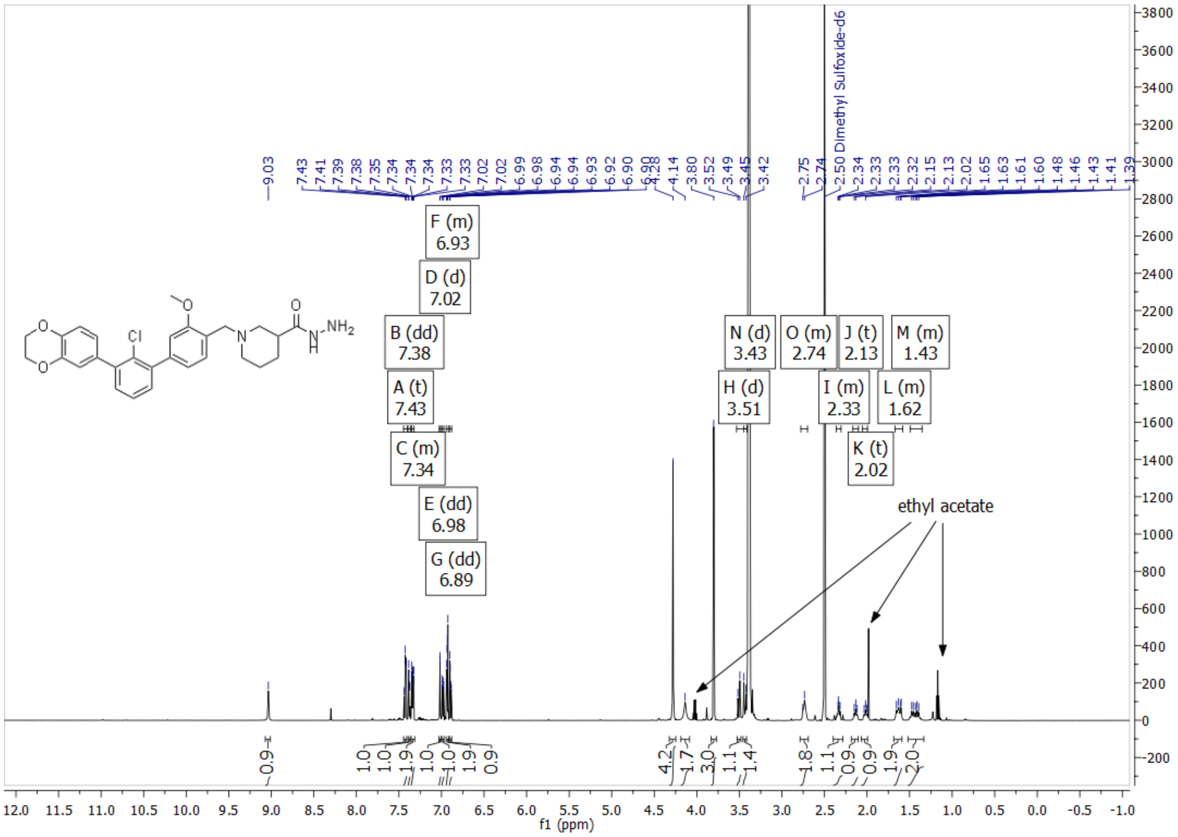

4c: ^{13}C NMR, 151 MHz, DMSO-d$_6$

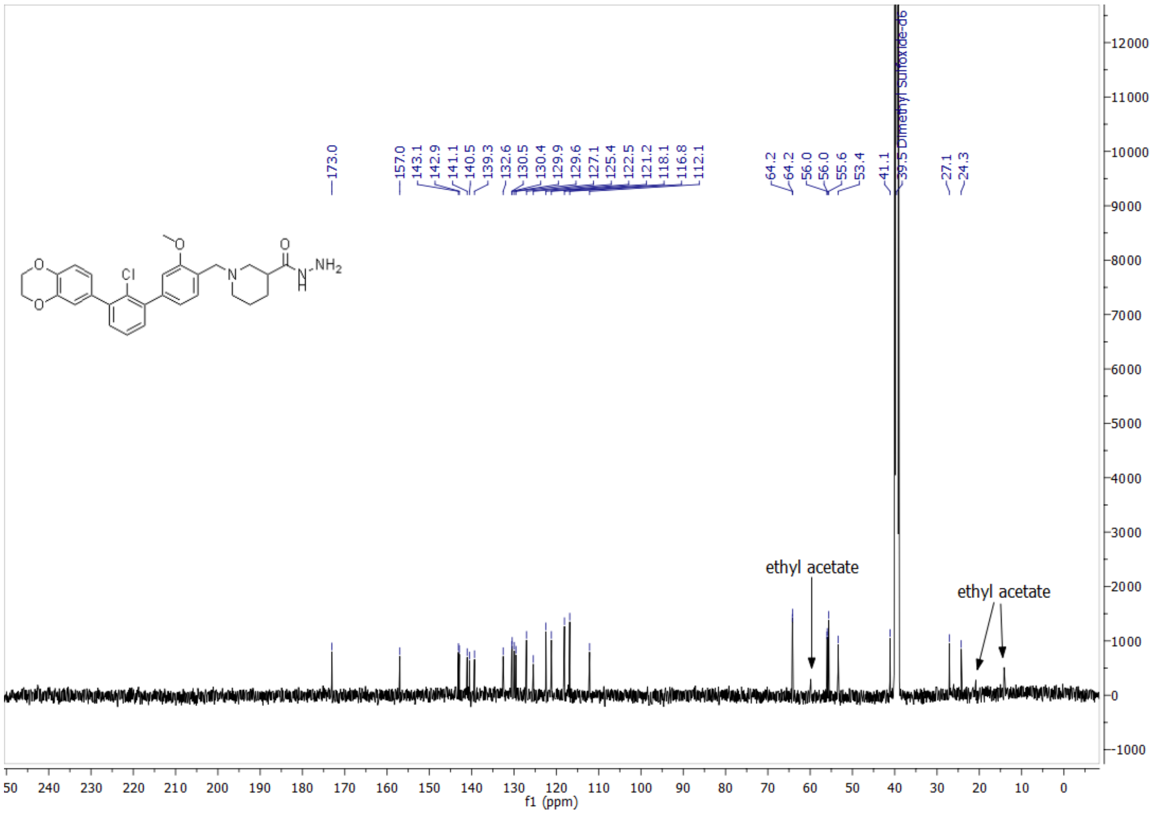

4d: ^{1}H NMR, 600 MHz, CDCl$_3$

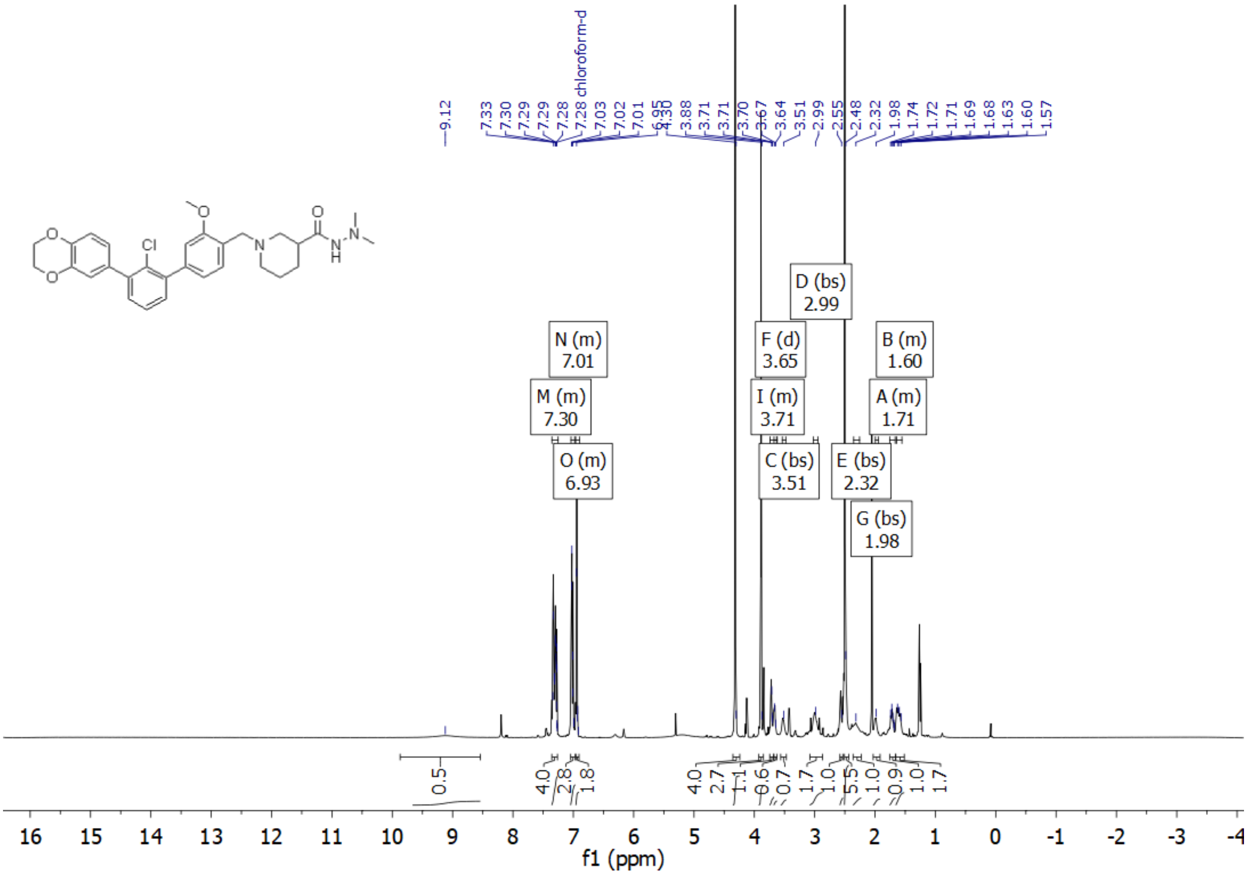

4d: ^{13}C NMR, 151 MHz, CDCl$_3$

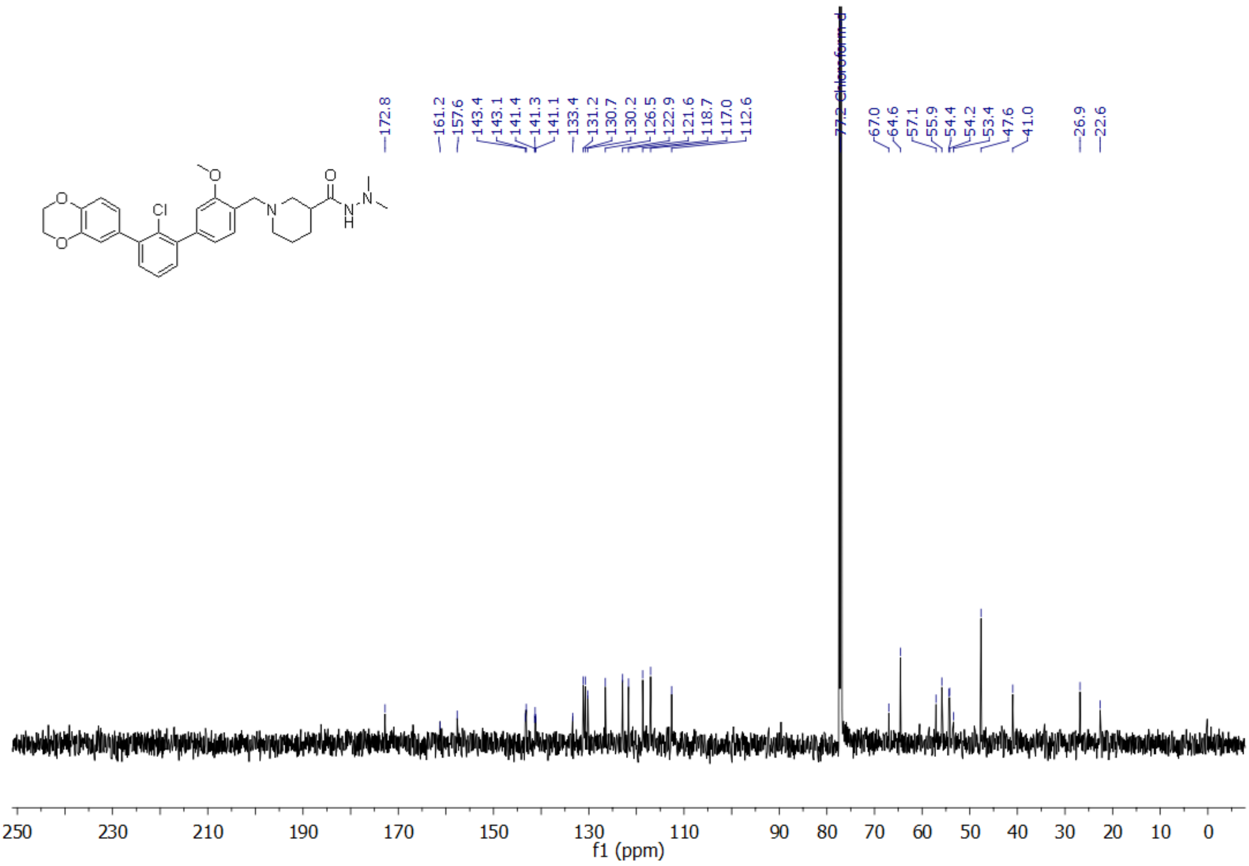

4f: ^{1}H NMR, 600 MHz, CDCl$_3$

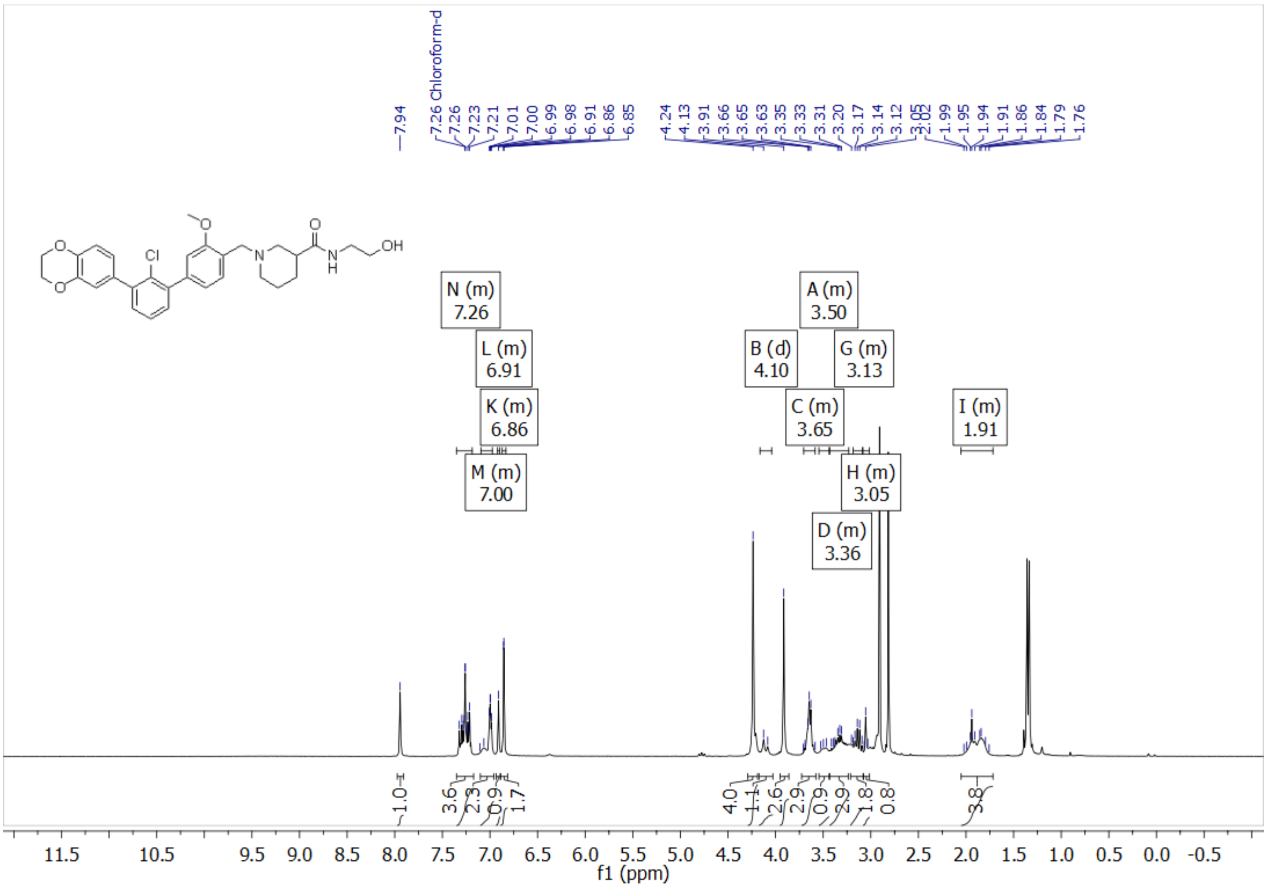

4f: ^{13}C NMR, 151 MHz, CDCl$_3$

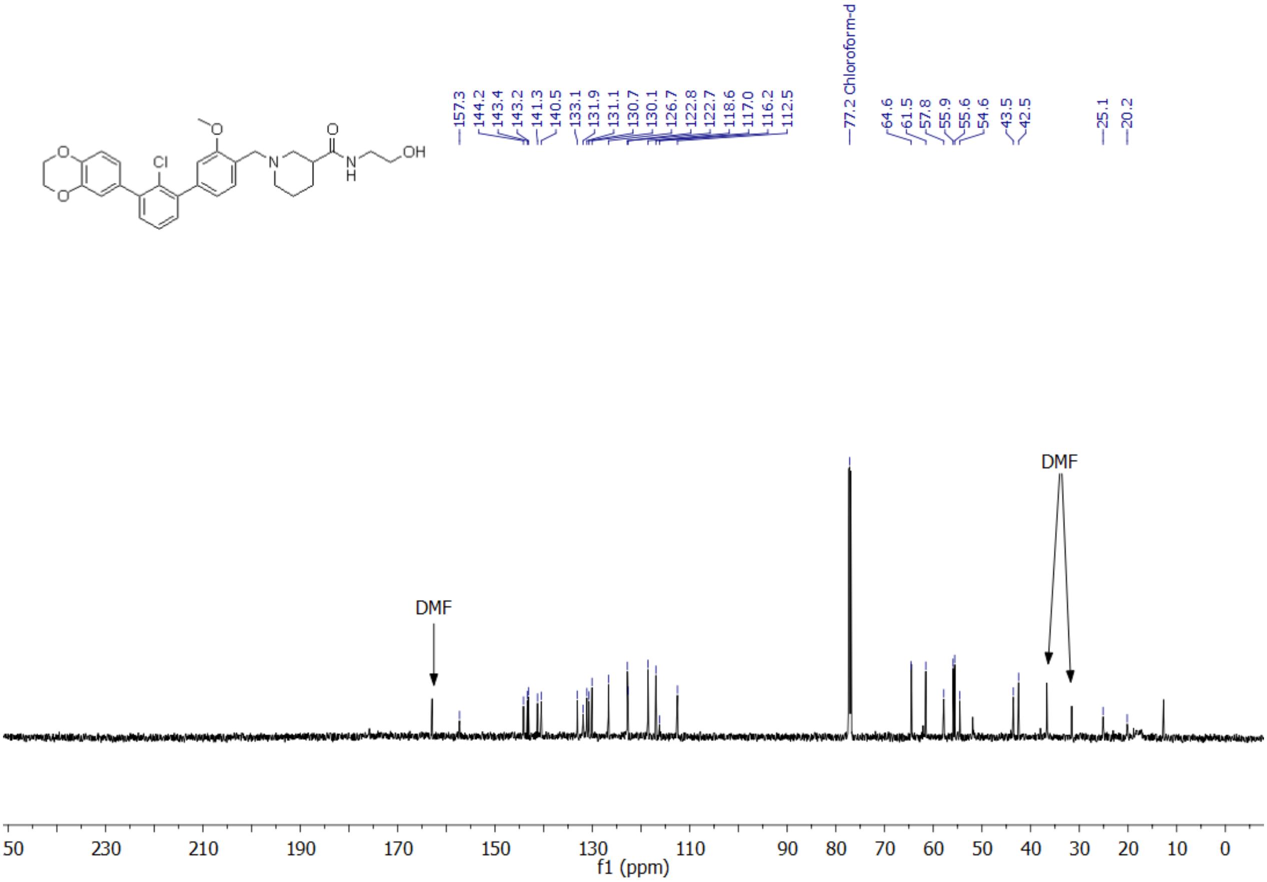

4g: ^{1}H NMR, 600 MHz, $CDCl_3$

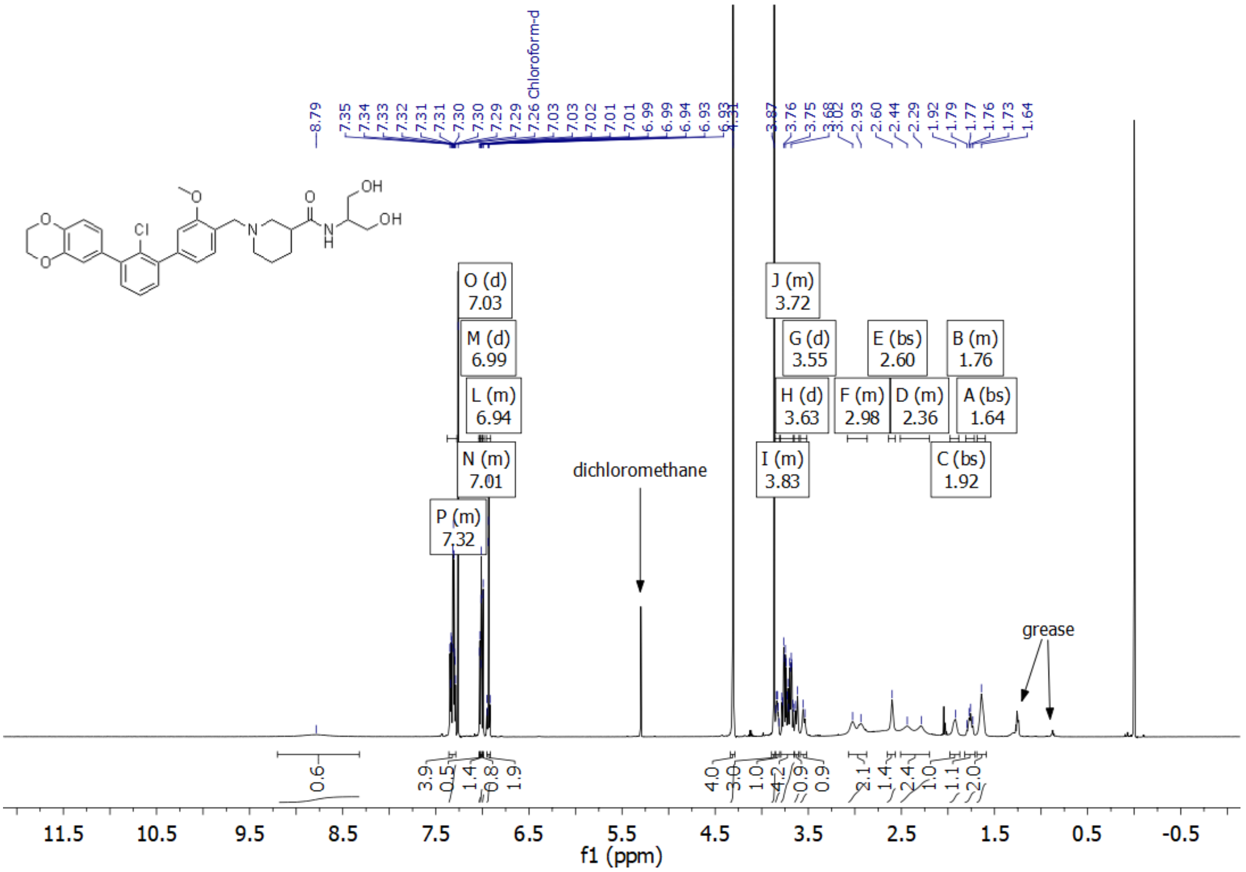

4g: ^{13}C NMR, 151 MHz, $CDCl_3$

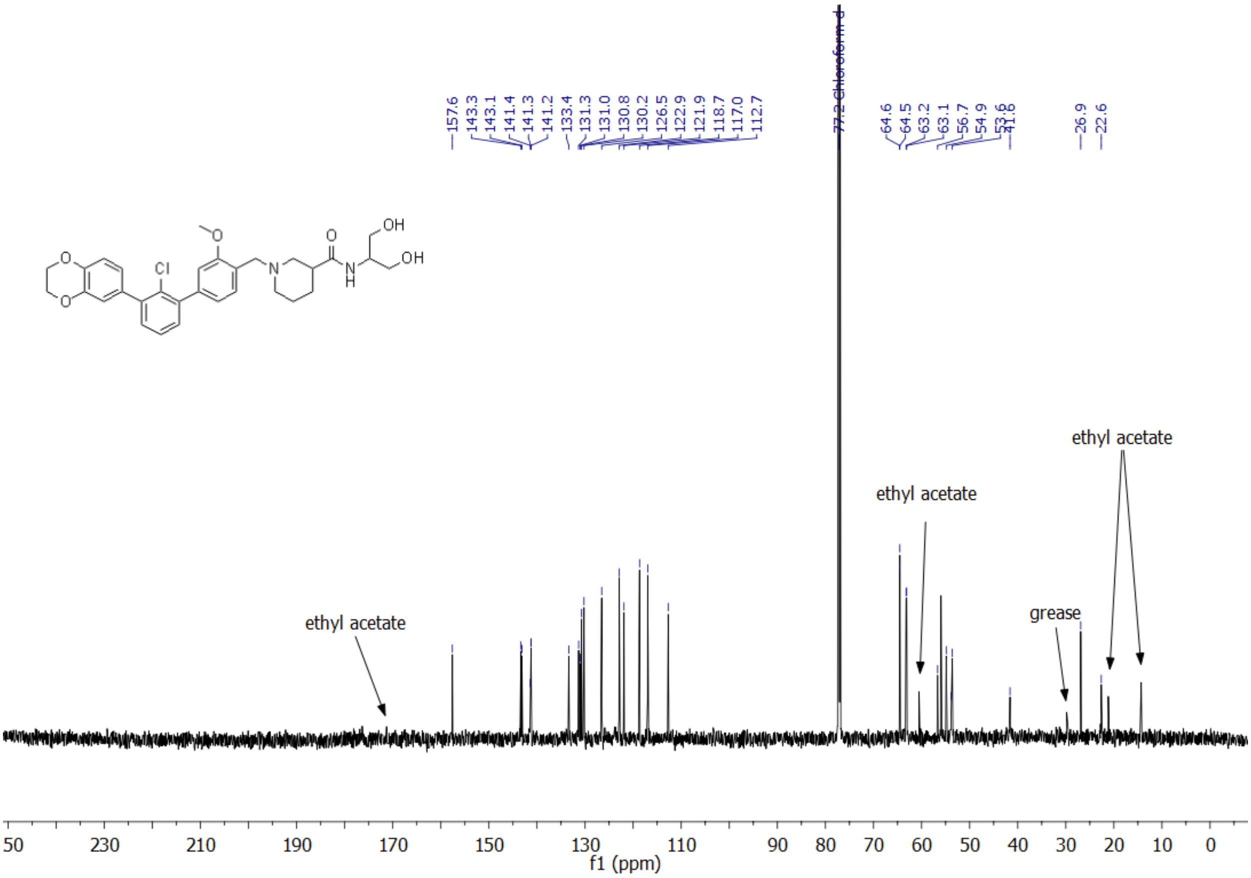

4h: ^{1}H NMR, 600 MHz, $CDCl_3$

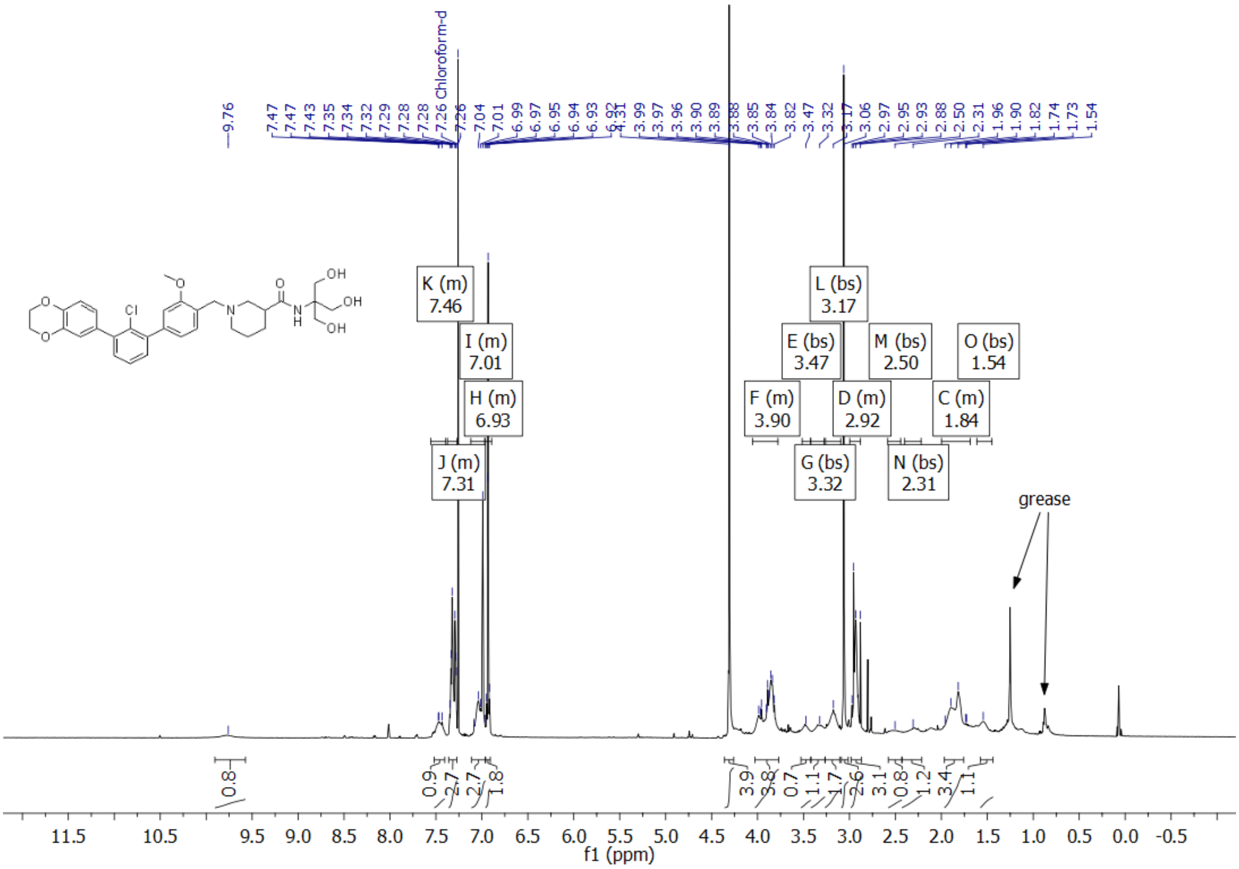

4h: ^{13}C NMR, 151 MHz, $CDCl_3$

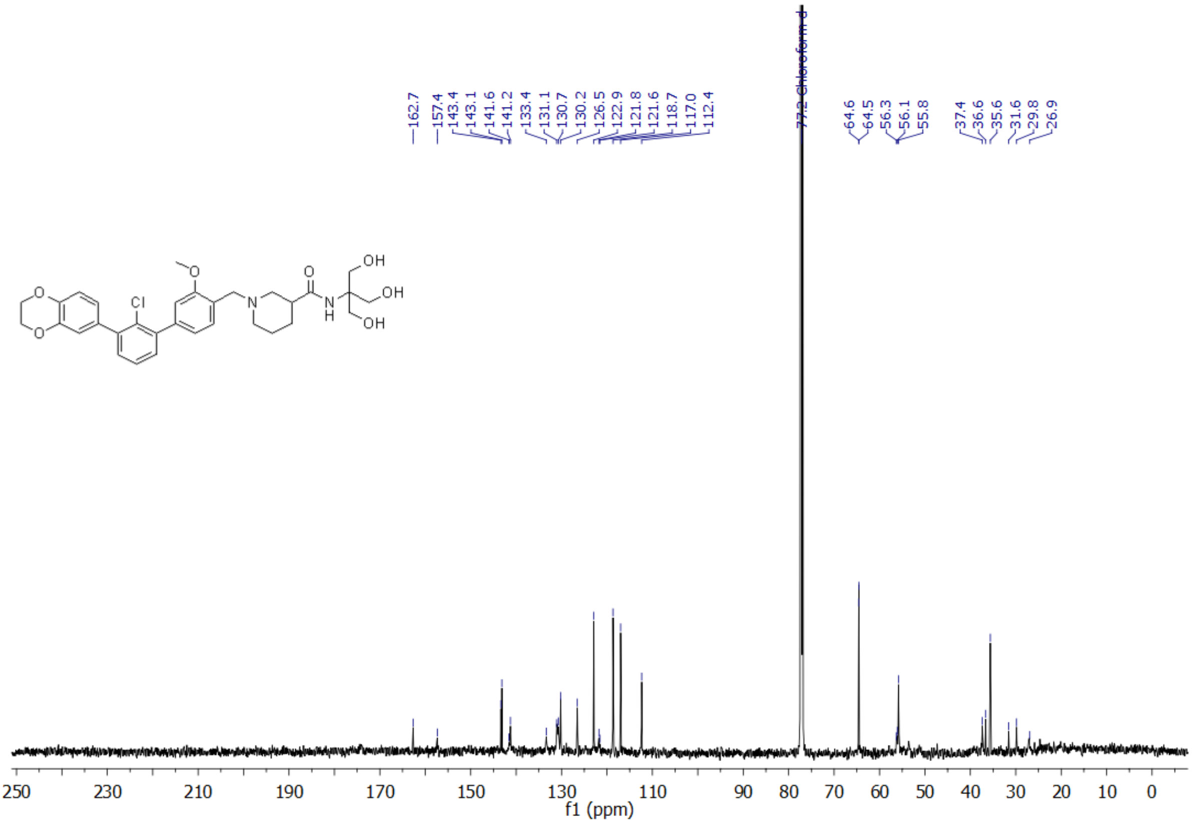

5a: ^{1}H NMR, 600 MHz, DMSO-d_6

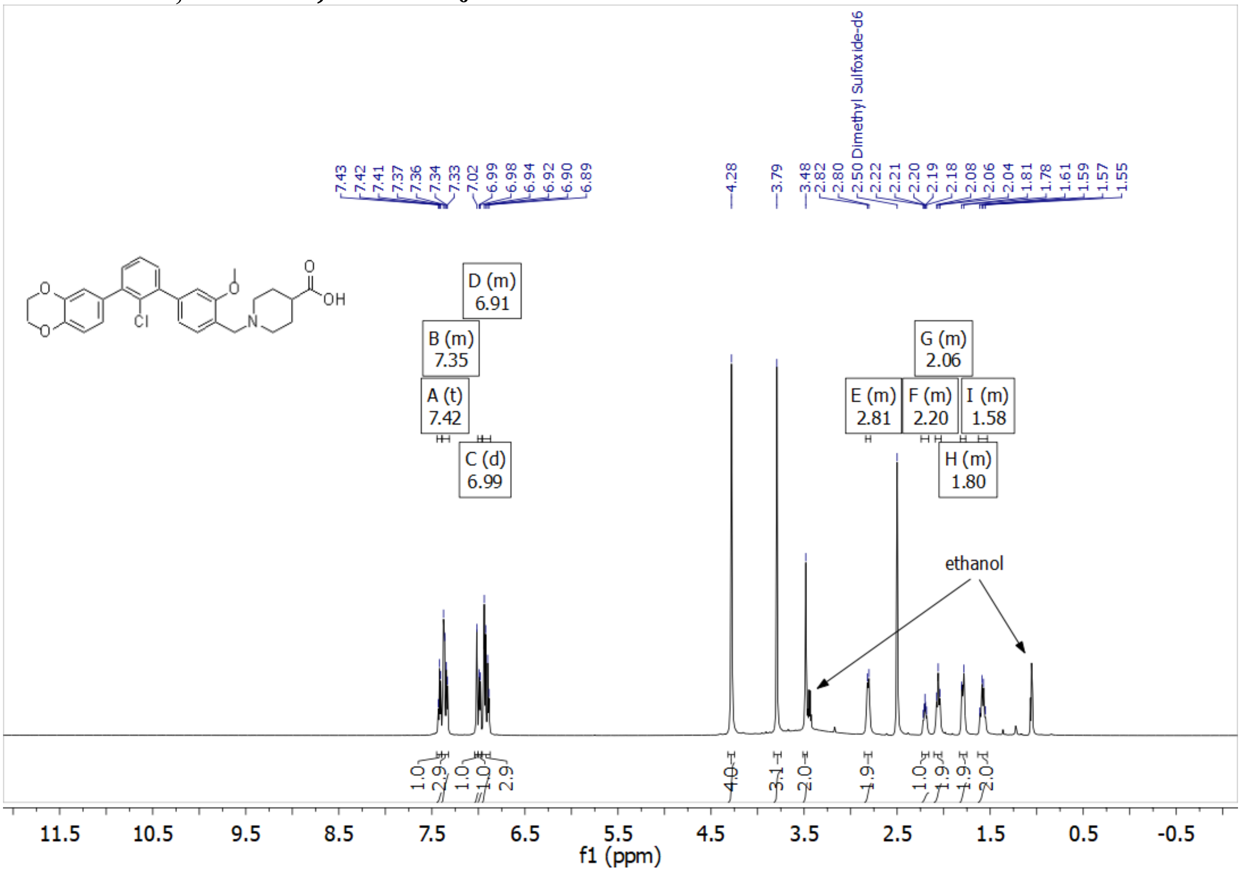

5a: ^{13}C NMR, 151 MHz, DMSO-d_6

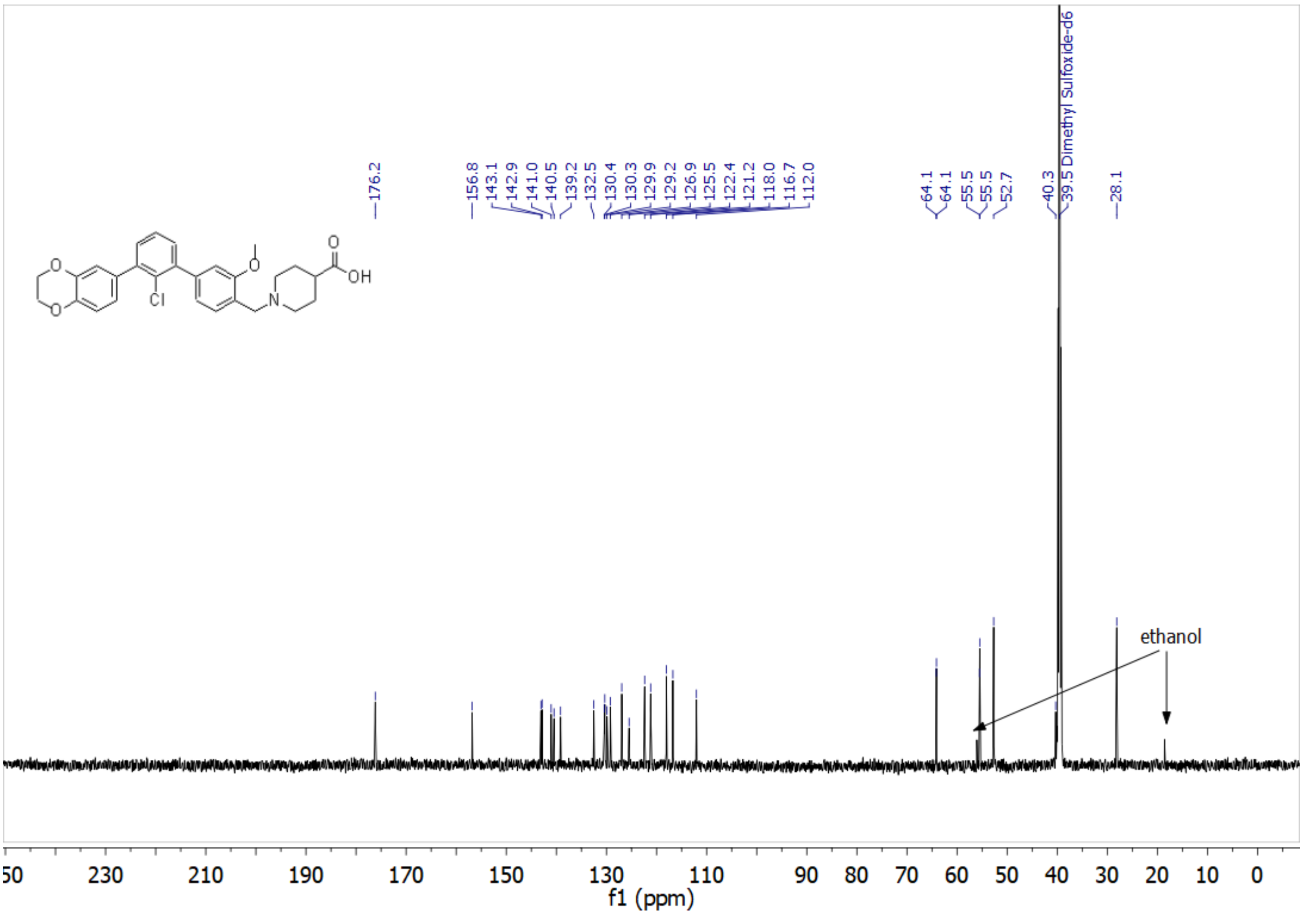

5b: ^{1}H NMR, 600 MHz, DMSO-d_6

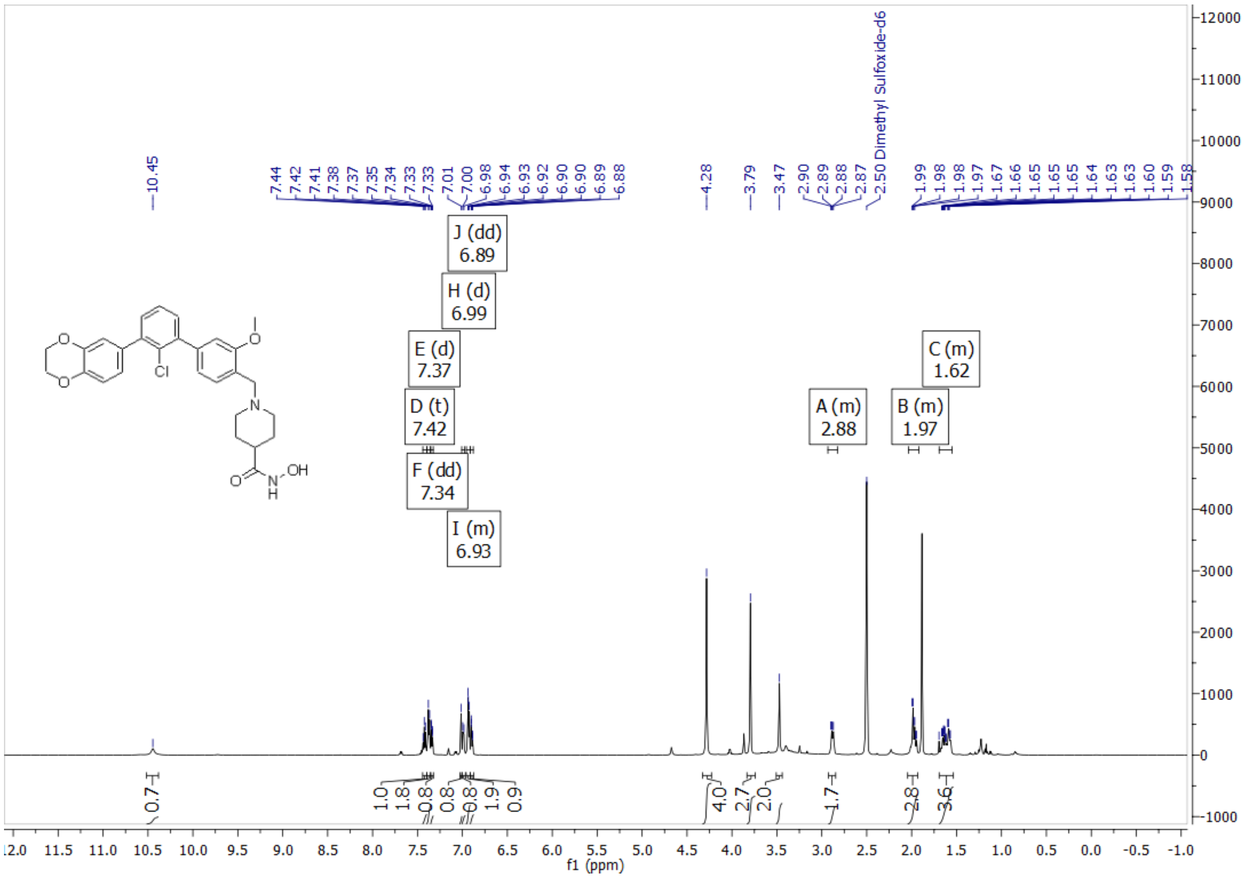

5b: ^{13}C NMR, 151 MHz, DMSO-d_6

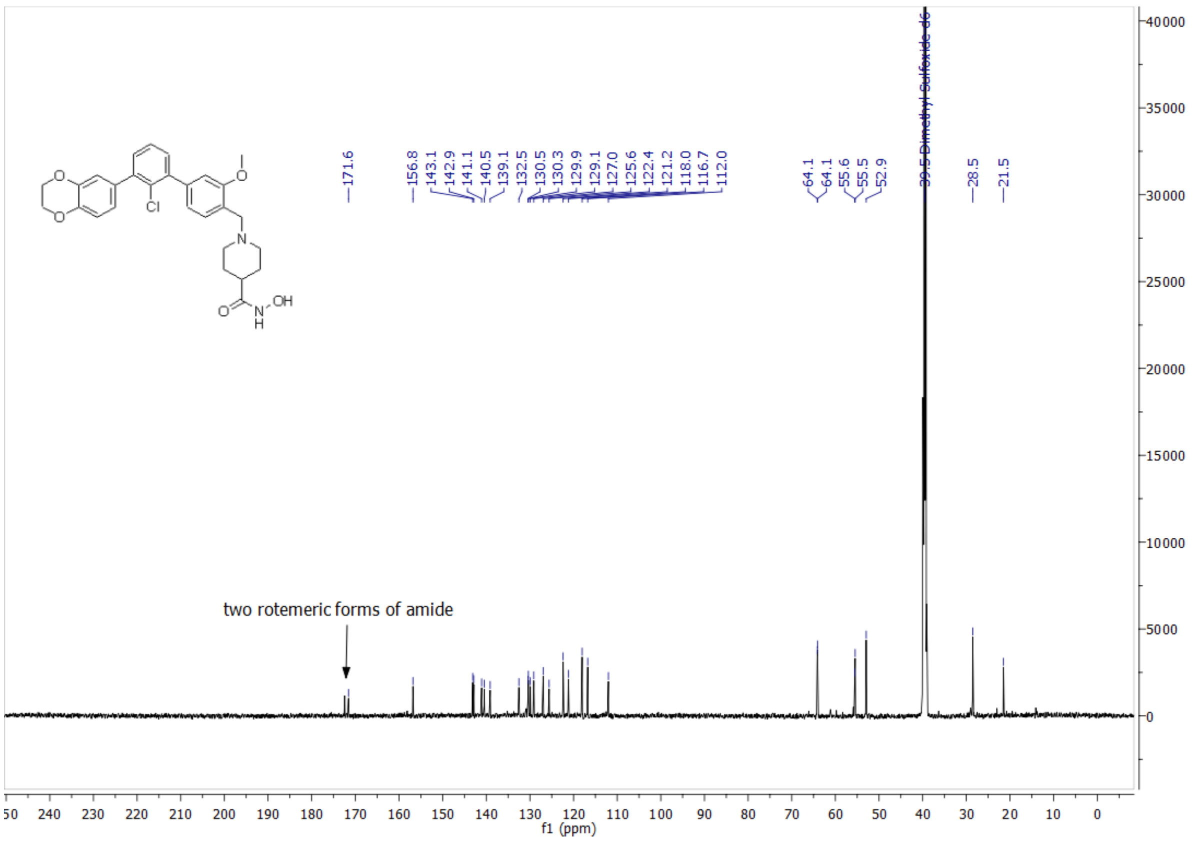

5c: ^{1}H NMR, 600 MHz, DMSO-d_6

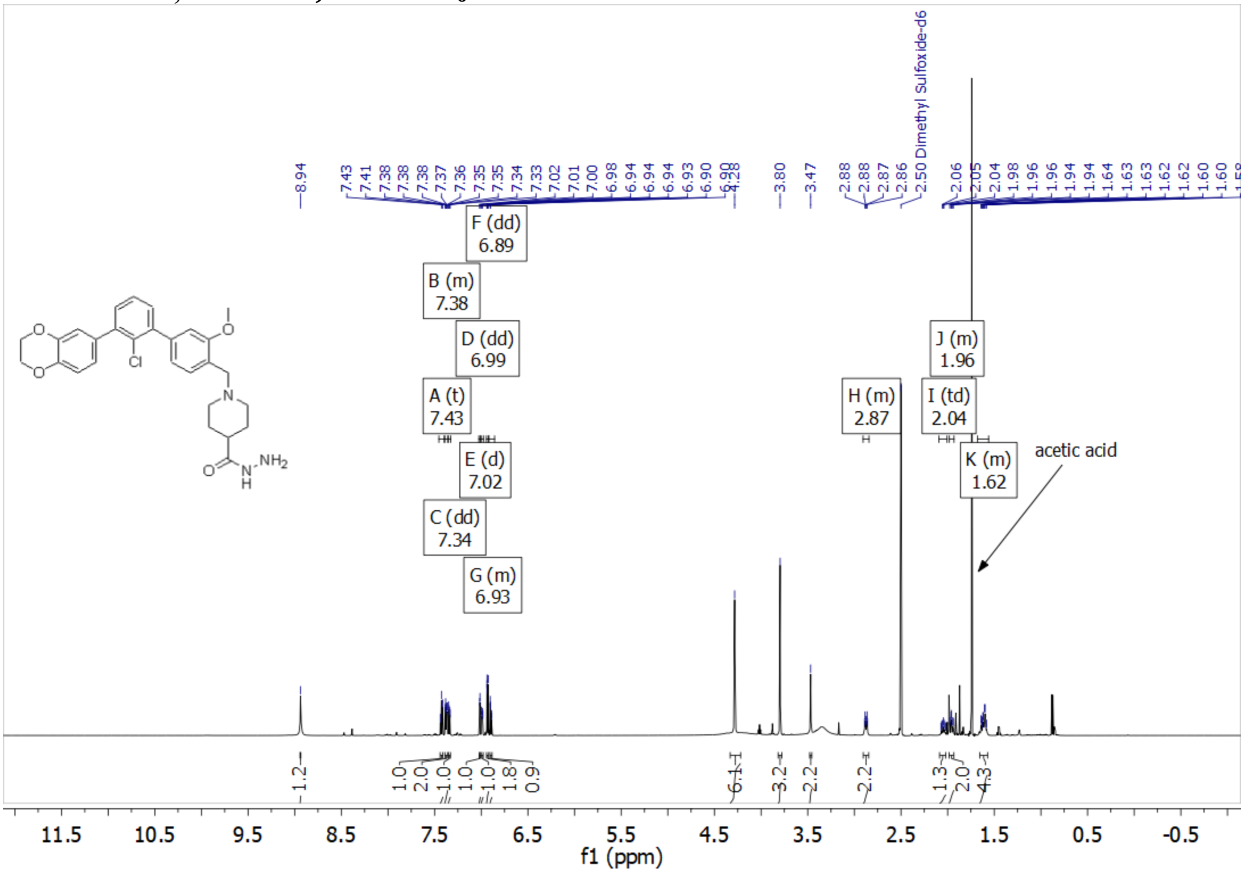

5c: ^{3}C NMR, 151 MHz, DMSO-d_6

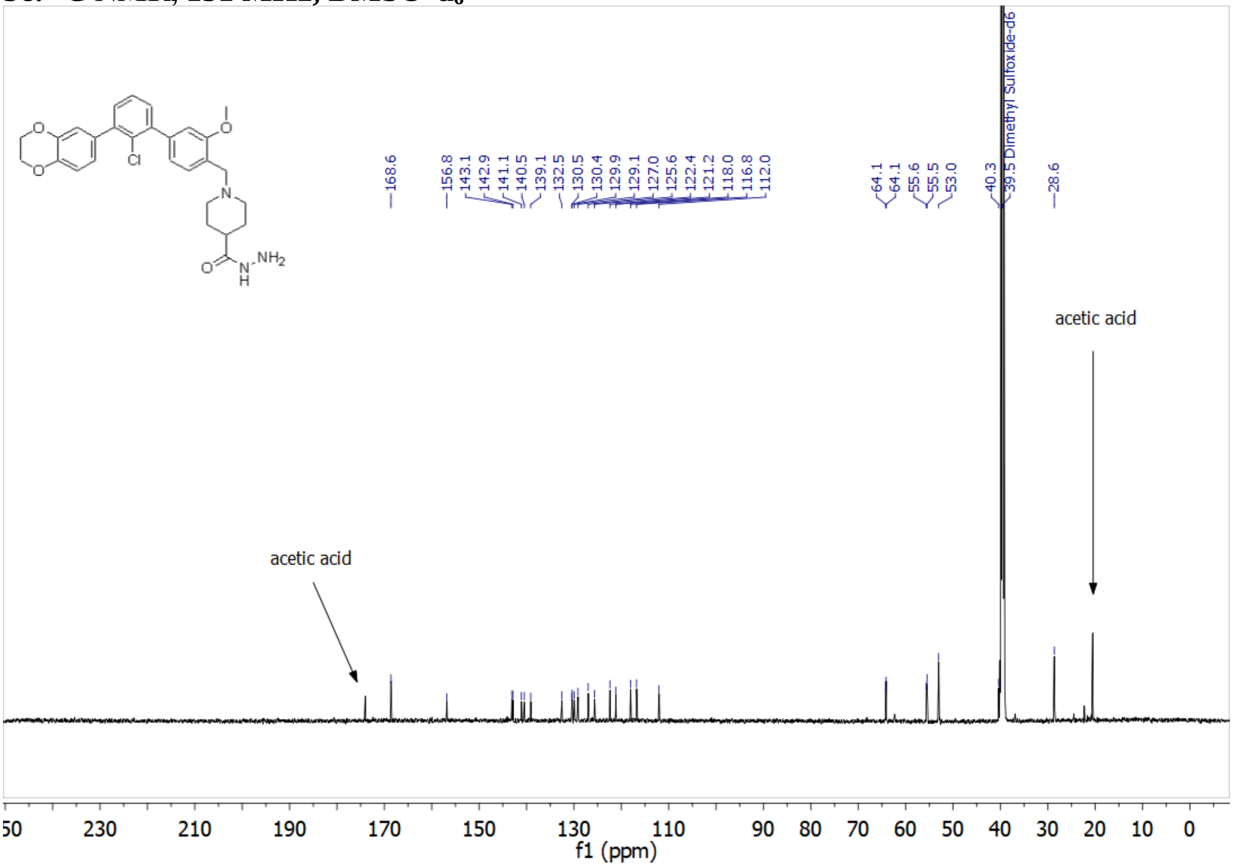

5d: ^{1}H NMR, 600 MHz, DMSO-d$_6$

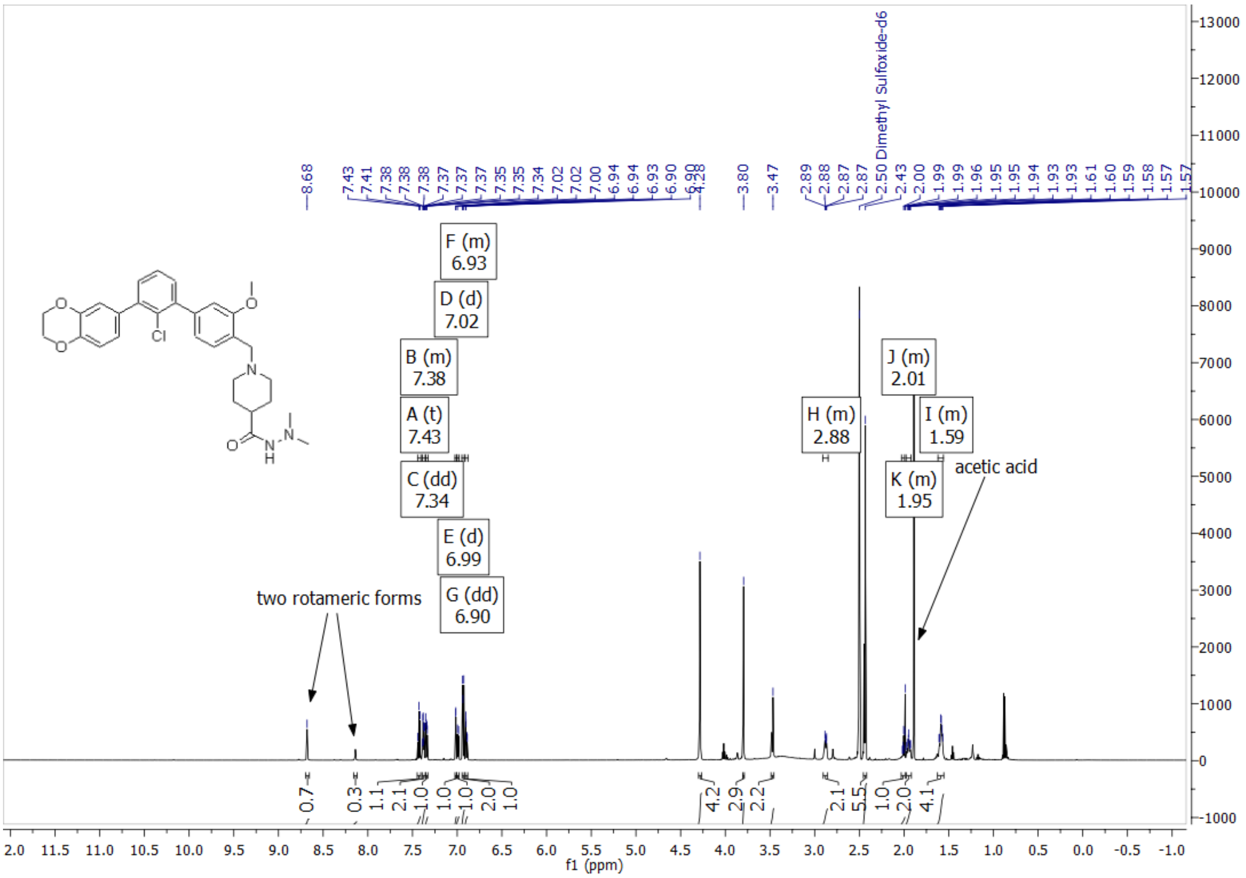

5d: ^{13}C NMR, 151 MHz, DMSO-d$_6$

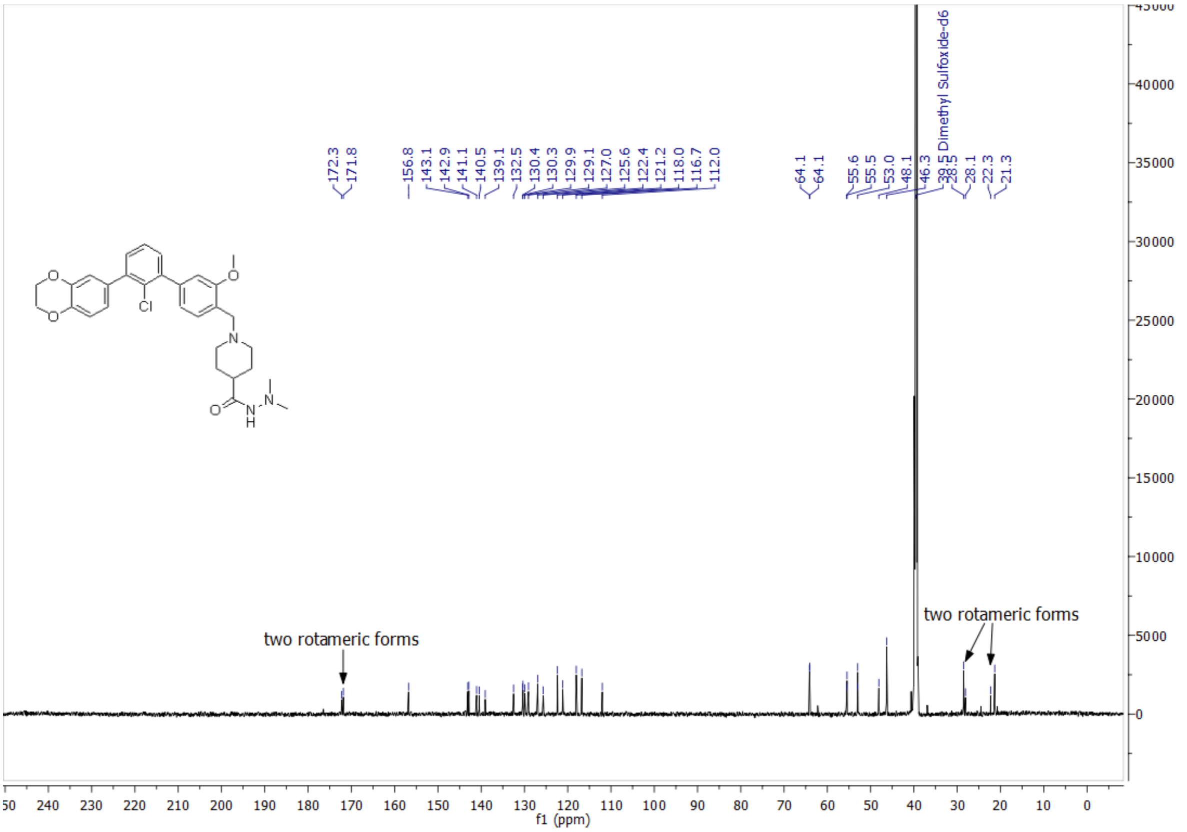

5e: ^{1}H NMR, 600 MHz, DMSO-d$_6$

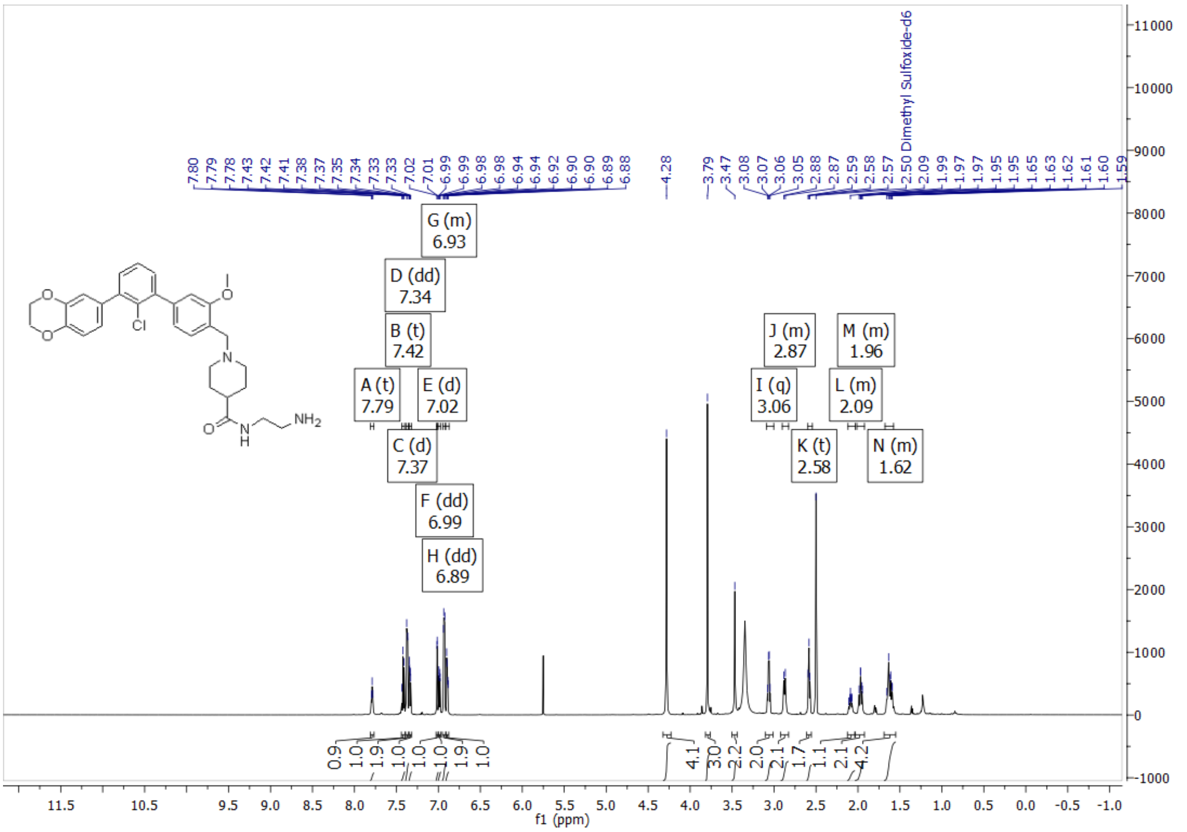

5e: ^{13}C NMR, 151 MHz, DMSO-d$_6$

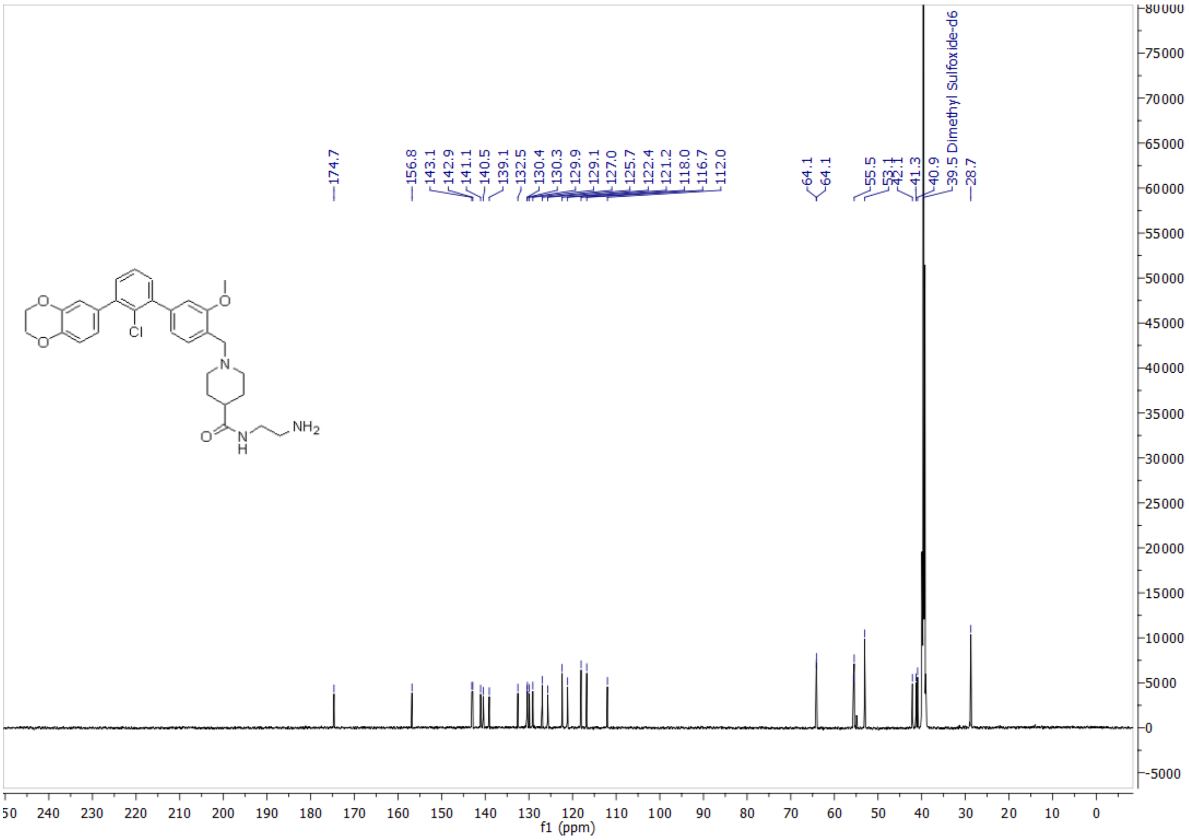

5f: ^{1}H NMR, 600 MHz, DMSO-d$_6$

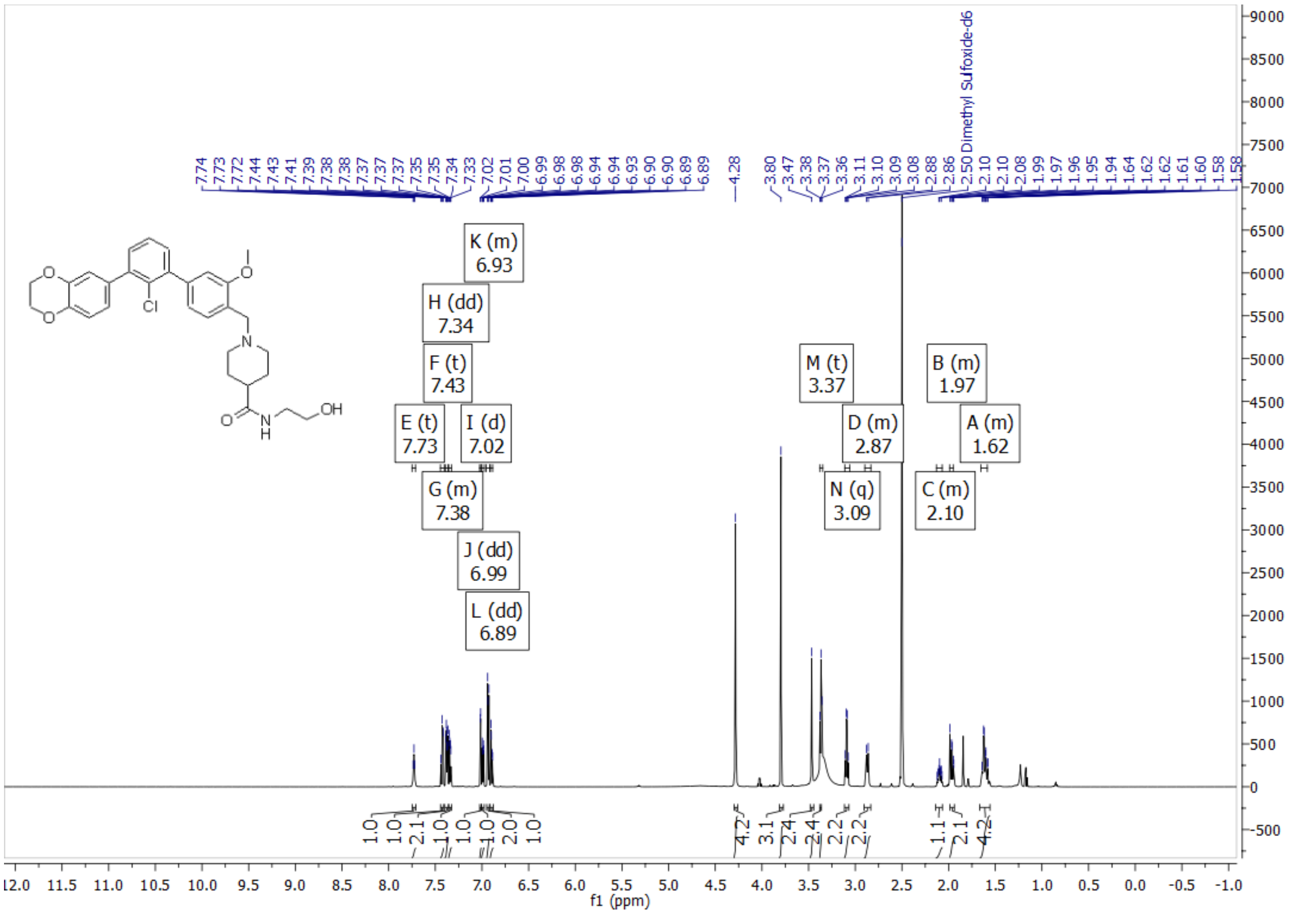

5f: ^{13}C NMR, 151 MHz, DMSO-d$_6$

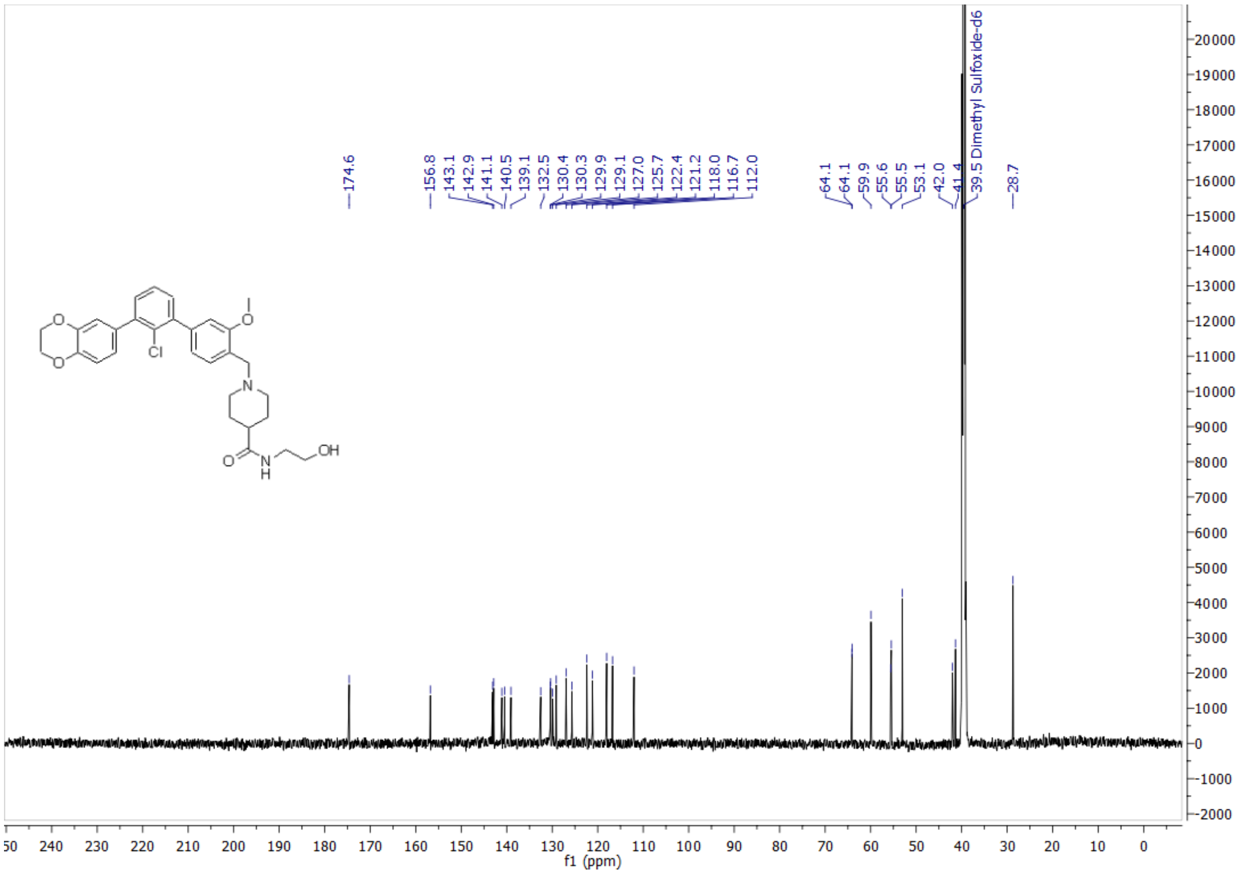

5g: ^{1}H NMR, 600 MHz, DMSO-d_6

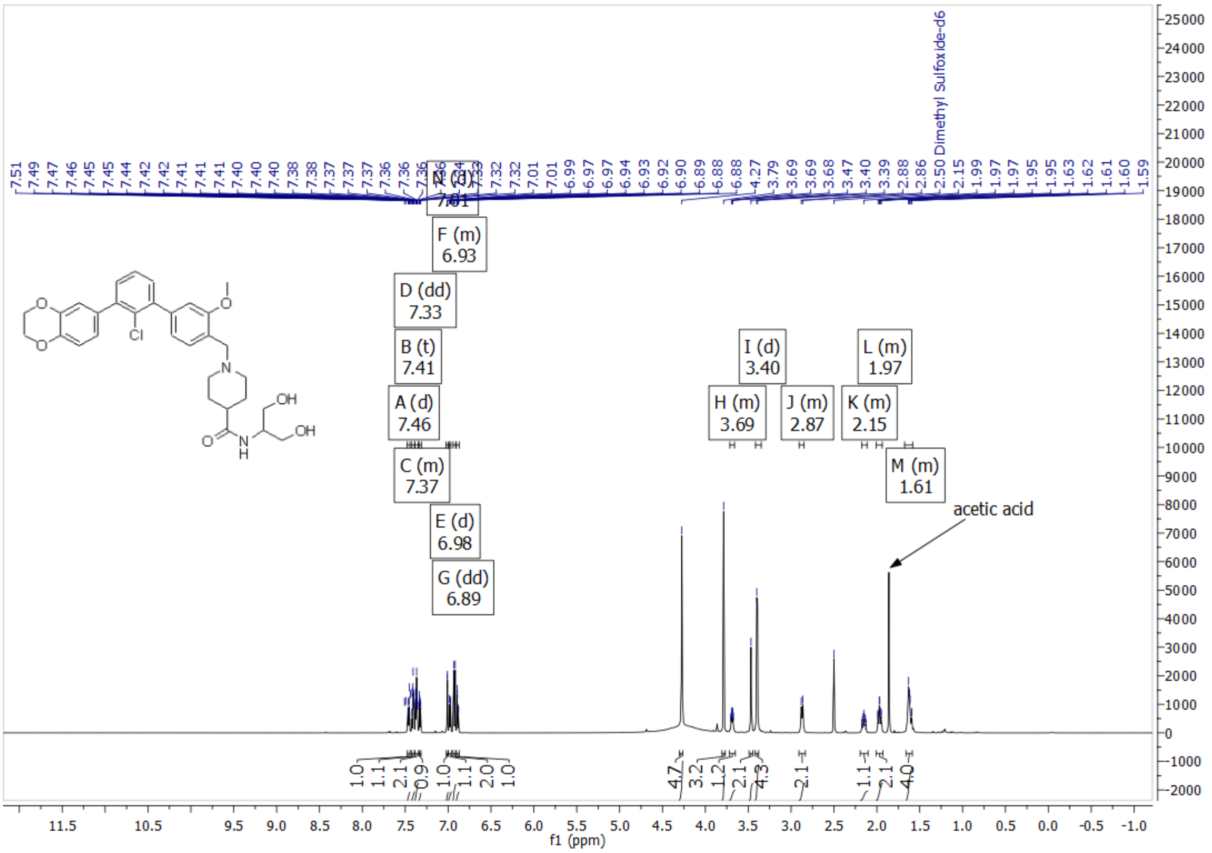

5g: ^{13}C NMR, 151 MHz, DMSO-d_6

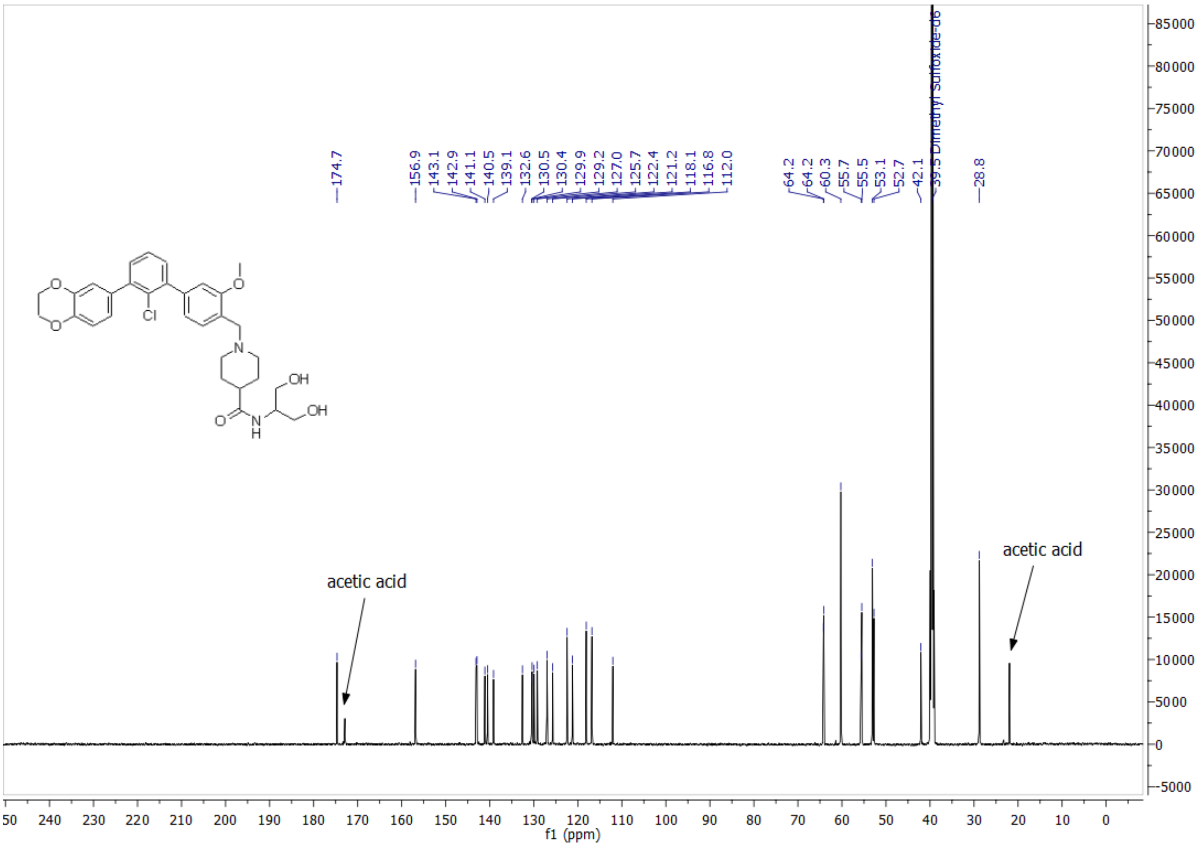

5h: ^{1}H NMR, 600 MHz, DMSO-d_6

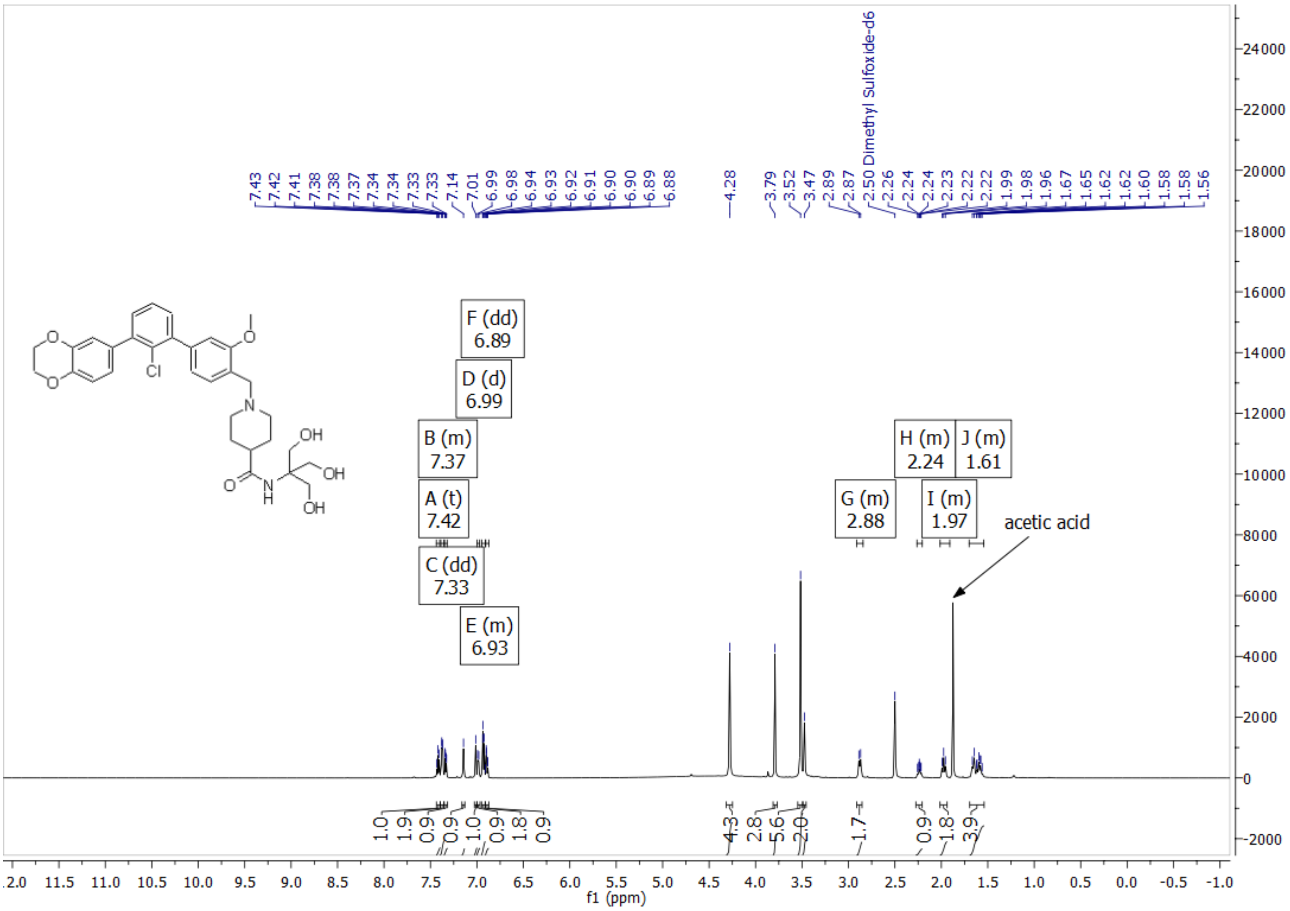

5h: ^{13}C NMR, 151 MHz, DMSO-d_6

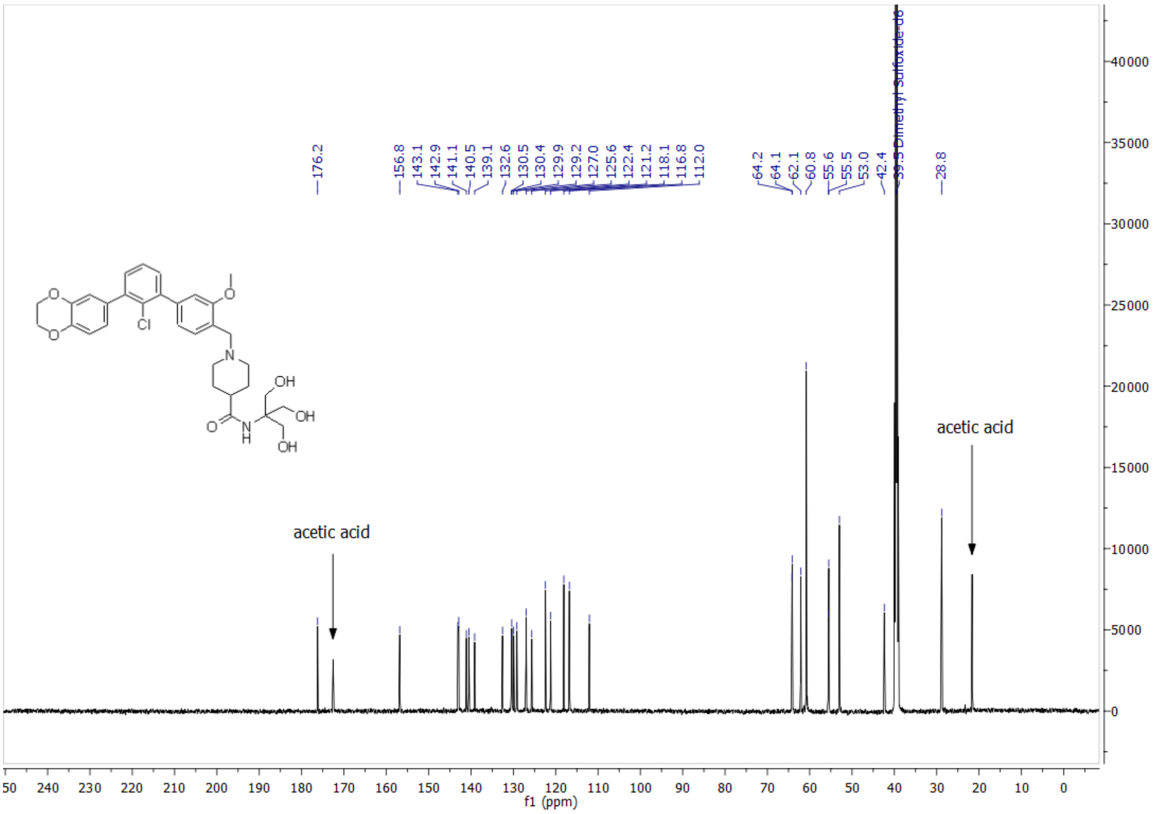

7. COPIES OF THE LCMS OF THE FINAL COMPOUNDS

1a:

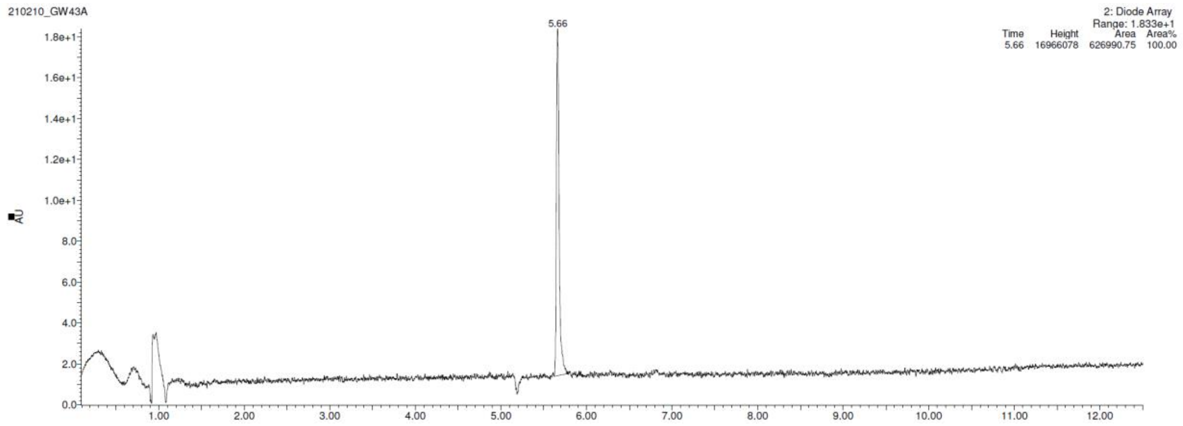

1b:

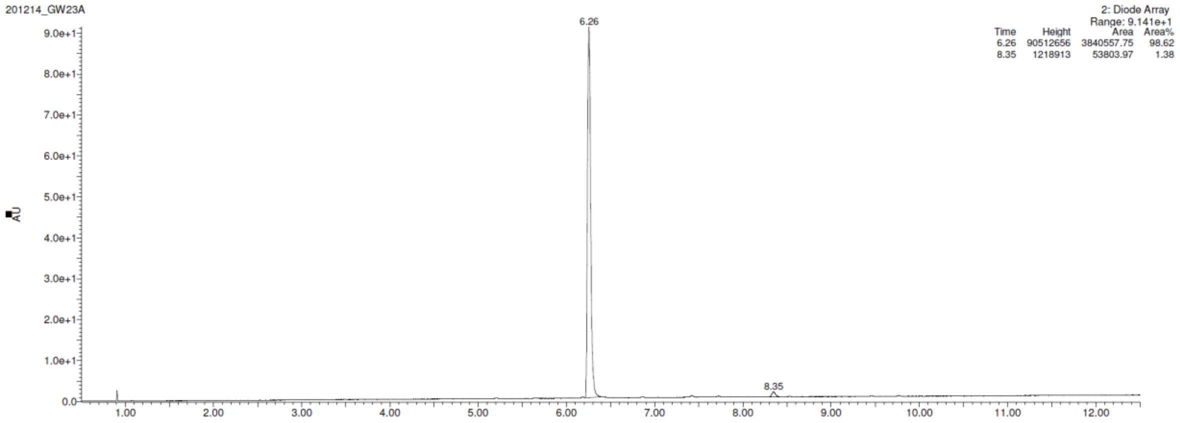

1c:

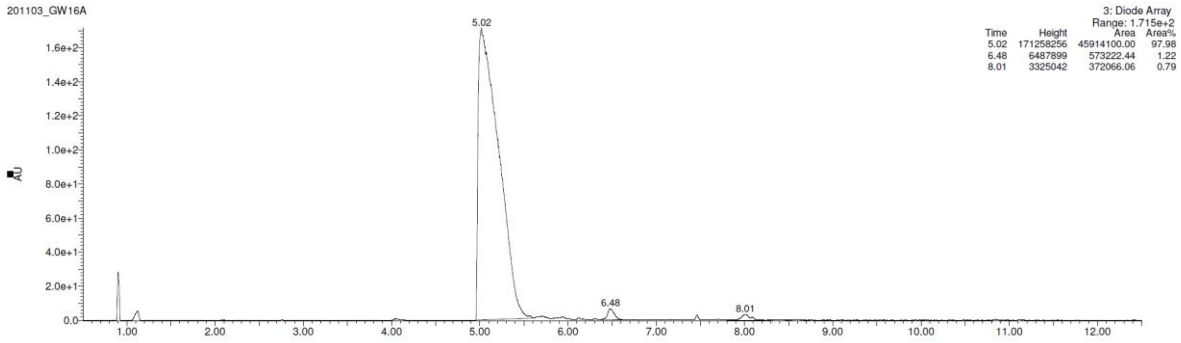

1d:

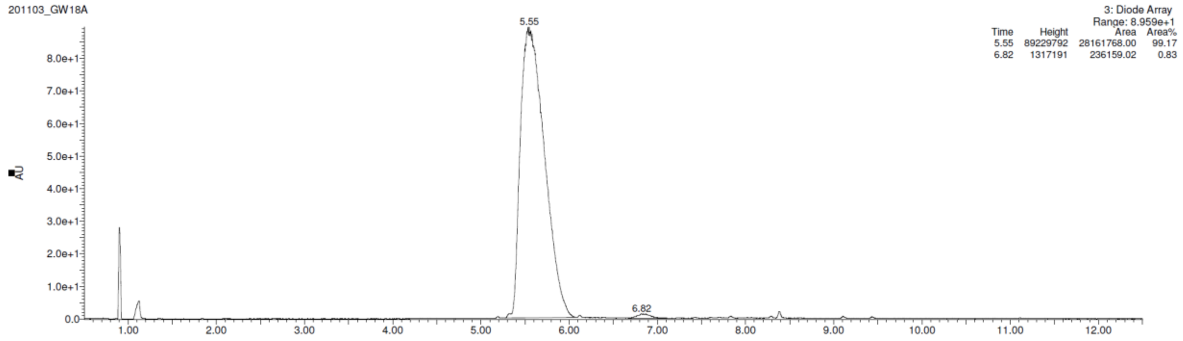
201103_GW18A
3: Diode Array
Range: 8.959e+1
Time Height Area Area%
5.55 89229792 28161768.00 99.17
6.82 1317191 236159.02 0.83
5.55
6.82
AU

1e:

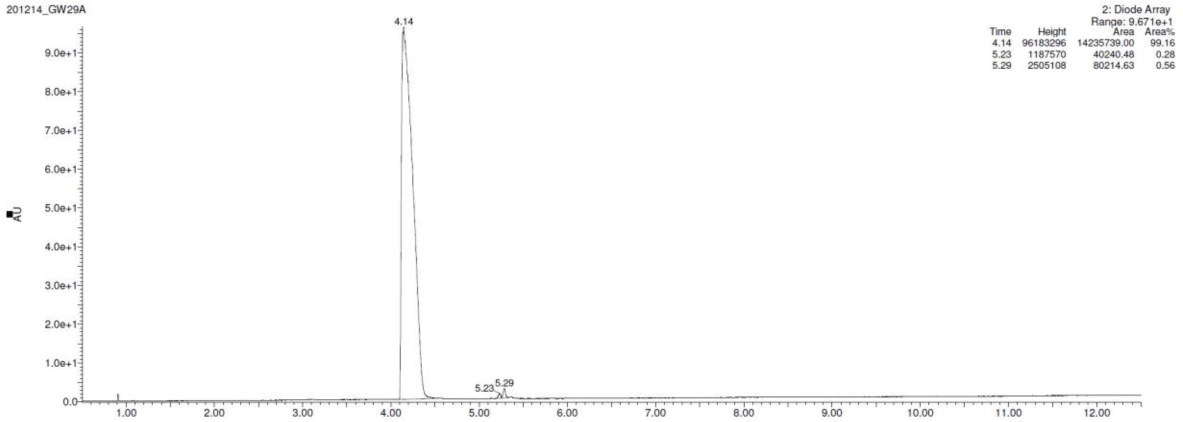
201214_GW29A
2: Diode Array
Range: 9.671e+1
Time Height Area Area%
4.14 96183296 14235739.00 99.16
5.23 1187570 40240.48 0.28
5.29 2505108 80214.63 0.56
4.14
5.23
5.29
AU

1f:

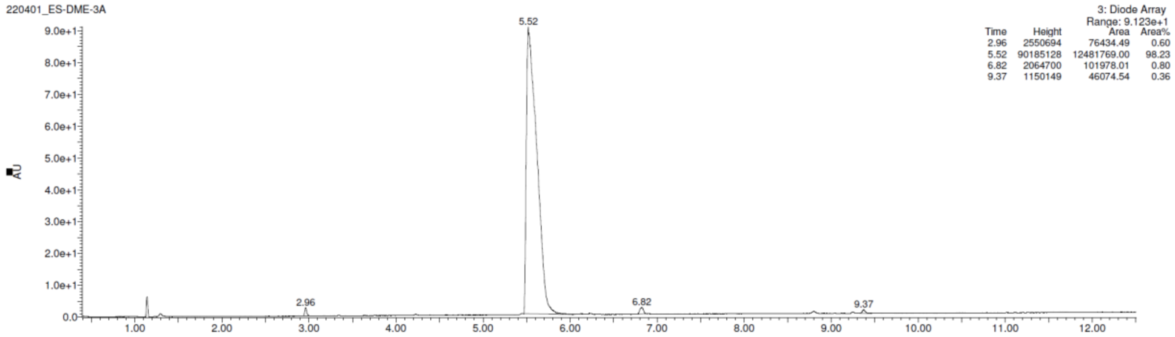
220401_ES-DME-3A
3: Diode Array
Range: 9.123e+1
Time Height Area Area%
2.96 2550694 76434.49 0.60
5.52 90185128 12481769.00 98.23
6.82 2064700 101978.01 0.80
9.37 1150149 46074.54 0.36
5.52
2.96
6.82
9.37
AU

1g:

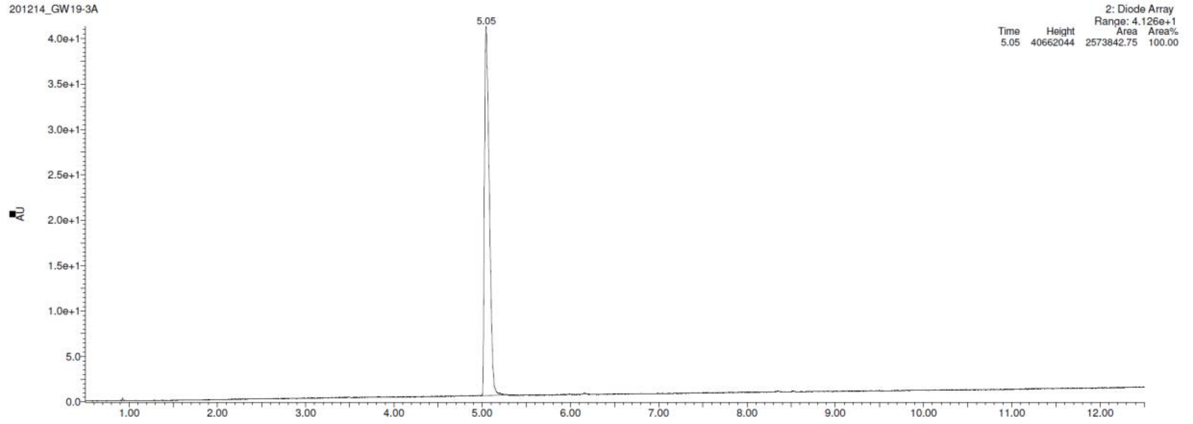
201214_GW19-3A
5.05
2: Diode Array
Range: 4.126e+1
Time Height Area Area%
5.05 40662044 2573842.75 100.00
AU
4.0e+1
3.5e+1
3.0e+1
2.5e+1
2.0e+1
1.5e+1
1.0e+1
5.0
0.0
1.00 2.00 3.00 4.00 5.00 6.00 7.00 8.00 9.00 10.00 11.00 12.00

1h:

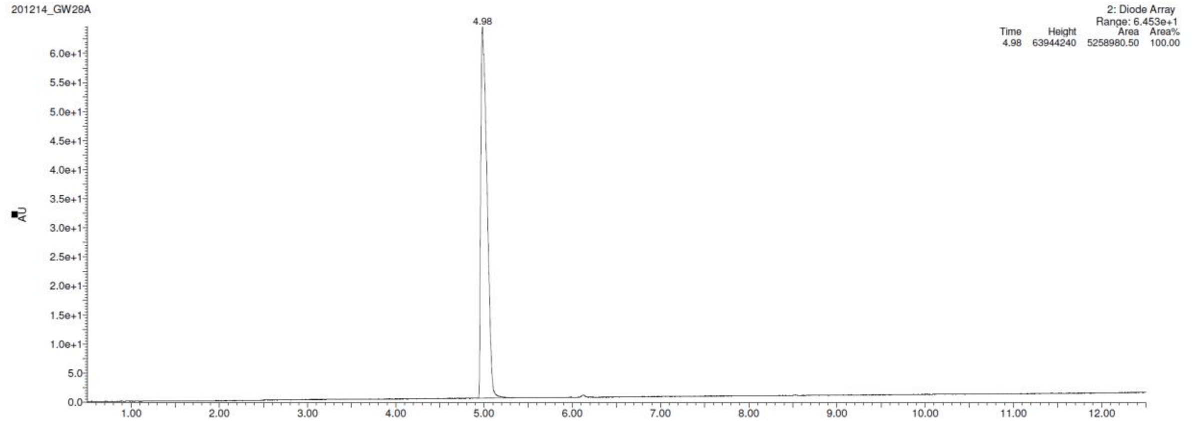
201214_GW28A
4.98
2: Diode Array
Range: 6.453e+1
Time Height Area Area%
4.98 63944240 5258980.50 100.00
AU
6.0e+1
5.5e+1
5.0e+1
4.5e+1
4.0e+1
3.5e+1
3.0e+1
2.5e+1
2.0e+1
1.5e+1
1.0e+1
5.0
0.0
1.00 2.00 3.00 4.00 5.00 6.00 7.00 8.00 9.00 10.00 11.00 12.00

2a:

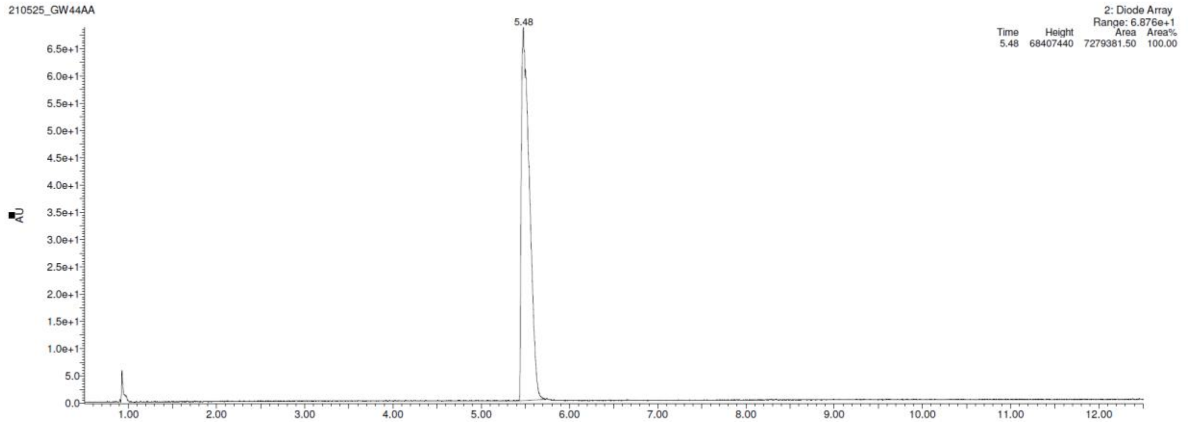
210525_GW44AA
5.48
2: Diode Array
Range: 6.876e+1
Time Height Area Area%
5.48 68407440 7279381.50 100.00
AU
6.5e+1
6.0e+1
5.5e+1
5.0e+1
4.5e+1
4.0e+1
3.5e+1
3.0e+1
2.5e+1
2.0e+1
1.5e+1
1.0e+1
5.0
0.0
1.00 2.00 3.00 4.00 5.00 6.00 7.00 8.00 9.00 10.00 11.00 12.00

2b:

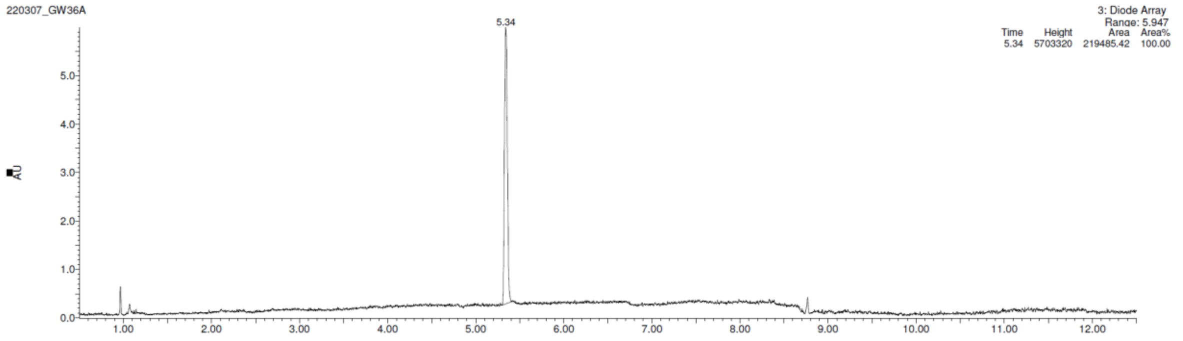

220307_GW36A
3: Diode Array
Range: 5.947
Time Height Area Area%
5.34 5703320 219485.42 100.00
5.34
AU

2c:

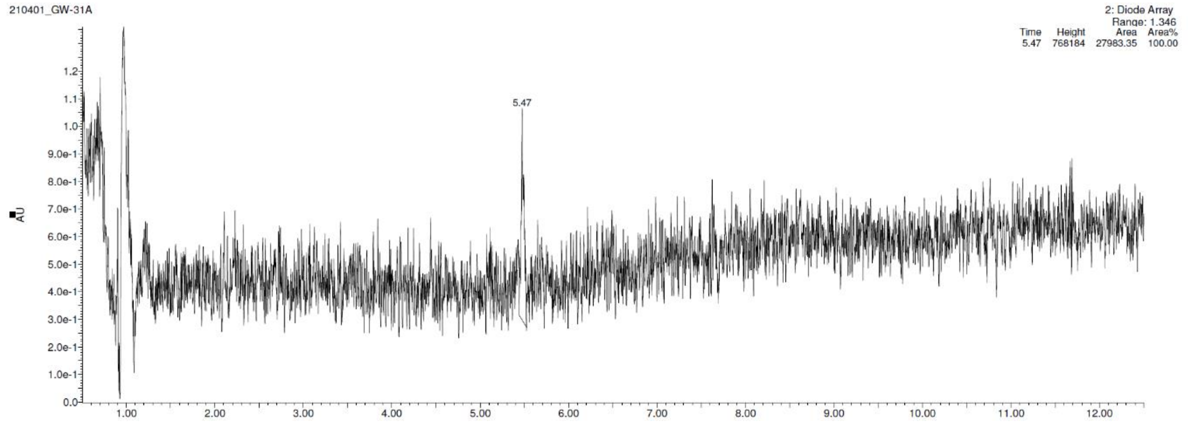

210401_GW-31A
2: Diode Array
Range: 1.346
Time Height Area Area%
5.47 768184 27983.35 100.00
5.47
AU

2d:

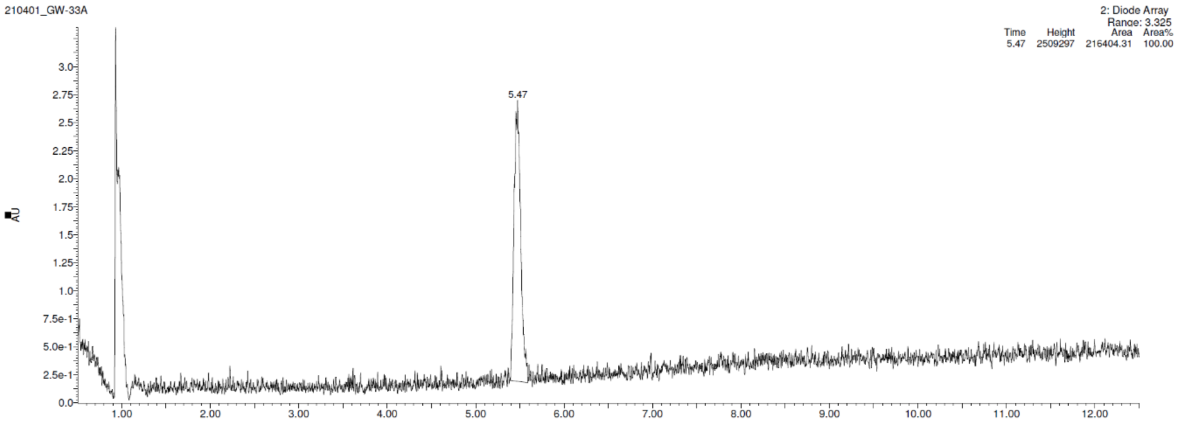

210401_GW-33A
2: Diode Array
Range: 3.325
Time Height Area Area%
5.47 2509297 216404.31 100.00
5.47
AU

2e:

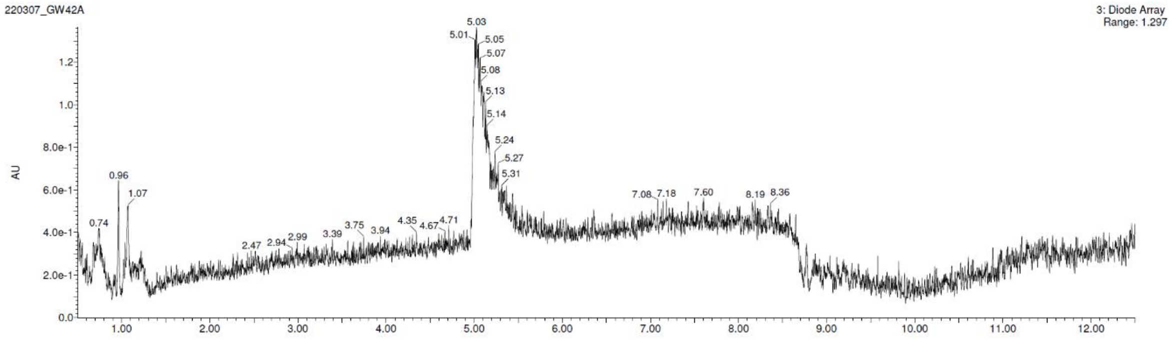
220307_GW42A
3: Diode Array
Range: 1.297
AU

2f:

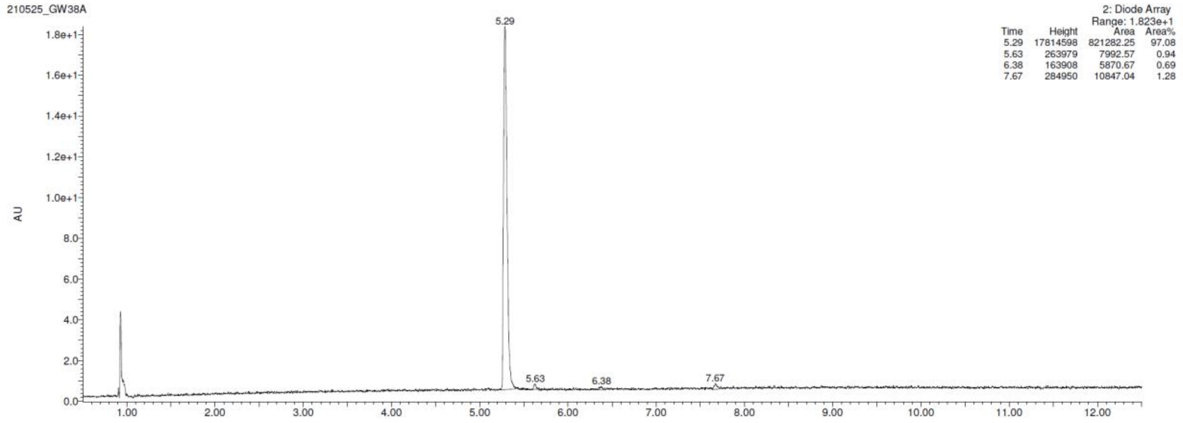
210525_GW38A
2: Diode Array
Range: 1.823e+1
Time Height Area Area%
5.29 17814598 821282.25 97.08
5.63 263979 7992.57 0.94
6.38 163908 5870.67 0.69
7.67 284950 10847.04 1.28
AU

2g:

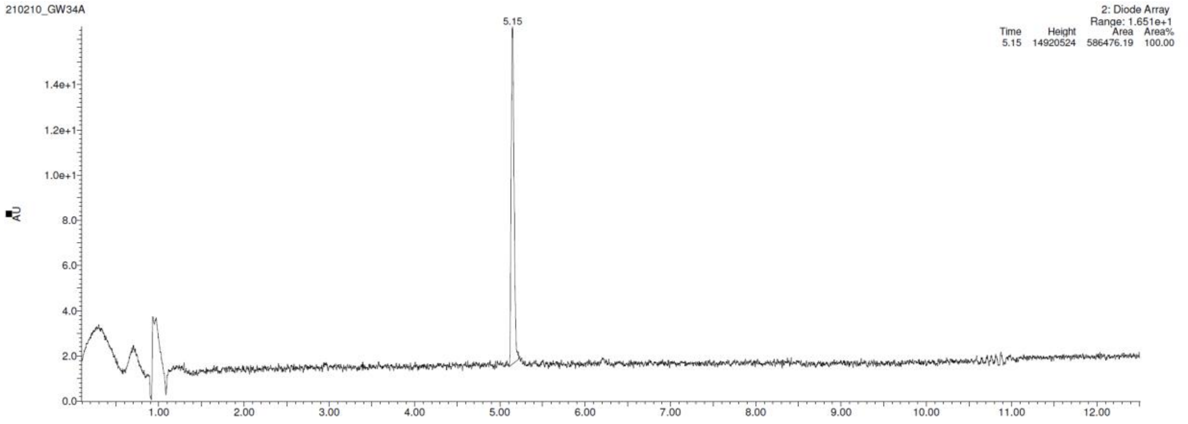
210210_GW34A
2: Diode Array
Range: 1.651e+1
Time Height Area Area%
5.15 14920524 586476.19 100.00
AU

2h:

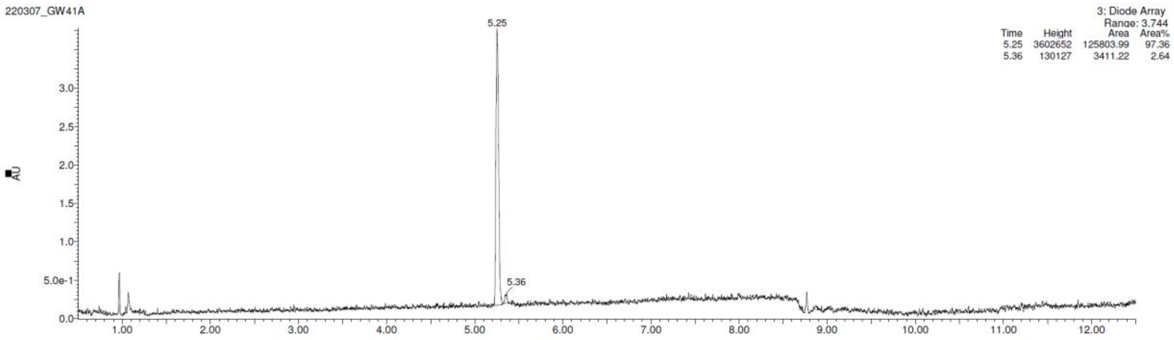
220307_GW41A
3: Diode Array
Range: 3.744
Time Height Area Area%
5.25 3602652 125803.99 97.36
5.36 130127 3411.22 2.64
5.25
5.36
AU

3a:

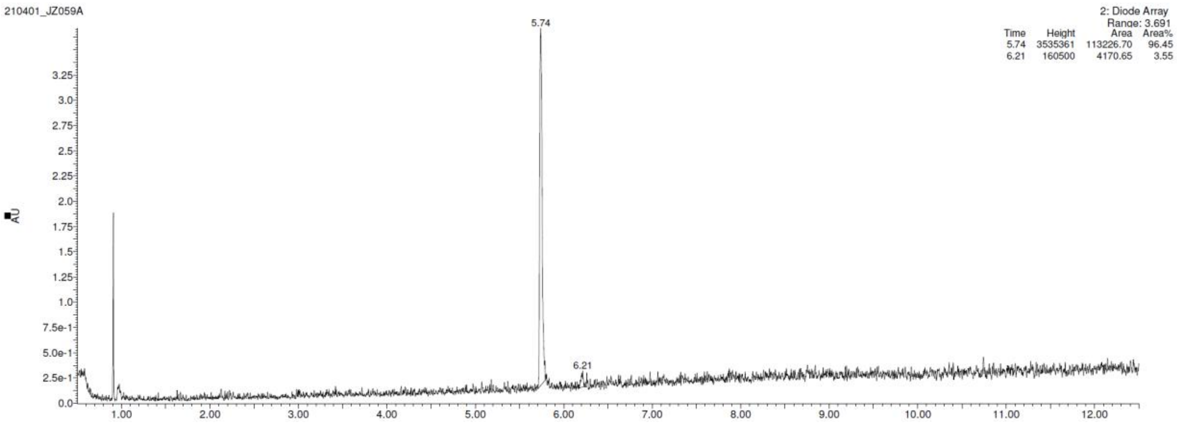
210401_JZ059A
2: Diode Array
Range: 3.691
Time Height Area Area%
5.74 3535361 113226.70 96.45
6.21 160500 4170.65 3.55
5.74
6.21
AU

3c:

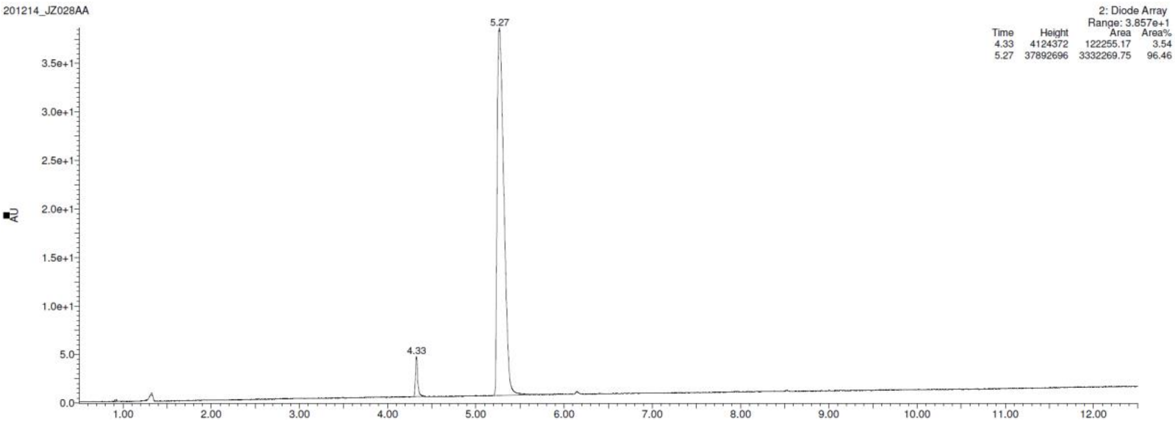
201214_JZ028AA
2: Diode Array
Range: 3.857e+1
Time Height Area Area%
4.33 4124372 122255.17 3.54
5.27 37892696 3332269.75 96.46
5.27
4.33
AU

3d:

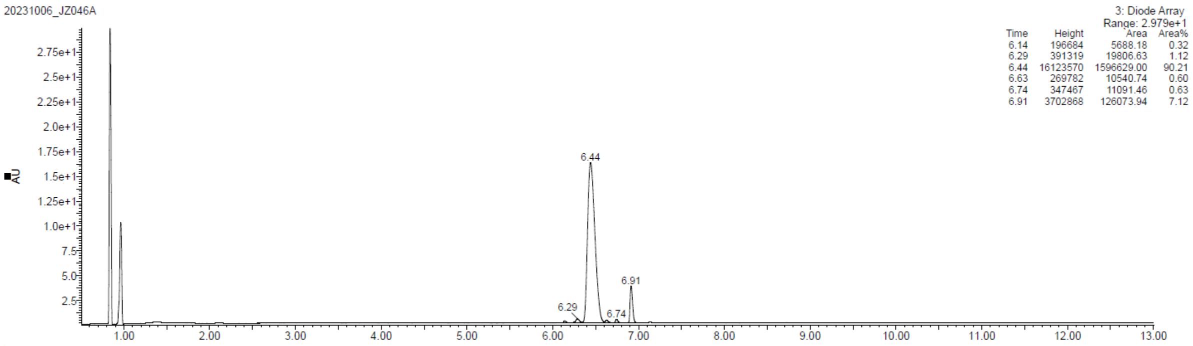
20231006_JZ046A
3: Diode Array
Range: 2.979e+1
Time Height Area Area%
6.14 196684 5688.18 0.32
6.29 391319 19806.63 1.12
6.44 16123570 1596629.00 90.21
6.63 269782 10540.74 0.60
6.74 347467 11091.46 0.63
6.91 3702868 126073.94 7.12
6.44
6.91
6.29
6.74
AU

3f:

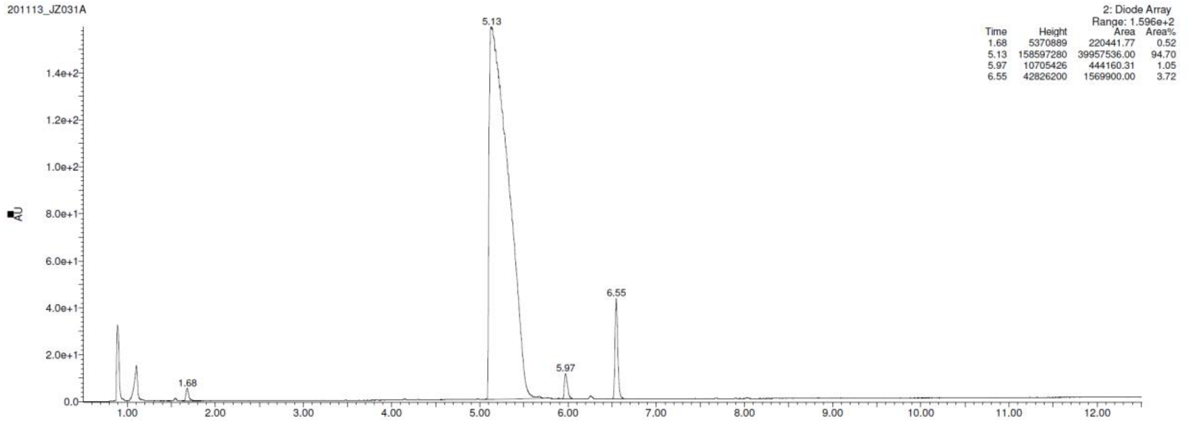
201113_JZ031A
2: Diode Array
Range: 1.596e+2
Time Height Area Area%
1.68 5370889 220441.77 0.52
5.13 158597280 39957536.00 94.70
5.97 10705426 444160.31 1.05
6.55 42826200 1569900.00 3.72
AU
5.13
6.55
5.97
1.68

3g:

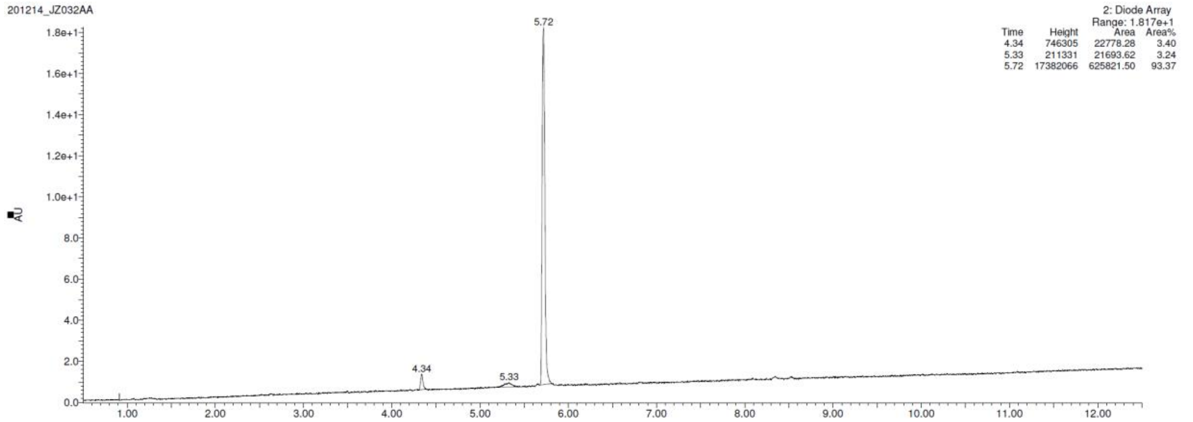
201214_JZ032AA
2: Diode Array
Range: 1.817e+1
Time Height Area Area%
4.34 746305 22778.28 3.40
5.33 211331 21693.62 3.24
5.72 17382066 625821.50 93.37
AU
5.72
4.34
5.33

3h:

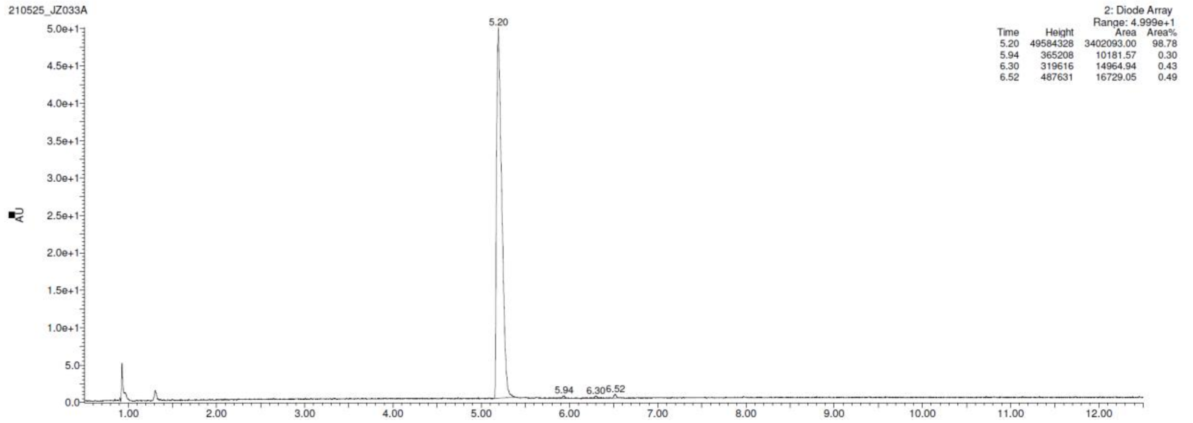
210525_JZ033A
2: Diode Array
Range: 4.999e+1
Time Height Area Area%
5.20 49584328 3402093.00 98.78
5.94 365208 10181.57 0.30
6.30 319616 14964.94 0.43
6.52 487631 16729.05 0.49
AU
5.20
5.94
6.30
6.52

4a:

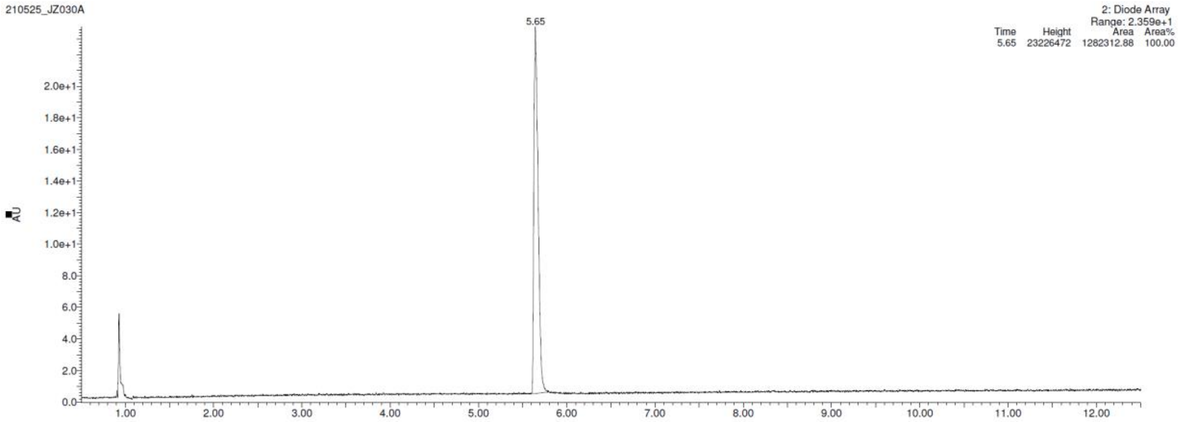
210525_JZ030A
2: Diode Array
Range: 2.359e+1
Time Height Area Area%
5.65 23226472 1282312.88 100.00
5.65
AU

4b:

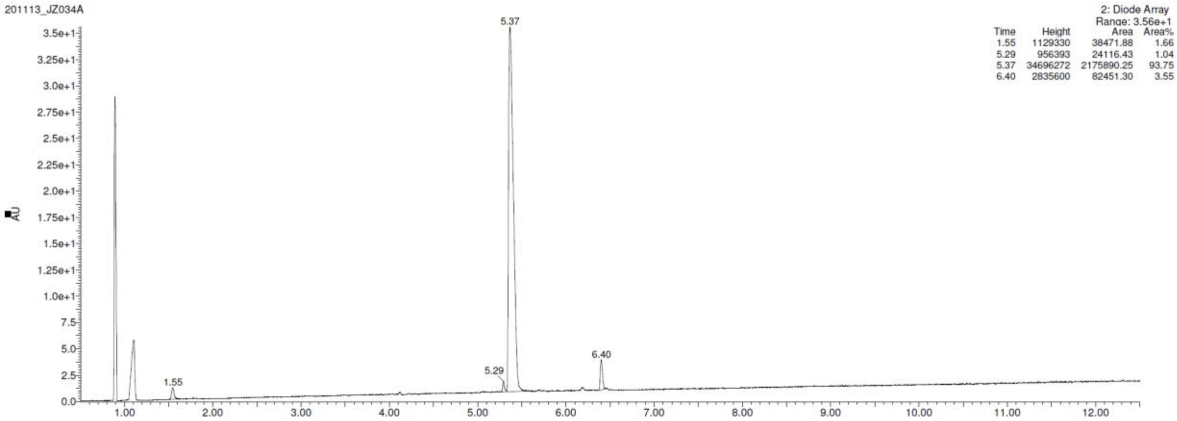
201113_JZ034A
2: Diode Array
Range: 3.56e+1
Time Height Area Area%
1.55 1129330 38471.88 1.66
5.29 956393 24116.43 1.04
5.37 34696272 2175890.25 93.75
6.40 2835600 82451.30 3.55
5.37
5.29
6.40
1.55
AU

4c:

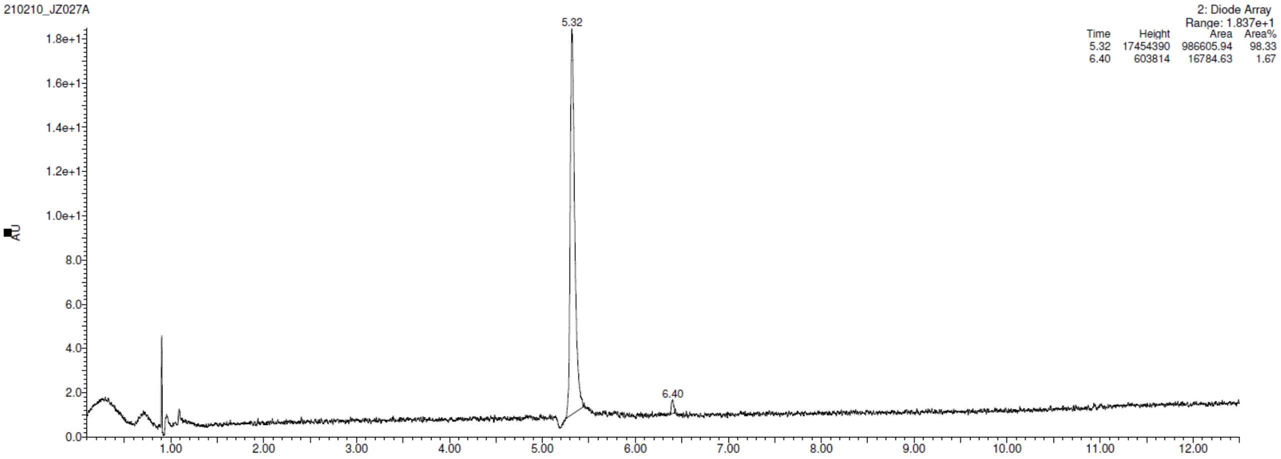
210210_JZ027A
2: Diode Array
Range: 1.837e+1
Time Height Area Area%
5.32 17454390 986605.94 98.33
6.40 603814 16784.63 1.67
5.32
6.40
AU

4d:

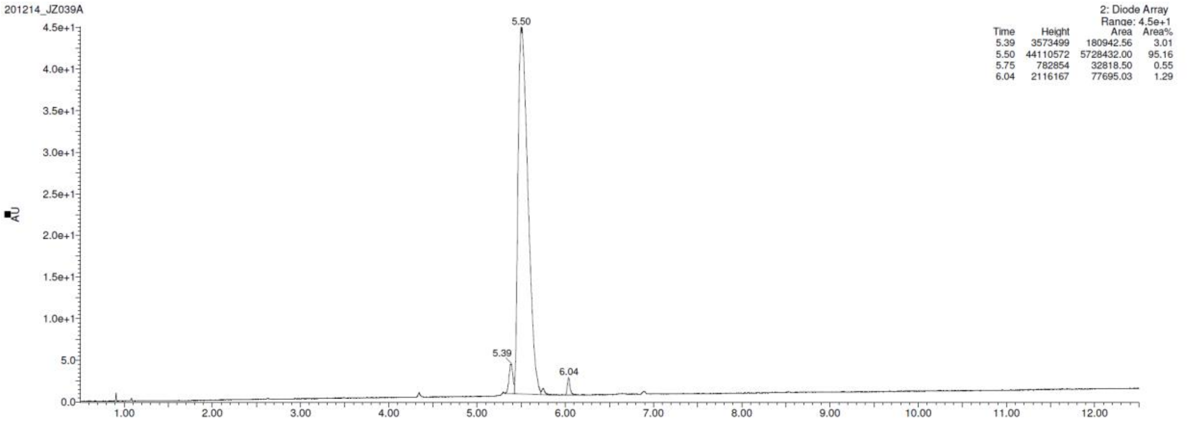
201214_JZ039A
2: Diode Array
Range: 4.5e+1
Time Height Area Area%
5.39 3573499 180942.56 3.01
5.50 44110572 5728432.00 95.16
5.75 782854 32818.50 0.55
6.04 2116167 77695.03 1.29
5.50
5.39
6.04
AU
4.5e+1
4.0e+1
3.5e+1
3.0e+1
2.5e+1
2.0e+1
1.5e+1
1.0e+1
5.0
0.0
1.00 2.00 3.00 4.00 5.00 6.00 7.00 8.00 9.00 10.00 11.00 12.00

4f:

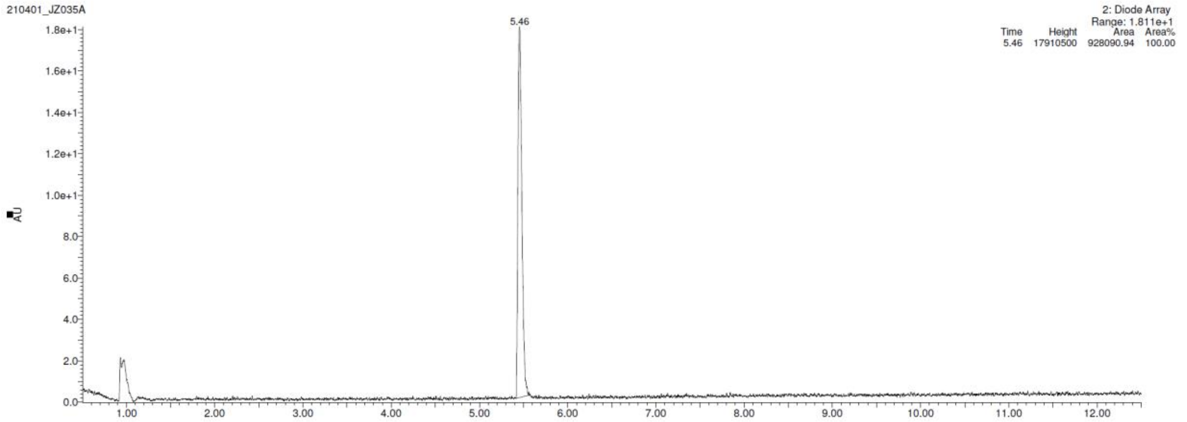
210401_JZ035A
2: Diode Array
Range: 1.811e+1
Time Height Area Area%
5.46 17910500 928090.94 100.00
5.46
AU
1.8e+1
1.6e+1
1.4e+1
1.2e+1
1.0e+1
8.0
6.0
4.0
2.0
0.0
1.00 2.00 3.00 4.00 5.00 6.00 7.00 8.00 9.00 10.00 11.00 12.00

4g:

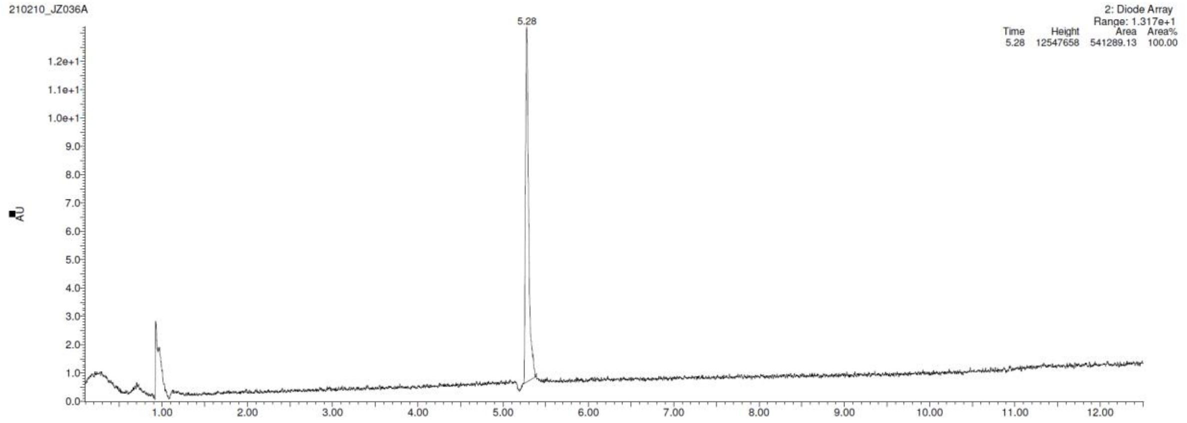
210210_JZ036A
2: Diode Array
Range: 1.317e+1
Time Height Area Area%
5.28 12547658 541289.13 100.00
5.28
AU
1.2e+1
1.1e+1
1.0e+1
9.0
8.0
7.0
6.0
5.0
4.0
3.0
2.0
1.0
0.0
1.00 2.00 3.00 4.00 5.00 6.00 7.00 8.00 9.00 10.00 11.00 12.00

4h:

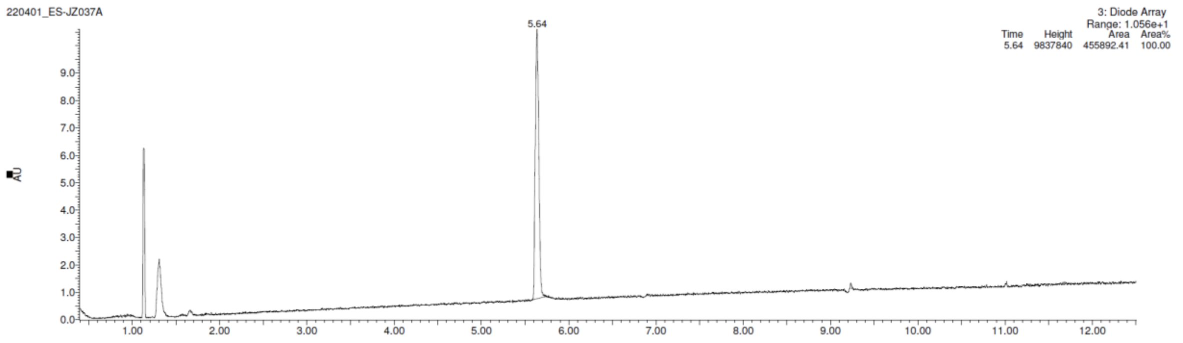
220401_ES-JZ037A
5.64
3: Diode Array
Range: 1.056e+1
Time Height Area Area%
5.64 9837840 455892.41 100.00
AU

5a:

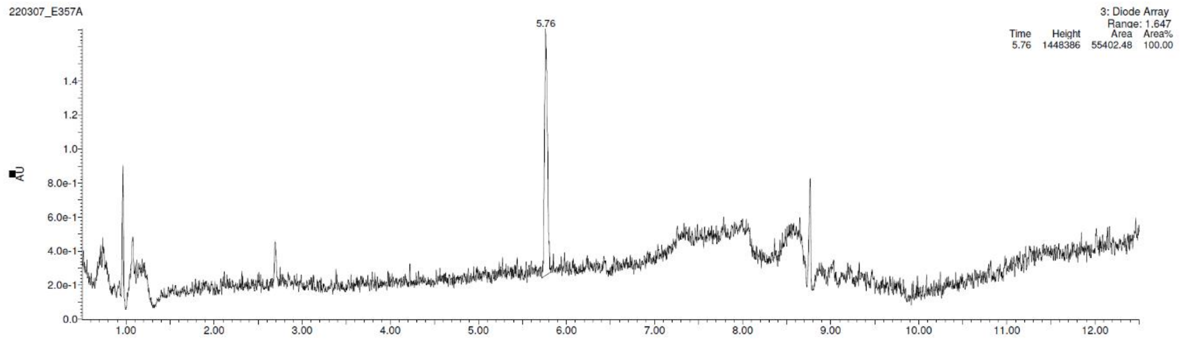
220307_E357A
5.76
3: Diode Array
Range: 1.647
Time Height Area Area%
5.76 1448386 55402.48 100.00
AU

5b:

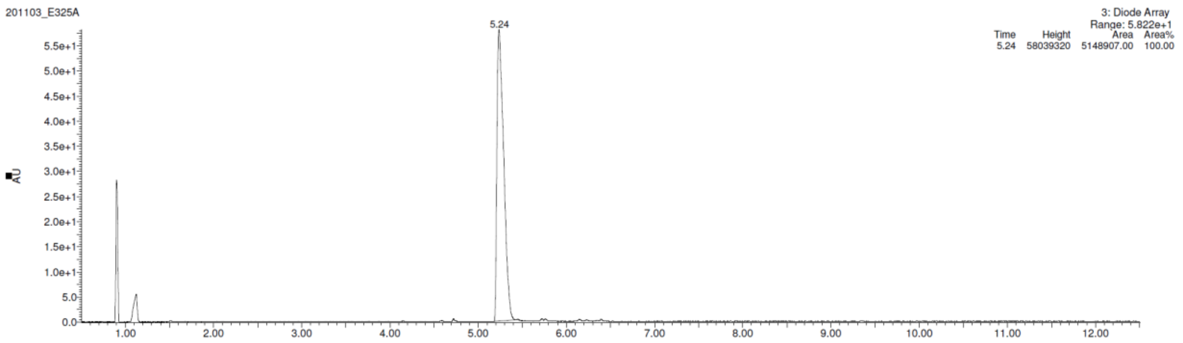
201103_E325A
5.24
3: Diode Array
Range: 5.822e+1
Time Height Area Area%
5.24 58039320 5148907.00 100.00
AU

5c:

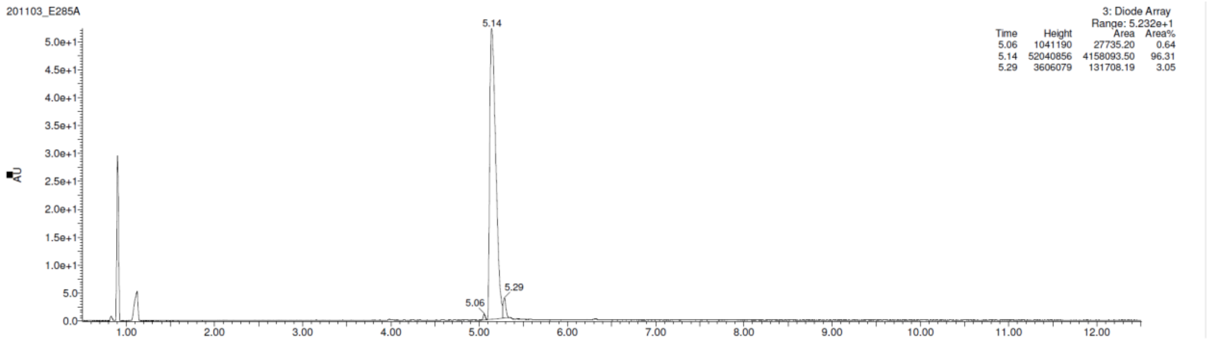
201103_E285A
5.14
5.06
5.29
3: Diode Array
Range: 5.232e+1
Time Height Area Area%
5.06 1041190 27735.20 0.64
5.14 52040856 4158093.50 96.31
5.29 3606079 131708.19 3.05
AU

5d:

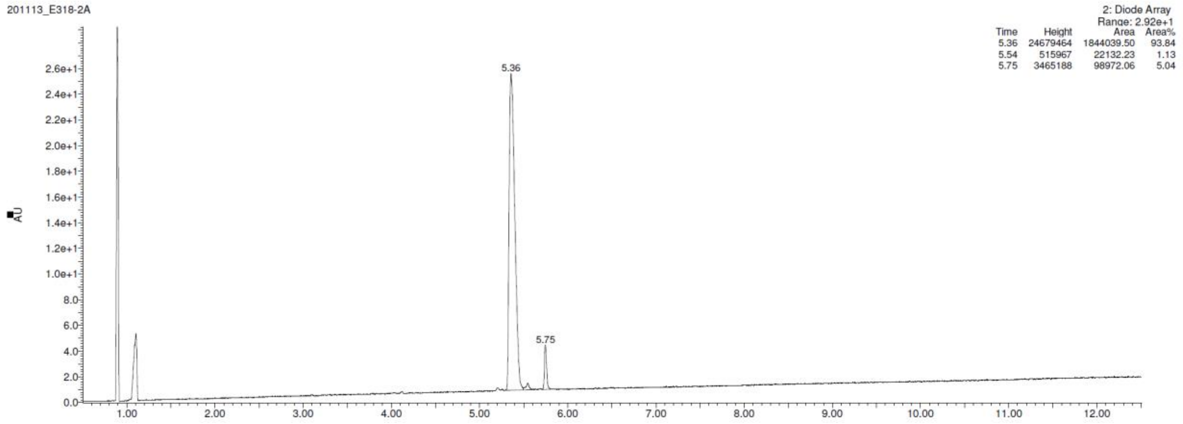
201113_E318-2A
2: Diode Array
Range: 2.92e+1
Time Height Area Area%
5.36 24679464 1844039.50 93.84
5.54 515967 22132.23 1.13
5.75 3465188 98972.06 5.04
5.36
5.75
AU

5e:

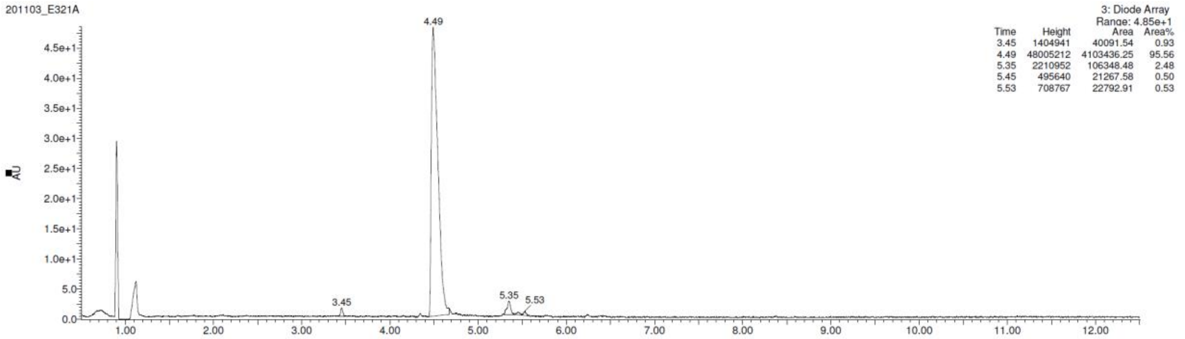
201103_E321A
3: Diode Array
Range: 4.85e+1
Time Height Area Area%
3.45 1404941 40091.54 0.93
4.49 48005212 4103436.25 95.56
5.35 2210952 106348.48 2.48
5.45 495640 21267.58 0.50
5.53 708767 22792.91 0.53
4.49
3.45
5.35
5.53
AU

5f:

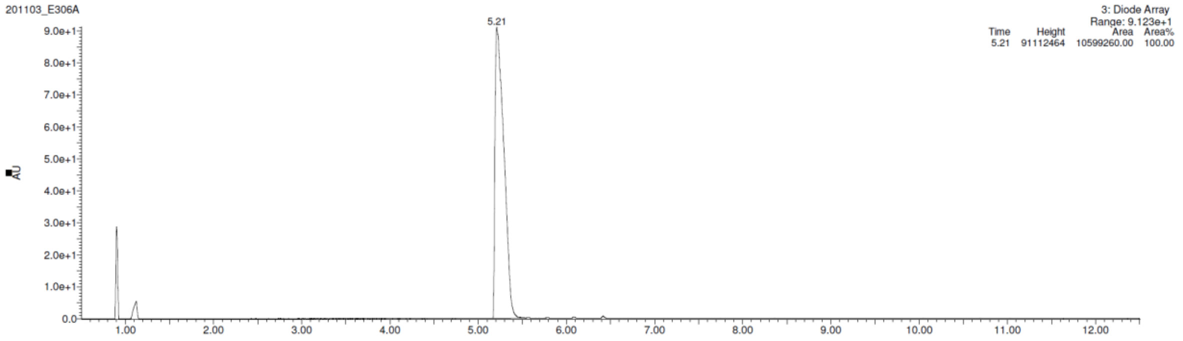
201103_E306A
3: Diode Array
Range: 9.123e+1
Time Height Area Area%
5.21 91112464 10599260.00 100.00
5.21
AU

5g:

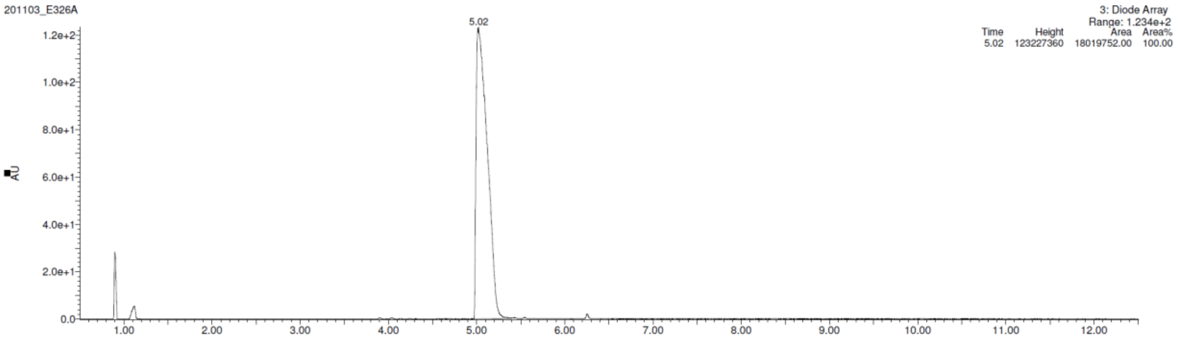
201103_E326A
3: Diode Array
Range: 1.234e+2
Time Height Area Area%
5.02 123227360 18019752.00 100.00
5.02
AU

5h:

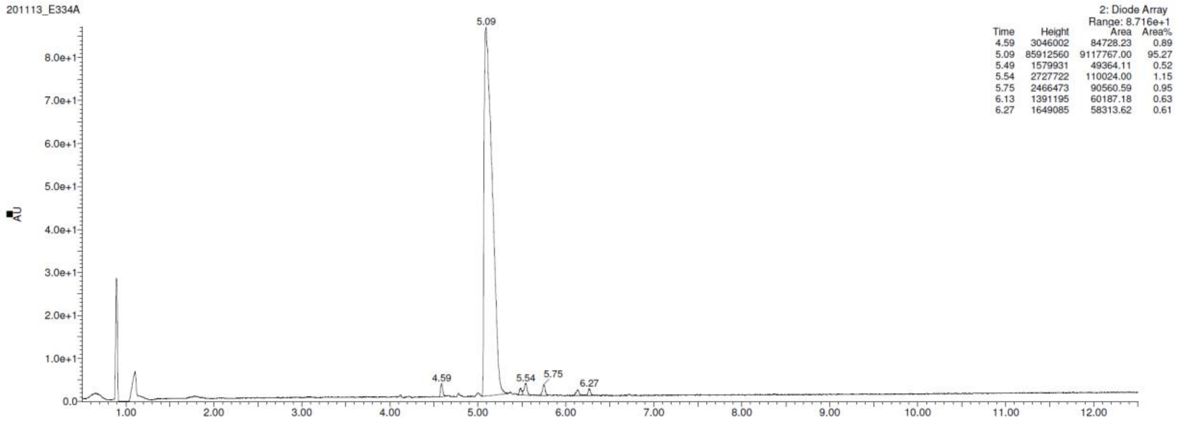
201113_E334A
2: Diode Array
Range: 8.716e+1
Time Height Area Area%
4.59 3046002 84728.23 0.89
5.09 85912560 9117767.00 95.27
5.49 1579931 49364.11 0.52
5.54 2727722 110024.00 1.15
5.75 2466473 90560.59 0.95
6.13 1391195 60187.18 0.63
6.27 1649085 58313.62 0.61
5.09
4.59
5.54
5.75
6.27
AU
8.0e+1
7.0e+1
6.0e+1
5.0e+1
4.0e+1
3.0e+1
2.0e+1
1.0e+1
0.0
1.00
2.00
3.00
4.00
5.00
6.00
7.00
8.00
9.00
10.00
11.00
12.00
