# Supplementary material for: Solubilizer Tag Effect on PD-L1/Inhibitor Binding Properties for m-Terphenyl Derivatives
Source: ACS Med Chem Lett. 2023 Dec 14;15(1):36–44. doi: 10.1021/acsmedchemlett.3c00306 (PMC10788941; doi:10.1021/acsmedchemlett.3c00306)
Supplement: Supplementary file 2 — ml3c00306_si_002.pdf [file ml3c00306_si_002.pdf]

Identifier	PLP.Fitness	Goldscore.Fitness	Goldscore.External.HBond.Weighted	Goldscore.External.HBo
0|BMS1166_ion99|BMS1166_ion99|mol2|1|dock2|1	129.1684	92.9789	4.4747	4.47
1|BMS1166_ion99|BMS1166_ion99|mol2|1|dock4|1	132.6484	94.6995	5.4530	5.45
2|BMS1166_ion99|BMS1166_ion99|mol2|1|dock5|1	119.5771	48.5657	7.0227	7.02
3|BMS1166_neu|BMS1166_neu|mol2|1|dock2|1	92.3351	29.4989	1.8501	1.85
4|BMS1166_neu|BMS1166_neu|mol2|1|dock6|1	128.3935	99.2360	5.3087	5.30
5|BMS1166_neu|BMS1166_neu|mol2|1|dock9|1	129.1092	71.3686	1.2035	1.20
6|DME3_ion42|DME3_ion42|mol2|1|dock2|1	123.6499	59.7962	6.5269	6.52
7|DME3_ion42|DME3_ion42|mol2|1|dock6|1	129.4230	49.6774	4.9862	4.98
8|DME3_ion42|DME3_ion42|mol2|1|dock9|1	122.1635	25.4584	3.5590	3.55
9|DME3_neu58|DME3_neu58|mol2|1|dock2|1	118.2728	59.6648	0.6766	0.67
10|DME3_neu58|DME3_neu58|mol2|1|dock3|1	119.3001	61.6106	2.4073	2.40
11|DME3_neu58|DME3_neu58|mol2|1|dock4|1	127.5056	58.8112	1.1663	1.16
12|E285_ion90|E285_ion90|mol2|1|dock1|1	126.7303	83.4431	7.7425	7.74
13|E285_ion90|E285_ion90|mol2|1|dock6|1	125.7717	82.4954	2.8367	2.83
14|E285_ion90|E285_ion90|mol2|1|dock7|1	124.6835	81.6309	2.4444	2.44
15|E306_ion90|E306_ion90|mol2|1|dock3|1	134.5279	60.1969	6.6517	6.65
16|E306_ion90|E306_ion90|mol2|1|dock8|1	137.3580	40.8238	8.5357	8.53
17|E306_ion90|E306_ion90|mol2|1|dock9|1	134.2218	58.1145	4.9028	4.90
18|E318_ion90|E318_ion90|mol2|1|dock3|1	124.4964	76.3058	4.9822	4.98
19|E318_ion90|E318_ion90|mol2|1|dock4|1	124.6543	81.7032	1.4681	1.46
20|E318_ion90|E318_ion90|mol2|1|dock7|1	125.3189	71.5761	4.9985	4.99
21|E321_ion90|E321_ion90|mol2|1|dock8|1	129.2257	31.4683	2.5860	2.58
22|E321_ion90|E321_ion90|mol2|1|dock9|1	123.4363	31.5298	8.3690	8.36
23|E321_ion90|E321_ion90|mol2|1|dock10|1	122.5480	69.5859	7.3609	7.36
24|E325_ion85|E325_ion85|mol2|1|dock1|1	130.6839	70.3021	4.3974	4.39
25|E325_ion85|E325_ion85|mol2|1|dock8|1	120.4483	57.0403	4.5326	4.53
26|E325_ion85|E325_ion85|mol2|1|dock9|1	120.1608	52.3211	3.7759	3.77
27|E326_ion90|E326_ion90|mol2|1|dock3|1	135.5482	48.8301	8.2713	8.27
28|E326_ion90|E326_ion90|mol2|1|dock7|1	134.7118	73.8679	10.0592	10.05
29|E326_ion90|E326_ion90|mol2|1|dock8|1	132.3545	39.8029	8.0106	8.01
30|E334_ion90|E334_ion90|mol2|1|dock6|1	138.3007	51.9020	5.5162	5.51
31|E334_ion90|E334_ion90|mol2|1|dock7|1	120.1226	67.3654	2.3981	2.39
32|E334_ion90|E334_ion90|mol2|1|dock9|1	123.2705	60.0334	10.8130	10.81
33|E337_ion90|E337_ion90|mol2|1|dock1|1	142.1032	58.8256	22.9899	22.98
34|E337_ion90|E337_ion90|mol2|1|dock3|1	127.4797	61.7821	3.0829	3.08
35|E337_ion90|E337_ion90|mol2|1|dock7|1	143.6826	78.0008	21.1850	21.18
36|GW16_neu70|GW16_neu70|mol2|1|dock4|1	118.1959	68.7396	0.1175	0.11
37|GW16_neu70|GW16_neu70|mol2|1|dock6|1	117.5107	81.1417	0.5283	0.52
38|GW16_neu70|GW16_neu70|mol2|1|dock8|1	119.9321	80.9840	0.1136	0.11
39|GW18_neu67|GW18_neu67|mol2|1|dock5|1	121.8755	54.2593	0.5204	0.52
40|GW18_neu67|GW18_neu67|mol2|1|dock7|1	122.7618	93.3249	0.0386	0.03
41|GW18_neu67|GW18_neu67|mol2|1|dock8|1	119.7052	61.3674	0.1963	0.19
42|GW19_neu64|GW19_neu64|mol2|1|dock3|1	124.3627	87.8470	2.1434	2.14
43|GW19_neu64|GW19_neu64|mol2|1|dock5|1	128.6183	33.5990	6.4281	6.42
44|GW19_neu64|GW19_neu64|mol2|1|dock7|1	124.0113	86.9249	1.9721	1.97
45|GW23_neu74|GW23_neu74|mol2|1|dock2|1	119.7701	30.8399	0.5657	0.56
46|GW23_neu74|GW23_neu74|mol2|1|dock6|1	123.8198	56.1716	0.1884	0.18
47|GW23_neu74|GW23_neu74|mol2|1|dock7|1	119.9821	59.0713	0.1856	0.18
48|GW28_neu68|GW28_neu68|mol2|1|dock2|1	123.0016	55.1349	0.5000	0.50
49|GW28_neu68|GW28_neu68|mol2|1|dock3|1	117.3478	77.7400	3.4827	3.48
50|GW28_neu68|GW28_neu68|mol2|1|dock9|1	126.7320	36.2032	3.5895	3.58
51|GW29_ion46|GW29_ion46|mol2|1|dock2|1	121.6304	59.5759	3.0860	3.08
52|GW29_ion46|GW29_ion46|mol2|1|dock4|1	122.4859	65.0489	6.4886	6.48
53|GW29_ion46|GW29_ion46|mol2|1|dock9|1	126.1147	27.4088	1.5000	1.50
54|GW29_ion52|GW29_ion52|mol2|1|dock1|1	121.5653	49.9494	0.1550	0.15
55|GW29_ion52|GW29_ion52|mol2|1|dock6|1	98.0488	11.1941	5.3900	5.39
56|GW29_ion52|GW29_ion52|mol2|1|dock8|1	121.9876	35.6594	2.8377	2.83
57|GW31_ion96|GW31_ion96|mol2|1|dock5|1	124.3241	79.5866	4.8966	4.89
58|GW31_ion96|GW31_ion96|mol2|1|dock7|1	119.9892	65.3936	1.0451	1.04
59|GW31_ion96|GW31_ion96|mol2|1|dock10|1	124.6594	72.4151	5.9023	5.90
60|GW33_ion95|GW33_ion95|mol2|1|dock2|1	124.0346	60.1341	5.9253	5.92
61|GW33_ion95|GW33_ion95|mol2|1|dock7|1	125.4256	62.8863	0.6796	0.67
62|GW33_ion95|GW33_ion95|mol2|1|dock8|1	125.4356	64.5642	0.1225	0.12
63|GW34_ion95|GW34_ion95|mol2|1|dock3|1	124.8725	40.4487	2.1125	2.11

<table>
<tr><td>64|GW34_ion95|GW34_ion95|mol2|1|dock6|1</td><td>138.6404</td><td>64.7595</td><td>5.9419</td><td>5.94</td></tr>
<tr><td>65|GW34_ion95|GW34_ion95|mol2|1|dock9|1</td><td>135.4053</td><td>76.2949</td><td>1.8121</td><td>1.81</td></tr>
<tr><td>66|GW36_ion87|GW36_ion87|mol2|1|dock1|1</td><td>127.2618</td><td>60.3036</td><td>4.1197</td><td>4.11</td></tr>
<tr><td>67|GW36_ion87|GW36_ion87|mol2|1|dock2|1</td><td>119.2014</td><td>62.0969</td><td>0.7137</td><td>0.71</td></tr>
<tr><td>68|GW36_ion87|GW36_ion87|mol2|1|dock3|1</td><td>125.7458</td><td>36.5717</td><td>7.6864</td><td>7.68</td></tr>
<tr><td>69|GW38_ion96|GW38_ion96|mol2|1|dock2|1</td><td>129.1458</td><td>77.1579</td><td>0.0000</td><td>0.00</td></tr>
<tr><td>70|GW38_ion96|GW38_ion96|mol2|1|dock3|1</td><td>126.8423</td><td>76.6345</td><td>0.0235</td><td>0.02</td></tr>
<tr><td>71|GW38_ion96|GW38_ion96|mol2|1|dock8|1</td><td>128.3116</td><td>30.6122</td><td>6.4544</td><td>6.45</td></tr>
<tr><td>72|GW41_ion94|GW41_ion94|mol2|1|dock3|1</td><td>88.4481</td><td>32.4032</td><td>0.1220</td><td>0.12</td></tr>
<tr><td>73|GW41_ion94|GW41_ion94|mol2|1|dock6|1</td><td>116.0075</td><td>57.7216</td><td>2.6453</td><td>2.64</td></tr>
<tr><td>74|GW41_ion94|GW41_ion94|mol2|1|dock8|1</td><td>87.9491</td><td>16.8846</td><td>0.3334</td><td>0.33</td></tr>
<tr><td>75|GW42_ion94|GW42_ion94|mol2|1|dock2|1</td><td>131.3214</td><td>53.6021</td><td>0.6712</td><td>0.67</td></tr>
<tr><td>76|GW42_ion94|GW42_ion94|mol2|1|dock5|1</td><td>122.1195</td><td>60.6616</td><td>2.2224</td><td>2.22</td></tr>
<tr><td>77|GW42_ion94|GW42_ion94|mol2|1|dock8|1</td><td>127.5379</td><td>56.4504</td><td>0.5455</td><td>0.54</td></tr>
<tr><td>78|GW43_ion41|GW43_ion41|mol2|1|dock1|1</td><td>117.0526</td><td>89.5817</td><td>4.9811</td><td>4.98</td></tr>
<tr><td>79|GW43_ion41|GW43_ion41|mol2|1|dock3|1</td><td>117.3140</td><td>77.2603</td><td>1.1742</td><td>1.17</td></tr>
<tr><td>80|GW43_ion41|GW43_ion41|mol2|1|dock4|1</td><td>117.8517</td><td>90.2195</td><td>4.0551</td><td>4.05</td></tr>
<tr><td>81|GW43_ion59|GW43_ion59|mol2|1|dock3|1</td><td>117.6610</td><td>58.2970</td><td>5.7770</td><td>5.77</td></tr>
<tr><td>82|GW43_ion59|GW43_ion59|mol2|1|dock4|1</td><td>116.8519</td><td>87.2361</td><td>8.4904</td><td>8.49</td></tr>
<tr><td>83|GW43_ion59|GW43_ion59|mol2|1|dock6|1</td><td>116.6843</td><td>86.9026</td><td>9.5905</td><td>9.59</td></tr>
<tr><td>84|GW44_ion98|GW44_ion98|mol2|1|dock2|1</td><td>119.7230</td><td>60.5949</td><td>1.7968</td><td>1.79</td></tr>
<tr><td>85|GW44_ion98|GW44_ion98|mol2|1|dock3|1</td><td>119.2724</td><td>68.4064</td><td>0.7682</td><td>0.76</td></tr>
<tr><td>86|GW44_ion98|GW44_ion98|mol2|1|dock5|1</td><td>117.5588</td><td>64.1885</td><td>7.4204</td><td>7.42</td></tr>
<tr><td>87|JZ027_ion95|JZ027_ion95|mol2|1|dock6|1</td><td>124.9229</td><td>85.5169</td><td>8.8001</td><td>8.80</td></tr>
<tr><td>88|JZ027_ion95|JZ027_ion95|mol2|1|dock7|1</td><td>124.6783</td><td>73.0244</td><td>7.8011</td><td>7.80</td></tr>
<tr><td>89|JZ027_ion95|JZ027_ion95|mol2|1|dock9|1</td><td>127.1279</td><td>75.6731</td><td>6.3871</td><td>6.38</td></tr>
<tr><td>90|JZ028_neu75|JZ028_neu75|mol2|1|dock3|1</td><td>120.1649</td><td>59.4440</td><td>1.1060</td><td>1.10</td></tr>
<tr><td>91|JZ028_neu75|JZ028_neu75|mol2|1|dock6|1</td><td>121.1528</td><td>66.4638</td><td>1.2122</td><td>1.21</td></tr>
<tr><td>92|JZ028_neu75|JZ028_neu75|mol2|1|dock8|1</td><td>120.6681</td><td>82.8073</td><td>1.8605</td><td>1.86</td></tr>
<tr><td>93|JZ030_ion97|JZ030_ion97|mol2|1|dock1|1</td><td>129.9620</td><td>89.4660</td><td>10.2511</td><td>10.25</td></tr>
<tr><td>94|JZ030_ion97|JZ030_ion97|mol2|1|dock3|1</td><td>129.3250</td><td>92.9382</td><td>10.2390</td><td>10.23</td></tr>
<tr><td>95|JZ030_ion97|JZ030_ion97|mol2|1|dock4|1</td><td>128.8830</td><td>75.3331</td><td>8.5187</td><td>8.51</td></tr>
<tr><td>96|JZ031_neu65|JZ031_neu65|mol2|1|dock2|1</td><td>131.7490</td><td>75.5553</td><td>1.0057</td><td>1.00</td></tr>
<tr><td>97|JZ031_neu65|JZ031_neu65|mol2|1|dock6|1</td><td>128.3324</td><td>51.3223</td><td>0.8521</td><td>0.85</td></tr>
<tr><td>98|JZ031_neu65|JZ031_neu65|mol2|1|dock8|1</td><td>130.2662</td><td>75.1475</td><td>0.0000</td><td>0.00</td></tr>
<tr><td>99|JZ032_neu71|JZ032_neu71|mol2|1|dock4|1</td><td>120.5520</td><td>36.9052</td><td>0.4291</td><td>0.42</td></tr>
<tr><td>100|JZ032_neu71|JZ032_neu71|mol2|1|dock6|1</td><td>134.1958</td><td>73.3919</td><td>2.8715</td><td>2.87</td></tr>
<tr><td>101|JZ032_neu71|JZ032_neu71|mol2|1|dock10|1</td><td>115.7378</td><td>78.7286</td><td>1.0729</td><td>1.07</td></tr>
<tr><td>102|JZ033_neu74|JZ033_neu74|mol2|1|dock5|1</td><td>135.7525</td><td>84.4705</td><td>4.2566</td><td>4.25</td></tr>
<tr><td>103|JZ033_neu74|JZ033_neu74|mol2|1|dock6|1</td><td>129.0442</td><td>65.0067</td><td>3.8511</td><td>3.85</td></tr>
<tr><td>104|JZ033_neu74|JZ033_neu74|mol2|1|dock7|1</td><td>126.8656</td><td>67.8722</td><td>5.0382</td><td>5.03</td></tr>
<tr><td>105|JZ034_ion85|JZ034_ion85|mol2|1|dock4|1</td><td>130.0978</td><td>73.4046</td><td>8.7391</td><td>8.73</td></tr>
<tr><td>106|JZ034_ion85|JZ034_ion85|mol2|1|dock6|1</td><td>126.8808</td><td>72.2204</td><td>4.9855</td><td>4.98</td></tr>
<tr><td>107|JZ034_ion85|JZ034_ion85|mol2|1|dock8|1</td><td>129.9576</td><td>59.1571</td><td>6.7719</td><td>6.77</td></tr>
<tr><td>108|JZ035_ion95|JZ035_ion95|mol2|1|dock5|1</td><td>134.9680</td><td>75.2872</td><td>12.1242</td><td>12.12</td></tr>
<tr><td>109|JZ035_ion95|JZ035_ion95|mol2|1|dock7|1</td><td>137.9542</td><td>74.8779</td><td>12.7768</td><td>12.77</td></tr>
<tr><td>110|JZ035_ion95|JZ035_ion95|mol2|1|dock8|1</td><td>134.4999</td><td>65.4022</td><td>7.2734</td><td>7.27</td></tr>
<tr><td>111|JZ036_ion94|JZ036_ion94|mol2|1|dock4|1</td><td>140.0723</td><td>82.1493</td><td>5.7006</td><td>5.70</td></tr>
<tr><td>112|JZ036_ion94|JZ036_ion94|mol2|1|dock5|1</td><td>140.5858</td><td>87.5189</td><td>6.2768</td><td>6.27</td></tr>
<tr><td>113|JZ036_ion94|JZ036_ion94|mol2|1|dock8|1</td><td>133.1403</td><td>52.2540</td><td>10.3710</td><td>10.37</td></tr>
<tr><td>114|JZ037_ion93|JZ037_ion93|mol2|1|dock1|1</td><td>125.8312</td><td>62.3656</td><td>3.7597</td><td>3.75</td></tr>
<tr><td>115|JZ037_ion93|JZ037_ion93|mol2|1|dock5|1</td><td>126.5616</td><td>78.2235</td><td>5.1637</td><td>5.16</td></tr>
<tr><td>116|JZ037_ion93|JZ037_ion93|mol2|1|dock10|1</td><td>119.2552</td><td>52.7523</td><td>0.5531</td><td>0.55</td></tr>
<tr><td>117|JZ039_ion93|JZ039_ion93|mol2|1|dock3|1</td><td>132.5609</td><td>70.8638</td><td>4.7437</td><td>4.74</td></tr>
<tr><td>118|JZ039_ion93|JZ039_ion93|mol2|1|dock5|1</td><td>135.5330</td><td>93.1216</td><td>5.3477</td><td>5.34</td></tr>
<tr><td>119|JZ039_ion93|JZ039_ion93|mol2|1|dock7|1</td><td>137.8770</td><td>83.5626</td><td>3.5754</td><td>3.57</td></tr>
<tr><td>120|JZ046_neu73|JZ046_neu73|mol2|1|dock1|1</td><td>127.7033</td><td>75.0509</td><td>1.2775</td><td>1.27</td></tr>
<tr><td>121|JZ046_neu73|JZ046_neu73|mol2|1|dock6|1</td><td>127.9903</td><td>61.1907</td><td>0.7874</td><td>0.78</td></tr>
<tr><td>122|JZ046_neu73|JZ046_neu73|mol2|1|dock10|1</td><td>129.1917</td><td>68.8392</td><td>0.5488</td><td>0.54</td></tr>
<tr><td>123|JZ059_ion48|JZ059_ion48|mol2|1|dock2|1</td><td>120.6605</td><td>50.4112</td><td>0.8486</td><td>0.84</td></tr>
<tr><td>124|JZ059_ion48|JZ059_ion48|mol2|1|dock3|1</td><td>121.0647</td><td>72.4204</td><td>1.8999</td><td>1.89</td></tr>
<tr><td>125|JZ059_ion48|JZ059_ion48|mol2|1|dock7|1</td><td>119.9493</td><td>55.4063</td><td>0.3403</td><td>0.34</td></tr>
<tr><td>126|JZ059_ion52|JZ059_ion52|mol2|1|dock1|1</td><td>126.1765</td><td>80.9280</td><td>3.9504</td><td>3.95</td></tr>
<tr><td>127|JZ059_ion52|JZ059_ion52|mol2|1|dock4|1</td><td>124.5513</td><td>84.1332</td><td>3.7587</td><td>3.75</td></tr>
<tr><td>128|JZ059_ion52|JZ059_ion52|mol2|1|dock6|1</td><td>124.2562</td><td>77.6808</td><td>1.6191</td><td>1.61</td></tr>
</table>

**129|R81_compA_ion|compA_ion70|mol2|1|dock4|1**	146.6772	67.8493	11.2742	11.27
**130|R81_compA_ion|compA_ion70|mol2|1|dock7|1**	146.3123	64.6964	15.8044	15.80
**131|R81_compA_ion|compA_ion70|mol2|1|dock10|1**	141.7588	95.3464	17.9038	17.90
**132|R81_compA_neutr|compA_neutr|mol2|1|dock1|1**	110.4924	48.3426	2.7241	2.72
**133|R81_compA_neutr|compA_neutr|mol2|1|dock8|1**	111.2601	50.3935	3.2296	3.22
**134|R81_compA_neutr|compA_neutr|mol2|1|dock10|1**	108.4200	56.3859	5.0293	5.02
**135|lig_native_ion59_7NLD|lig_native_ion59|mol2|1|dock2|1**	121.2434	87.4604	14.3038	14.30
**136|lig_native_ion59_7NLD|lig_native_ion59|mol2|1|dock3|1**	126.9200	92.6785	13.1279	13.12
**137|lig_native_ion59_7NLD|lig_native_ion59|mol2|1|dock4|1**	126.5225	91.0118	8.1514	8.15
**138|lig_native_neu41_7NLD|lig_native_neu41|mol2|1|dock3|1**	118.2694	89.8441	12.3496	12.34
**139|lig_native_neu41_7NLD|lig_native_neu41|mol2|1|dock4|1**	120.0824	74.1005	10.1230	10.12
**140|lig_native_neu41_7NLD|lig_native_neu41|mol2|1|dock6|1**	120.8597	94.8112	13.3168	13.31
